# Supplementary material for: Time-Varying Gene Expression Network Analysis Reveals Conserved Transition States in Hematopoietic Differentiation between Human and Mouse
Source: Genes (Basel). 2022 Oct 18;13(10):1890. doi: 10.3390/genes13101890 (PMC9601530; doi:10.3390/genes13101890)

mouse_HSCtoMEP_time 1

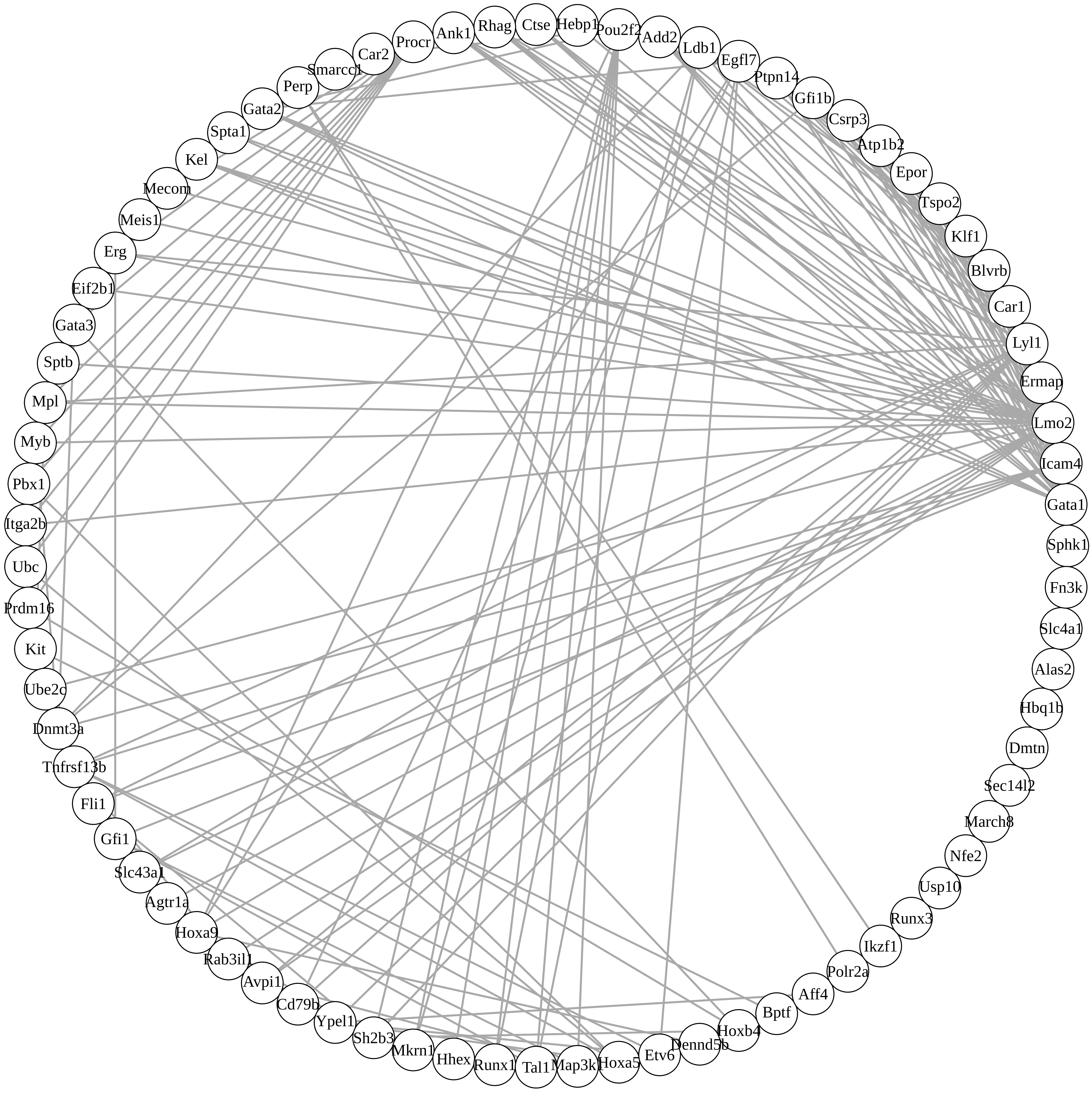

mouse_HSCtoMEP_time 2

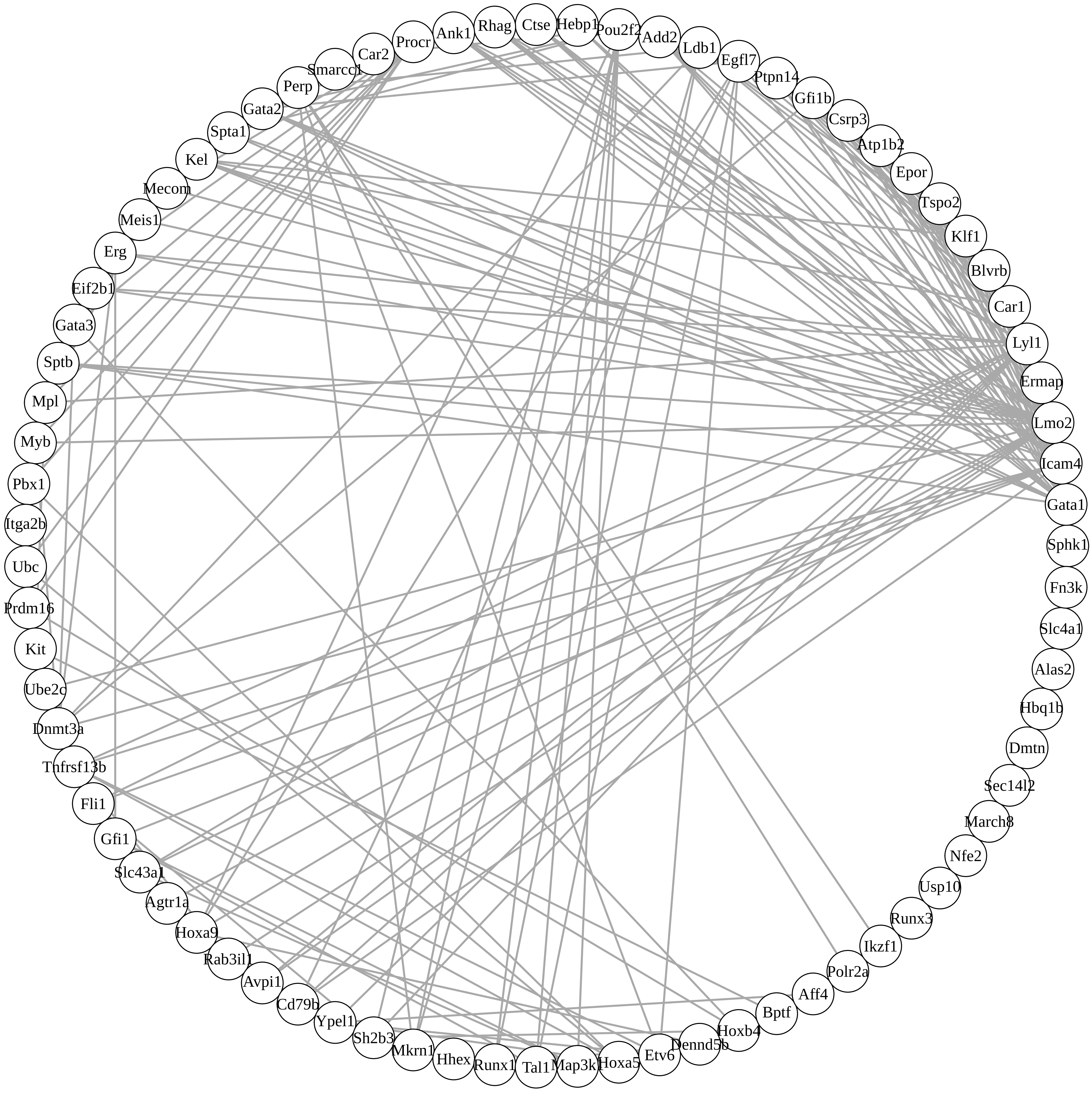

mouse_HSCtoMEP_time 3

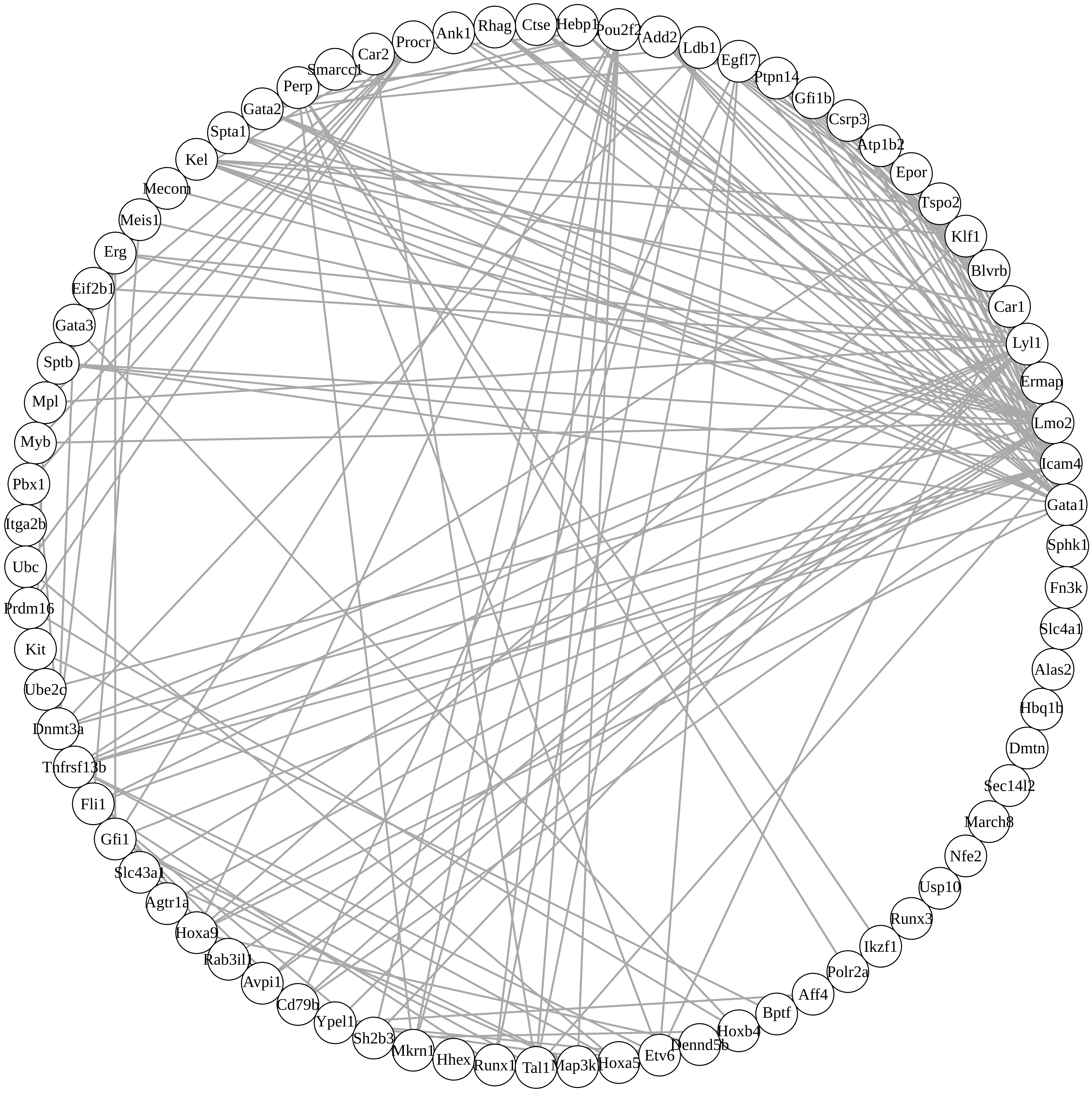

mouse_HSCtoMEP_time 4

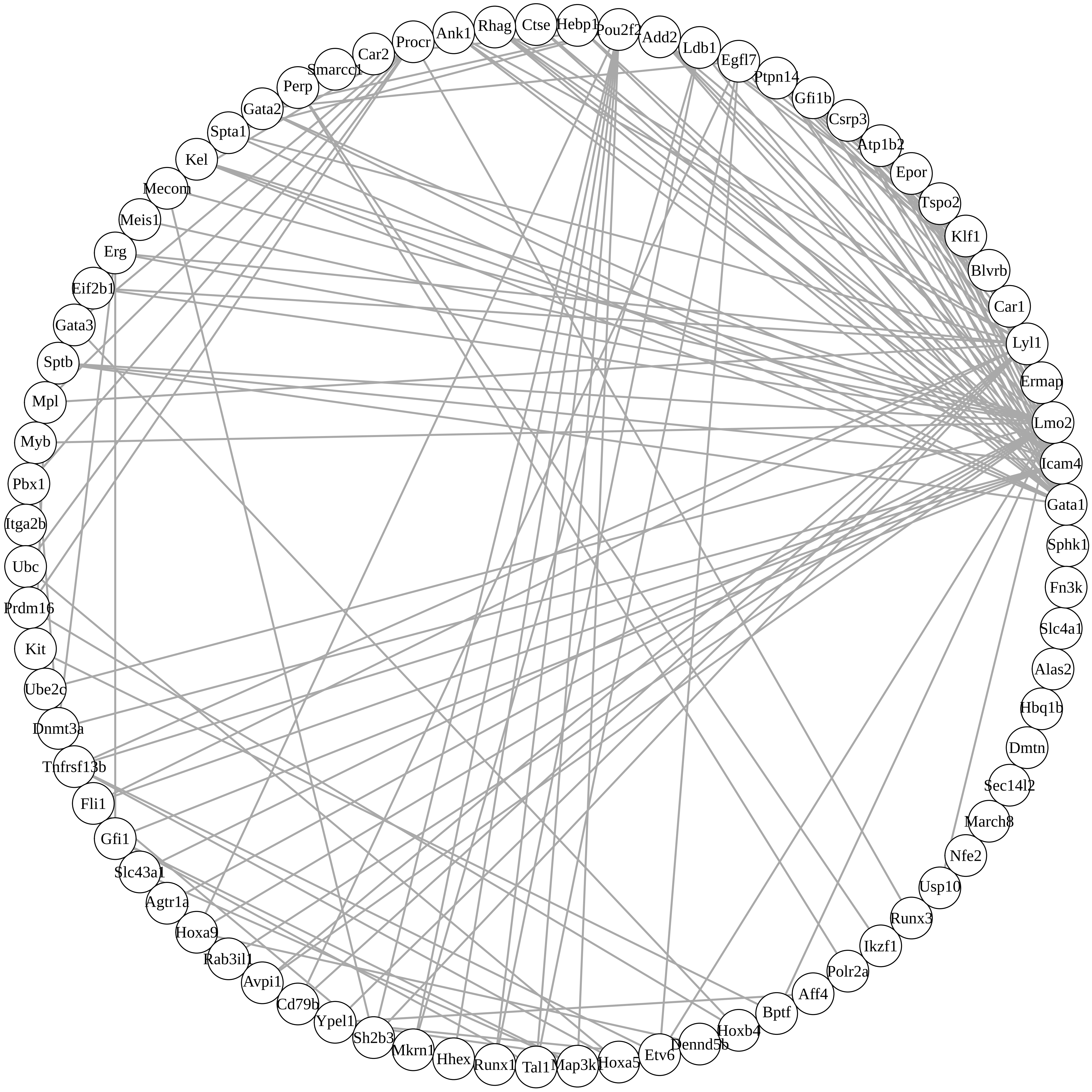

mouse_HSCtoMEP_time 5

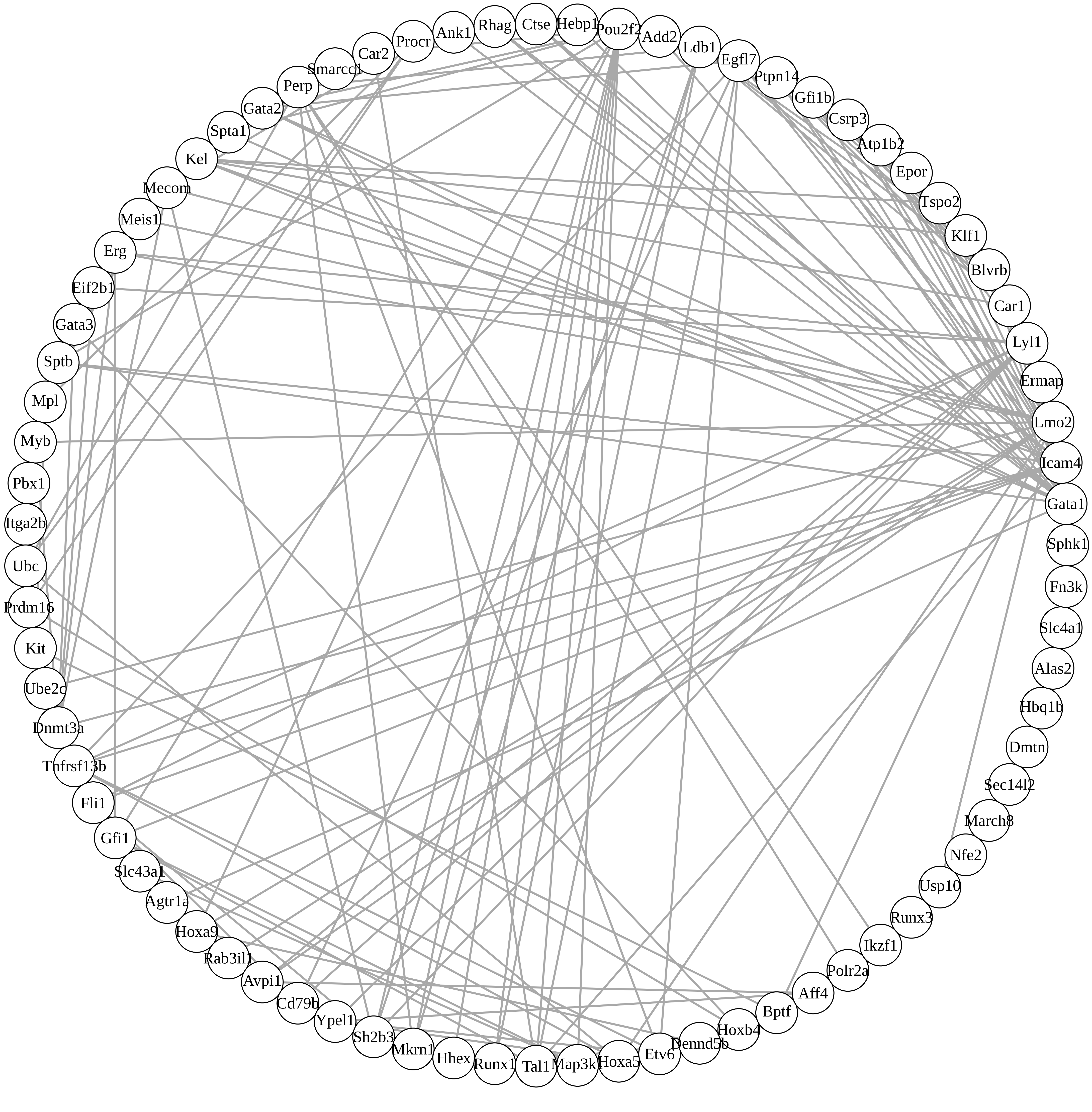

mouse_HSCtoMEP_time 6

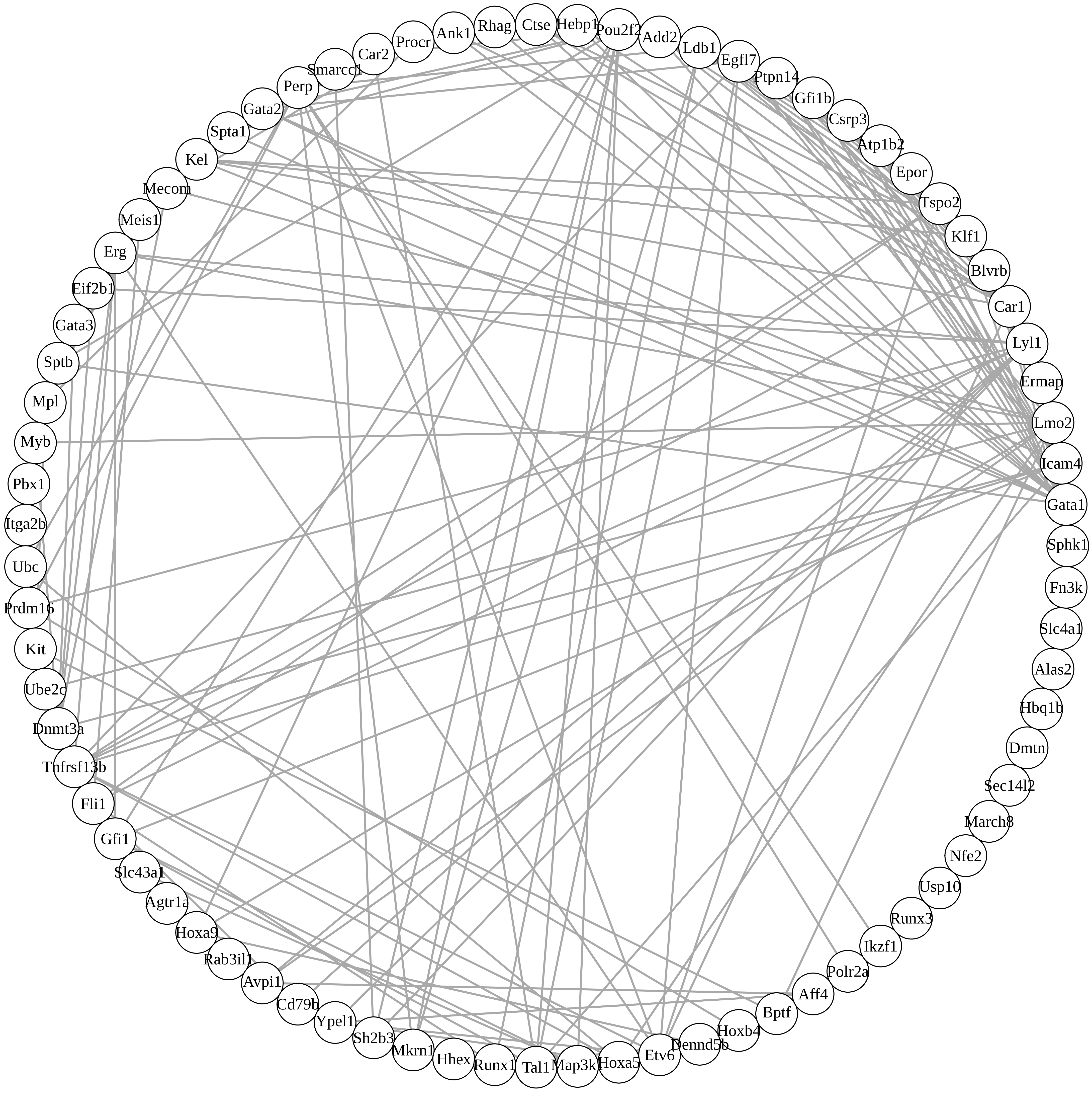

mouse_HSCtoMEP_time 7

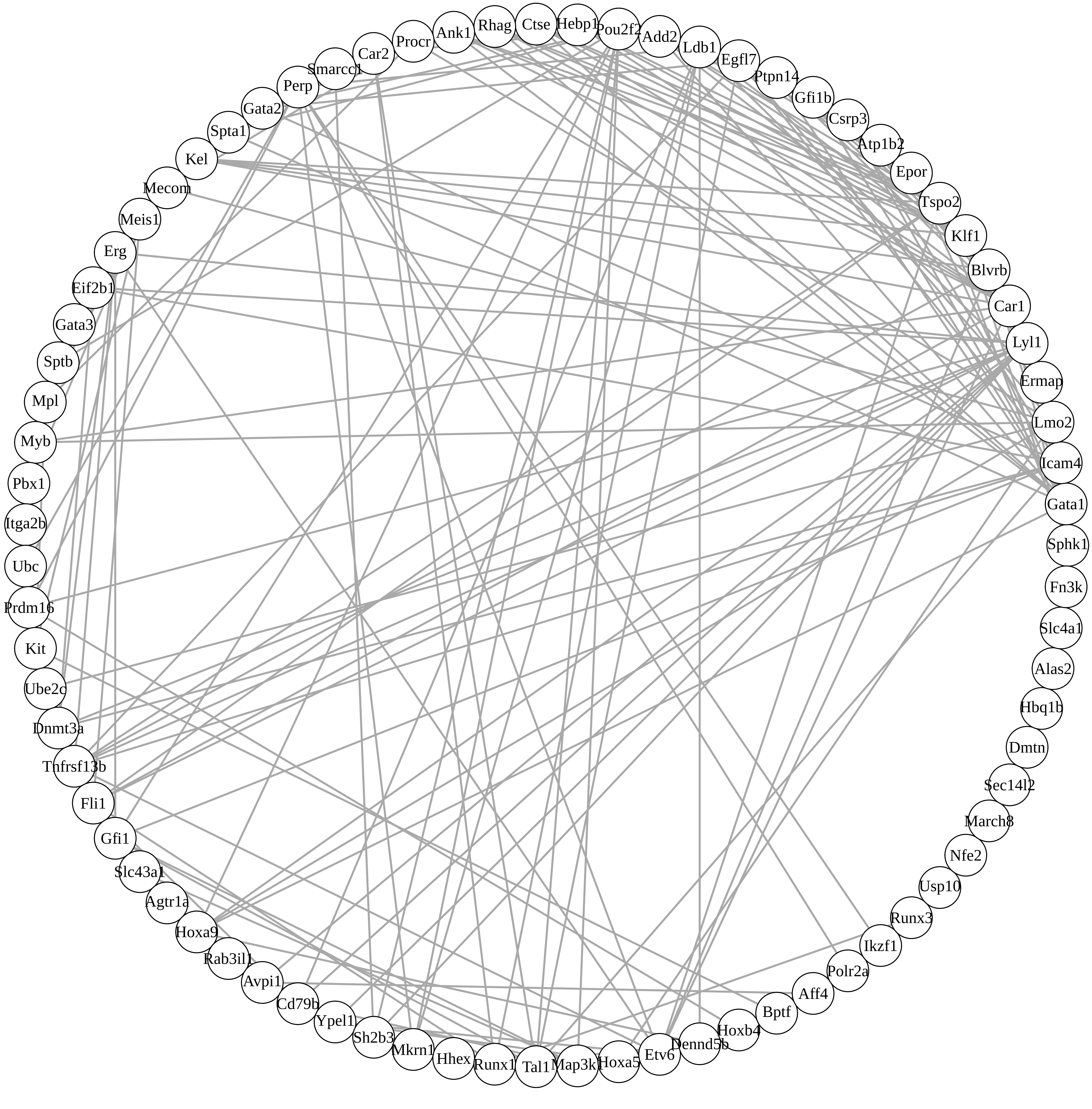

mouse_HSCtoMEP_time 8

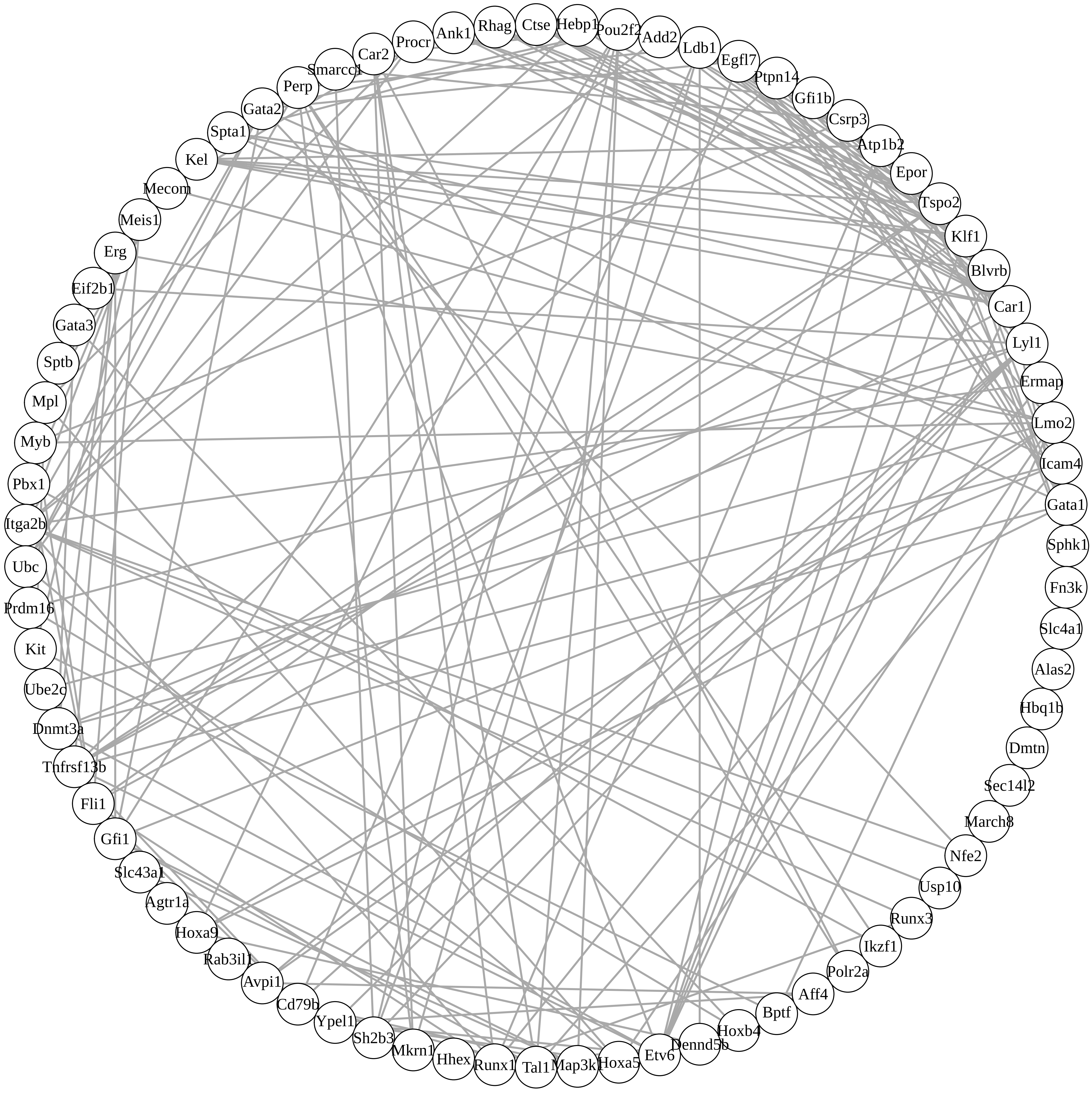

mouse_HSCtoMEP_time 9

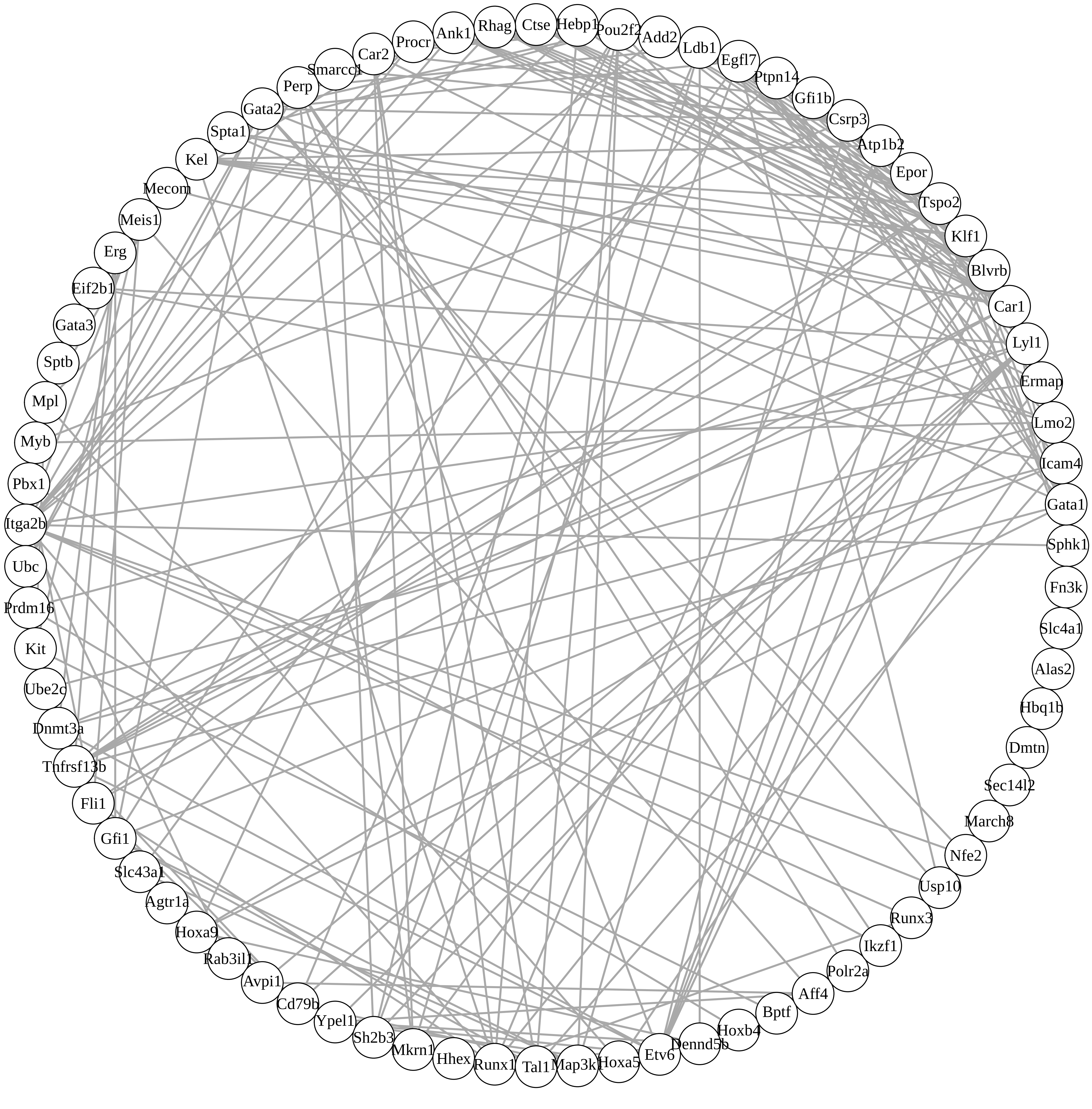

mouse_HSCtoMEP_time 10

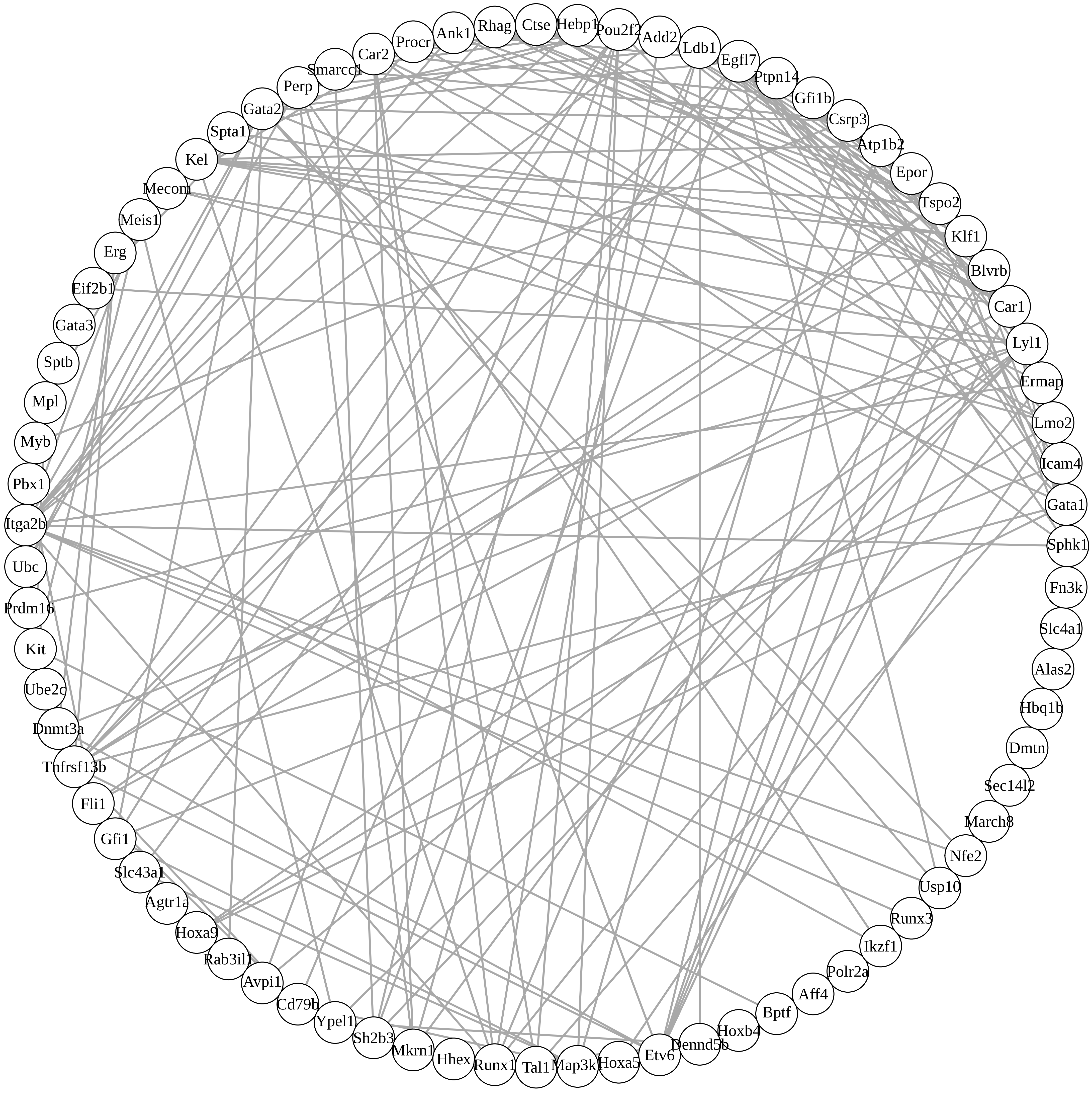

mouse_HSCtoMEP_time 11

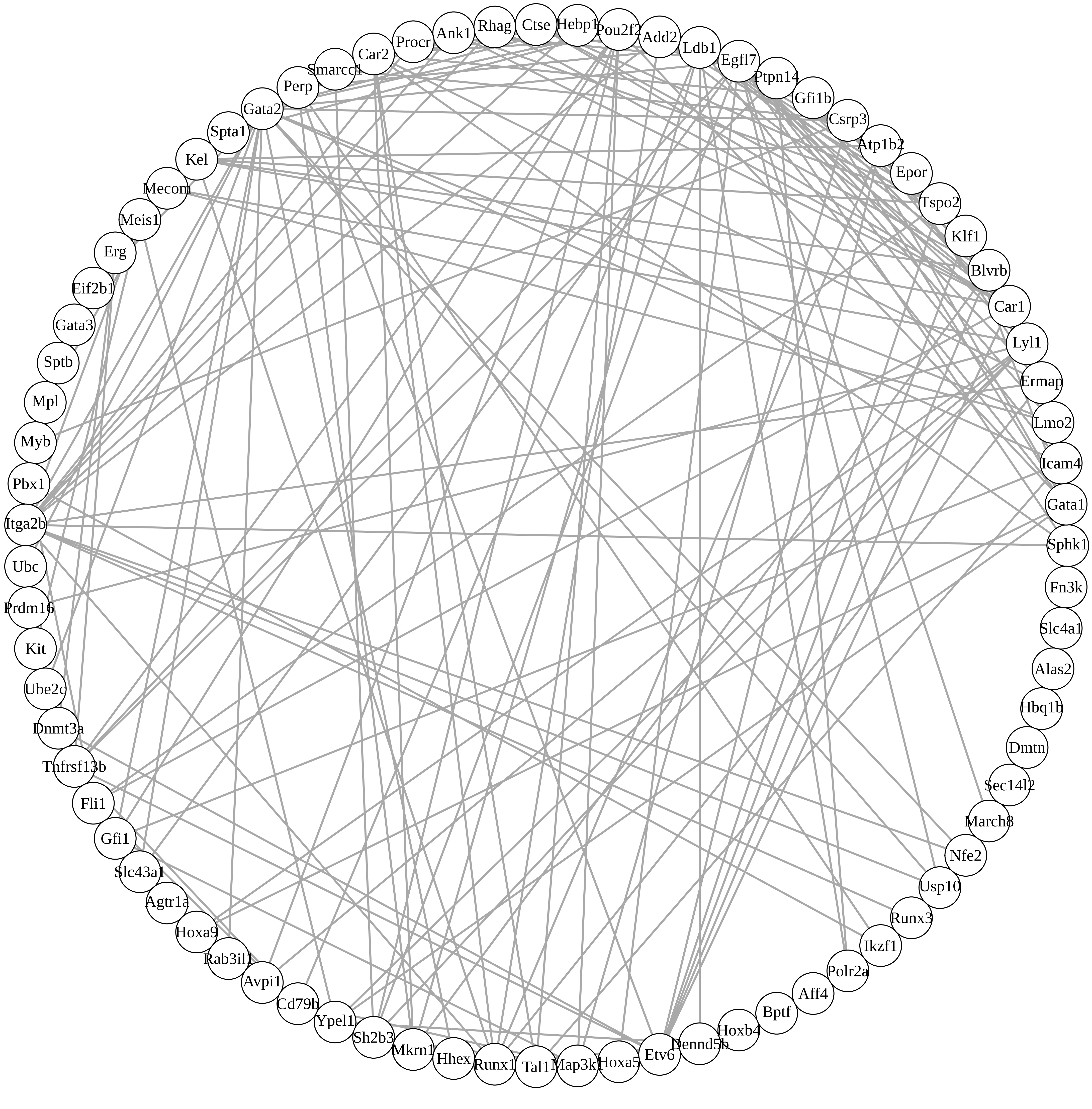

mouse_HSCtoMEP_time 12

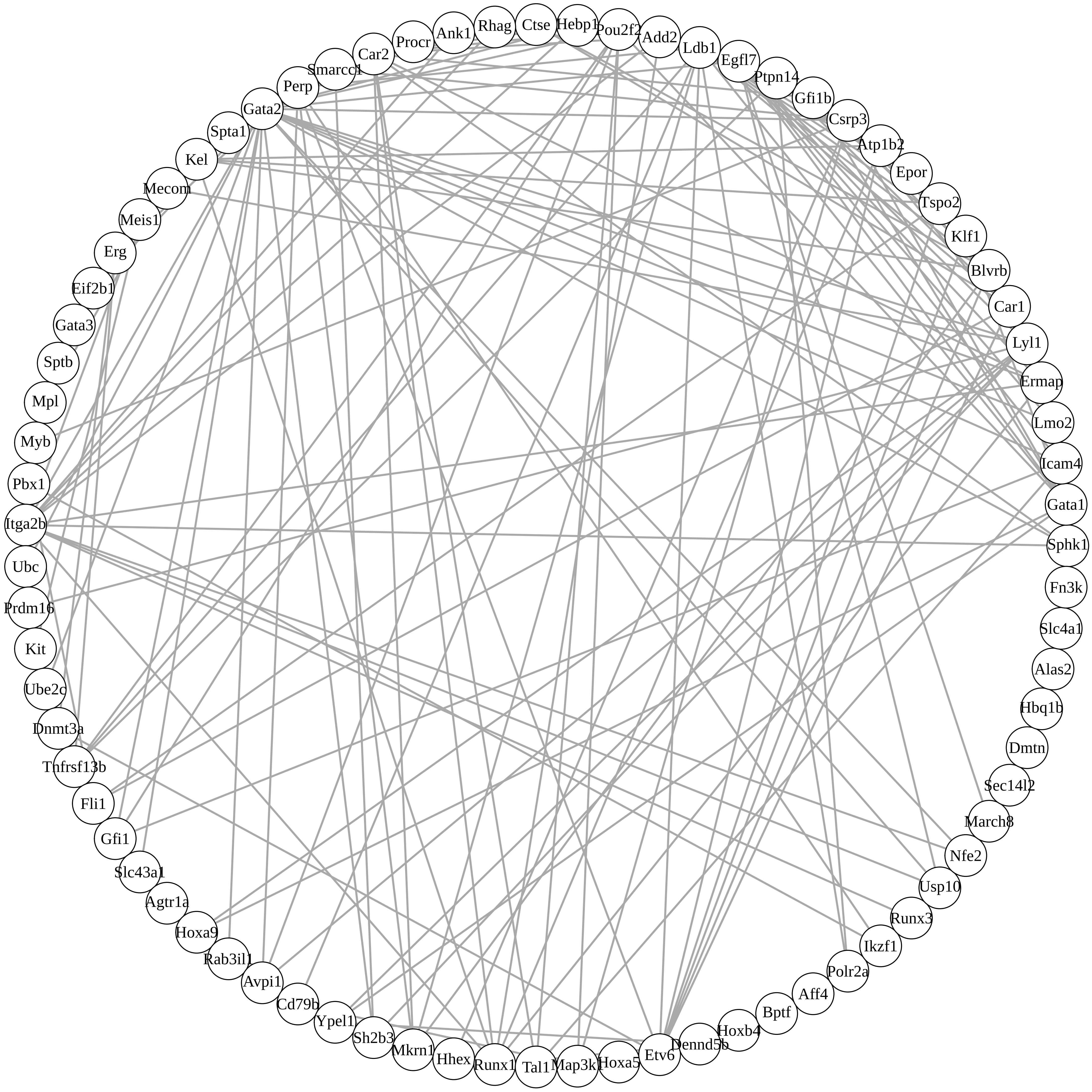

mouse_HSCtoMEP_time 13

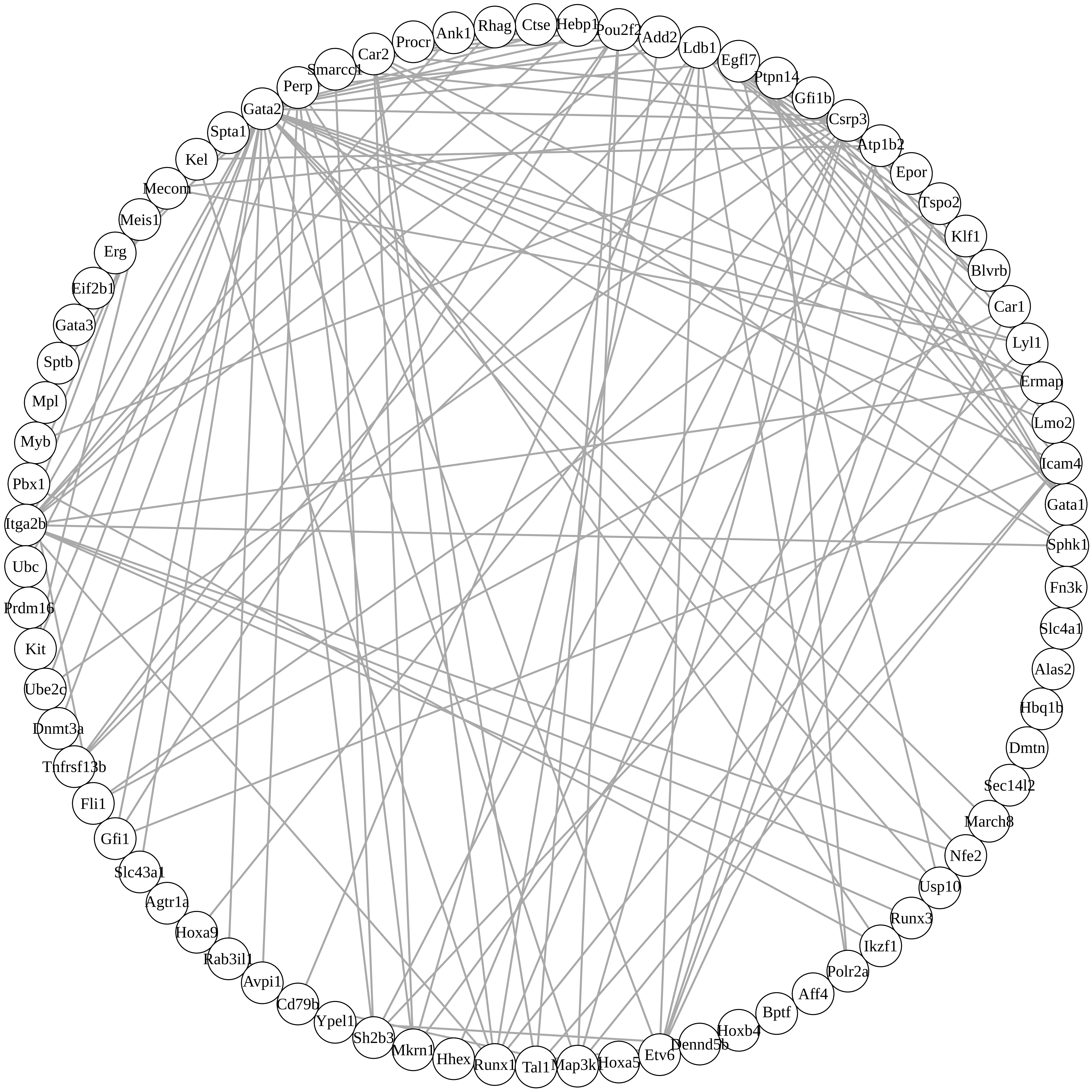

mouse_HSCtoMEP_time 14

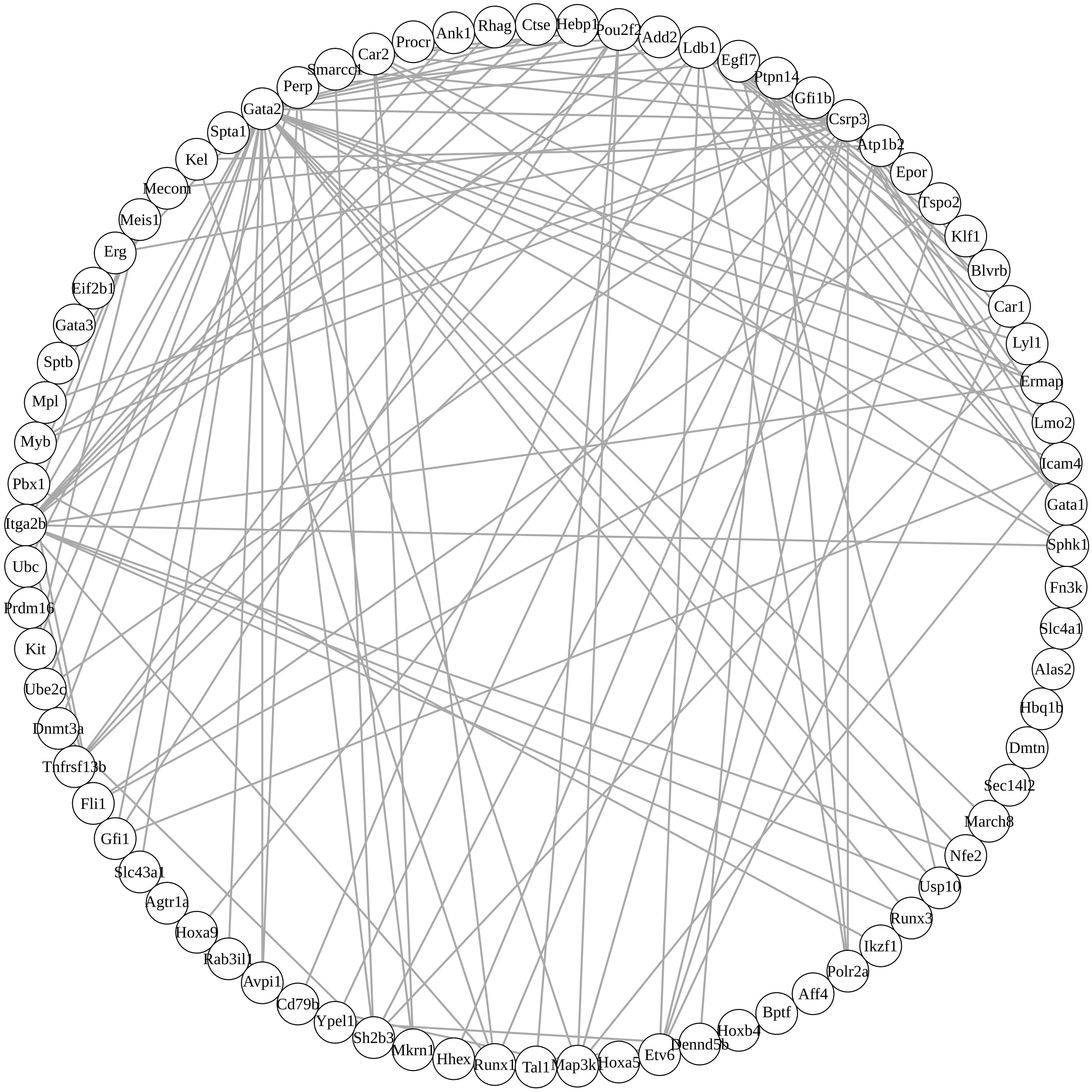

mouse_HSCtoMEP_time 15

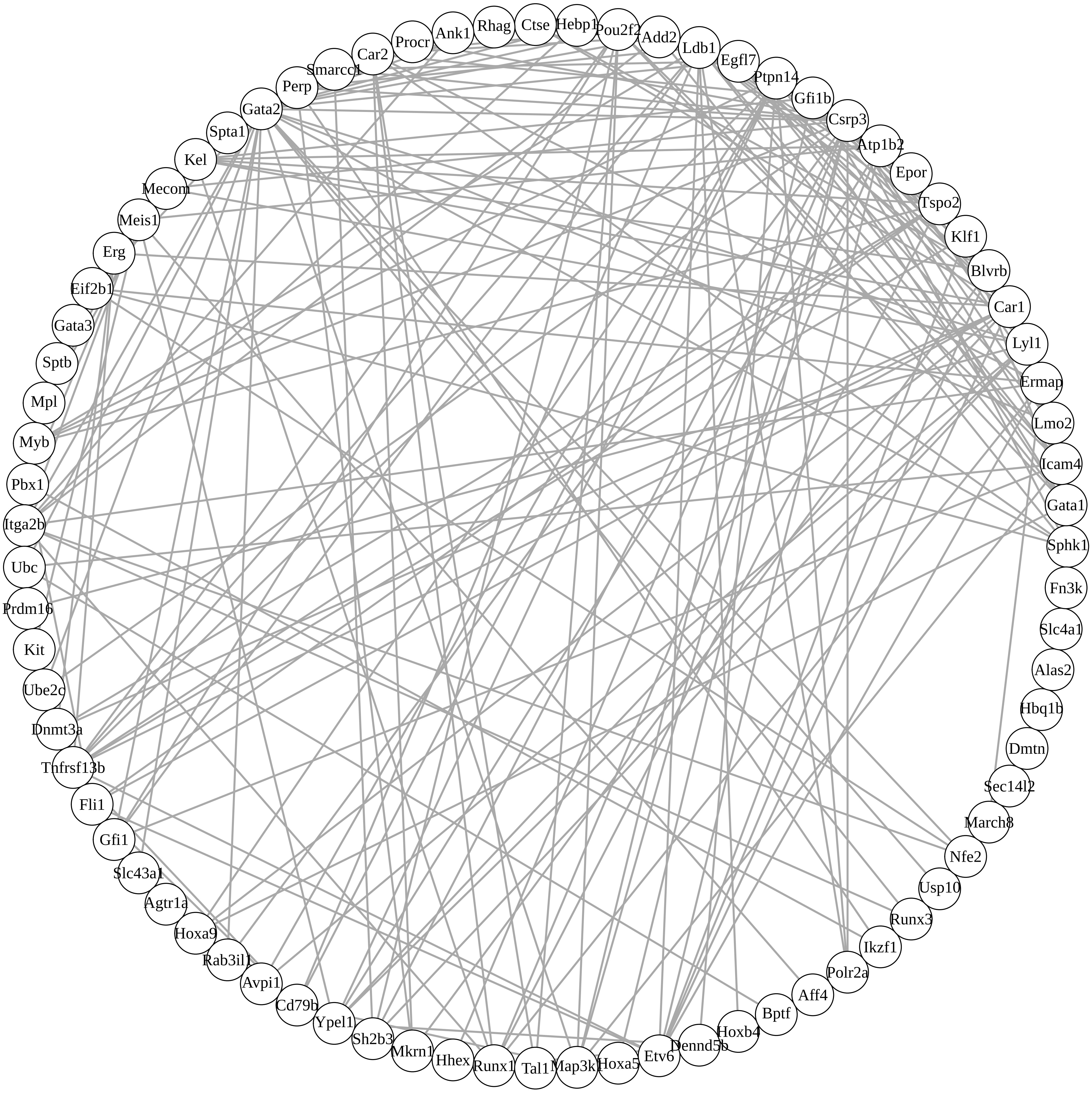

mouse_HSCtoMEP_time 16

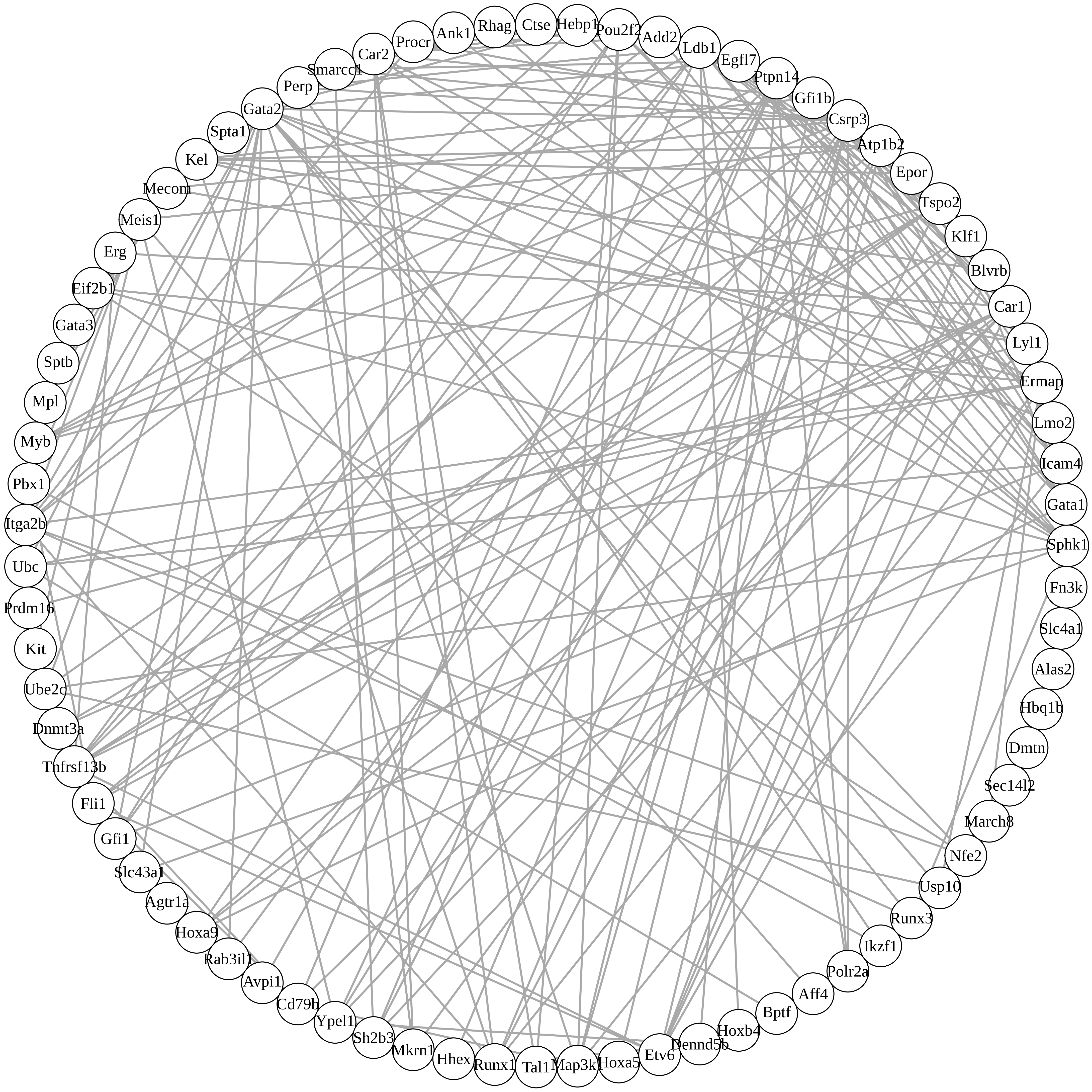

mouse_HSCtoMEP_time 17

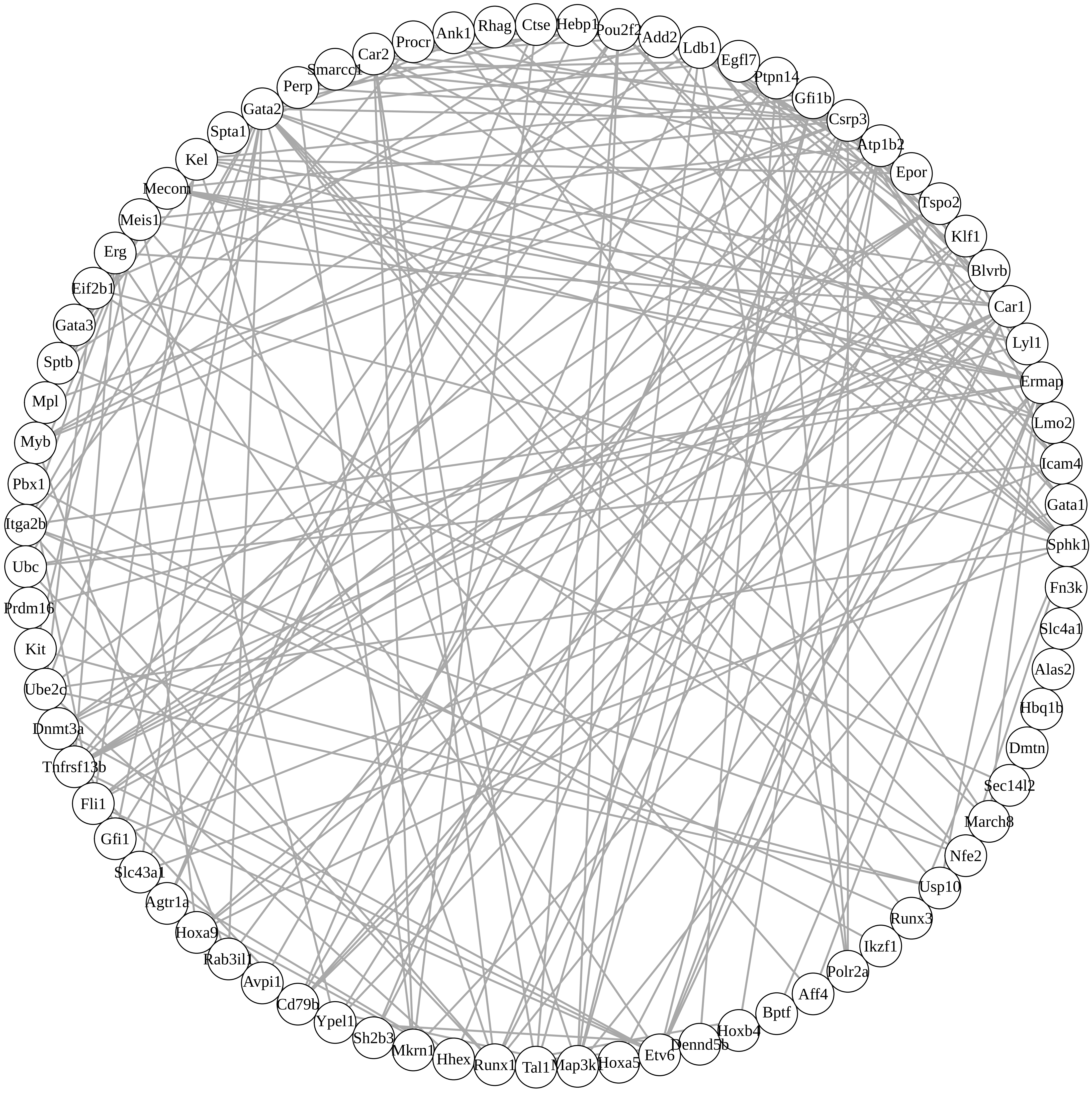

mouse_HSCtoMEP_time 18

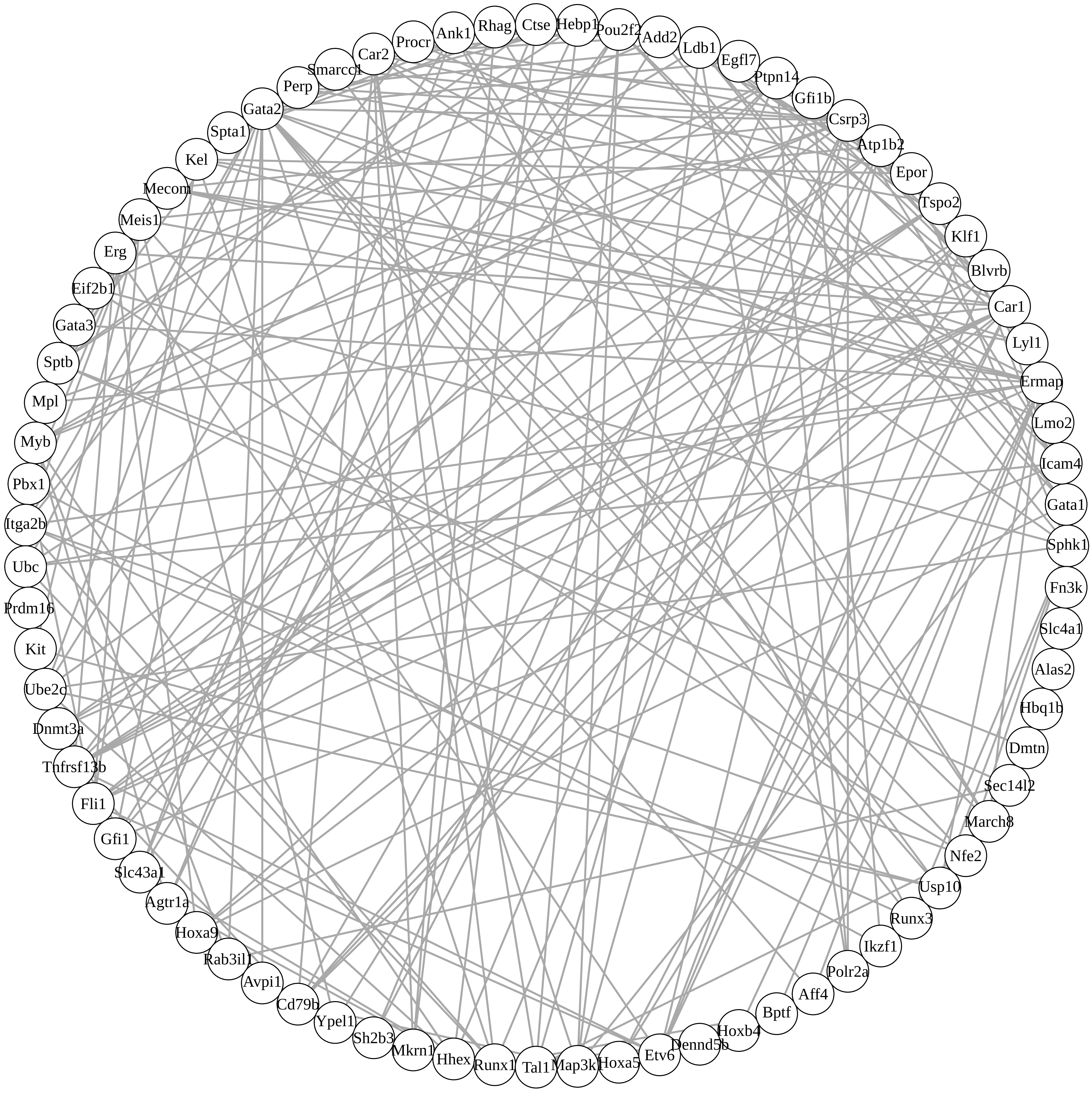

mouse_HSCtoMEP_time 19

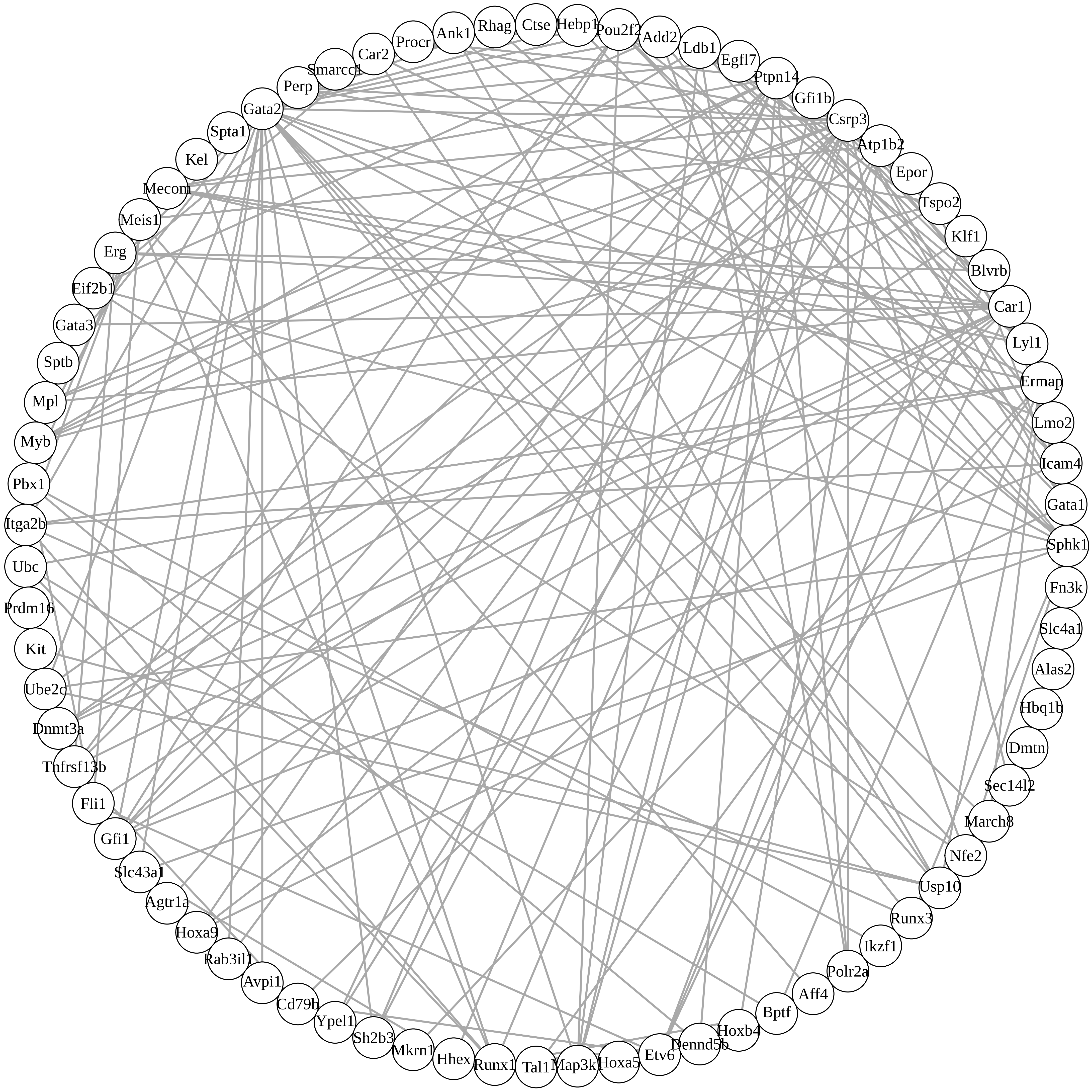

mouse_HSCtoMEP_time 20

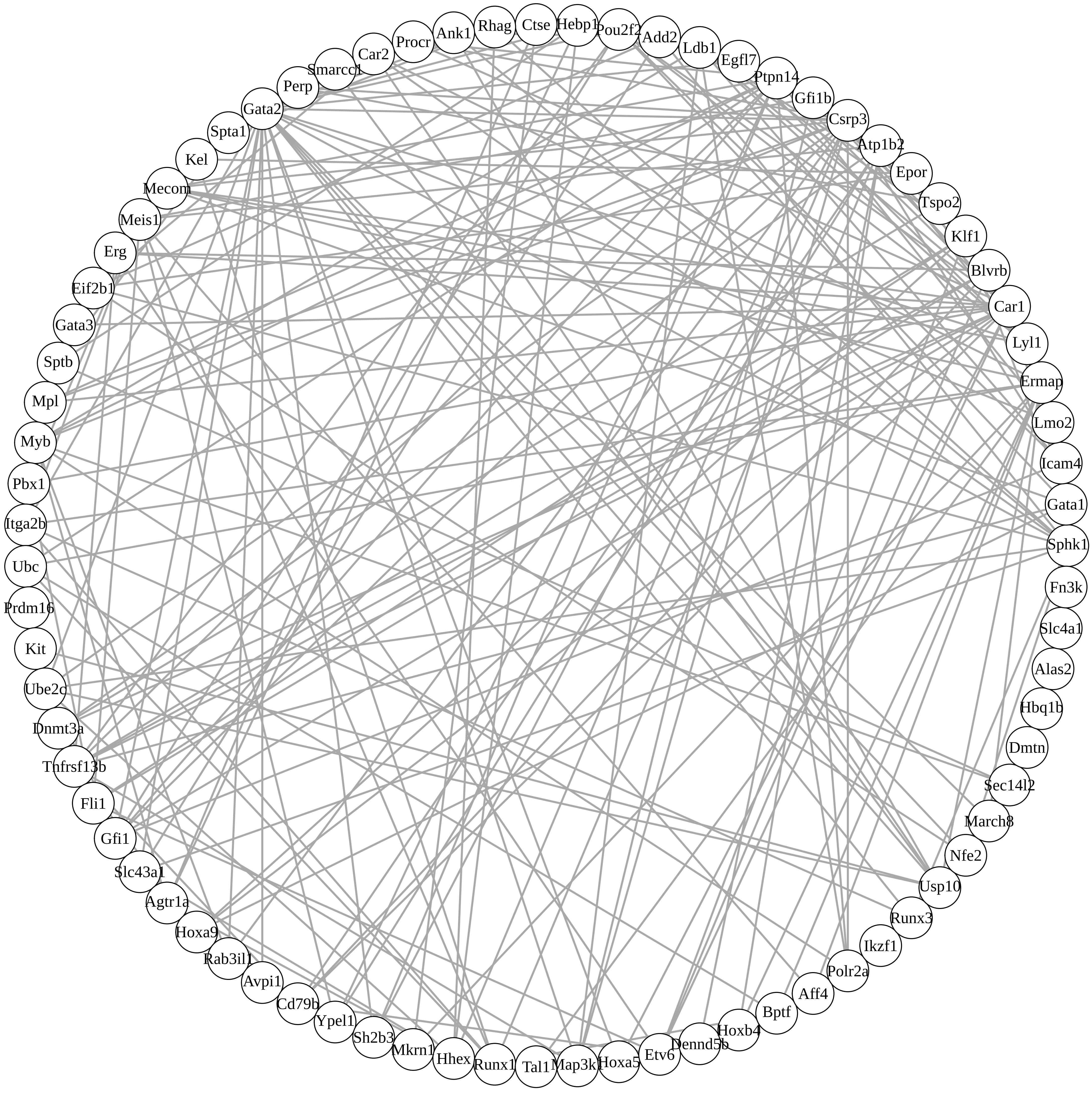

mouse_HSCtoMEP_time 21

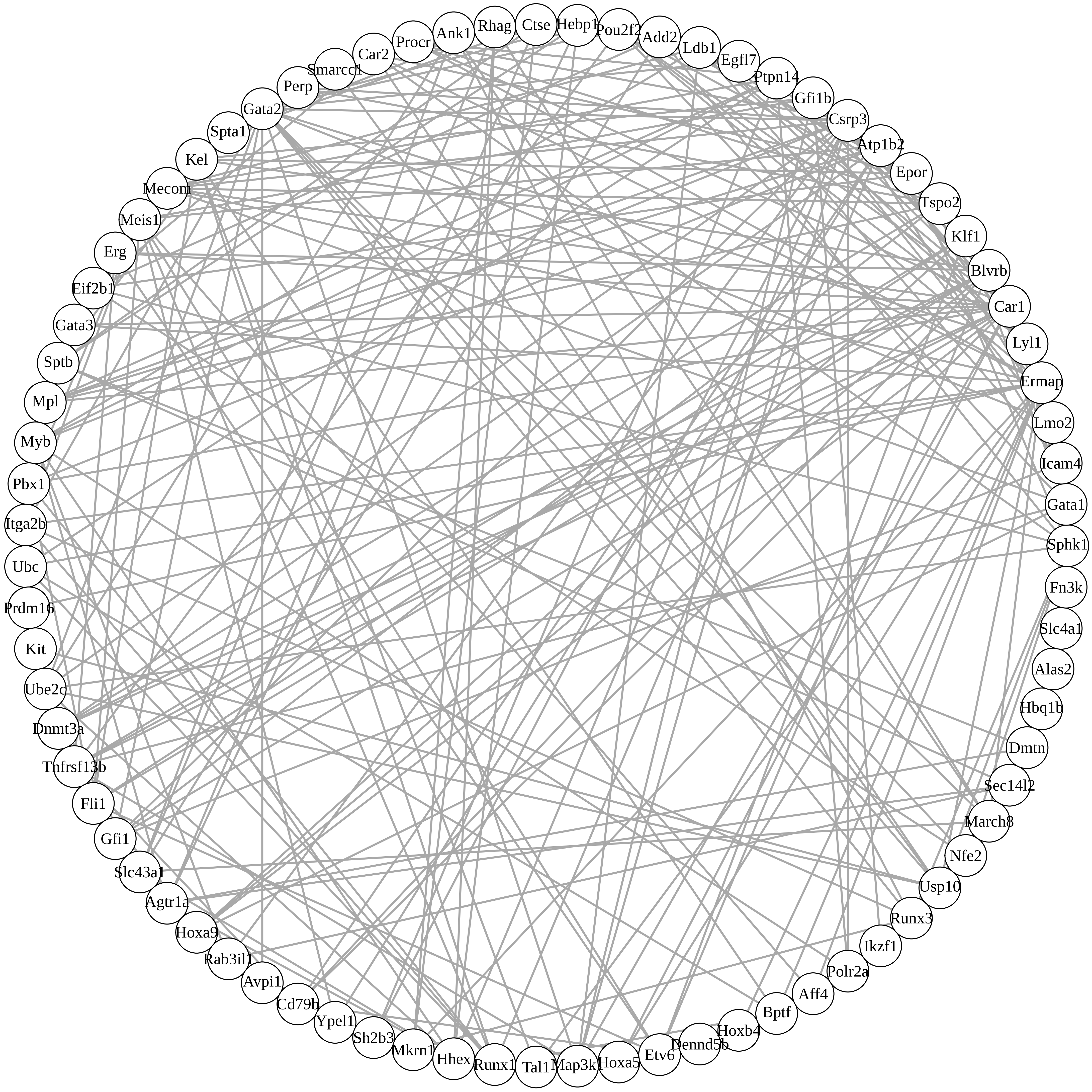

mouse_HSCtoMEP_time 22

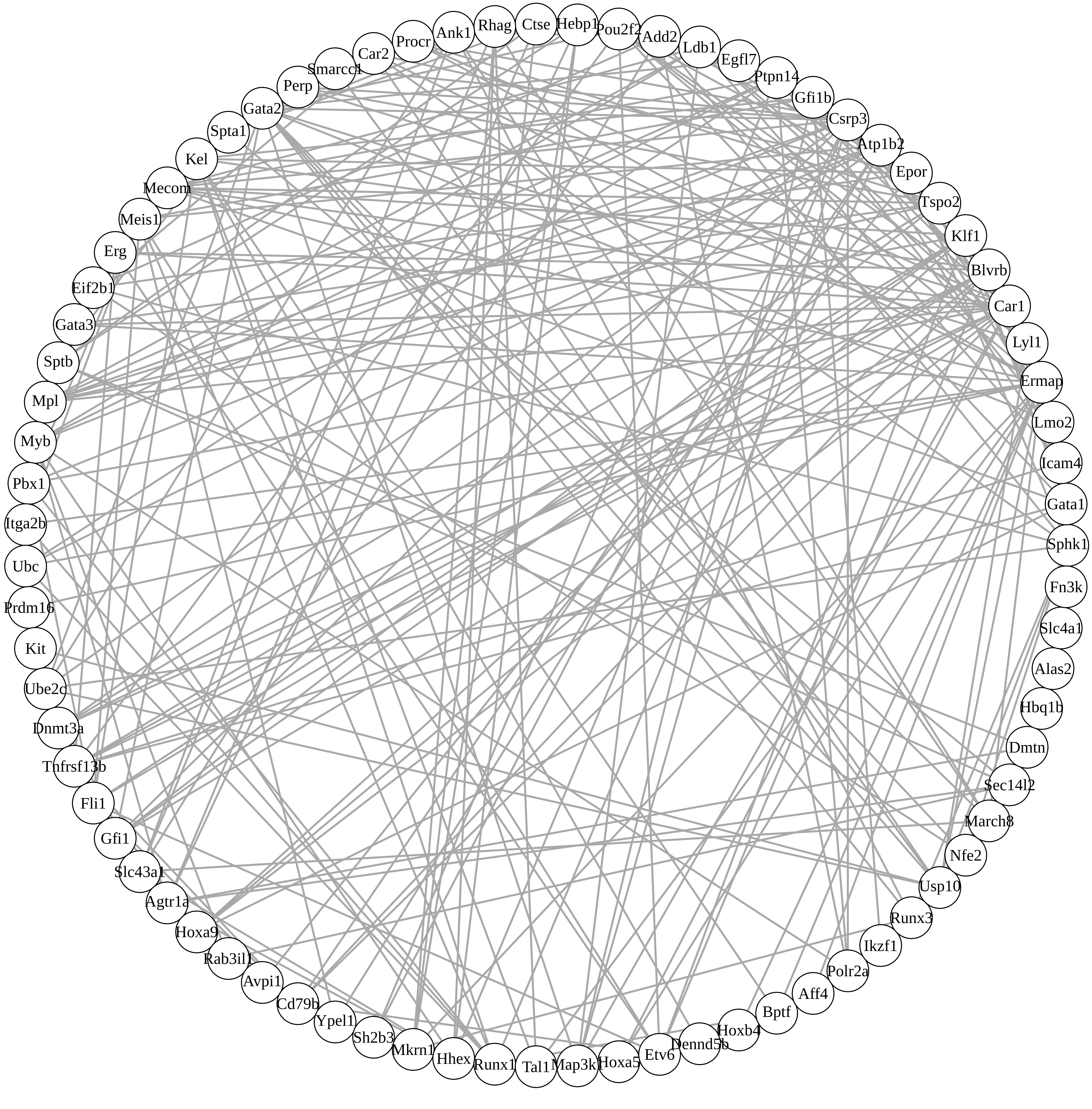

mouse_HSCtoMEP_time 23

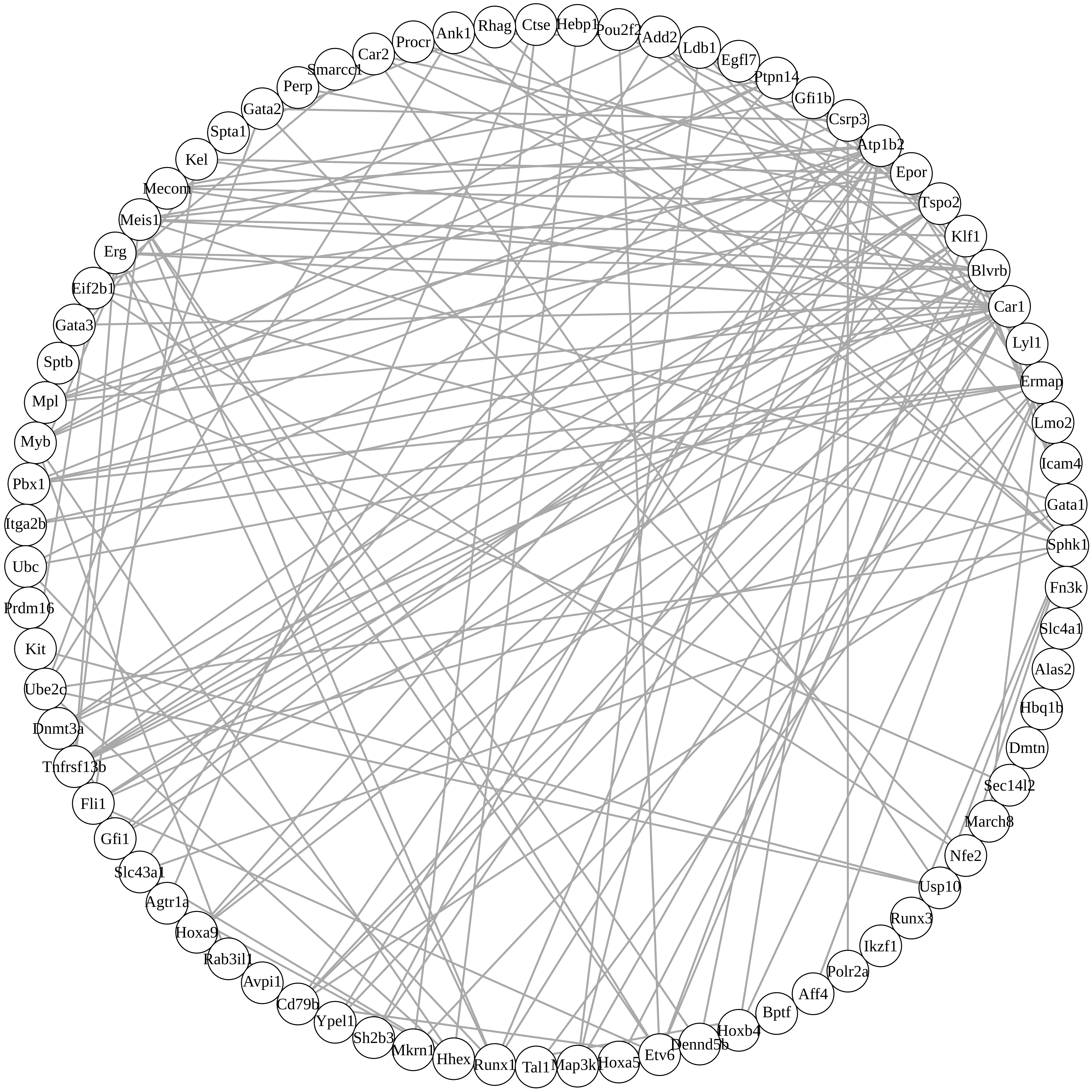

mouse_HSCtoMEP_time 24

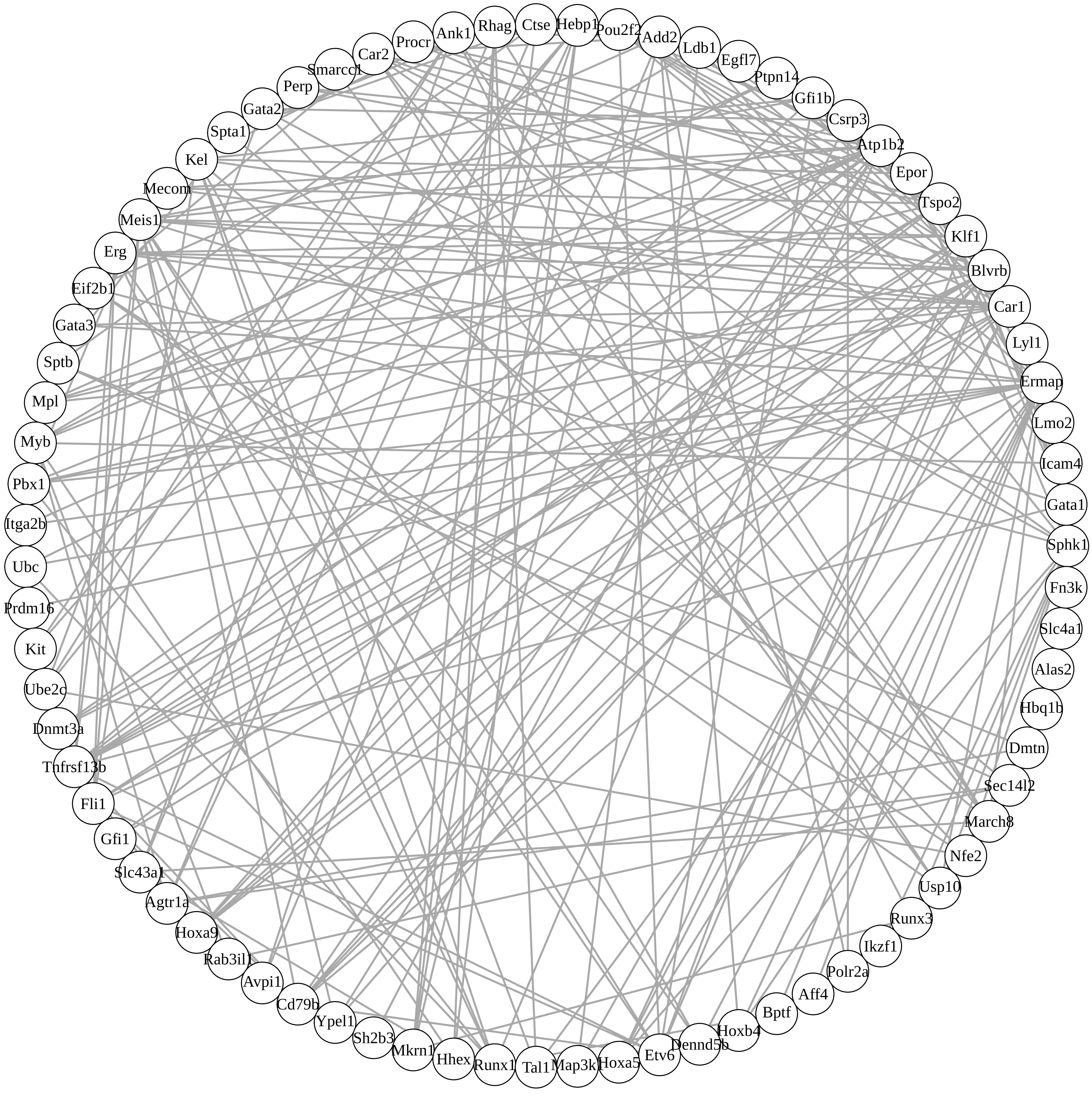

mouse_HSCtoMEP_time 25

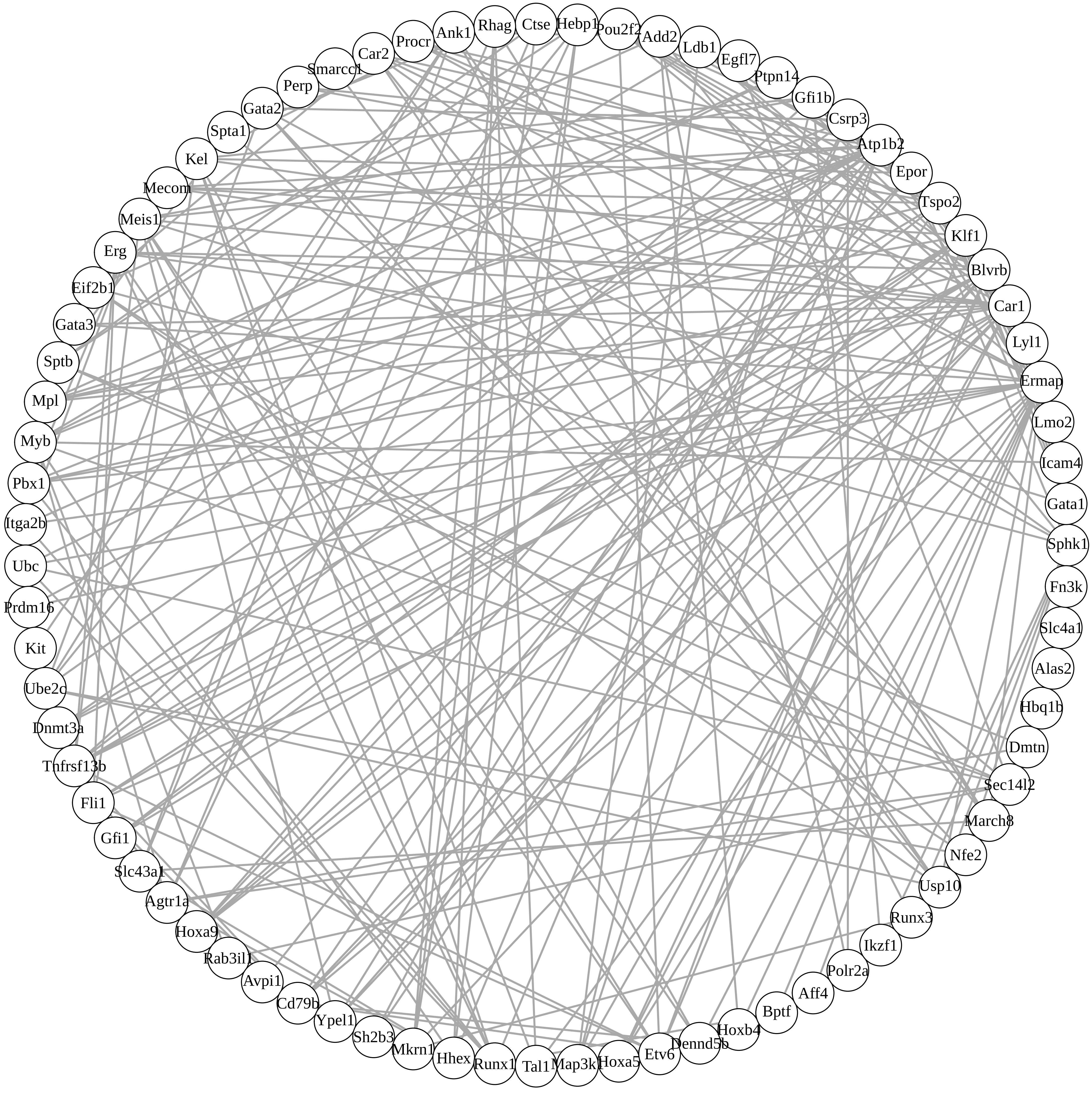

mouse_HSCtoGMP_time 1

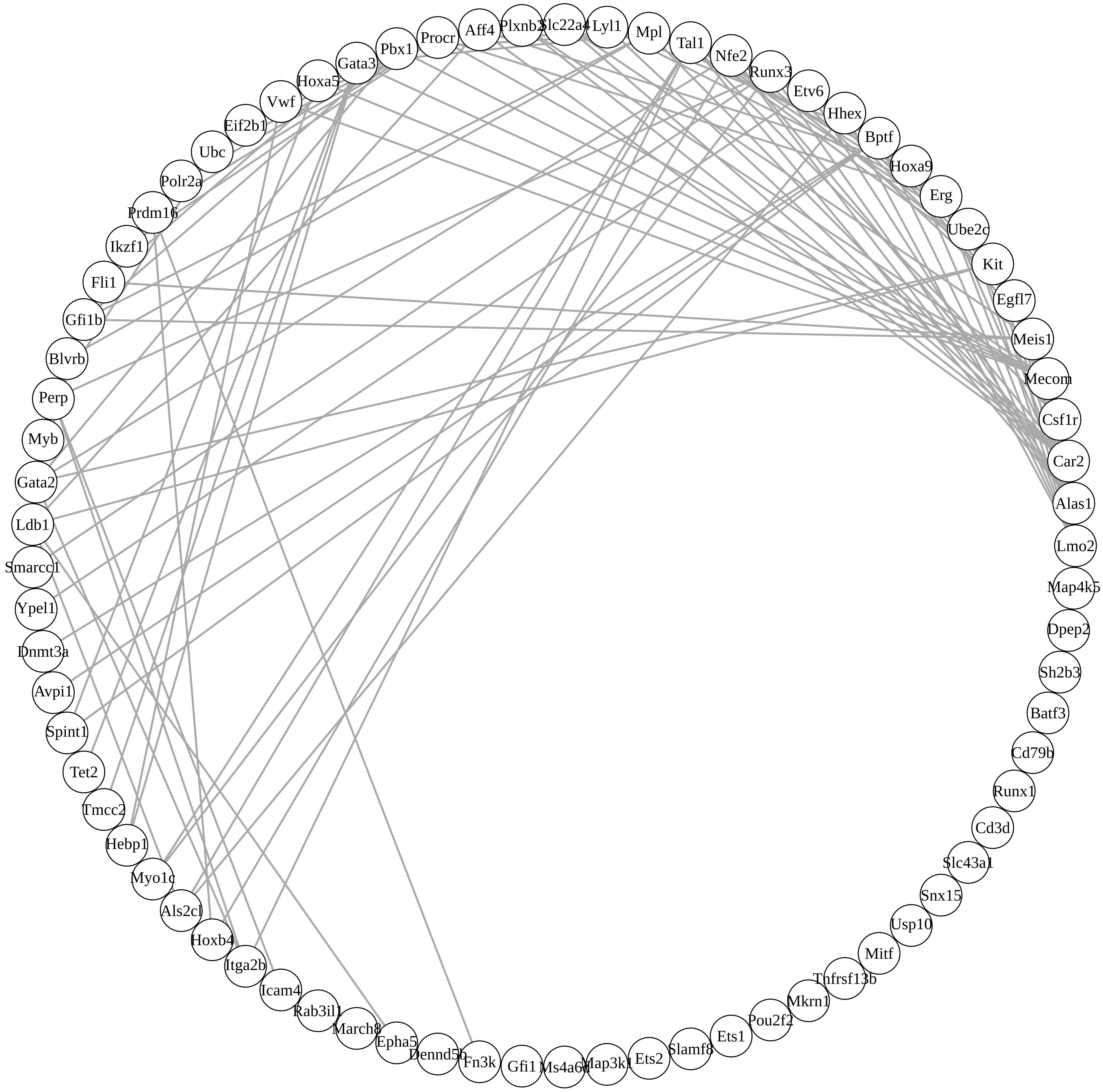

mouse_HSCtoGMP_time 2

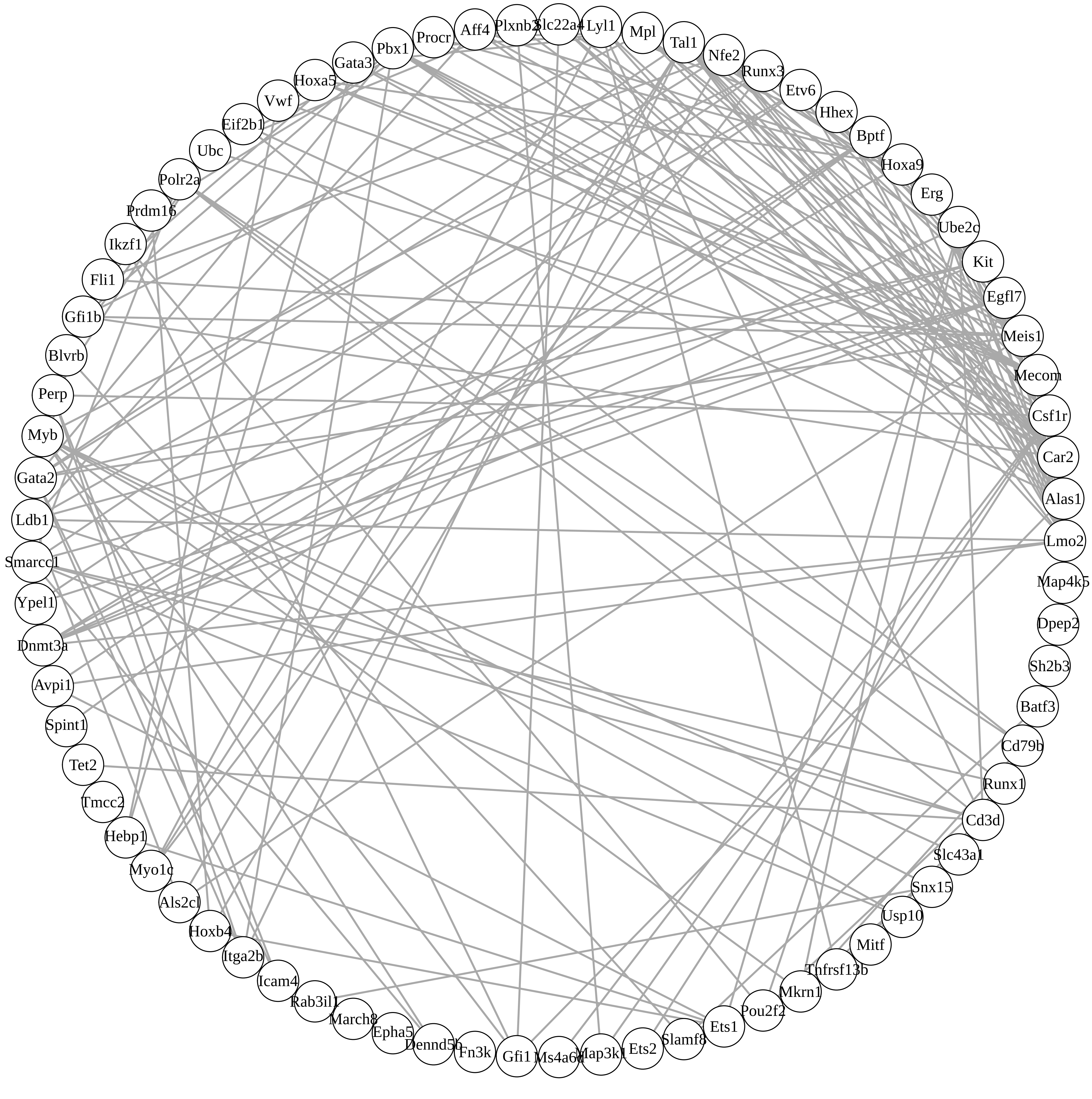

mouse_HSCtoGMP_time 3

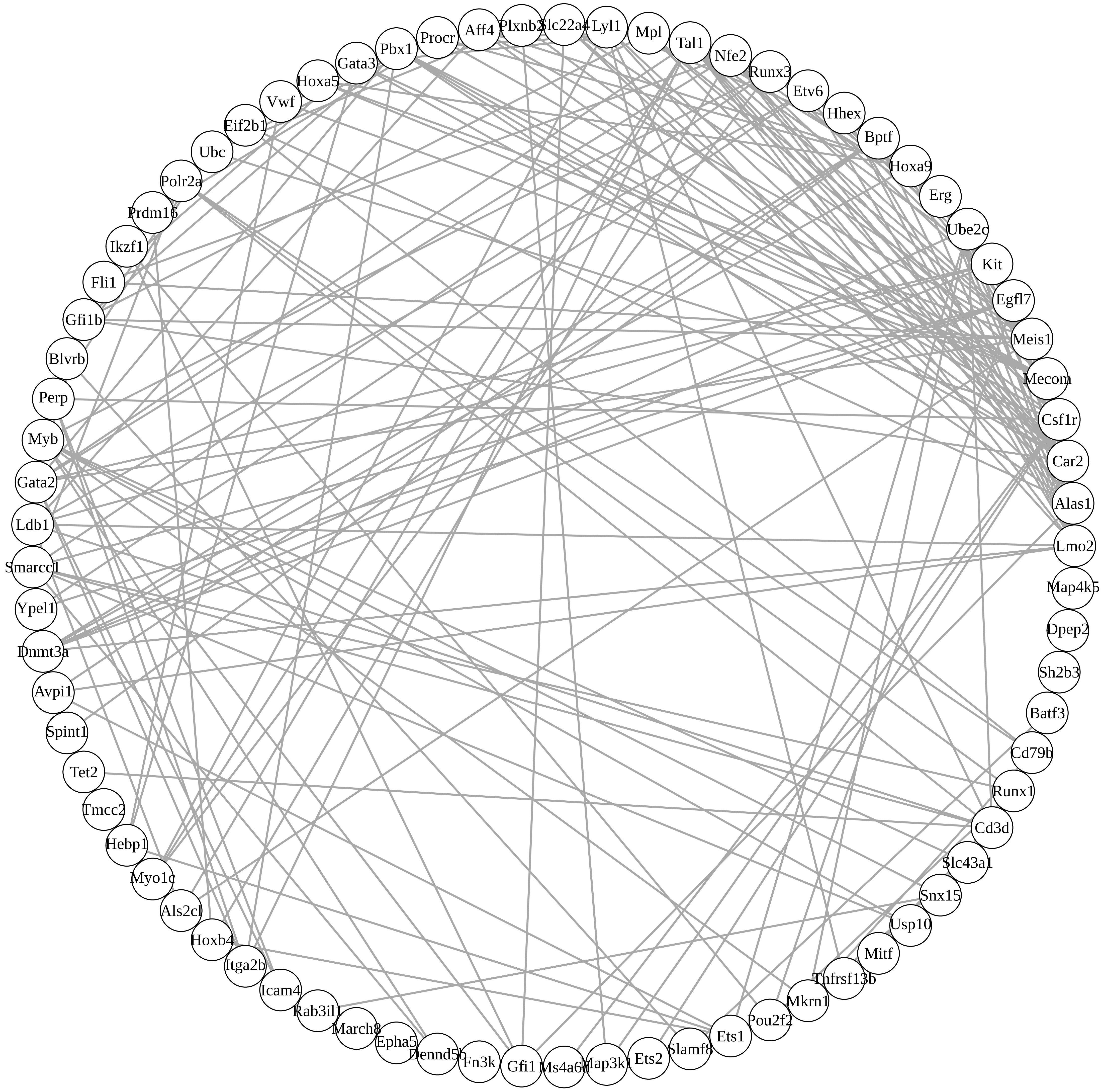

mouse_HSCtoGMP_time 4

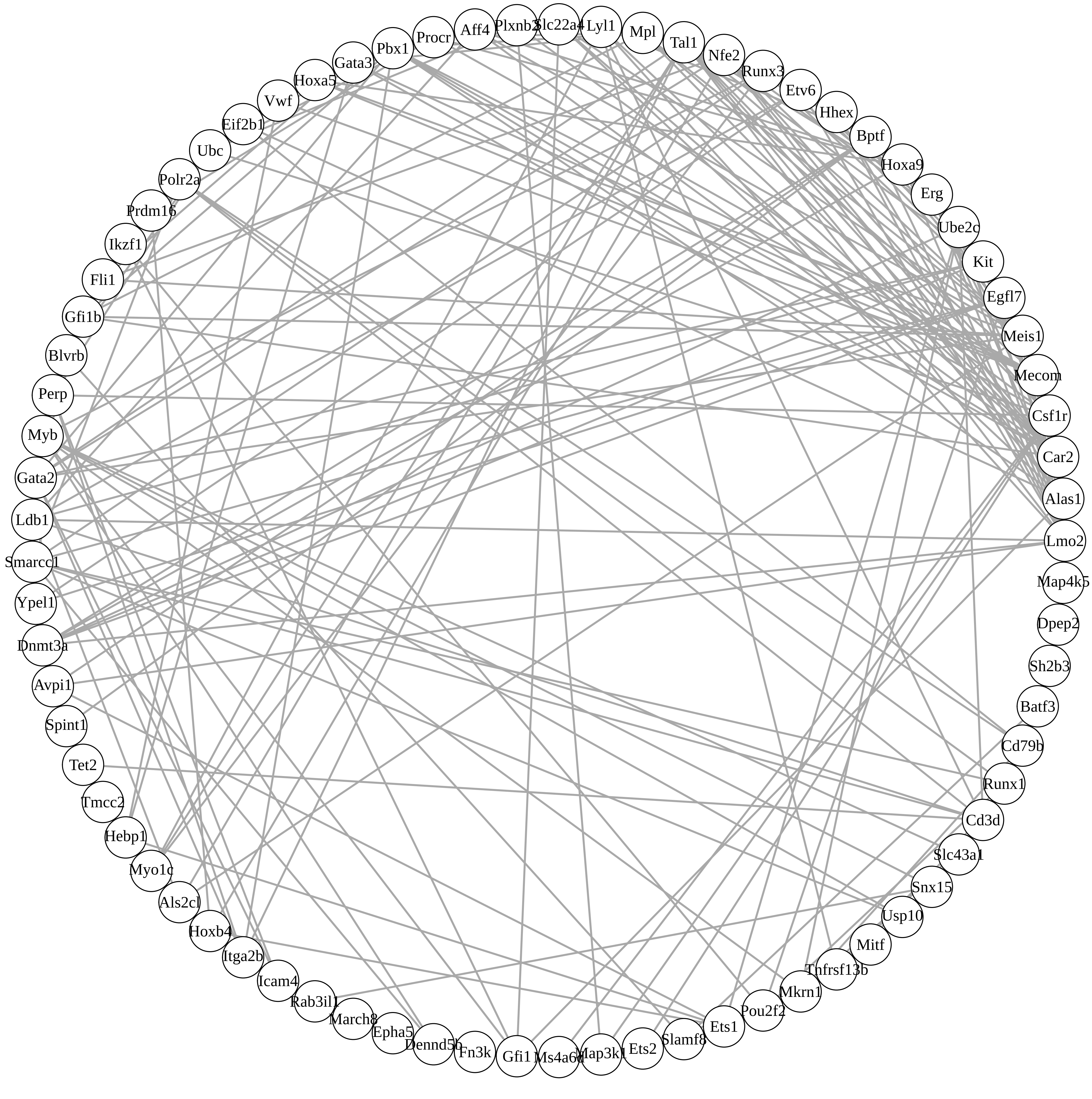

mouse_HSCtoGMP_time 5

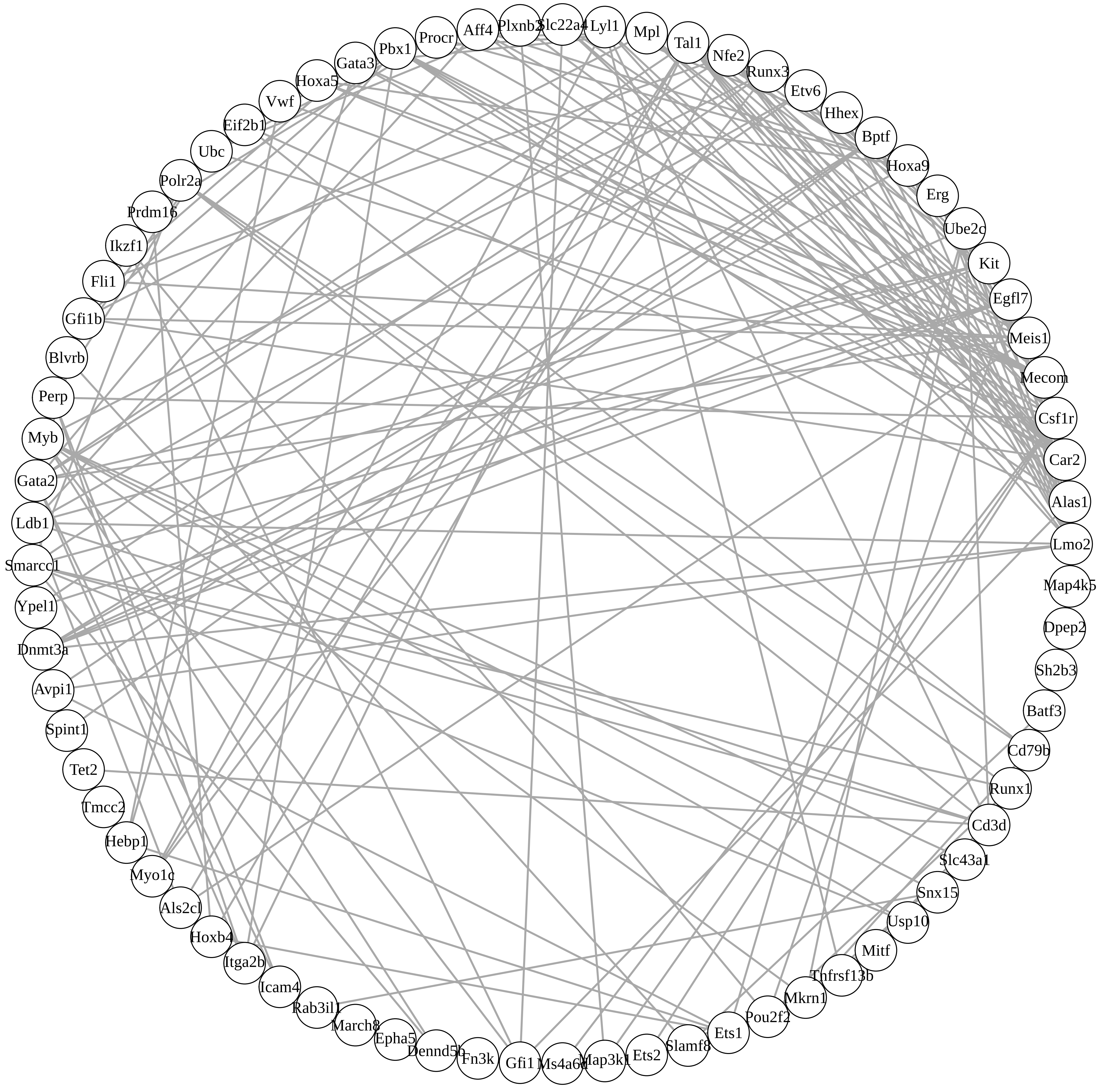

mouse_HSCtoGMP_time 6

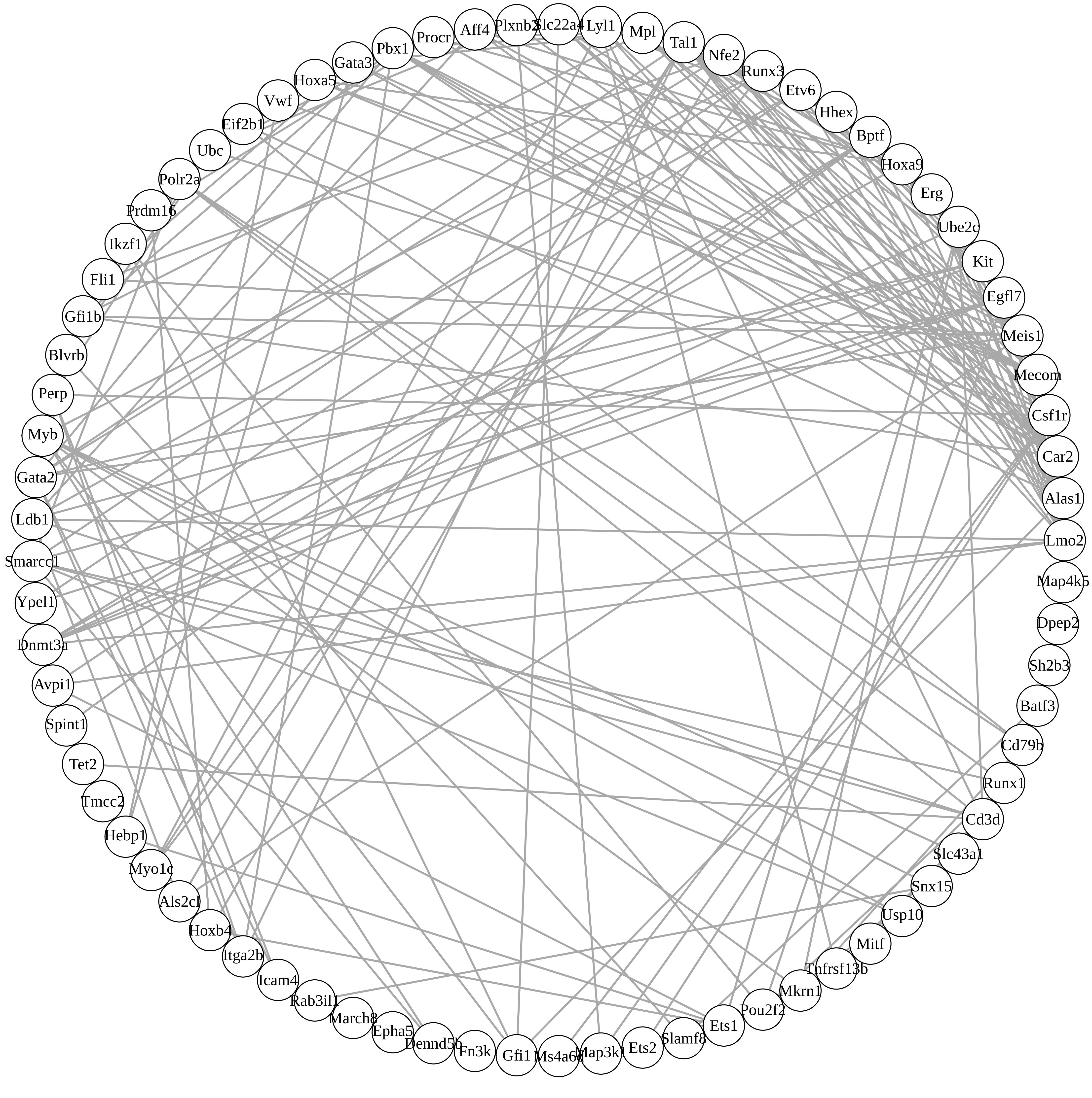

mouse_HSCtoGMP_time 7

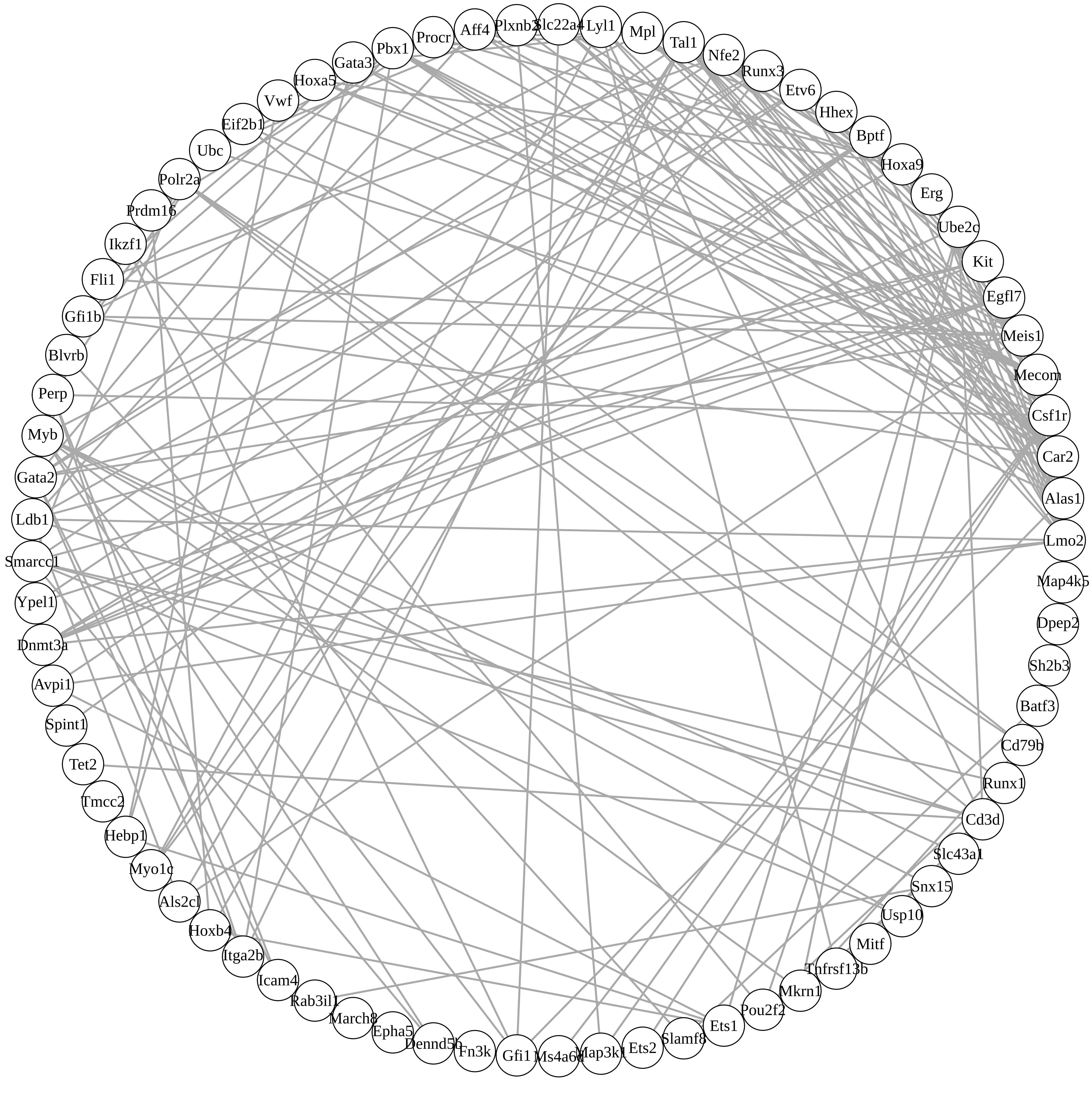

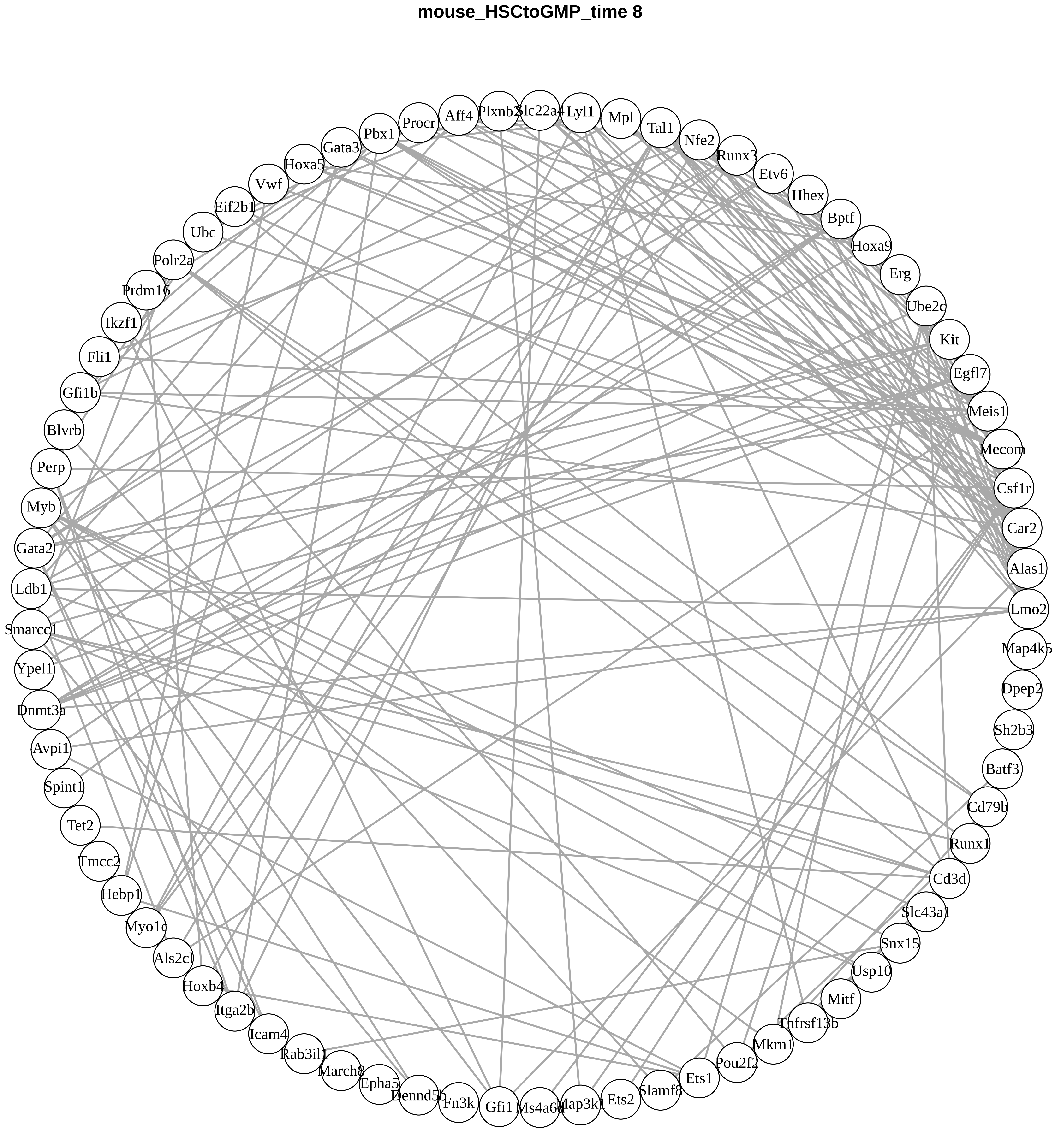
mouse_HSCtoGMP_time 8
Plxnb2
Slc22a4
Lyl1
Mpl
Tal1
Nfe2
Runx3
Etv6
Hhex
Bptf
Hoxa9
Erg
Ube2c
Kit
Egfl7
Meis1
Mecom
Csf1r
Car2
Alas1
Lmo2
Map4k5
Dpep2
Sh2b3
Batf3
Cd79b
Runx1
Cd3d
Slc43a1
Snx15
Usp10
Mitf
Tnfrsf13b
Mkrn1
Pou2f2
Ets1
Slamf8
Ets2
Map3k1
Ms4a6d
Gfi1
Fn3k
Dennd5b
Epha5
March8
Rab3il1
Icam4
Itga2b
Hoxb4
Als2cl
Myo1c
Hebp1
Tmcc2
Tet2
Spint1
Avpi1
Dnmt3a
Ypel1
Smarcc1
Ldb1
Gata2
Myb
Perp
Blvrb
Gfi1b
Fli1
Ikzf1
Prdm16
Polr2a
Ubc
Eif2b1
Vwf
Hoxa5
Gata3
Pbx1
Procr
Aff4

mouse_HSCtoGMP_time 9

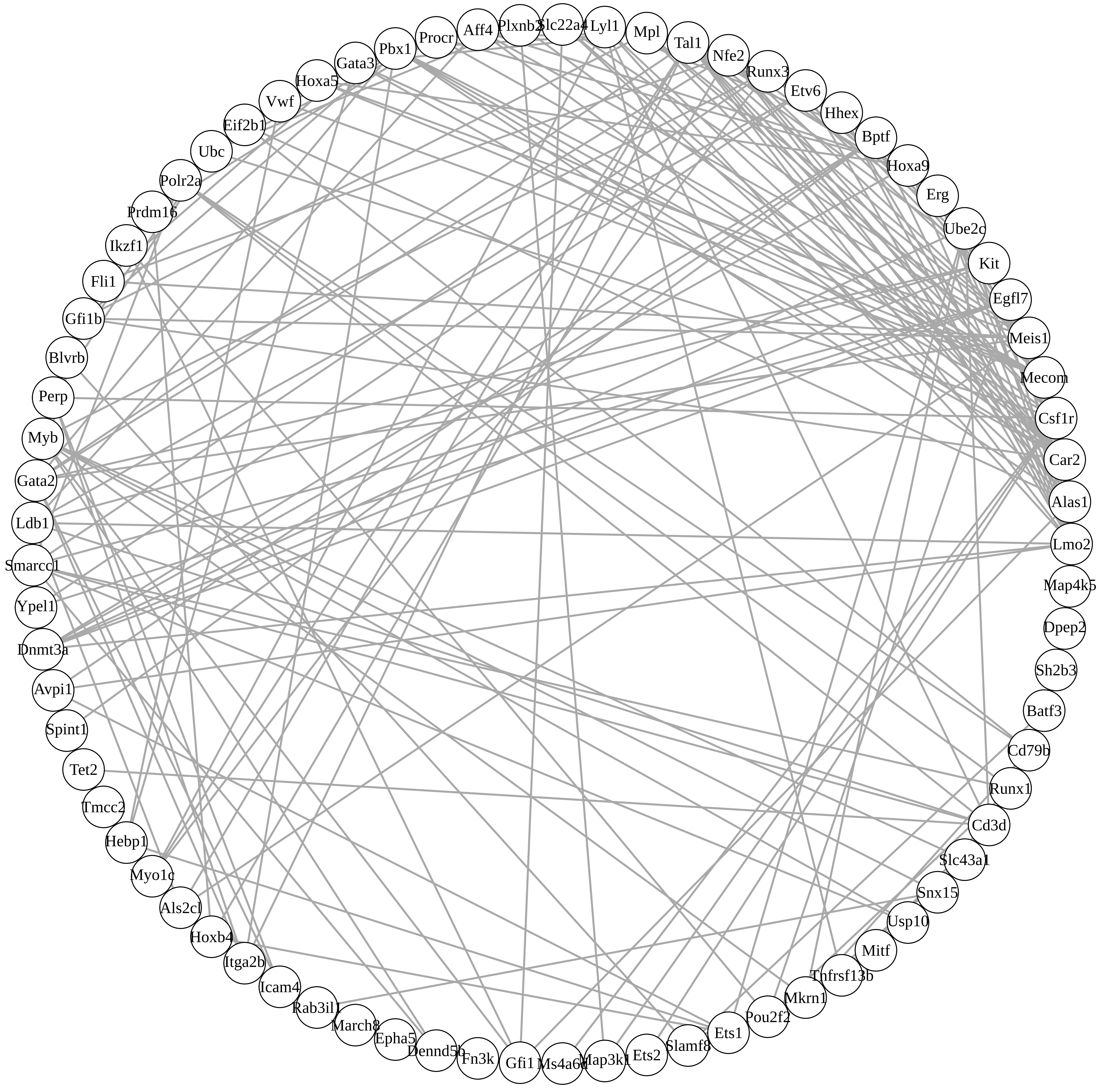

mouse_HSCtoGMP_time 10

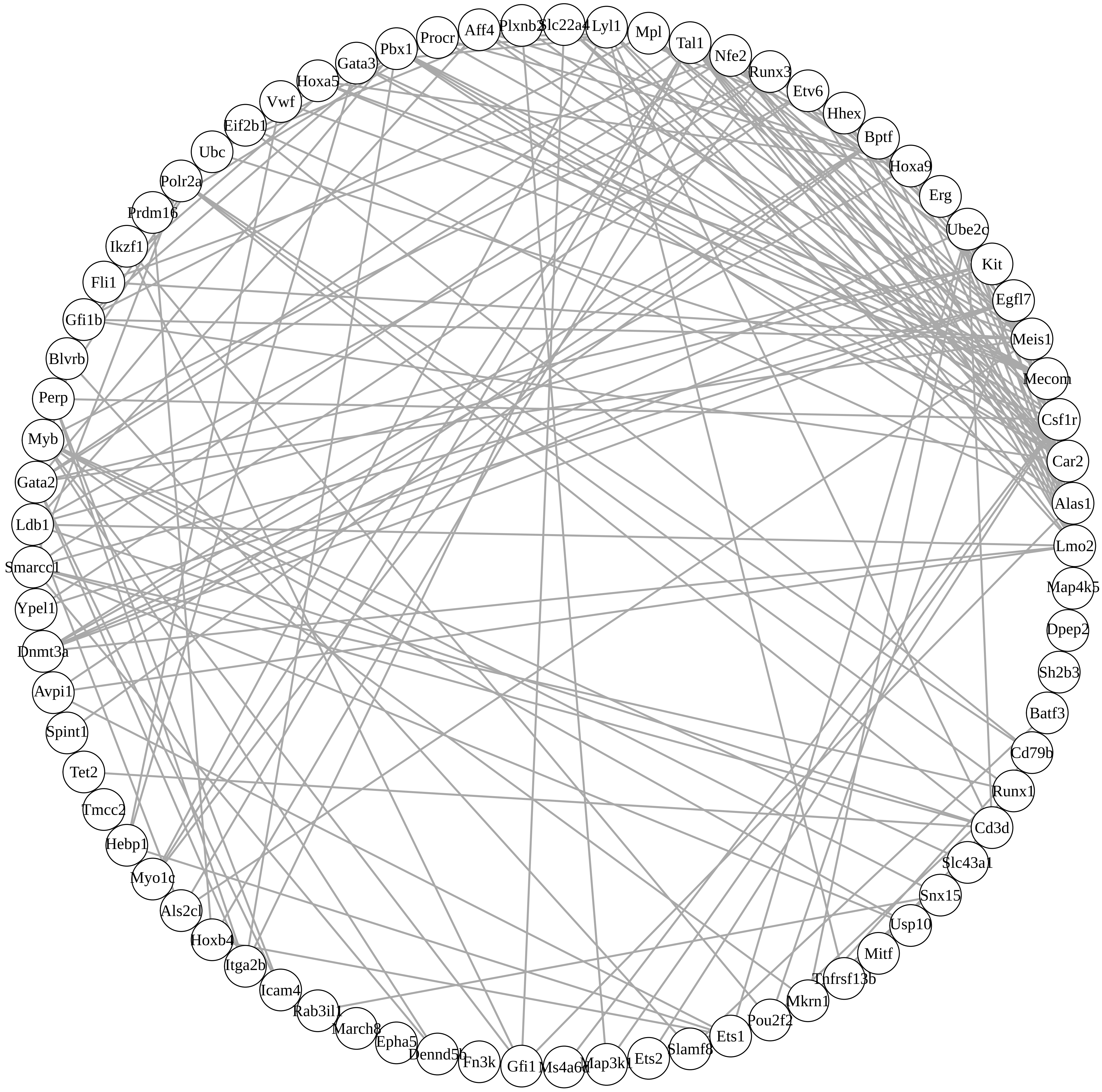

mouse_HSCtoGMP_time 11

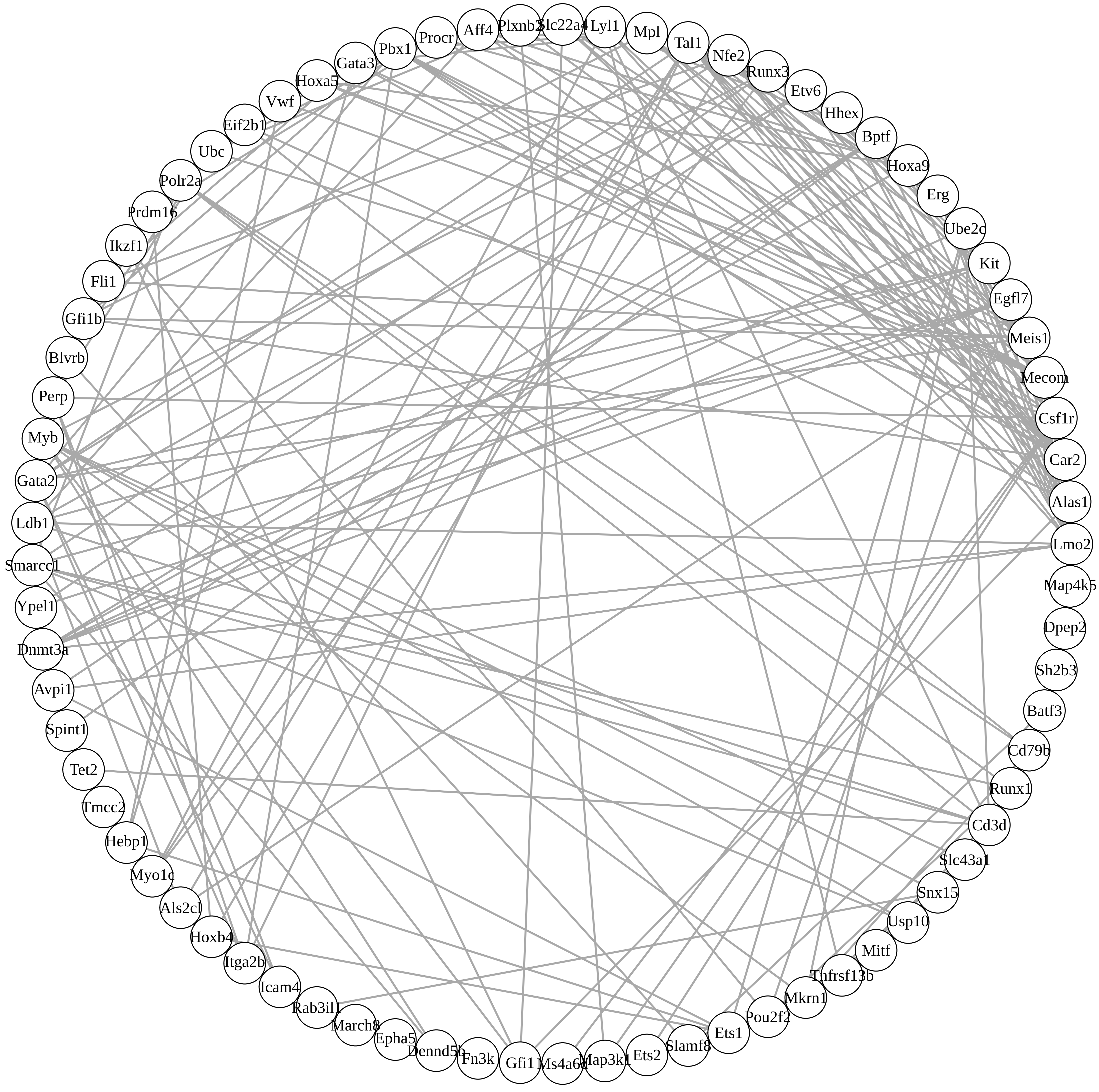

mouse_HSCtoGMP_time 12

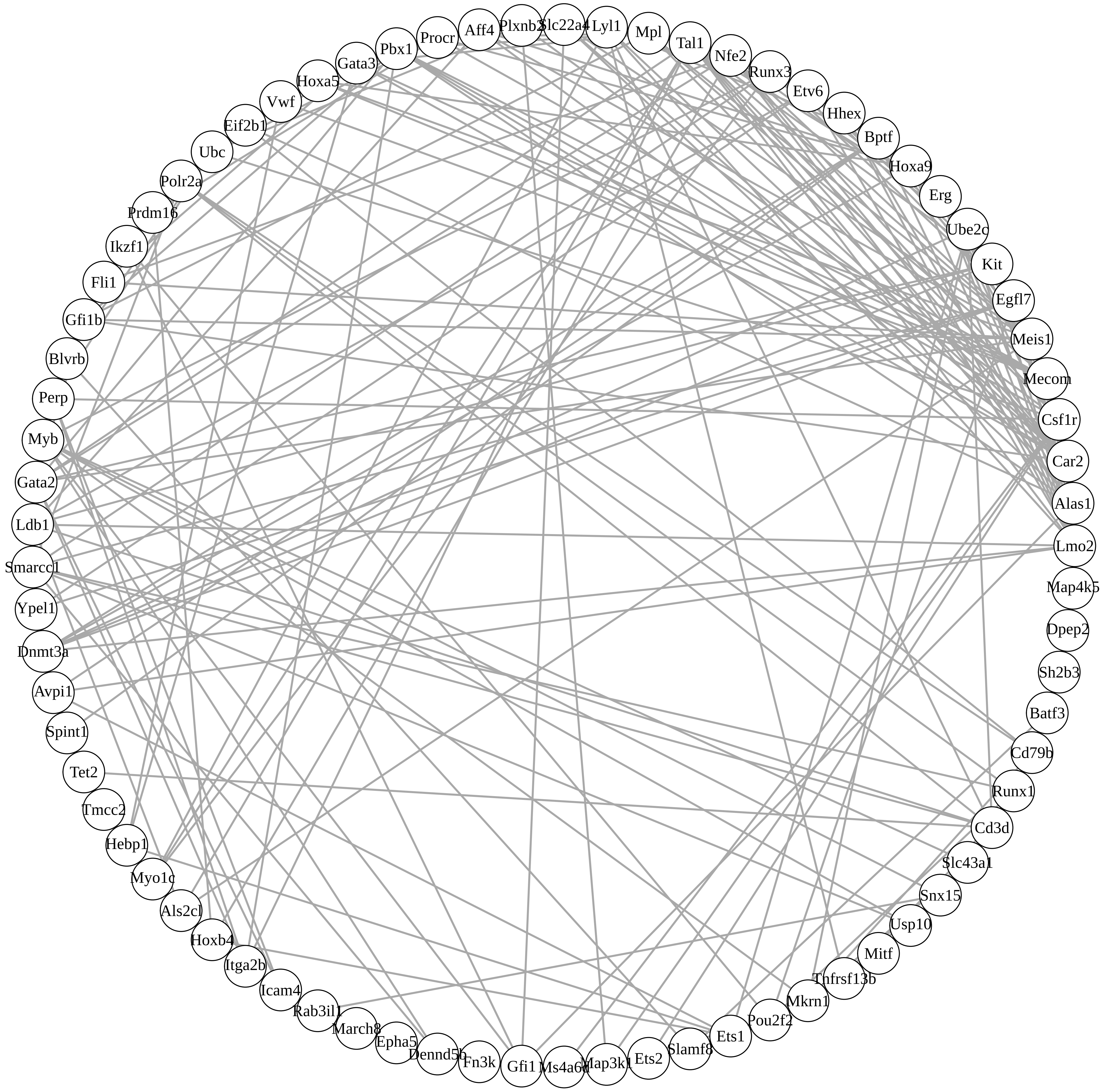

mouse_HSCtoGMP_time 13

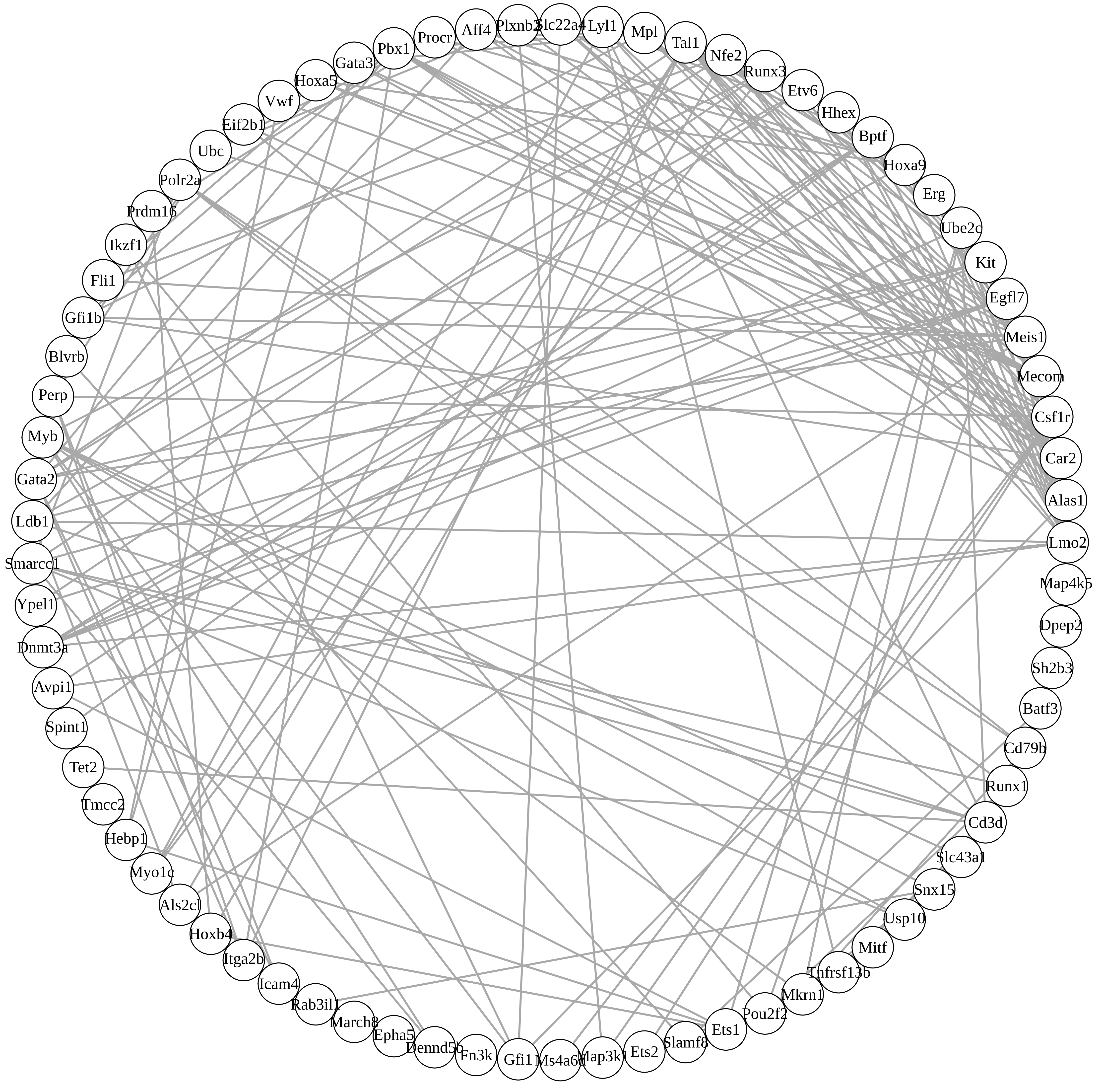

mouse_HSCtoGMP_time 14

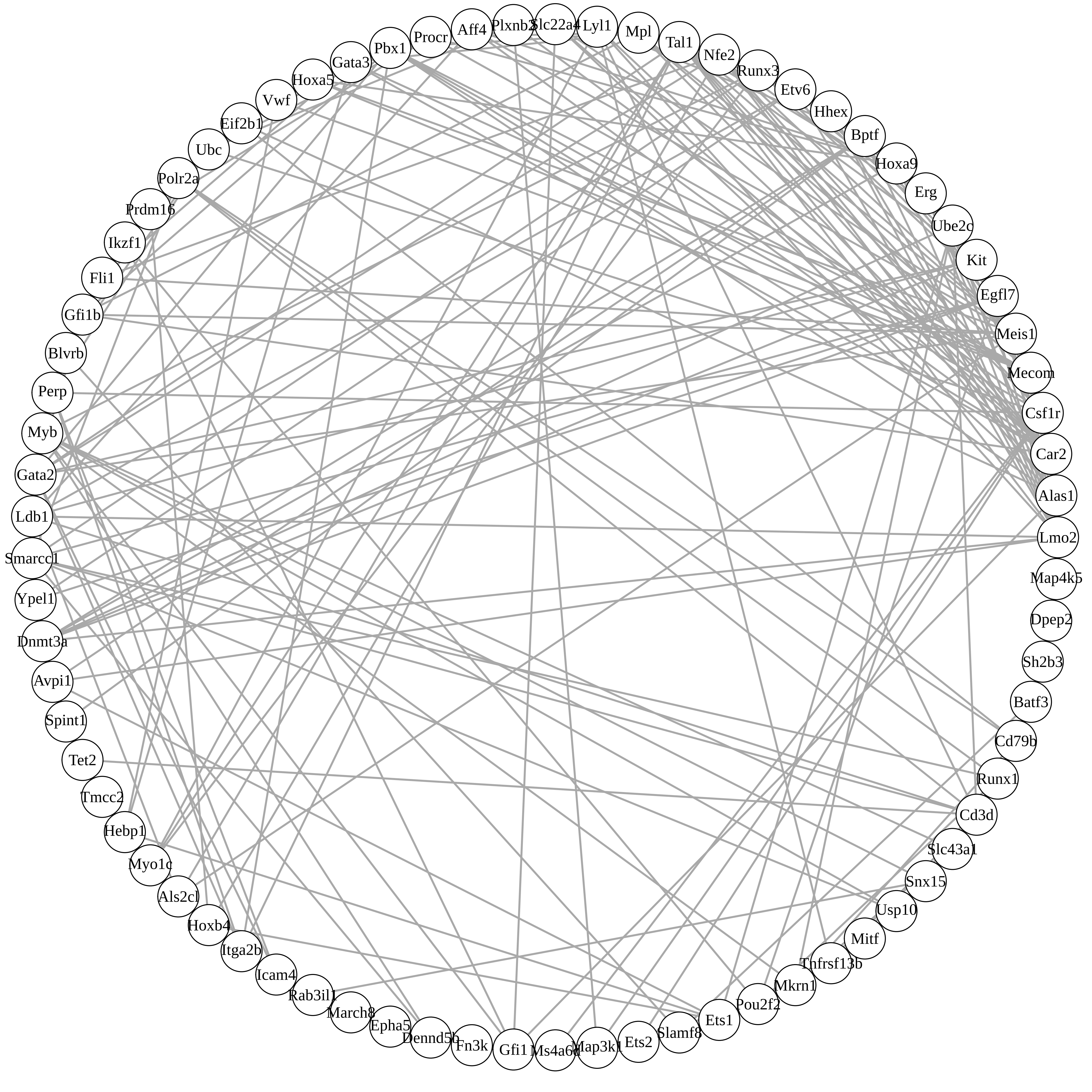

mouse_HSCtoGMP_time 15

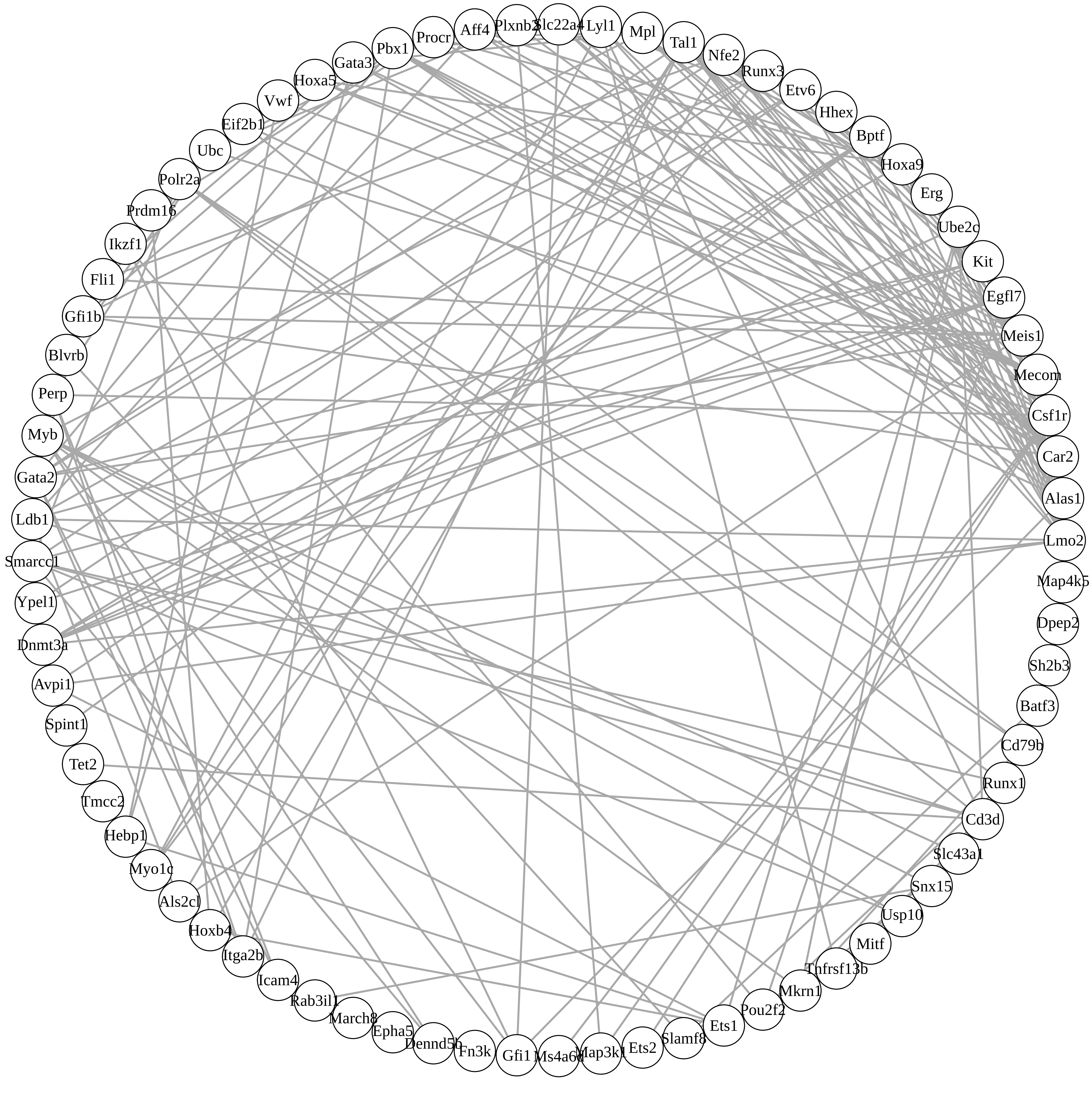

mouse_HSCtoGMP_time 16

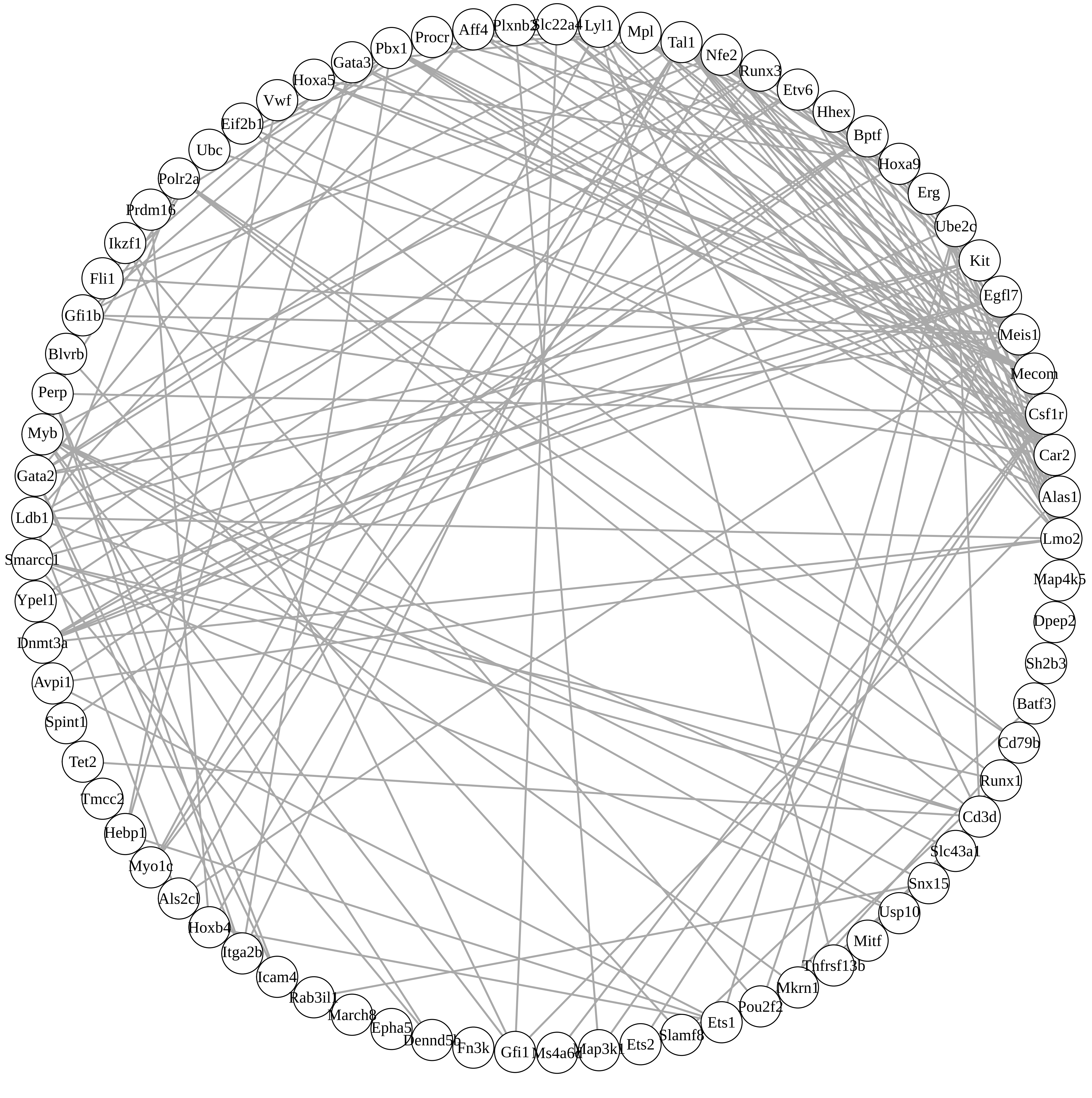

mouse_HSCtoGMP_time 17

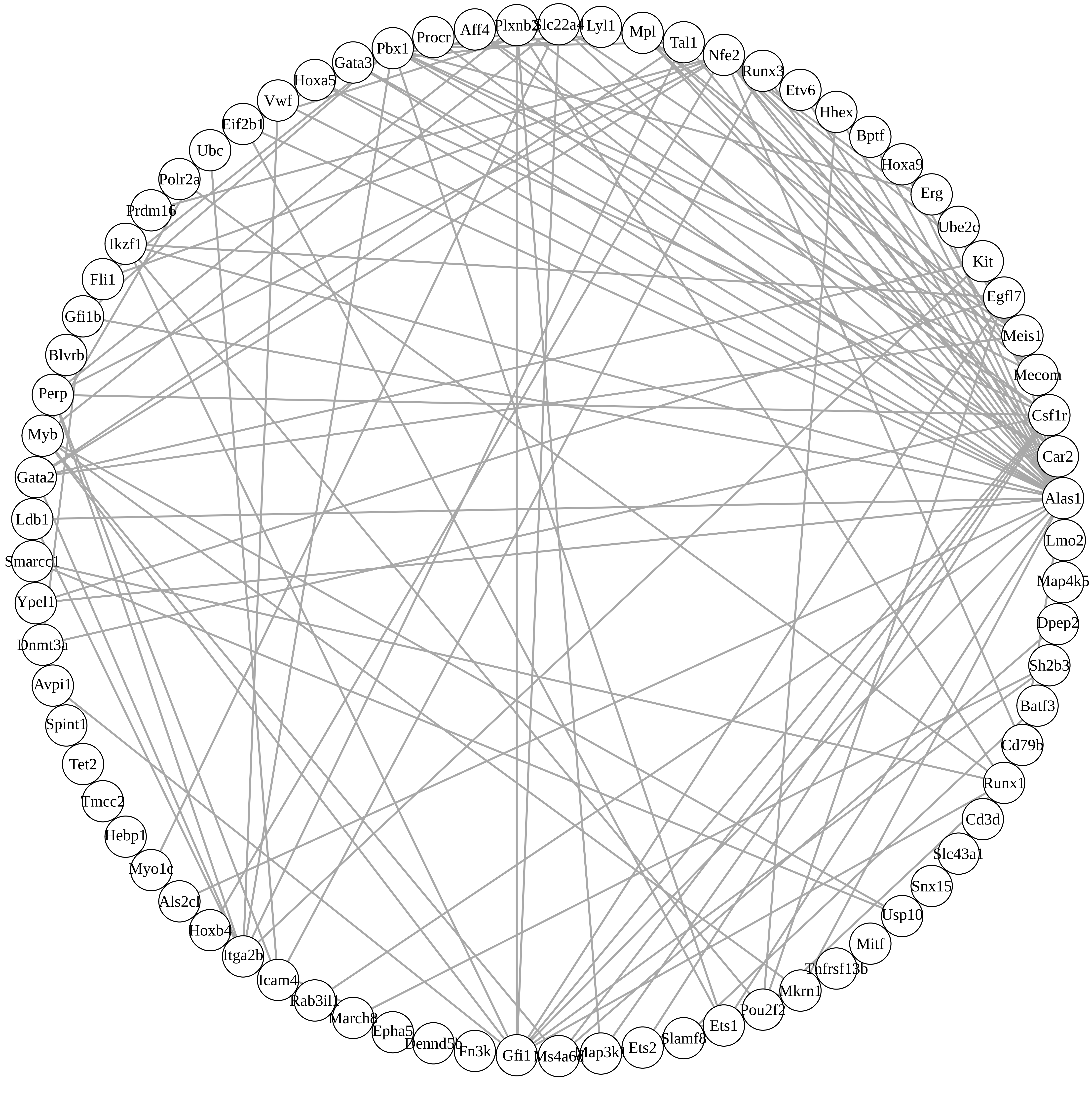

mouse_HSCtoGMP_time 18

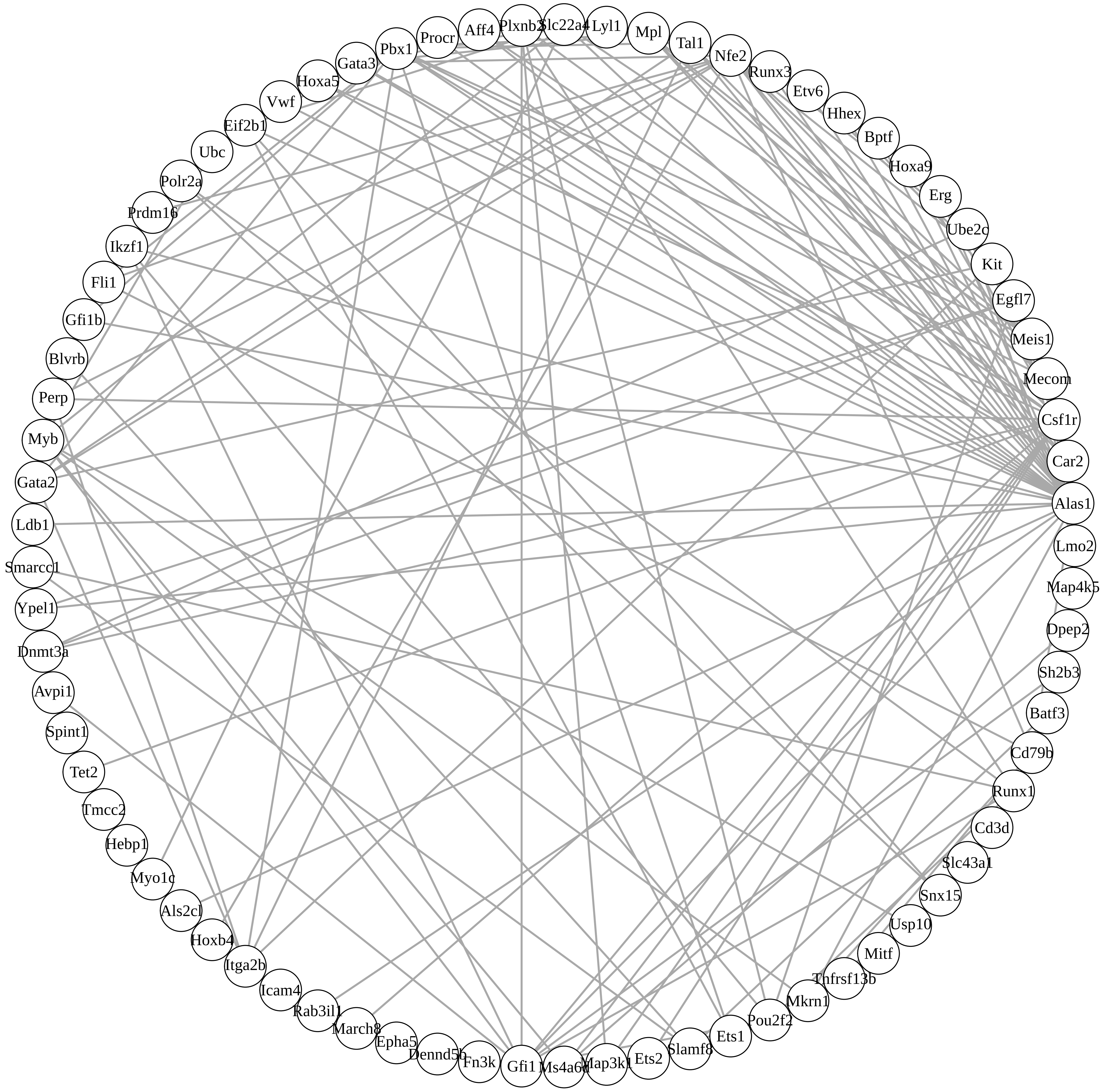

mouse_HSCtoGMP_time 19

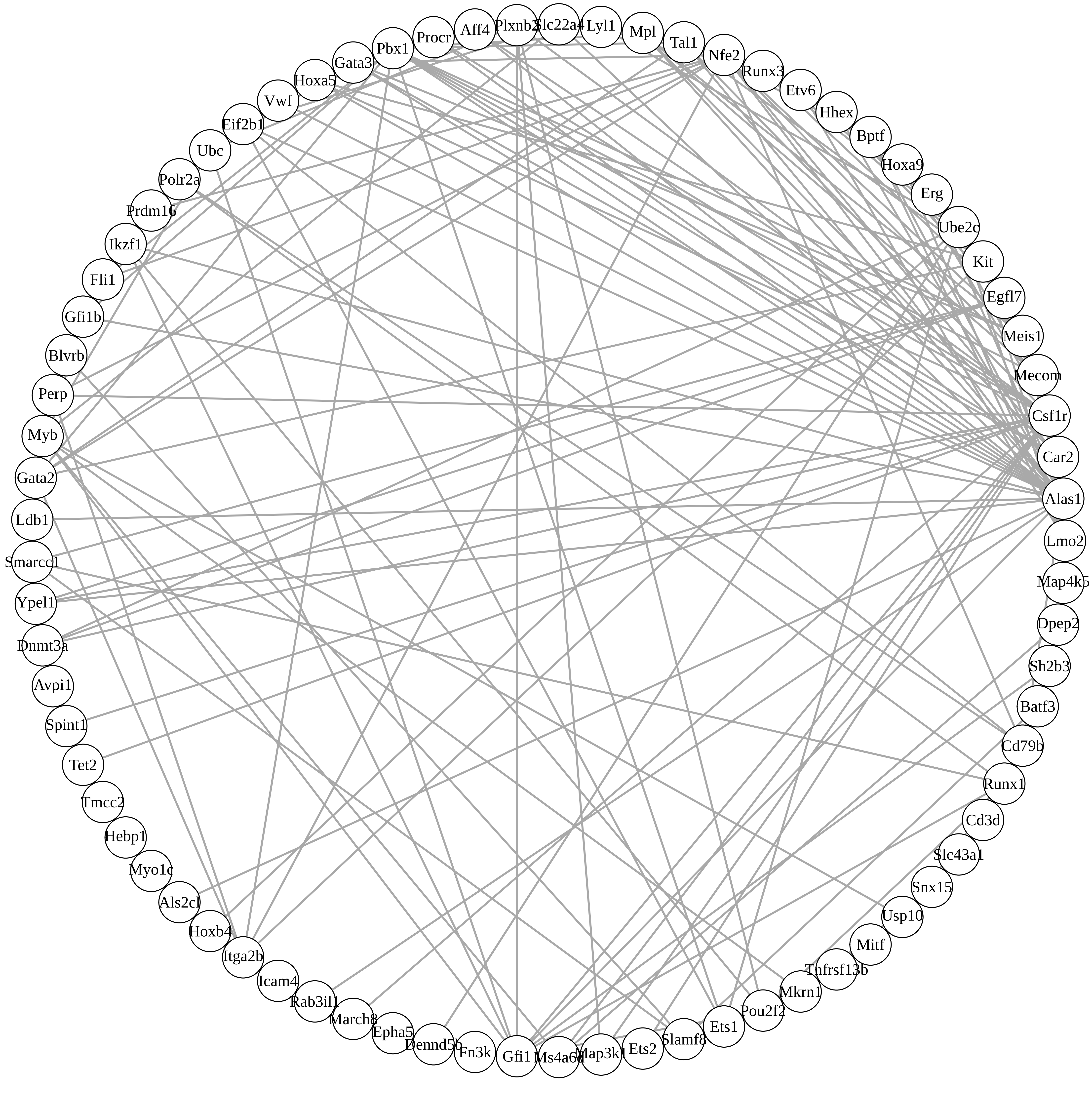

mouse_HSCtoGMP_time 20

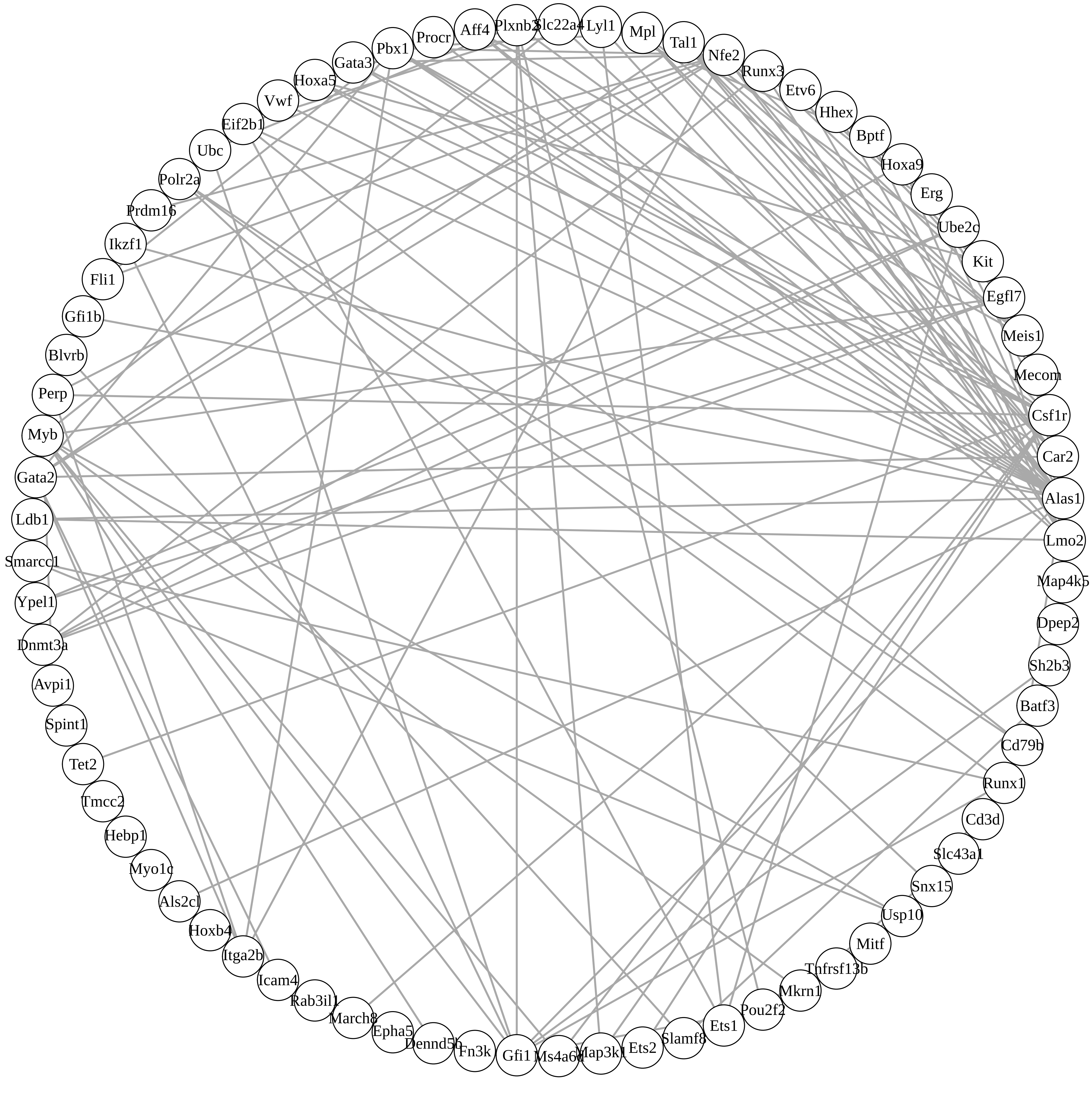

mouse_HSCtoGMP_time 21

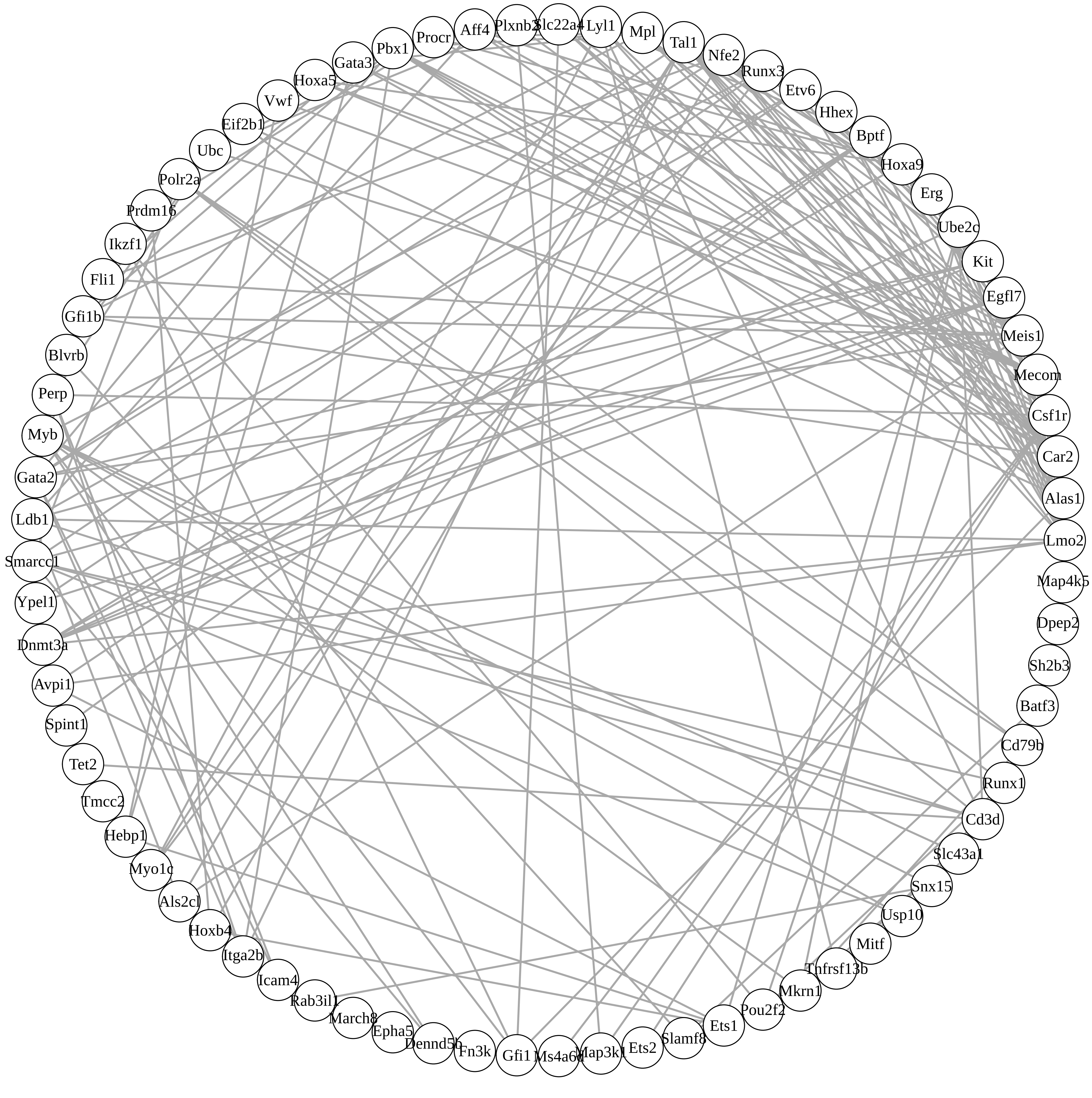

mouse_HSCtoGMP_time 22

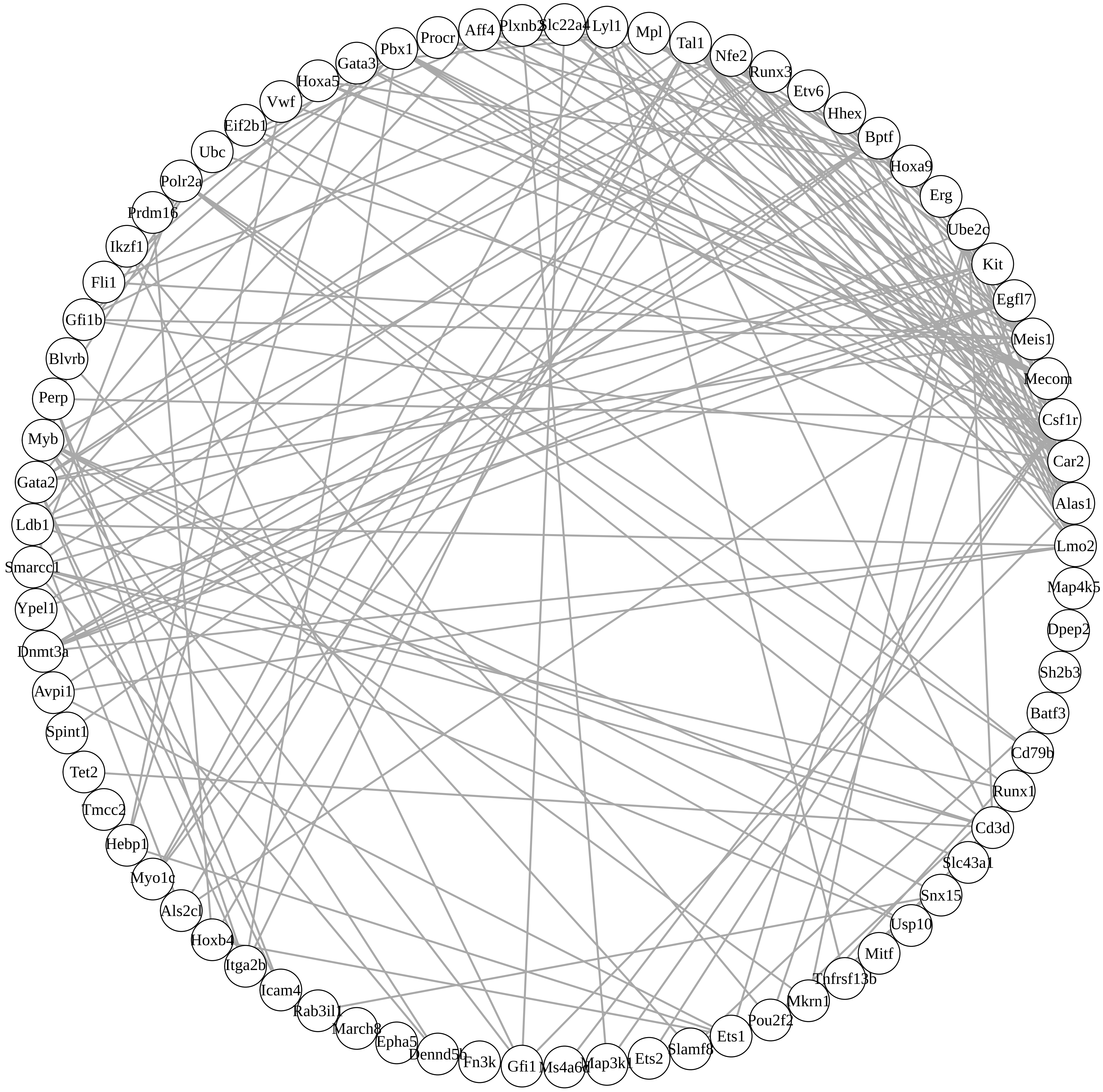

mouse_HSCtoGMP_time 23

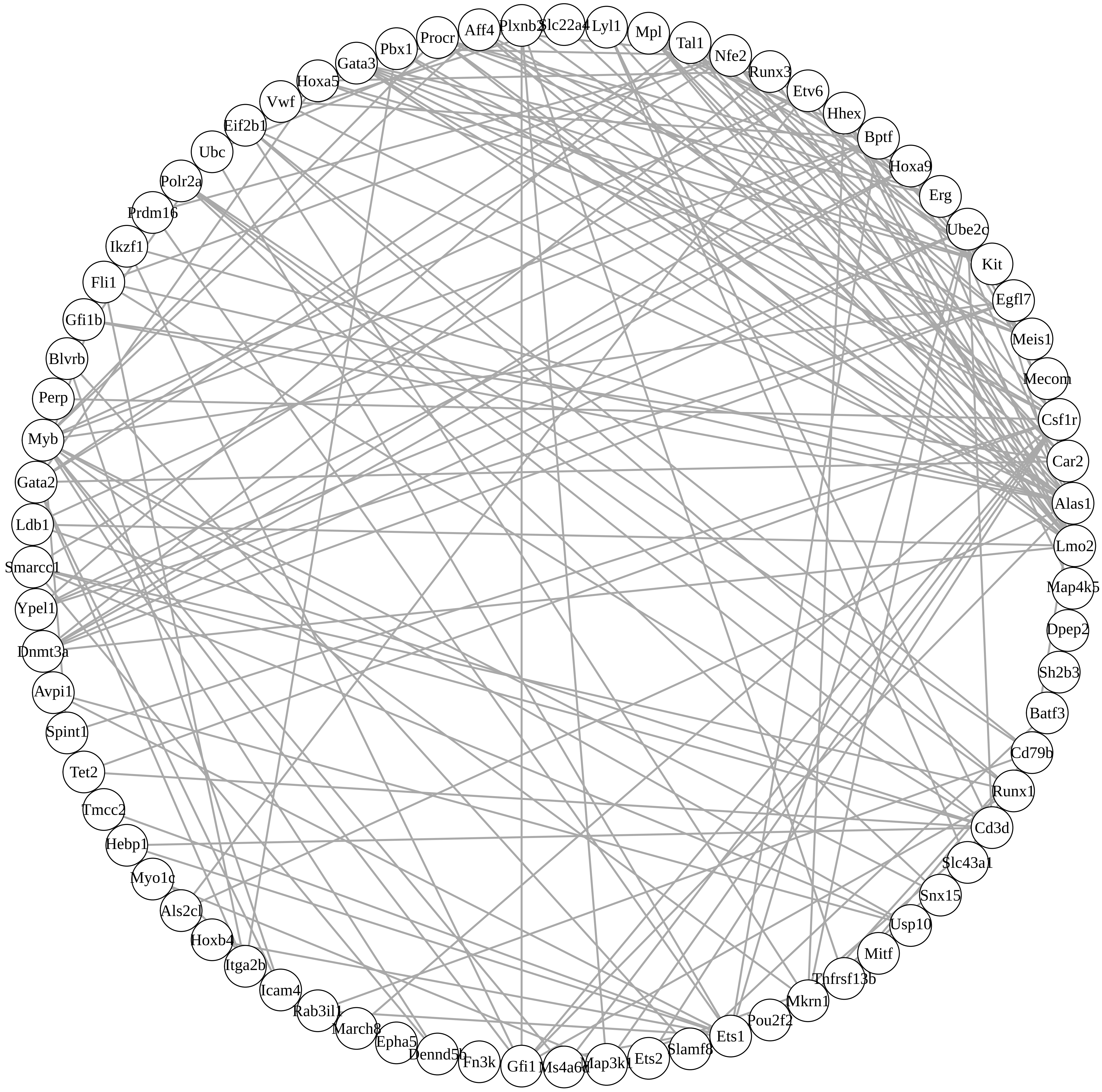

mouse_HSCtoGMP_time 24

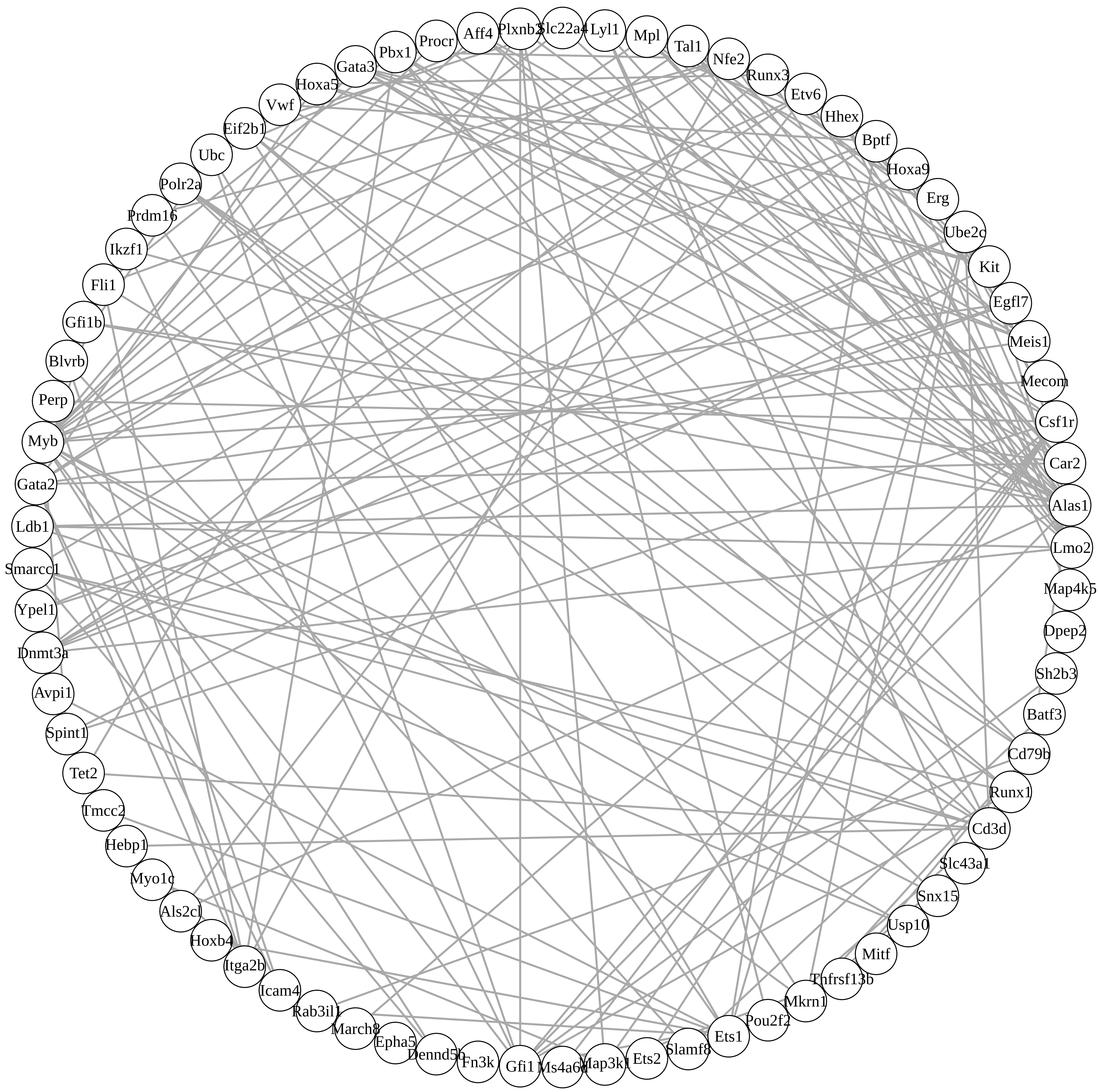

mouse_HSCtoGMP_time 25

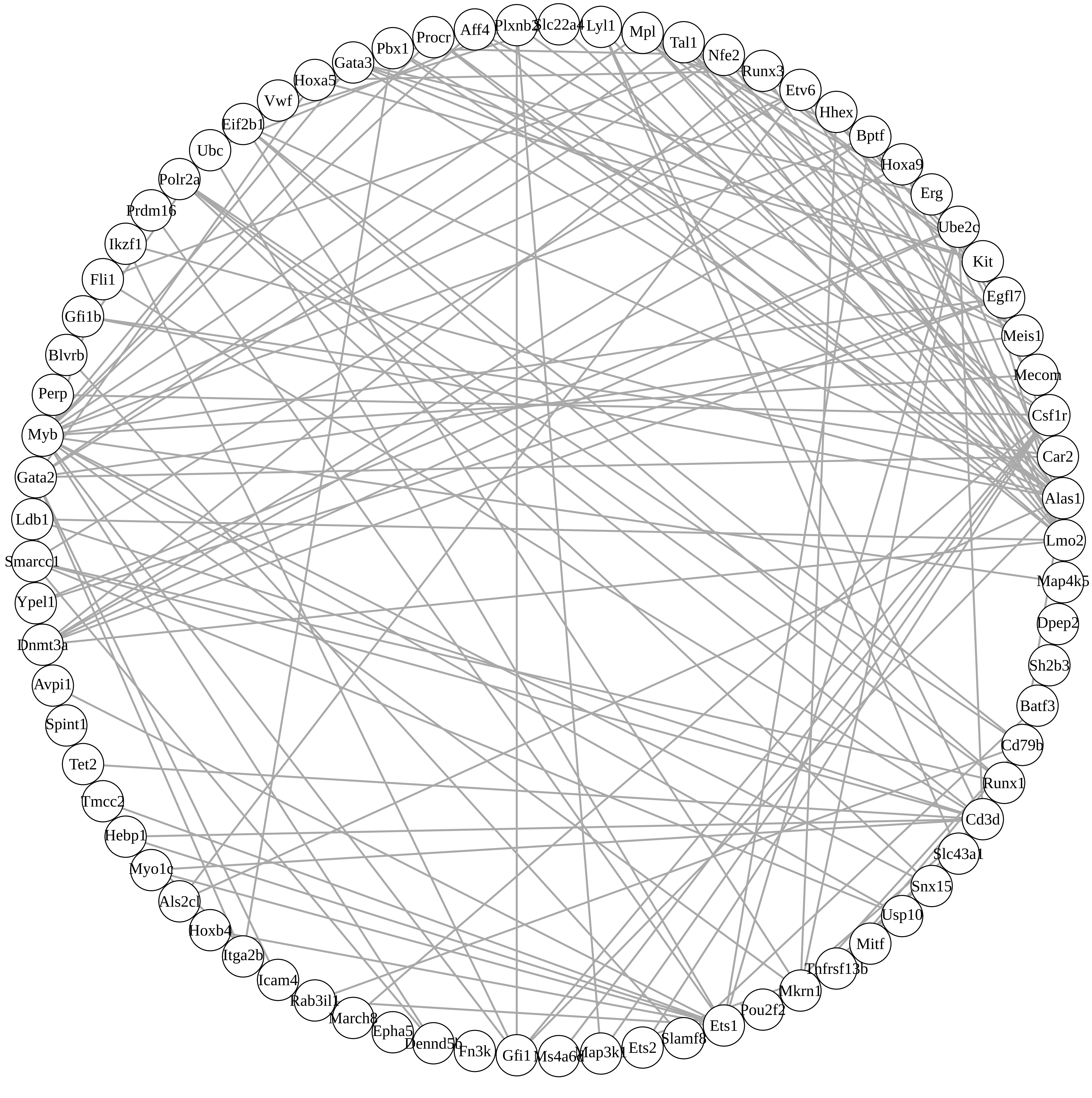

mouse_HSCtoLMPP_time 1

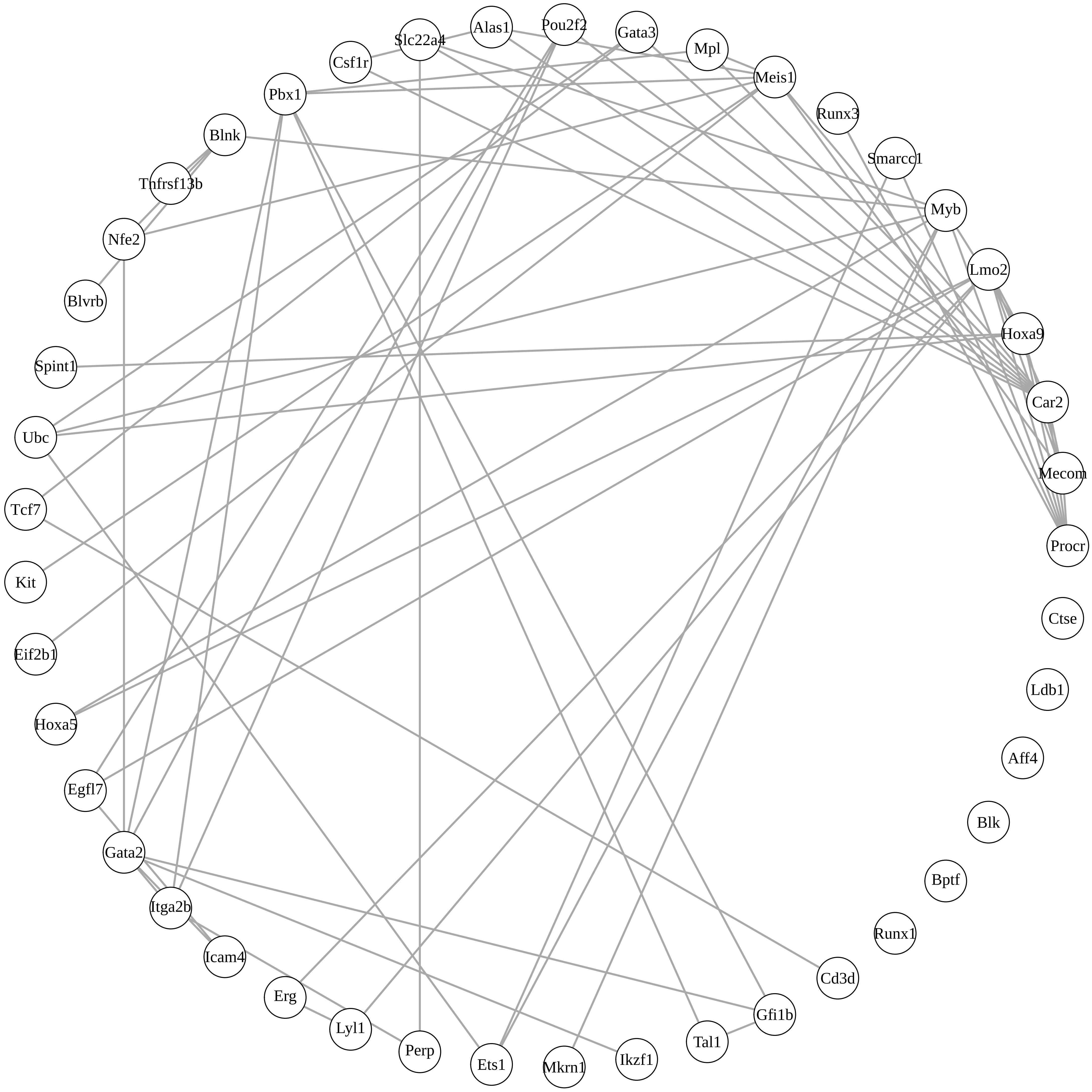

mouse_HSCtoLMPP_time 2

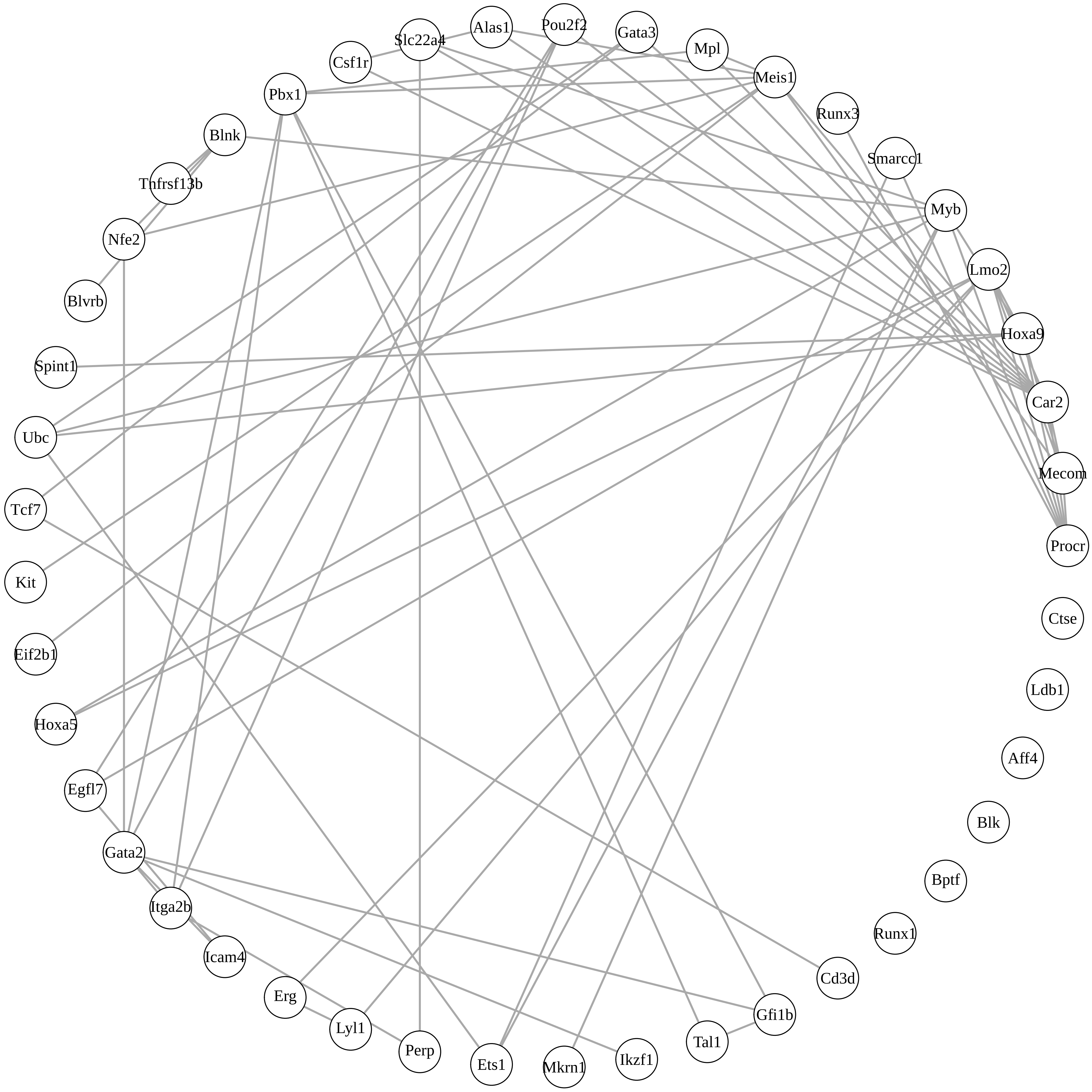

mouse_HSCtoLMPP_time 3

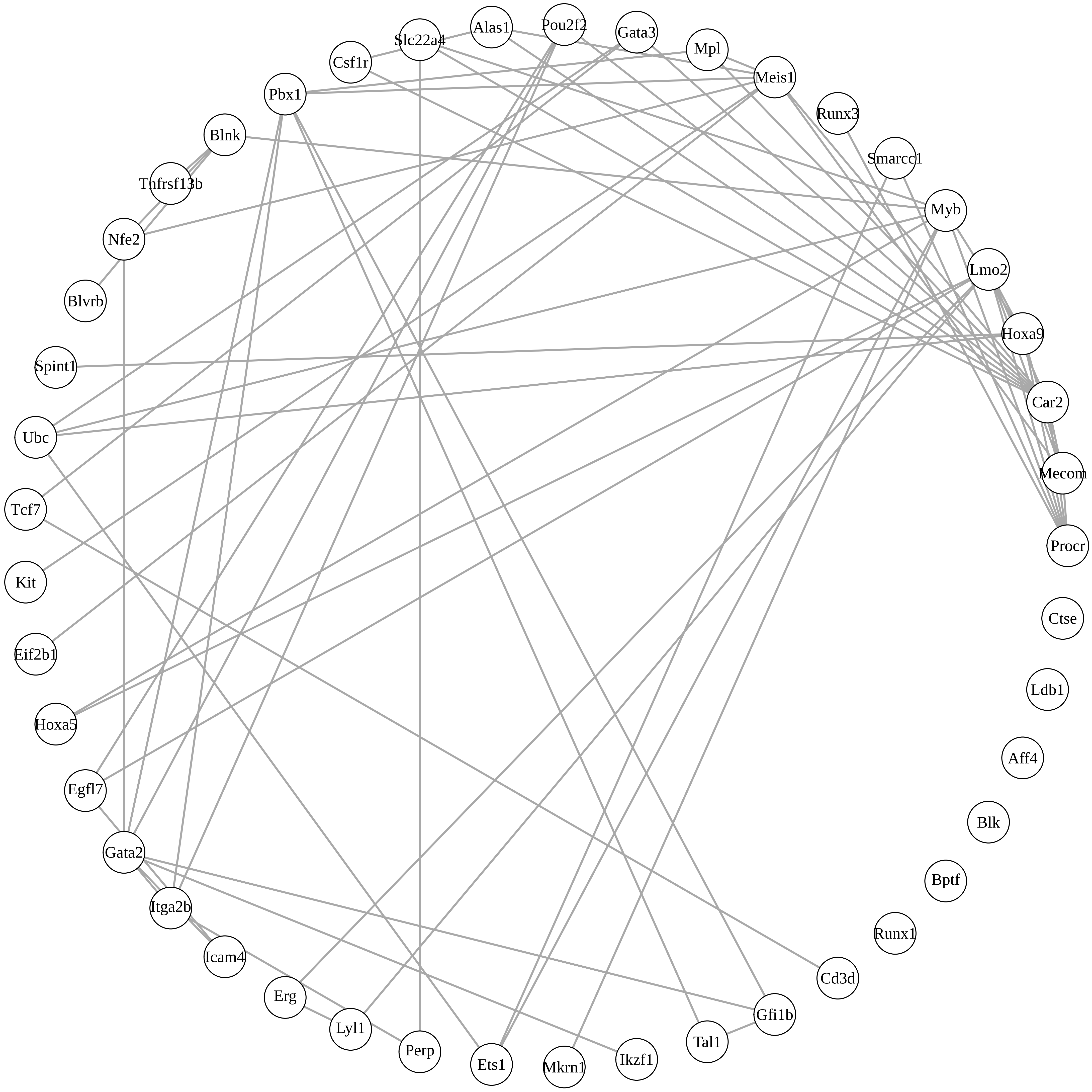

mouse_HSCtoLMPP_time 4

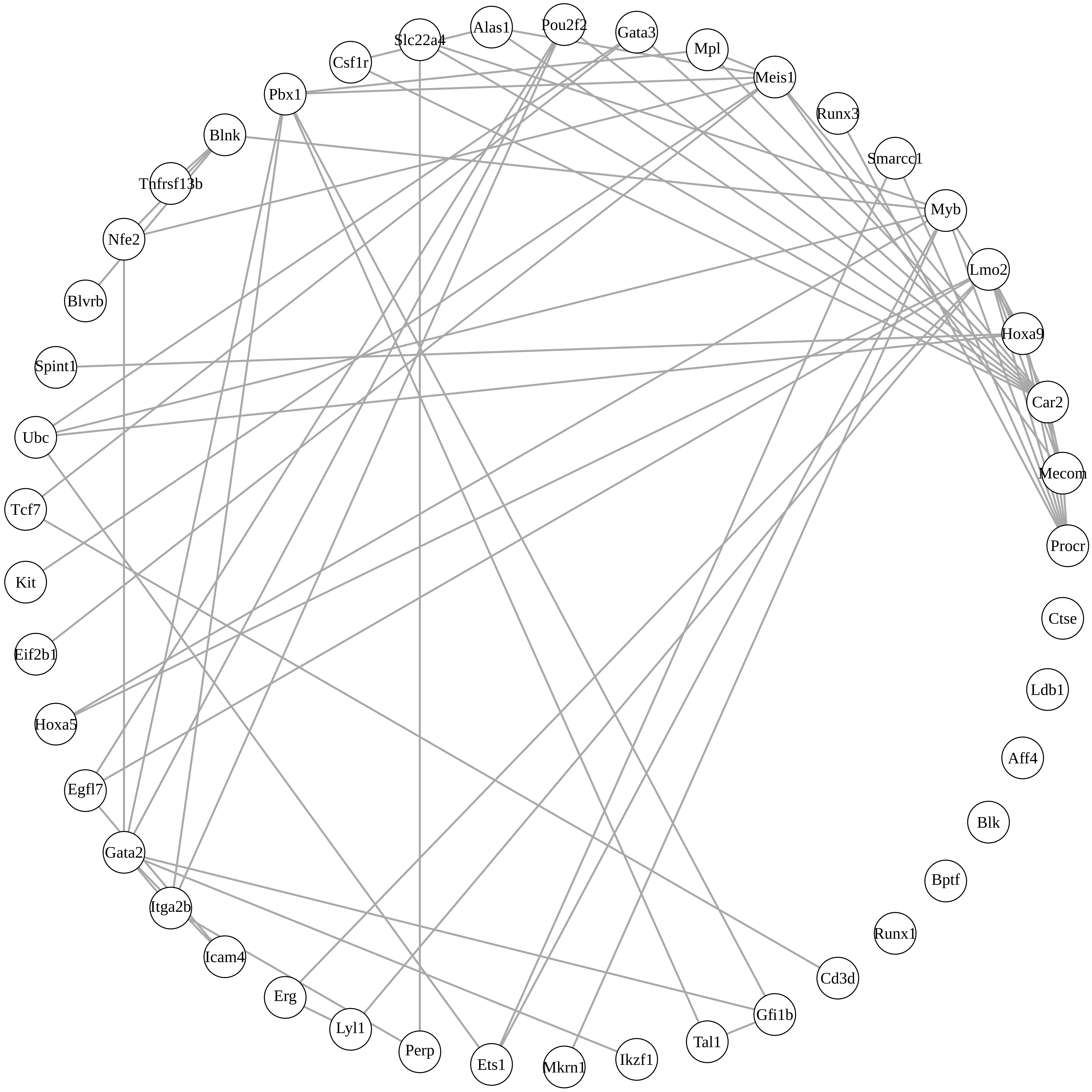

mouse_HSCtoLMPP_time 5

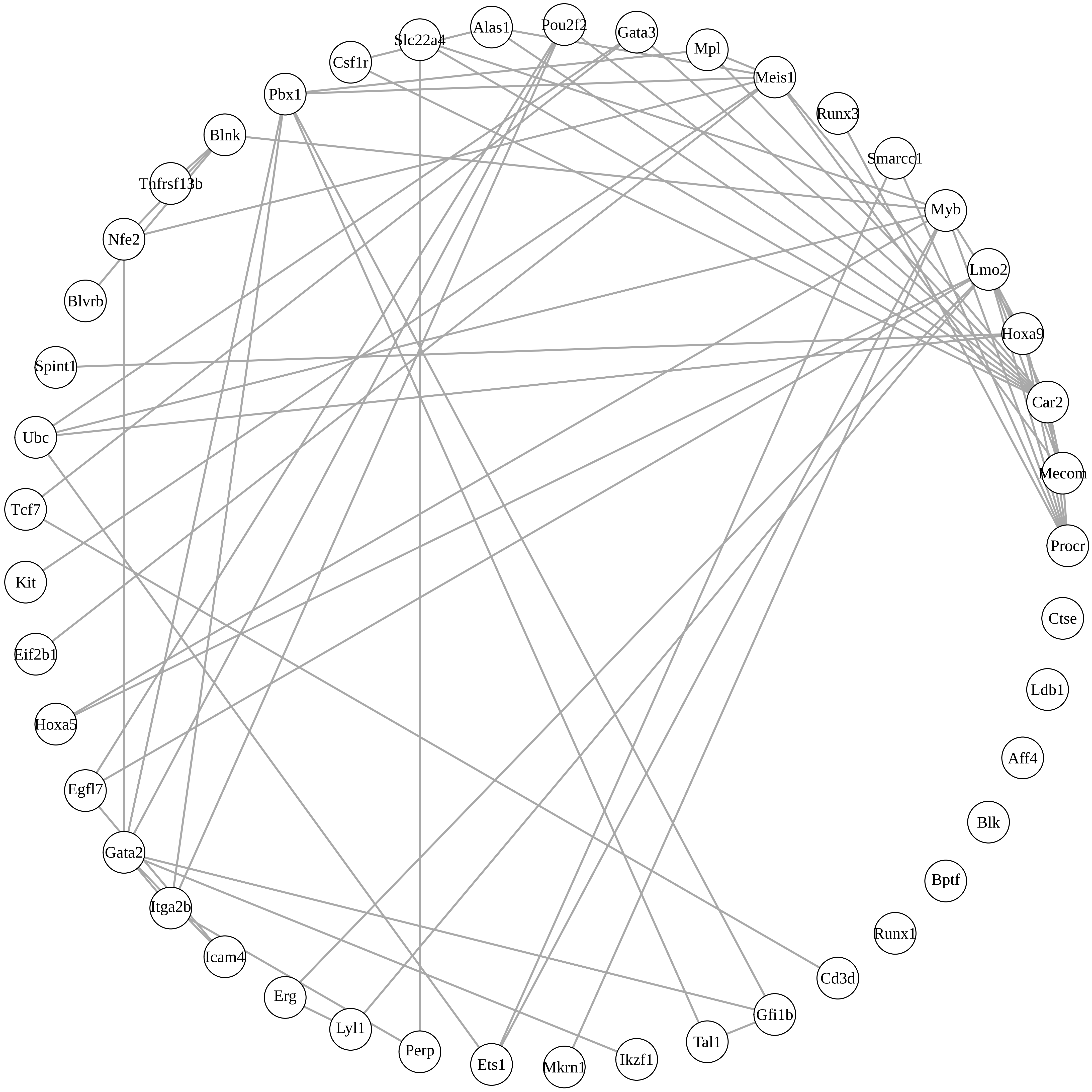

mouse_HSCtoLMPP_time 6

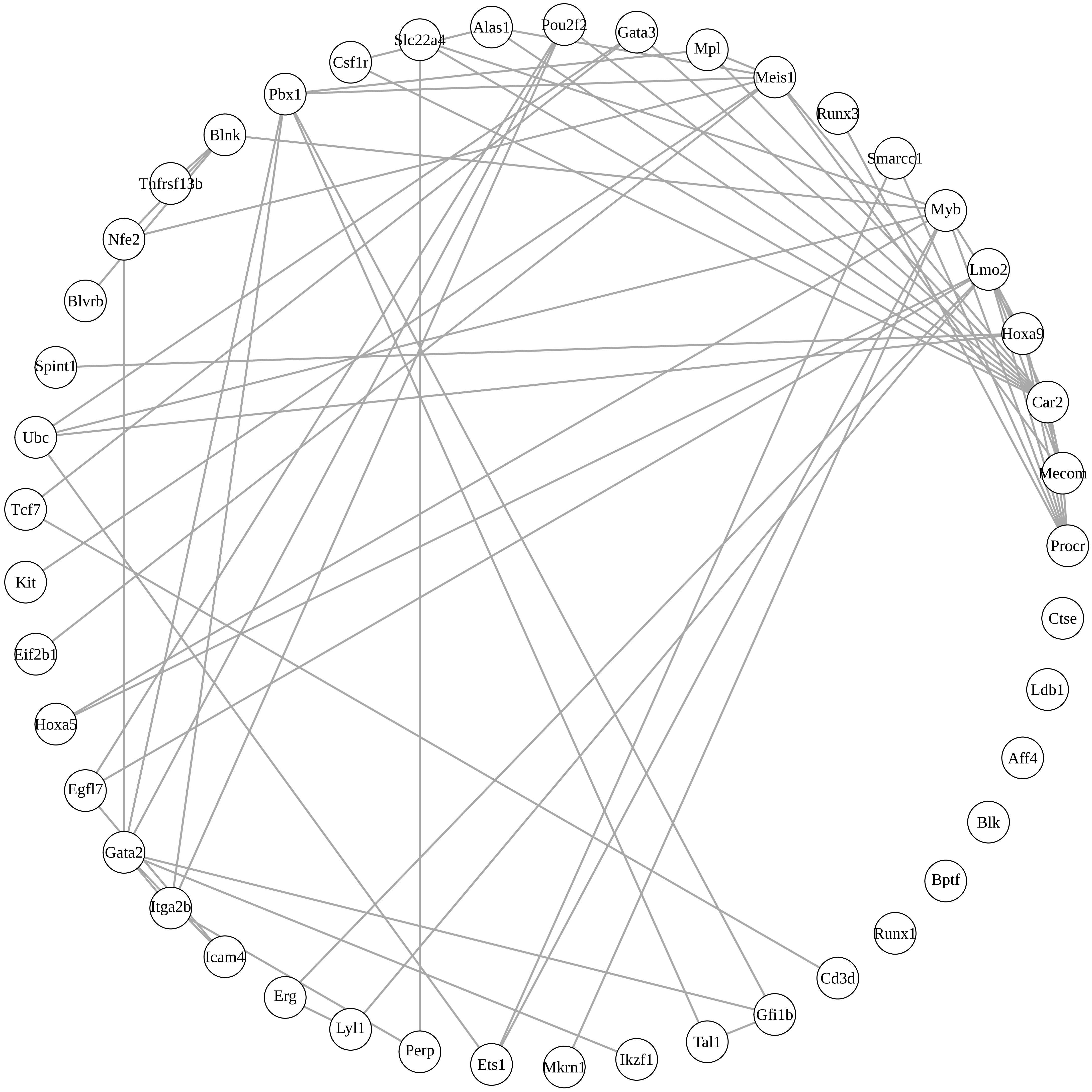

mouse_HSCtoLMPP_time 7

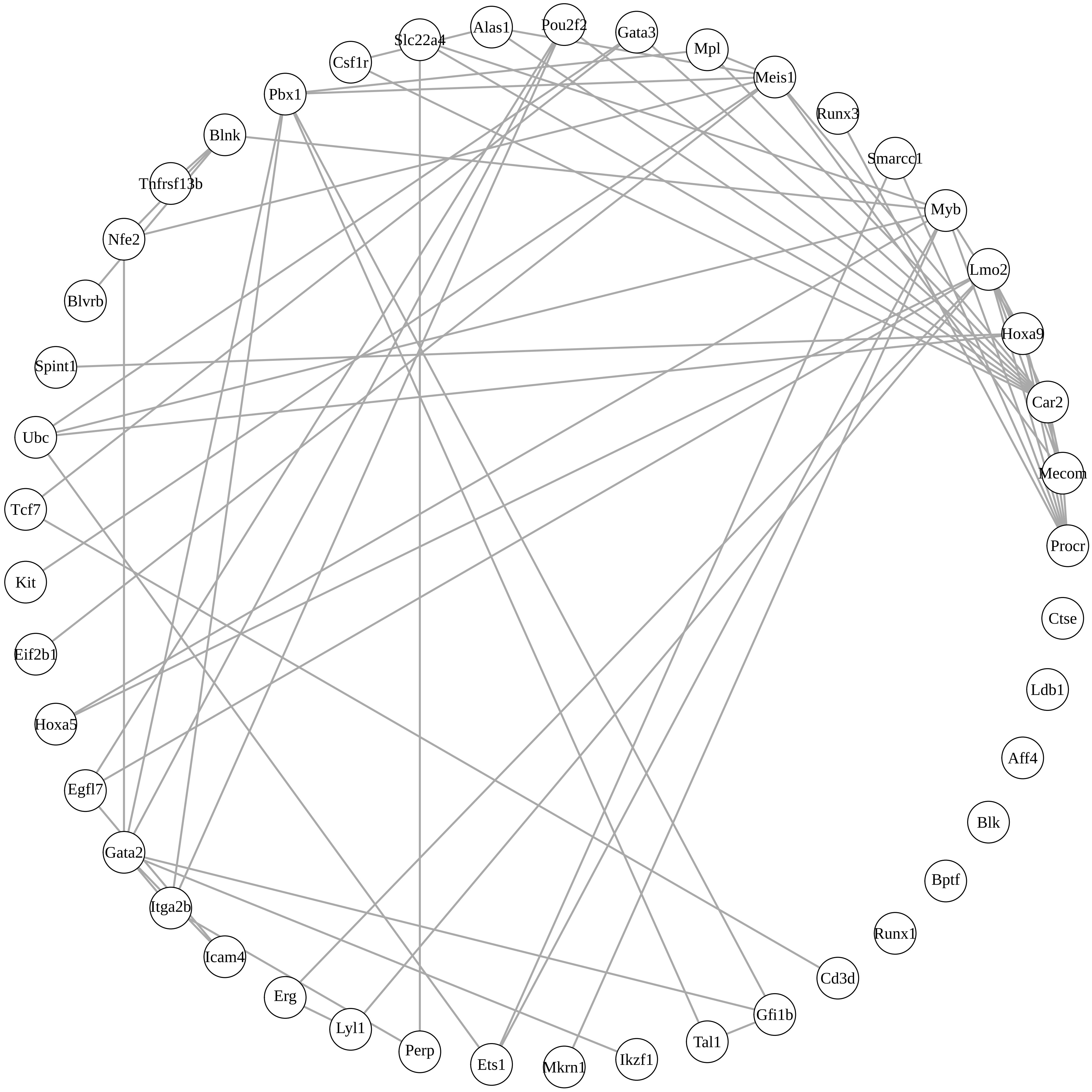

mouse_HSCtoLMPP_time 8

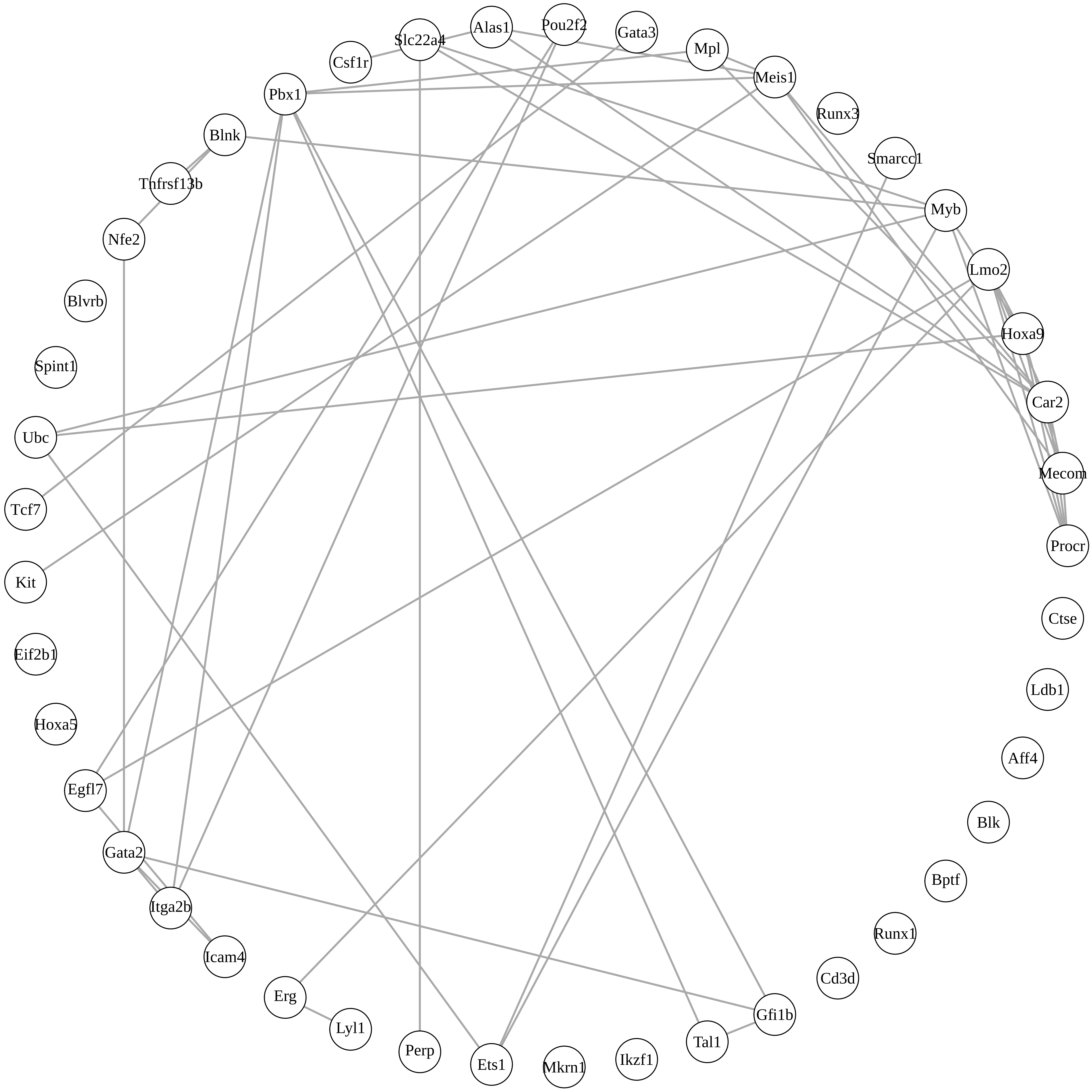

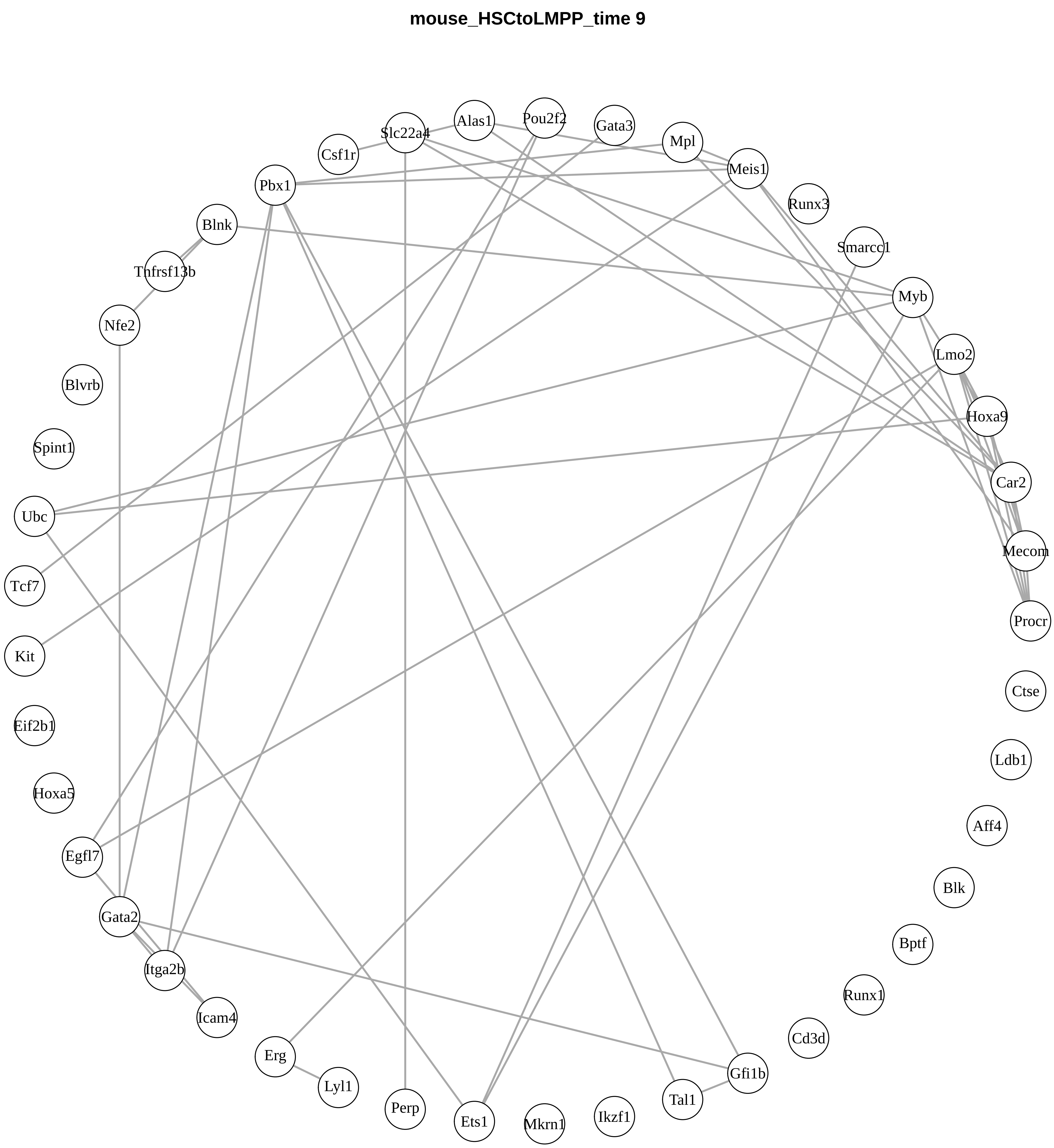
mouse_HSCtoLMPP_time 9
Slc22a4
Alas1
Pou2f2
Gata3
Mpl
Meis1
Csf1r
Pbx1
Runx3
Blnk
Smarcc1
Tnfrsf13b
Myb
Nfe2
Lmo2
Blvrb
Hoxa9
Spint1
Car2
Ubc
Mecom
Tcf7
Procr
Kit
Ctse
Eif2b1
Ldb1
Hoxa5
Aff4
Egfl7
Blk
Gata2
Bptf
Itga2b
Runx1
Icam4
Cd3d
Erg
Gfi1b
Lyl1
Tal1
Perp
Ets1
Mkrn1
Ikzf1

mouse_HSCtoLMPP_time 10

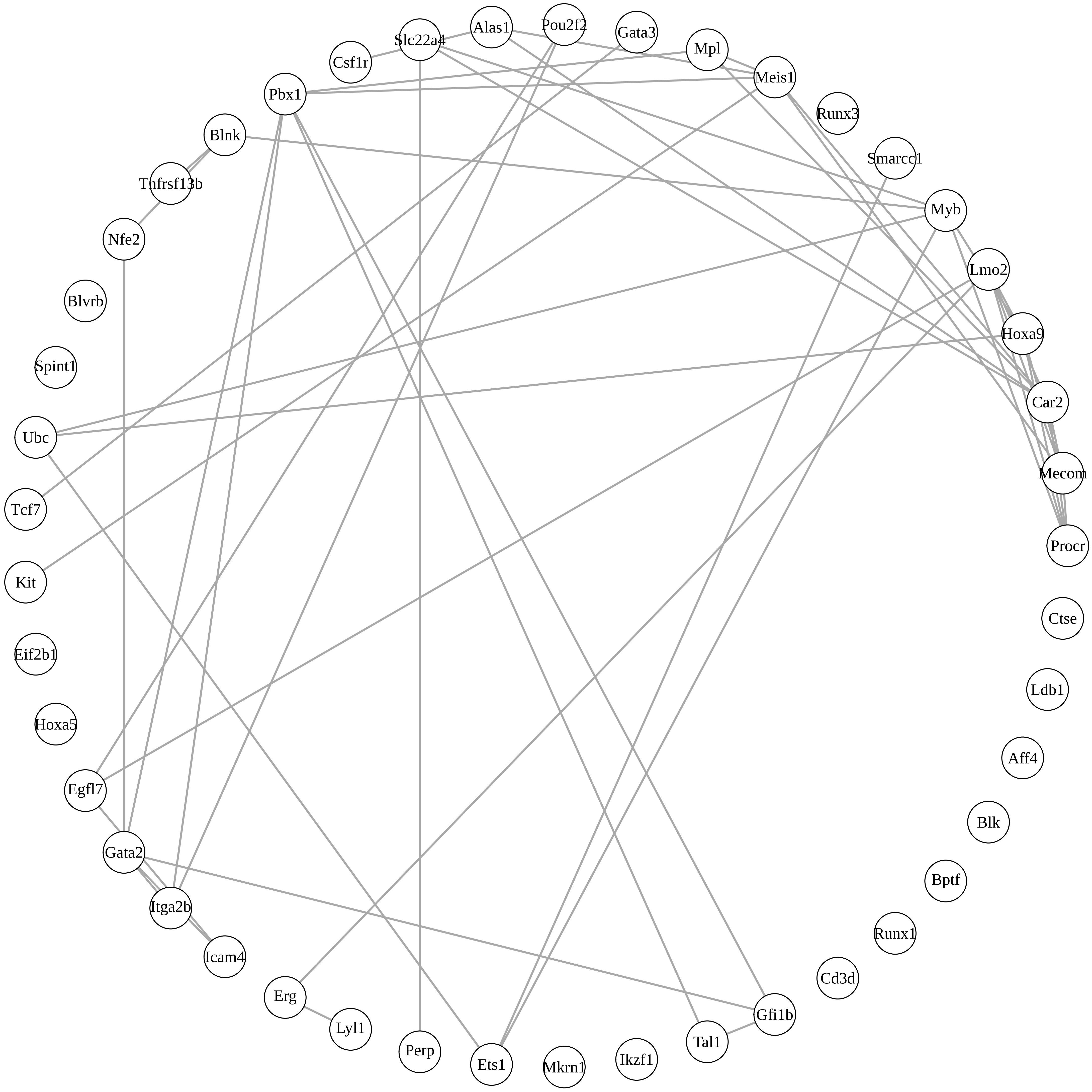

mouse_HSCtoLMPP_time 11

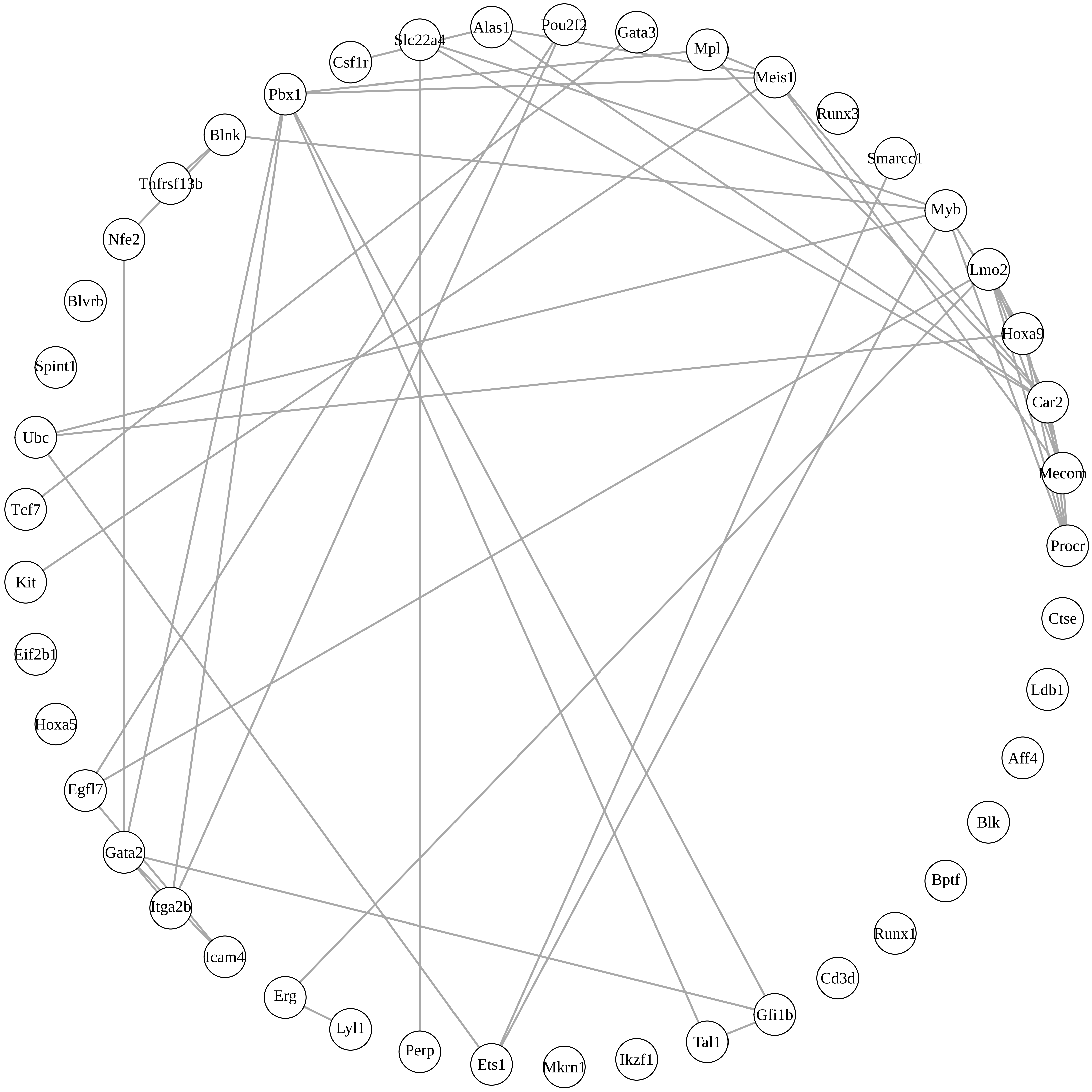

mouse_HSCtoLMPP_time 12

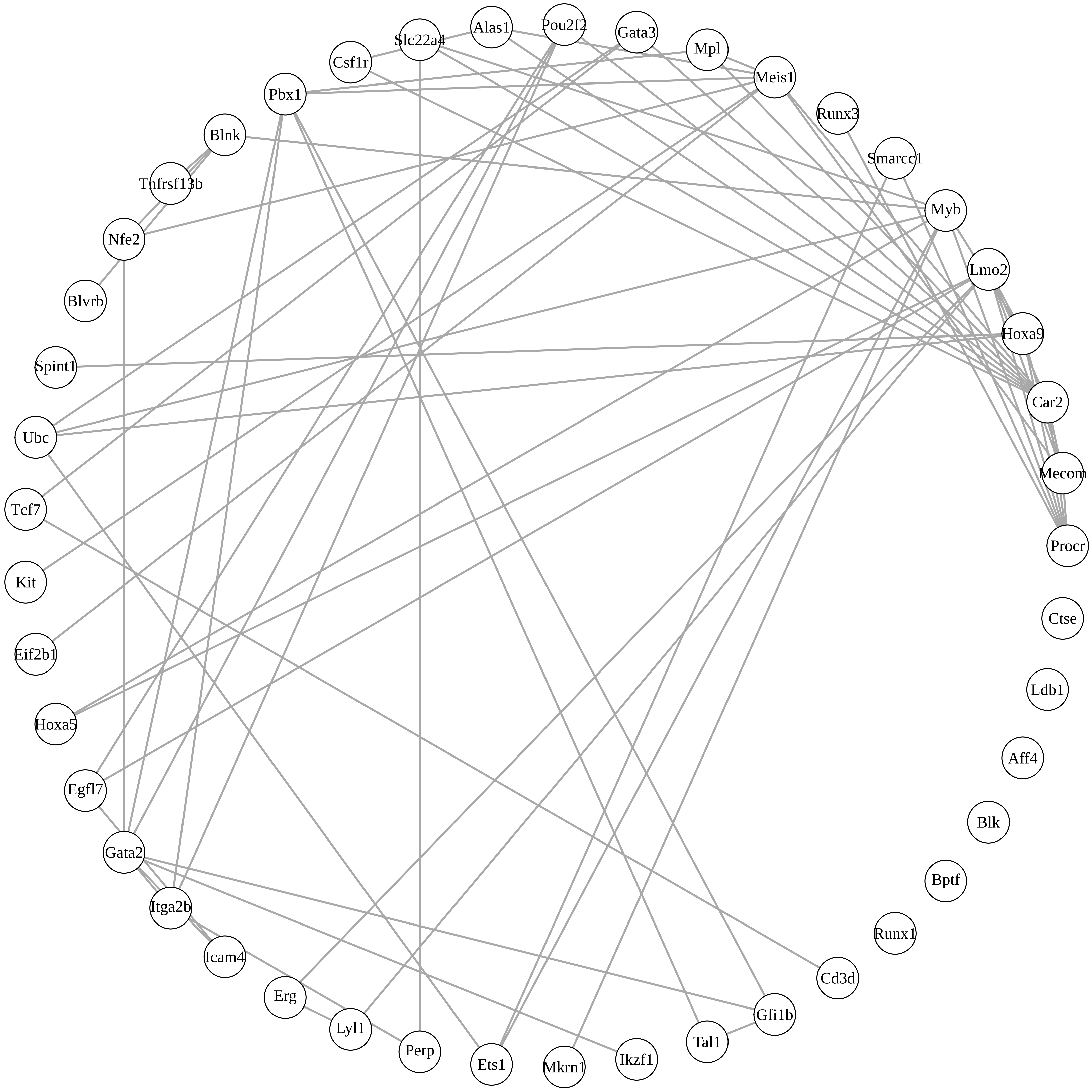

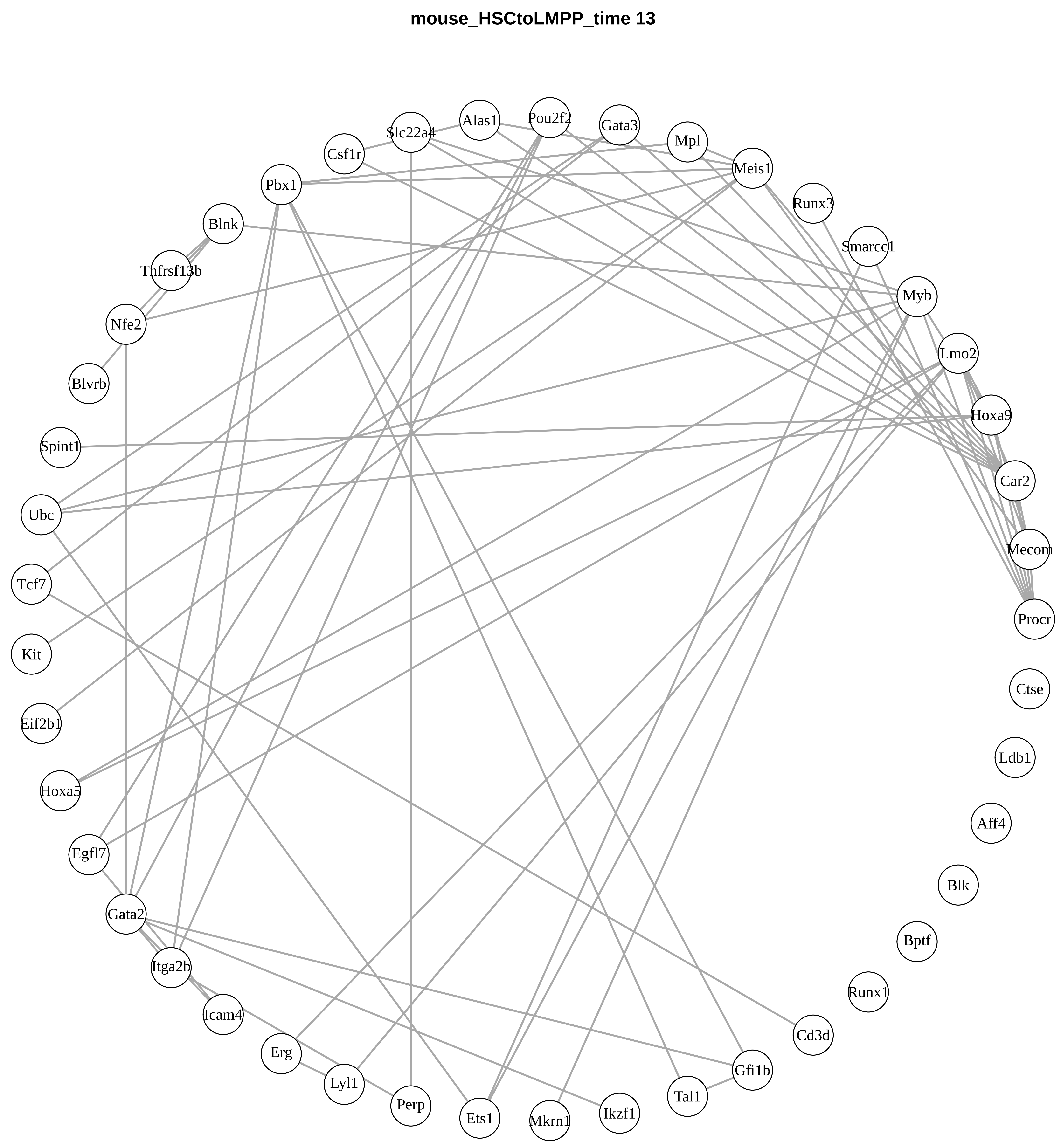
mouse_HSCtoLMPP_time 13
Slc22a4
Alas1
Pou2f2
Gata3
Mpl
Csf1r
Meis1
Pbx1
Runx3
Blnk
Smarcc1
Tnfrsf13b
Myb
Nfe2
Lmo2
Blvrb
Hoxa9
Spint1
Car2
Ubc
Mecom
Tcf7
Procr
Kit
Ctse
Eif2b1
Ldb1
Hoxa5
Aff4
Egfl7
Blk
Gata2
Bptf
Itga2b
Runx1
Icam4
Cd3d
Erg
Gfi1b
Lyl1
Perp
Ets1
Mkrn1
Ikzf1
Tal1

mouse_HSCtoLMPP_time 14

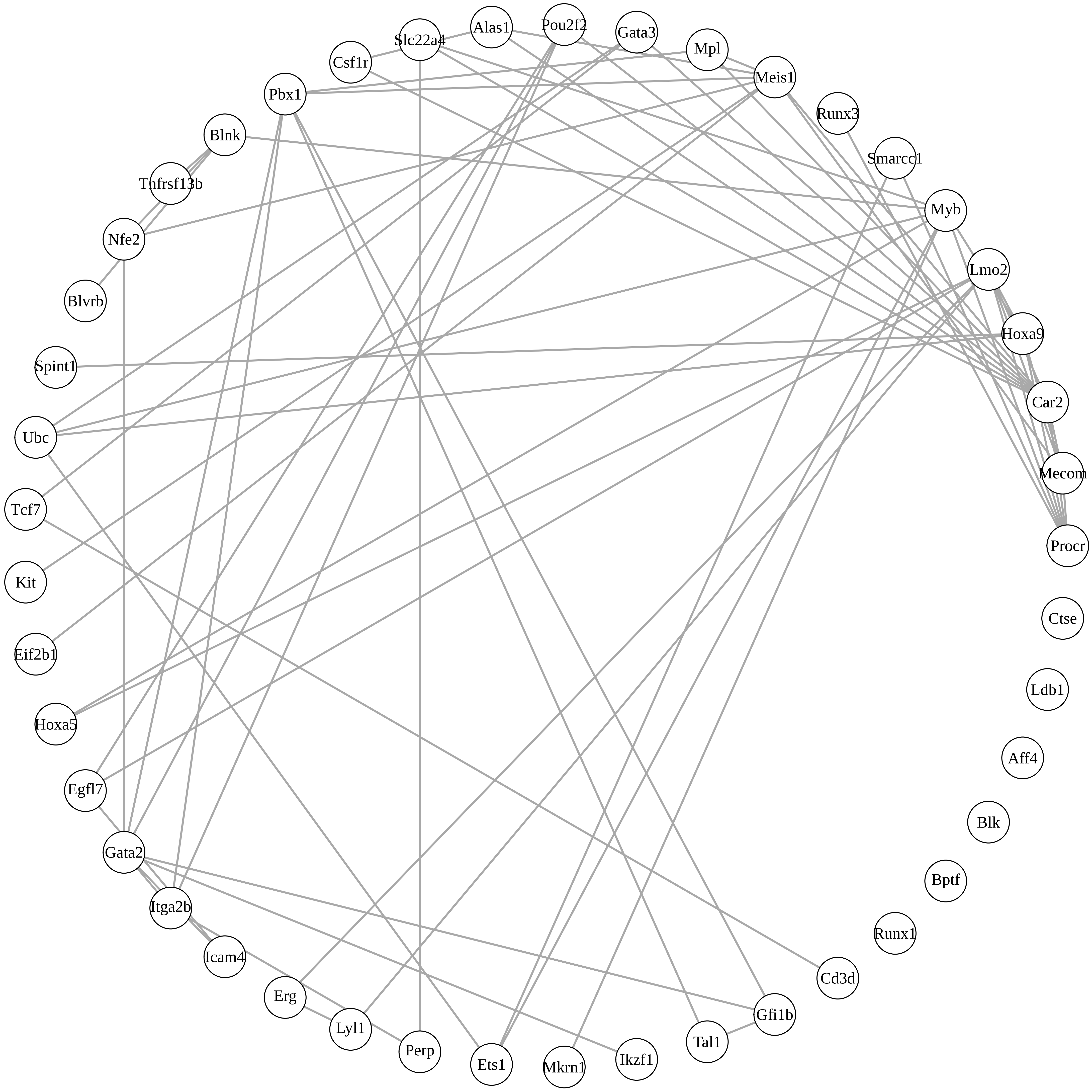

mouse_HSCtoLMPP_time 15

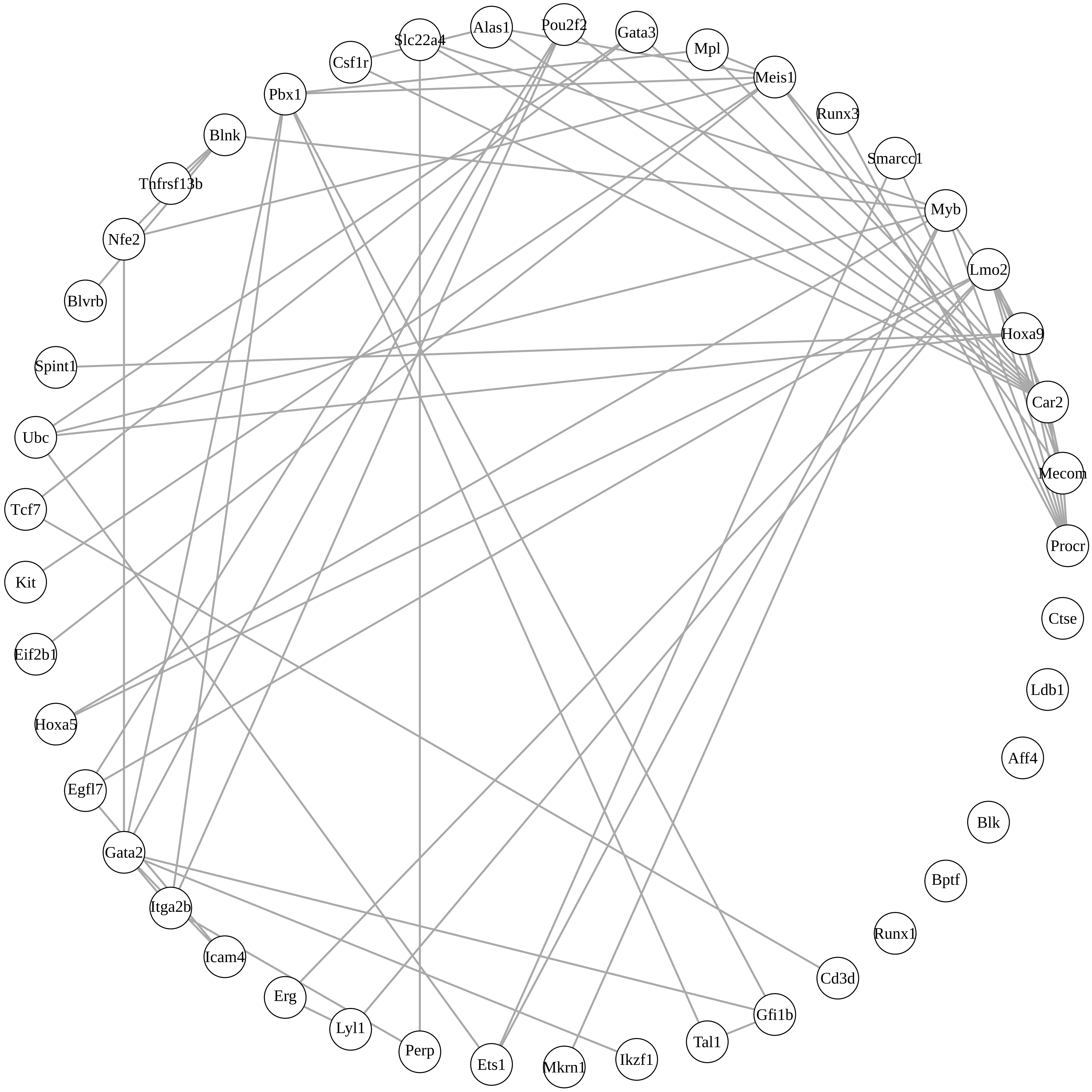

mouse_HSCtoLMPP_time 16

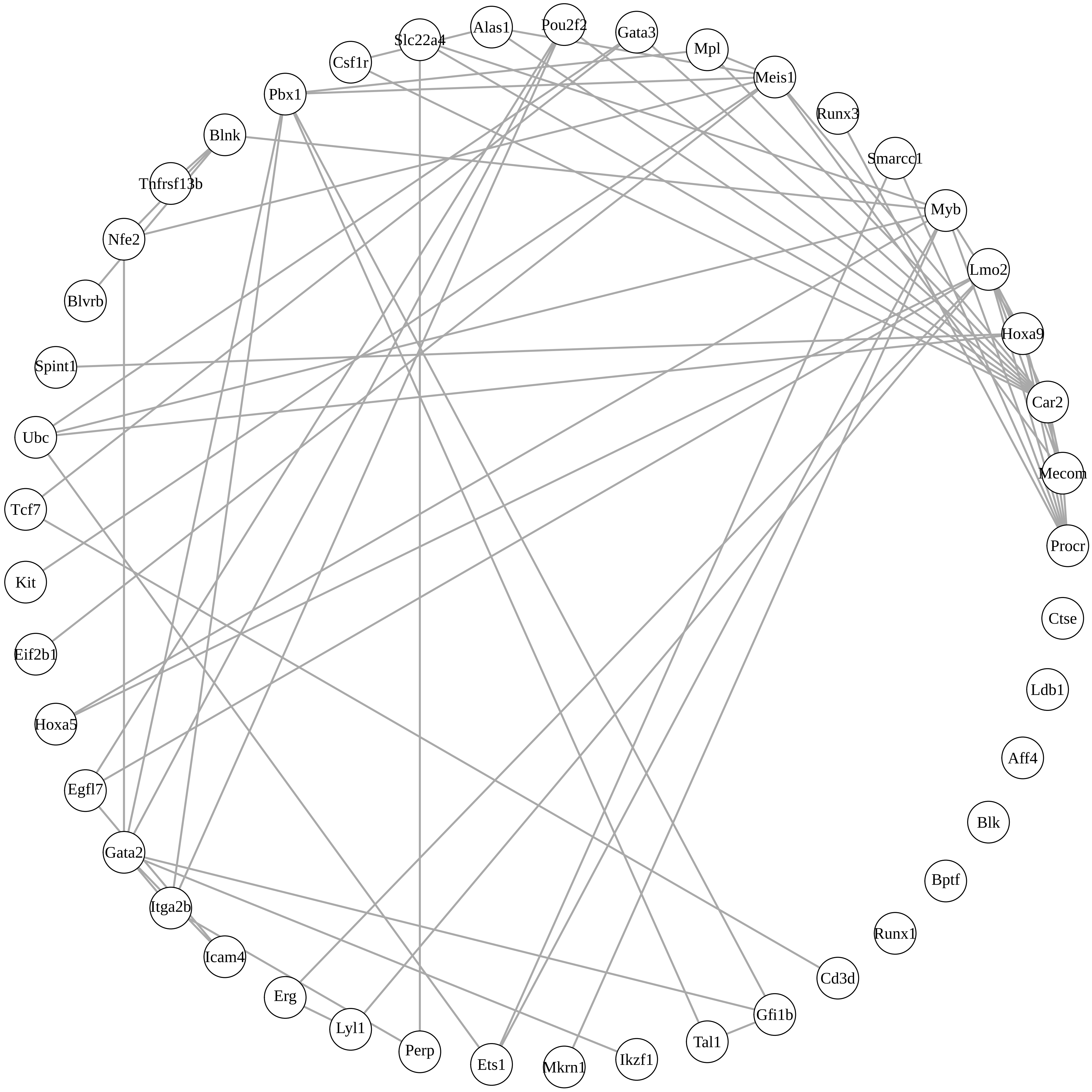

mouse_HSCtoLMPP_time 17

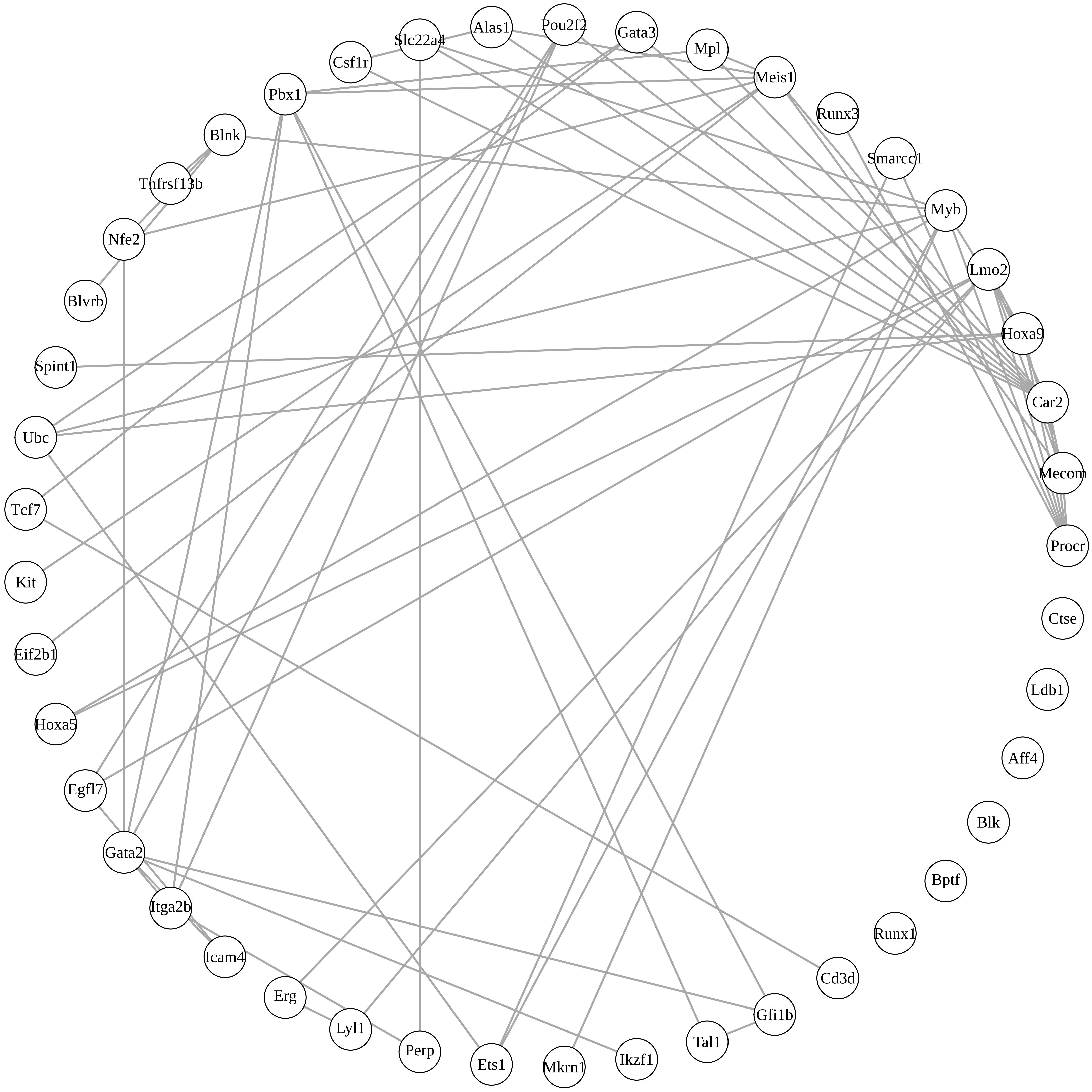

mouse_HSCtoLMPP_time 18

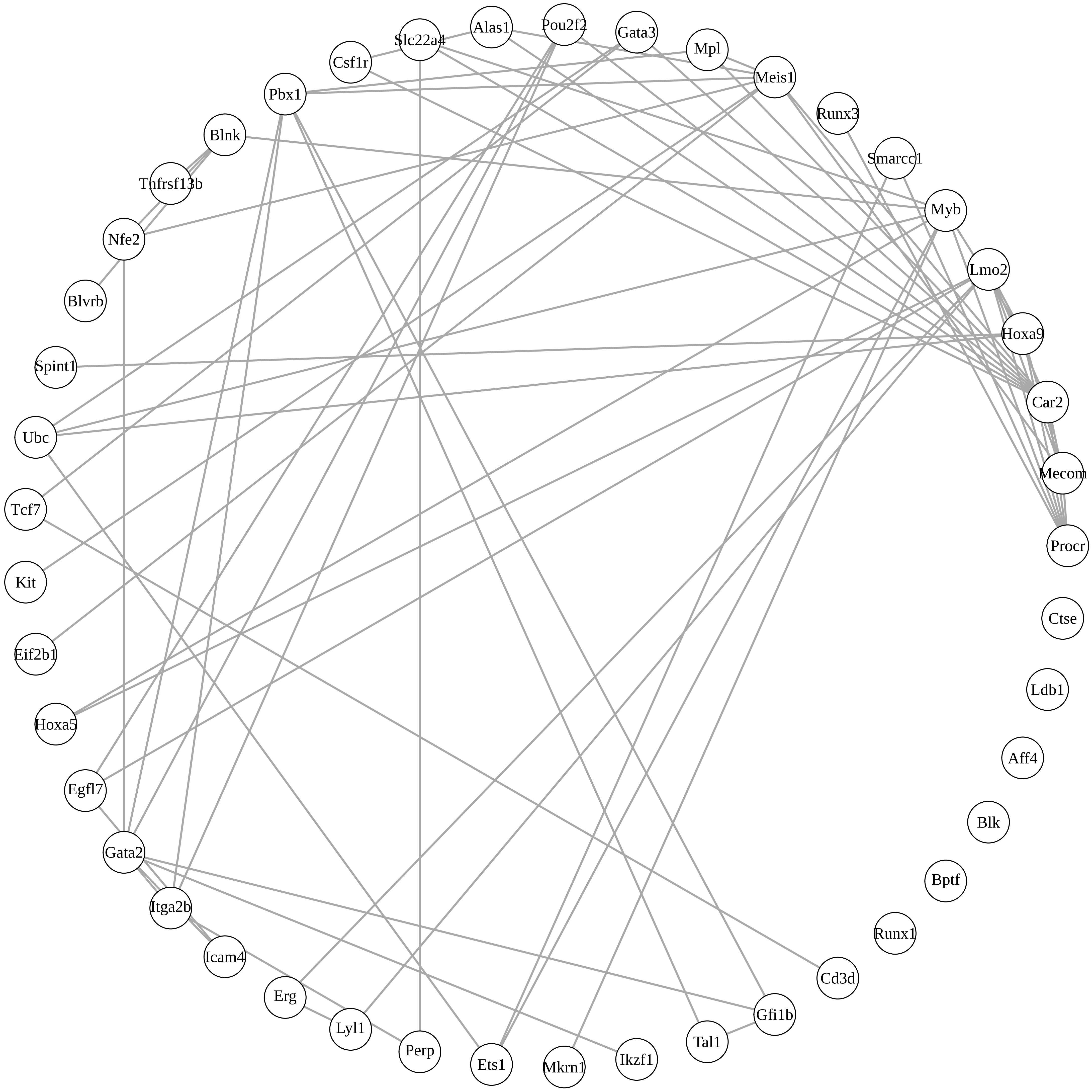

mouse_HSCtoLMPP_time 19

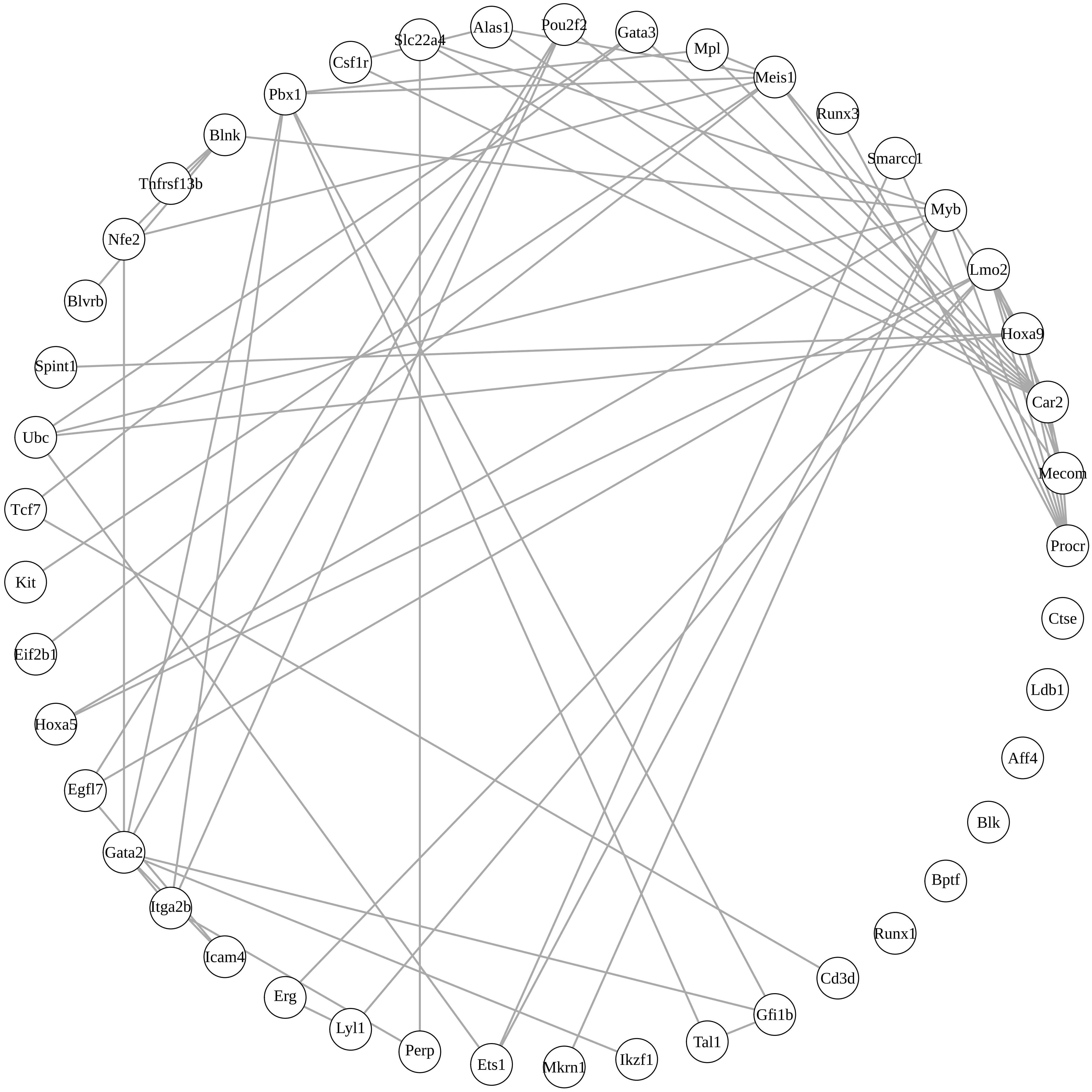

mouse_HSCtoLMPP_time 20

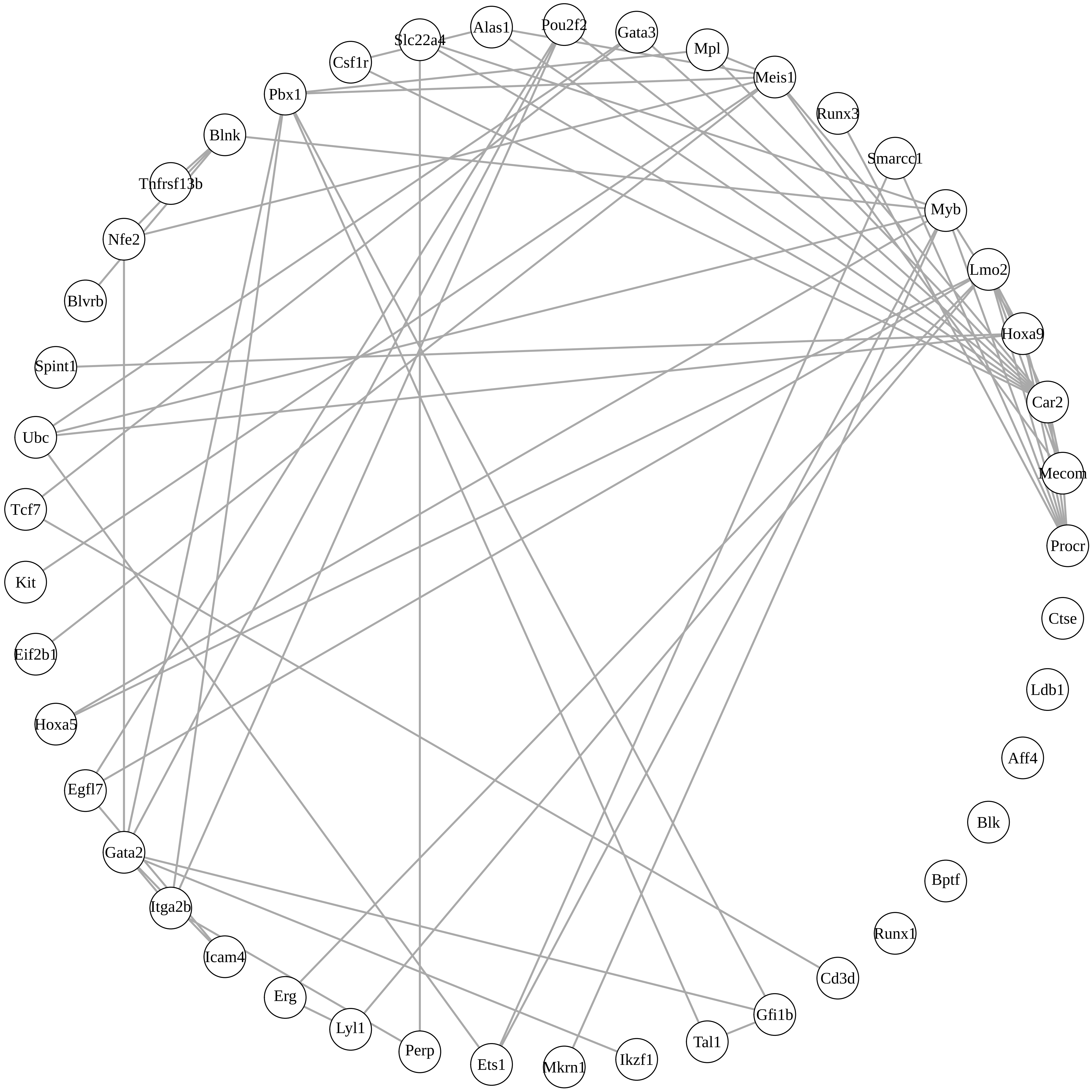

mouse_HSCtoLMPP_time 21

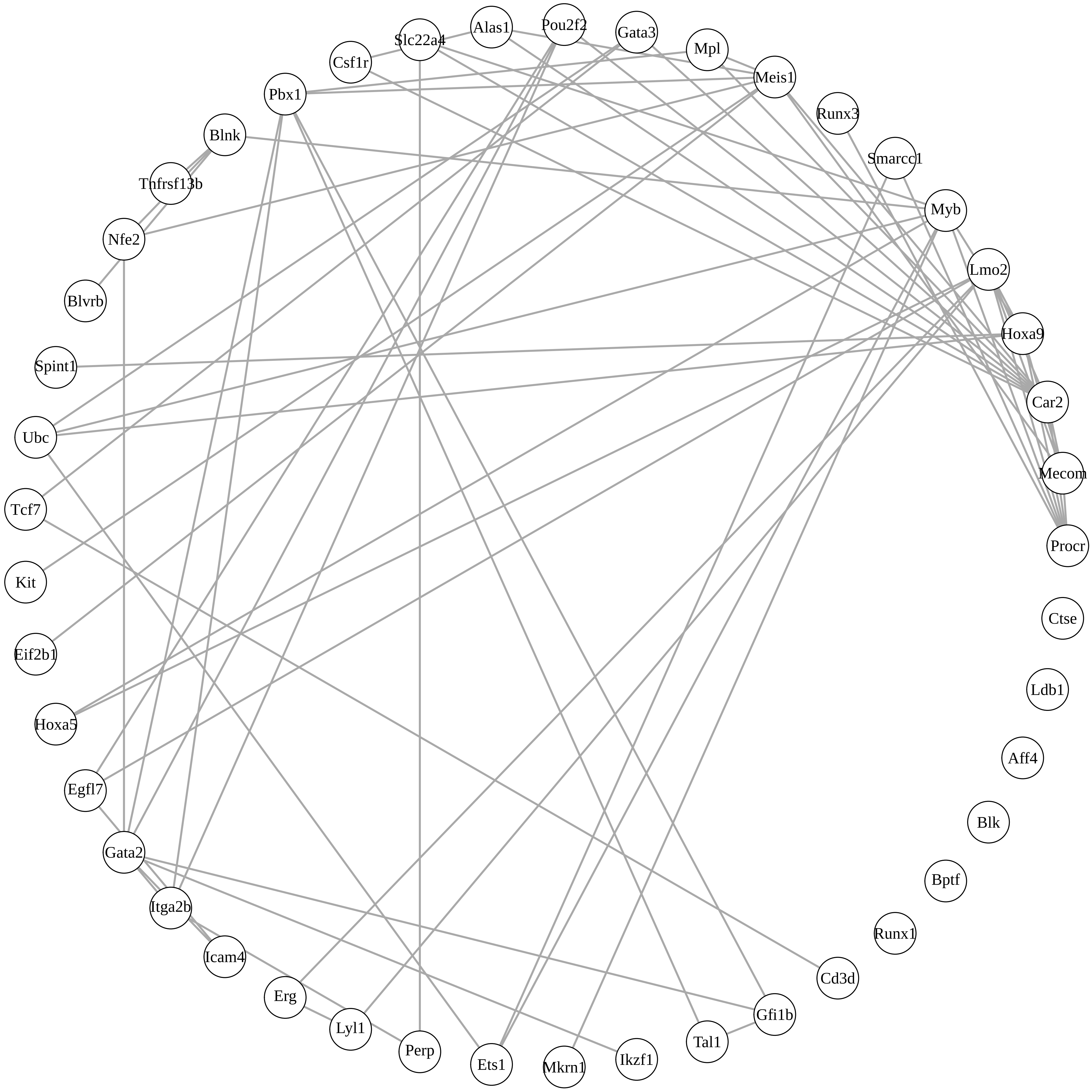

mouse_HSCtoLMPP_time 22

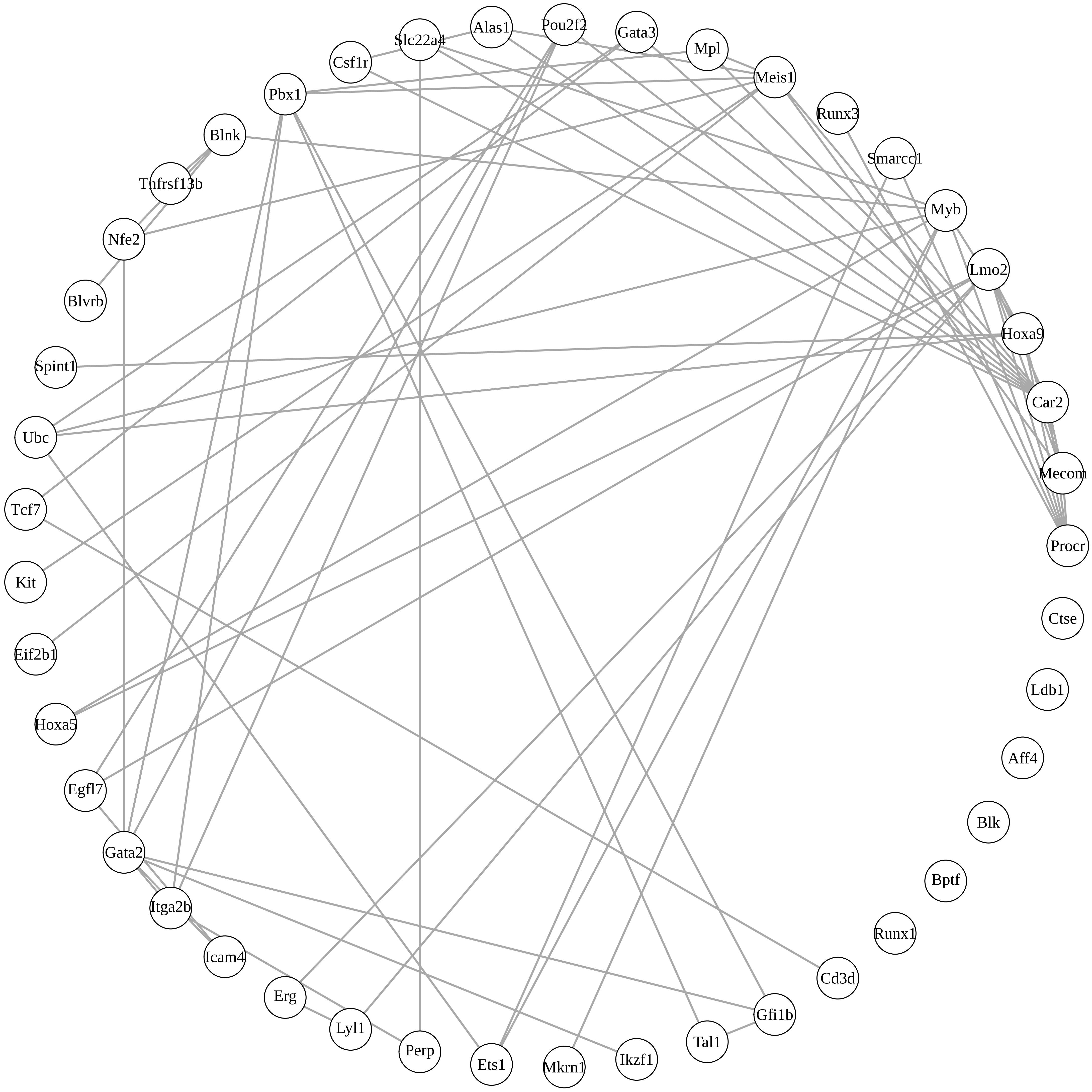

mouse_HSCtoLMPP_time 23

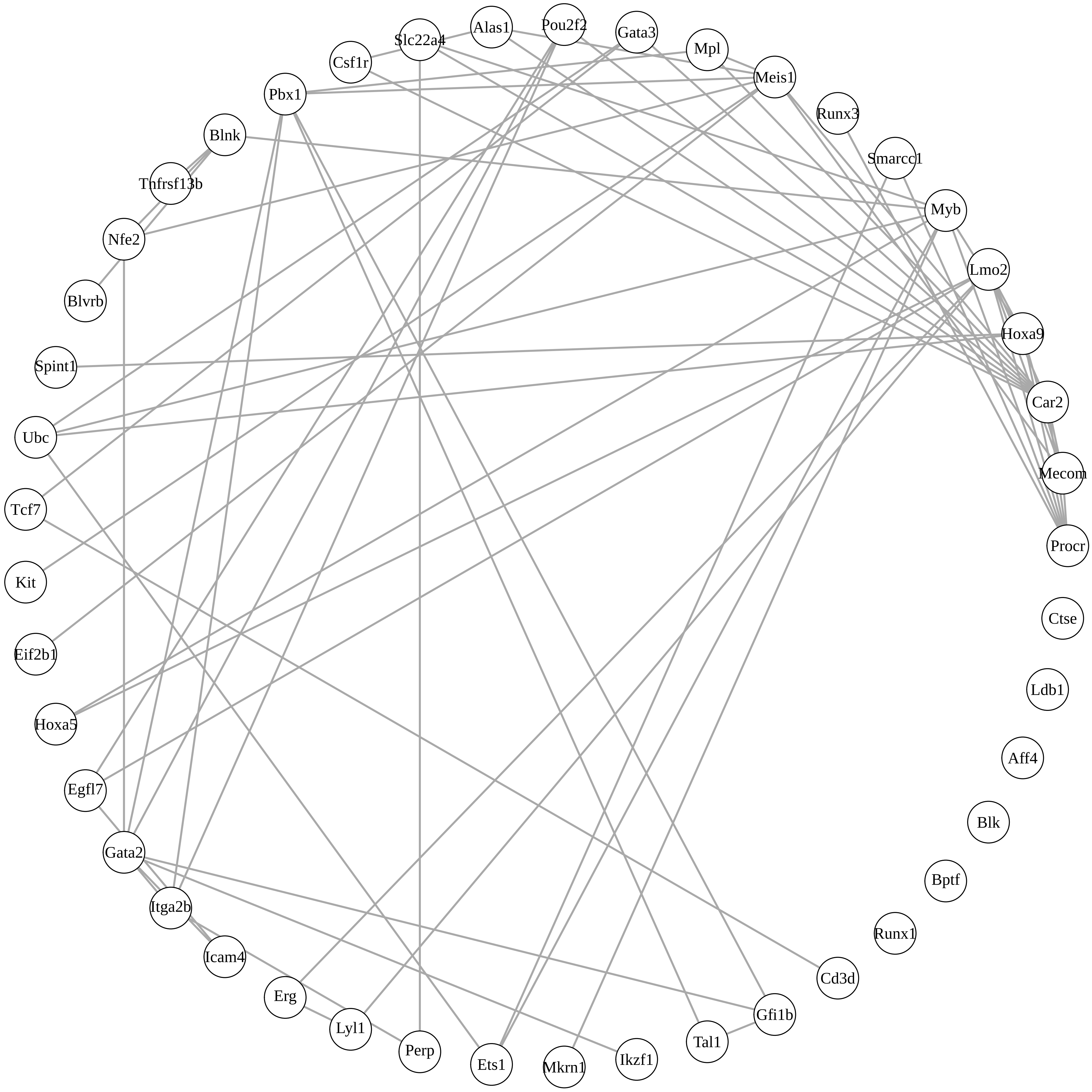

mouse_HSCtoLMPP_time 24

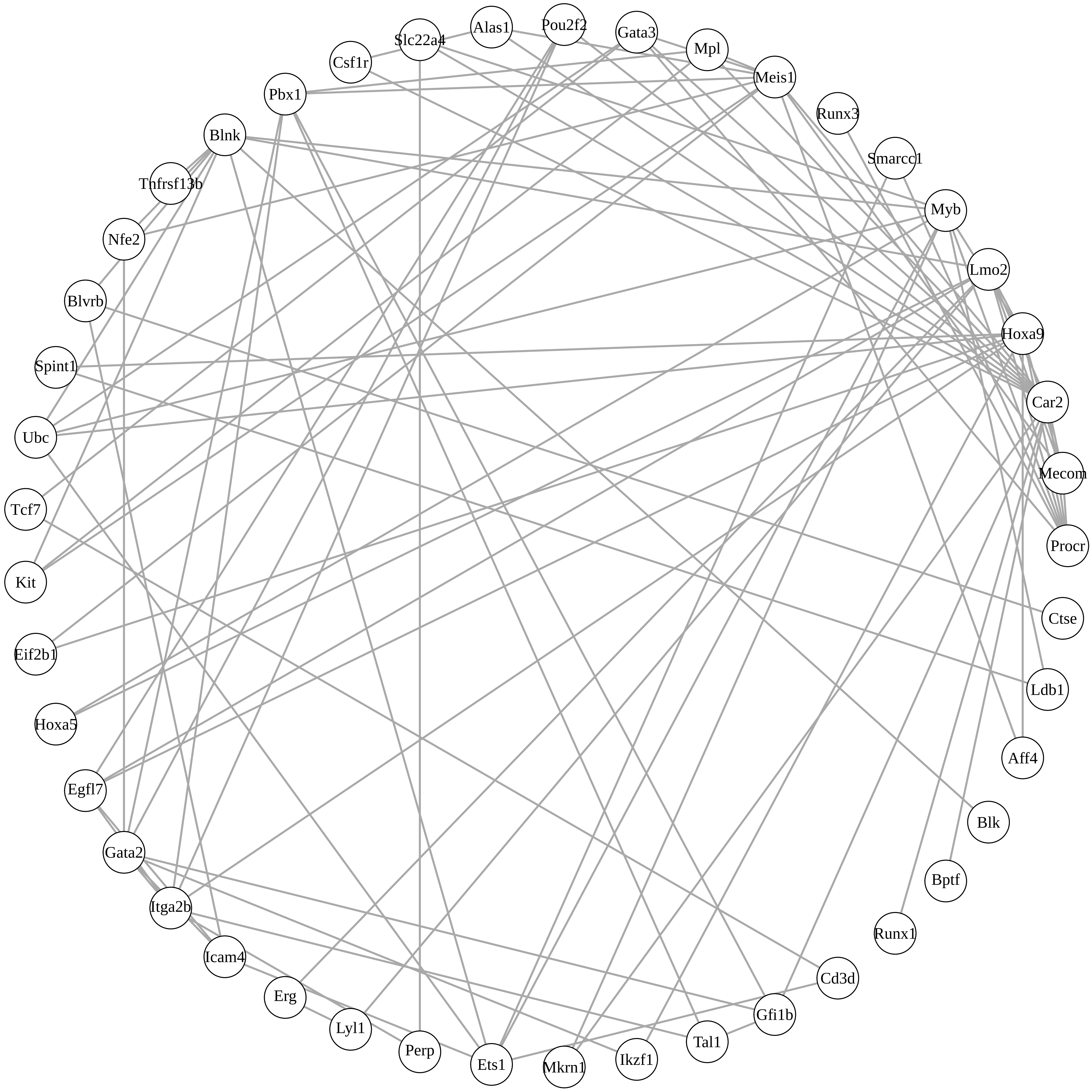

mouse_HSCtoLMPP_time 25

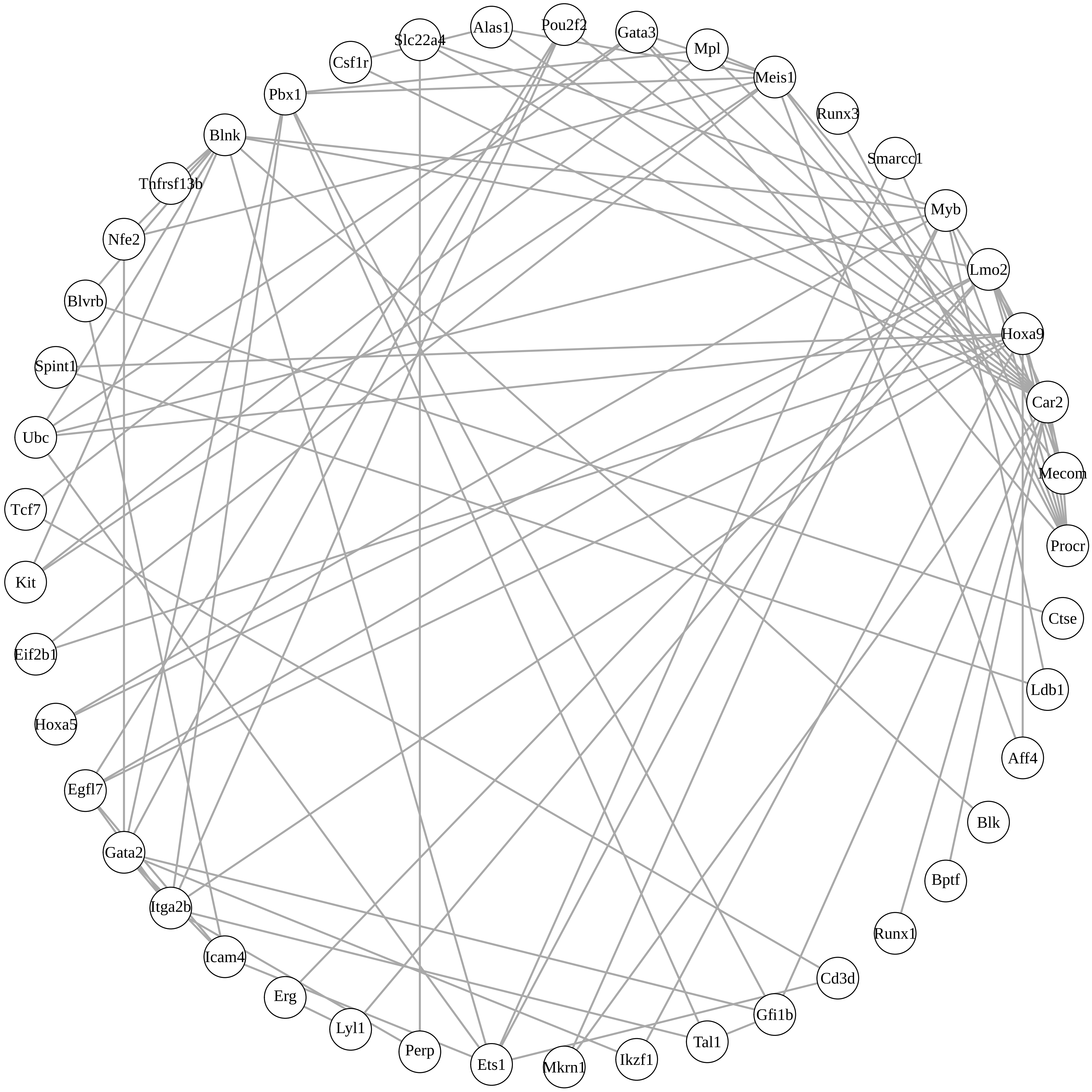

human_HSCtoMEP_time 1

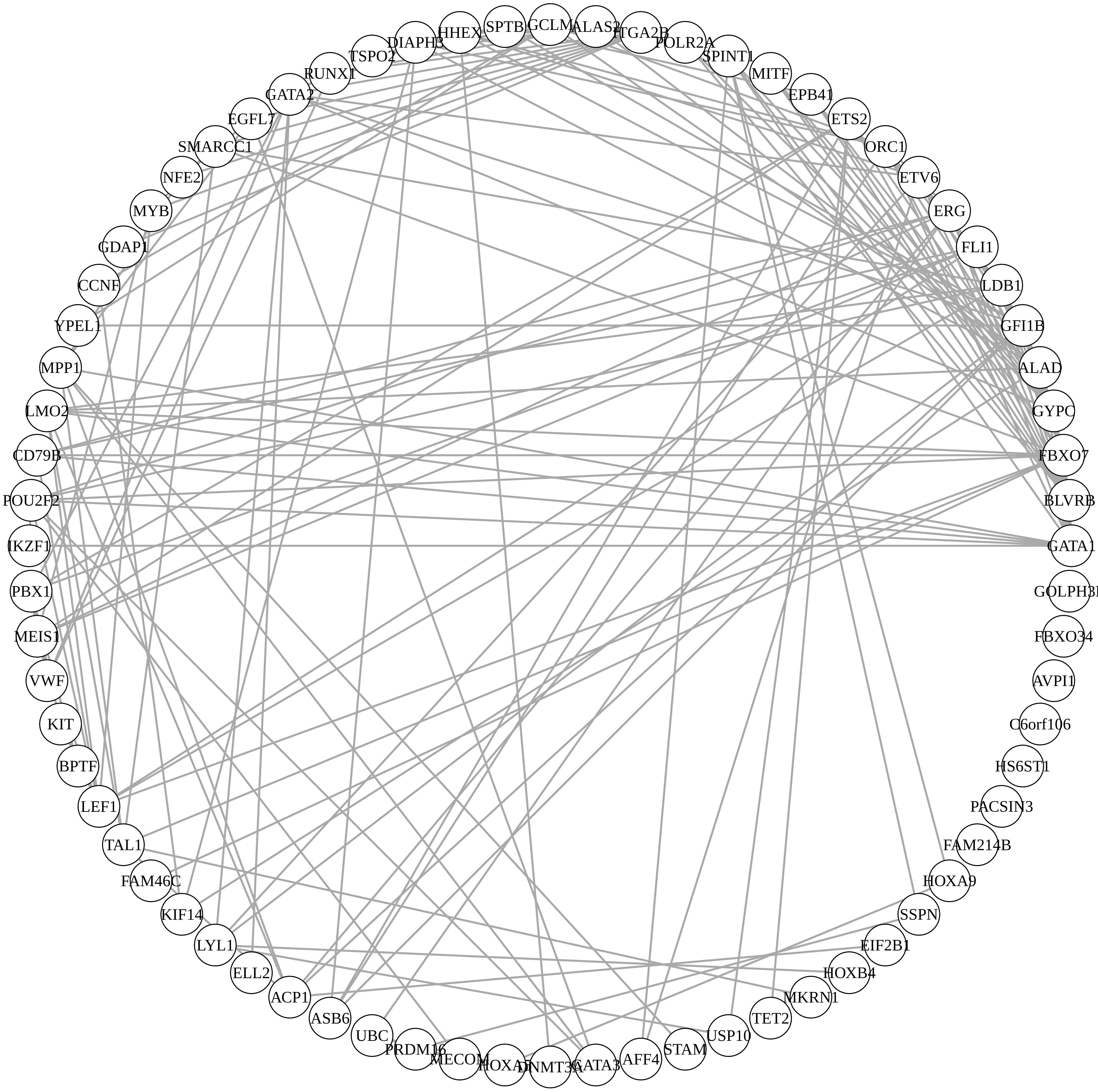

human_HSCtoMEP_time 2

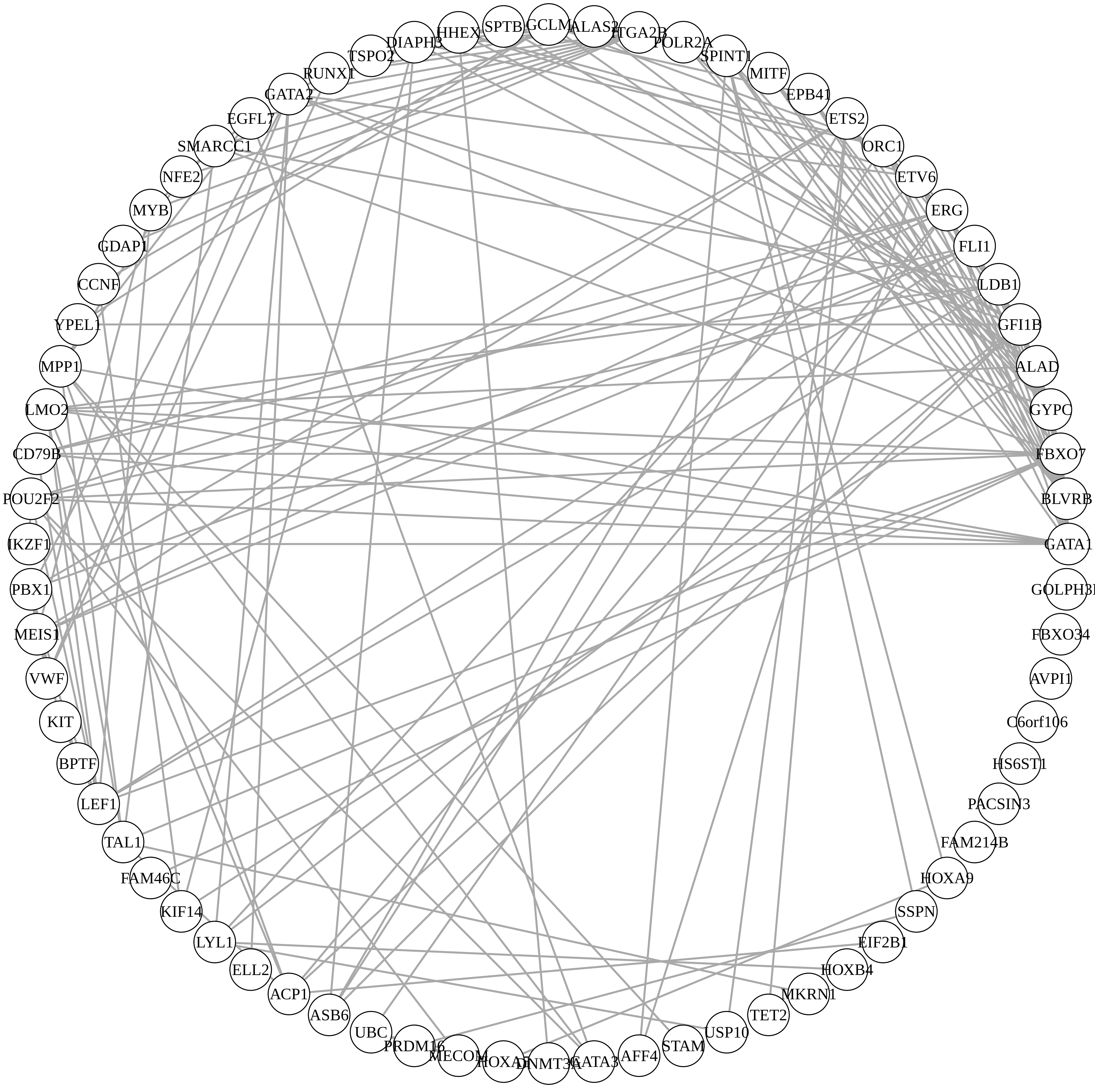

human_HSCtoMEP_time 3

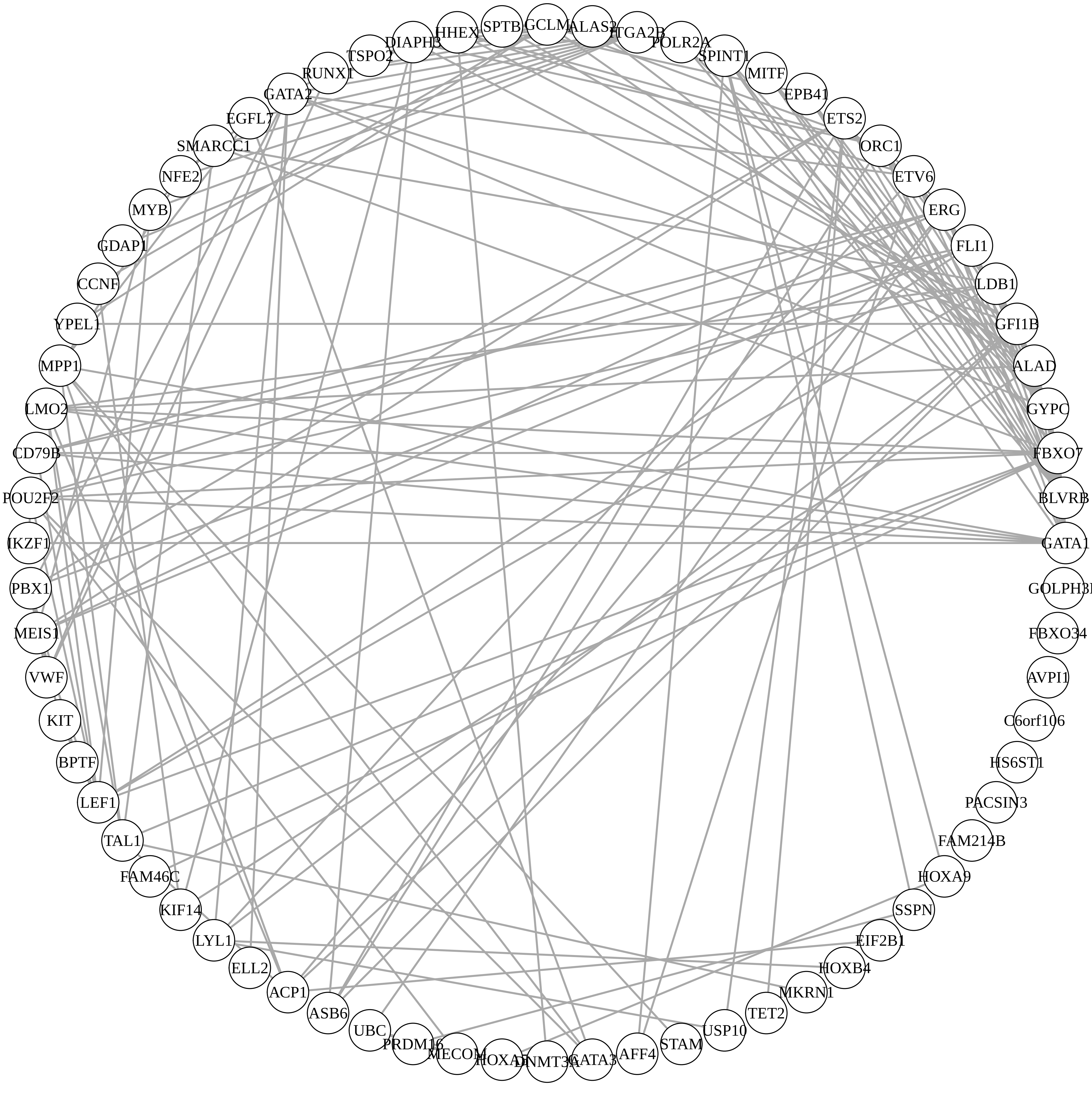

human_HSCtoMEP_time 4

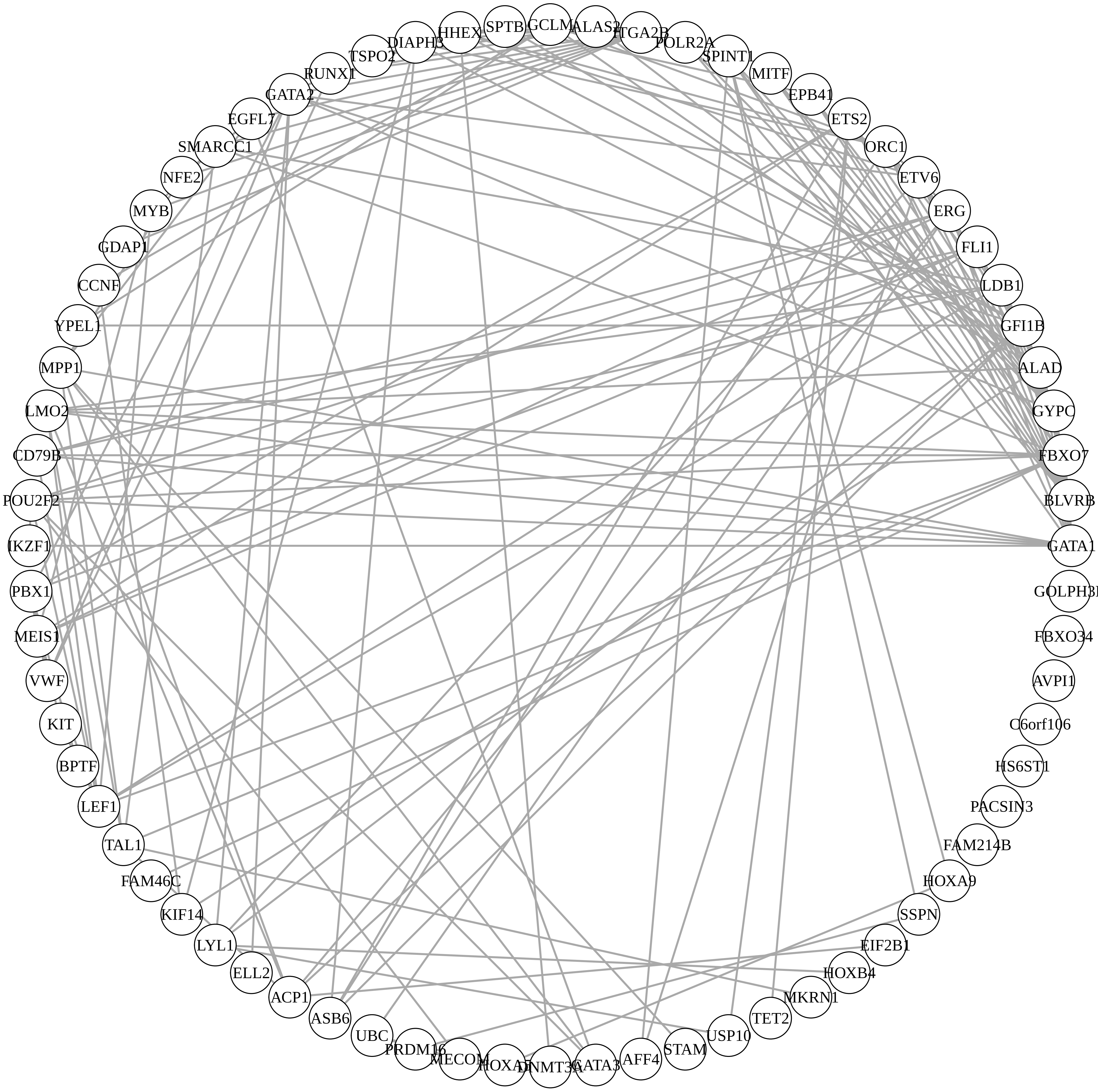

human_HSCtoMEP_time 5

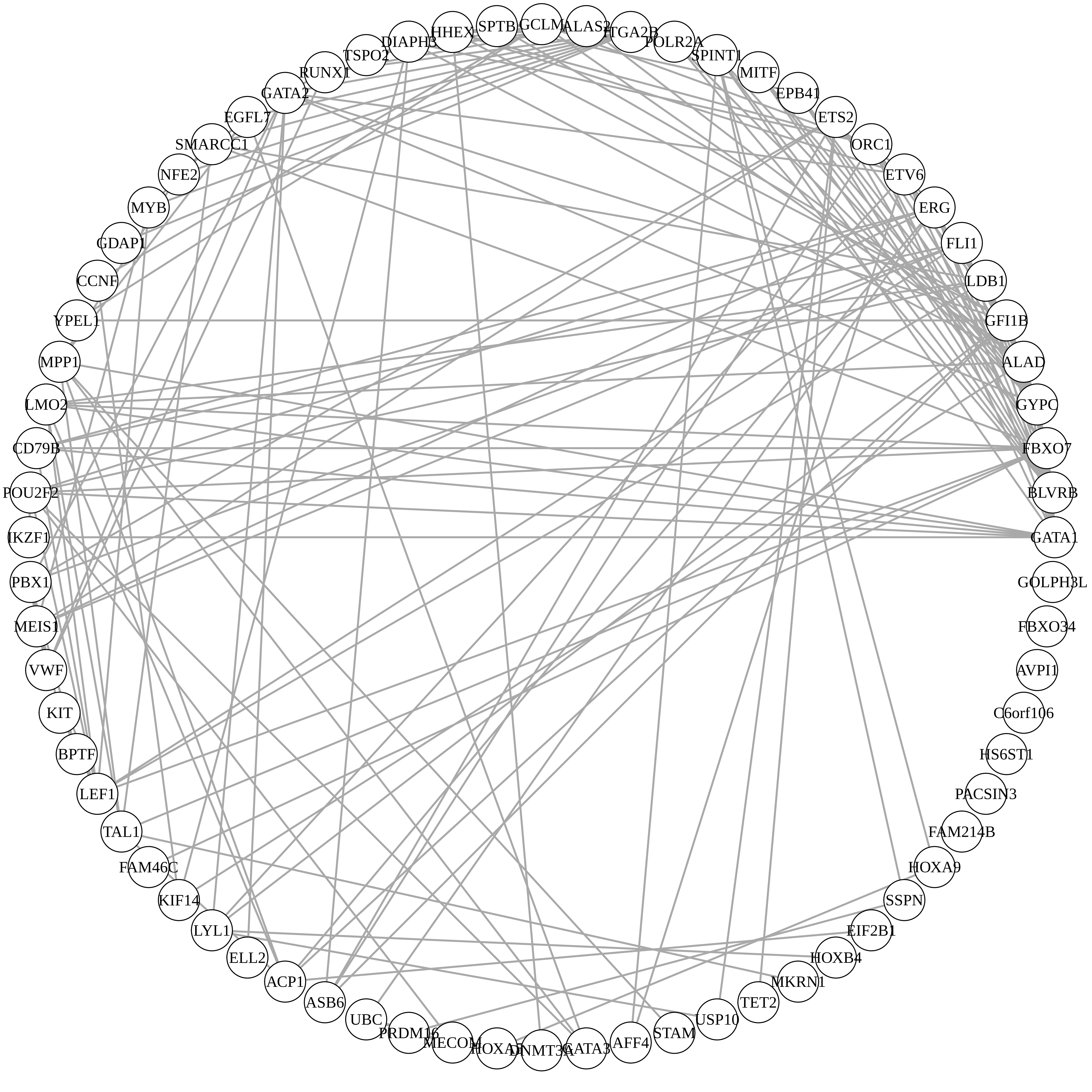

human_HSCtoMEP_time 6

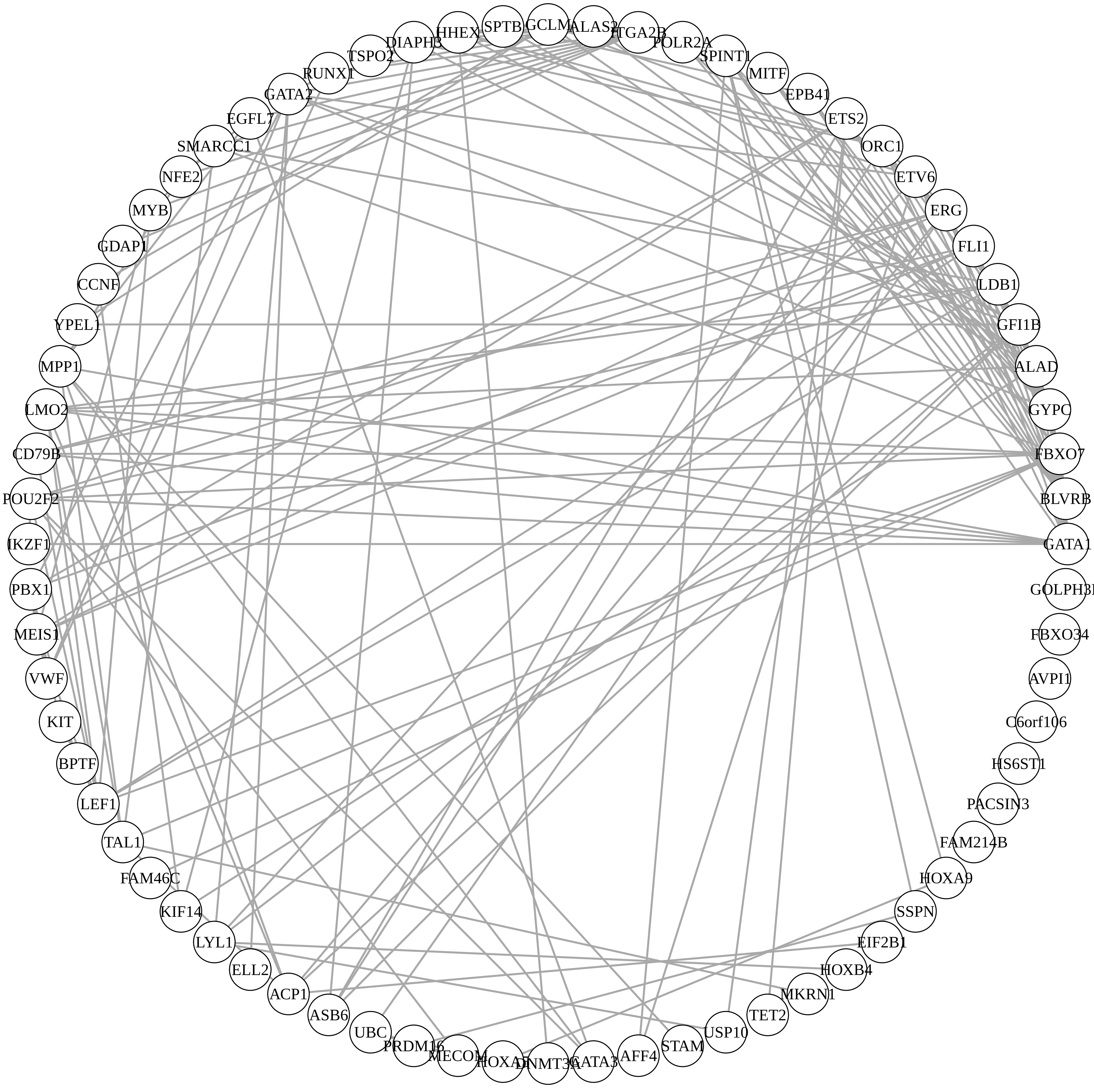

human_HSCtoMEP_time 7

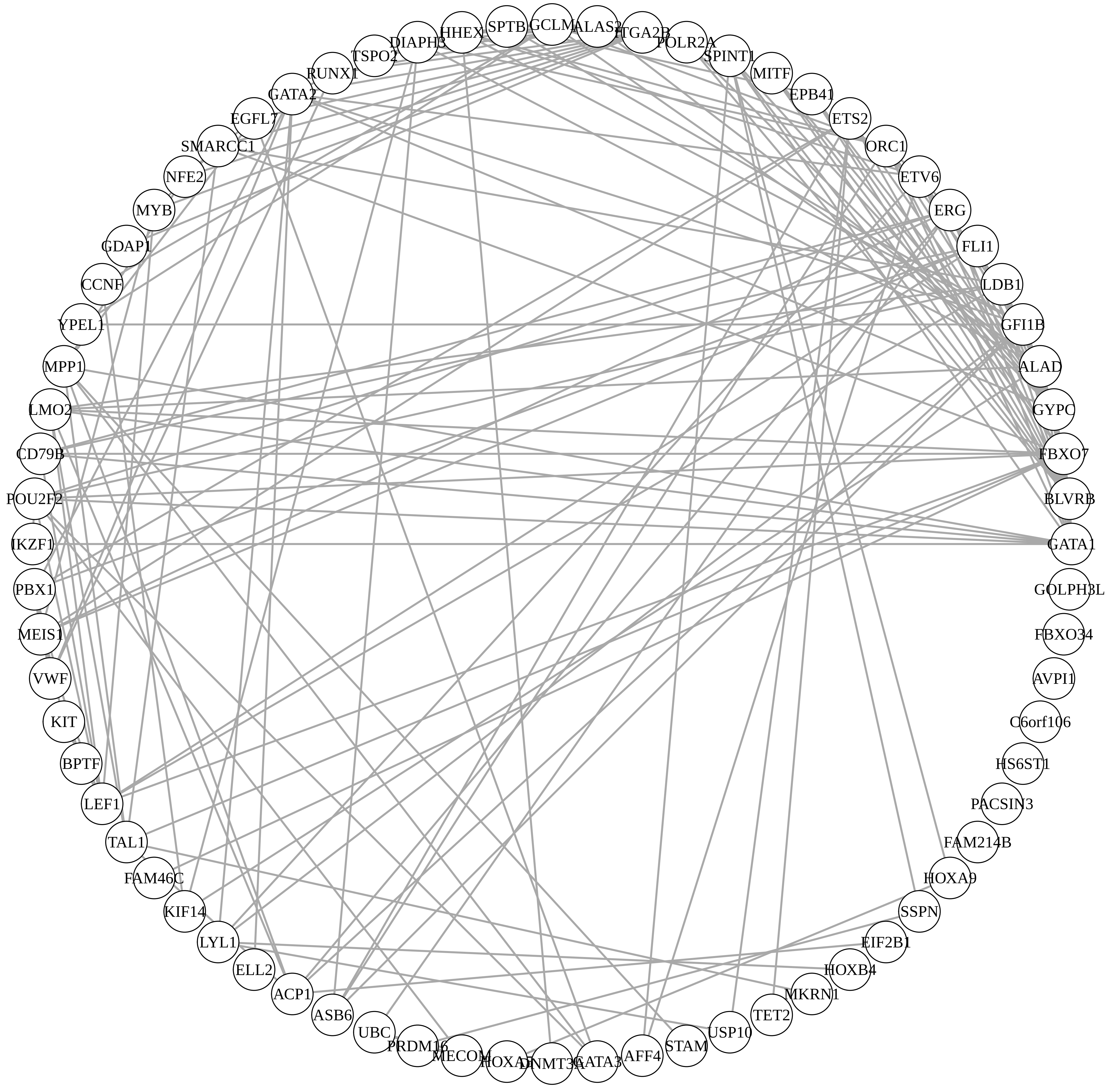

human_HSCtoMEP_time 8

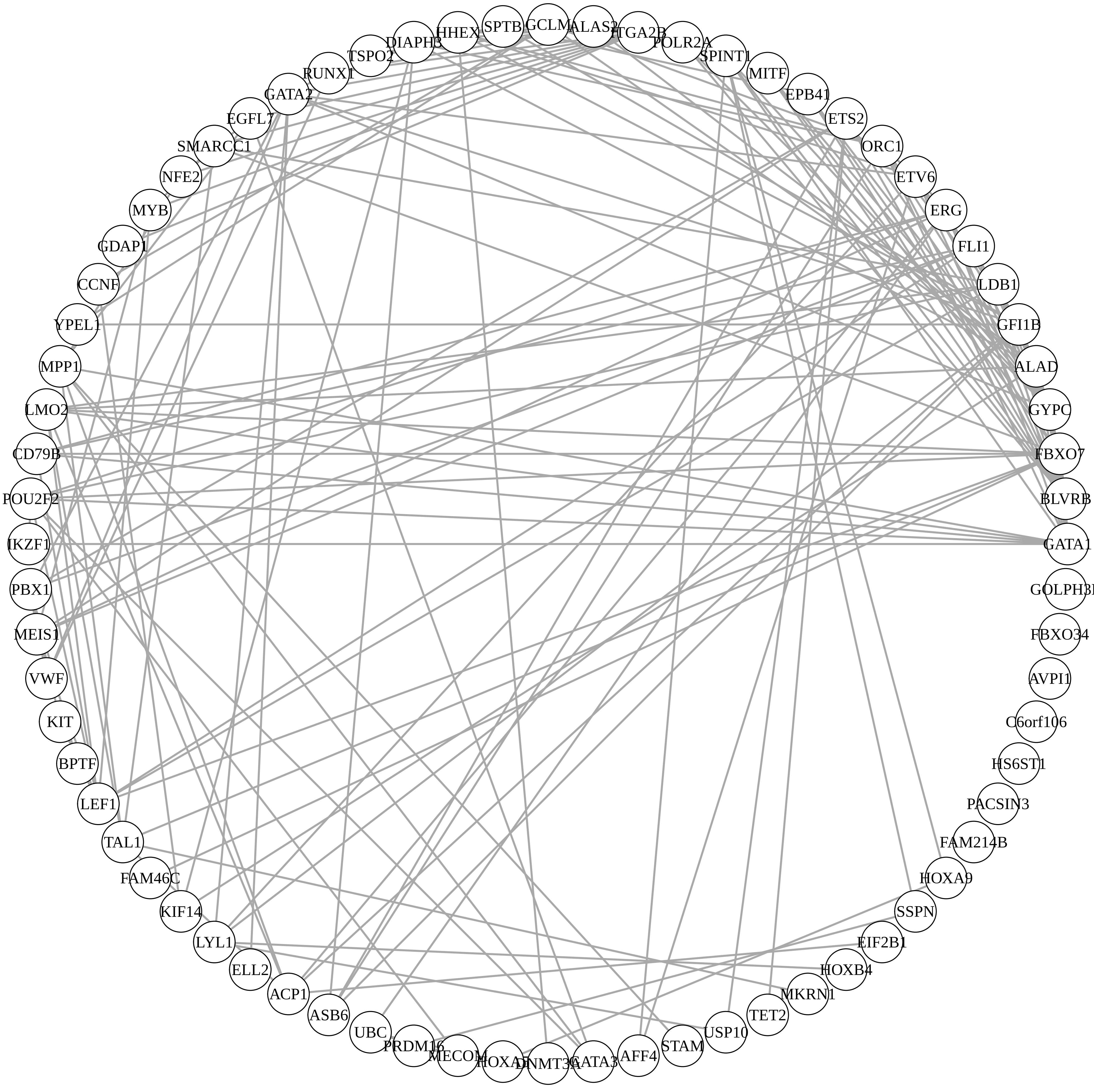

human_HSCtoMEP_time 9

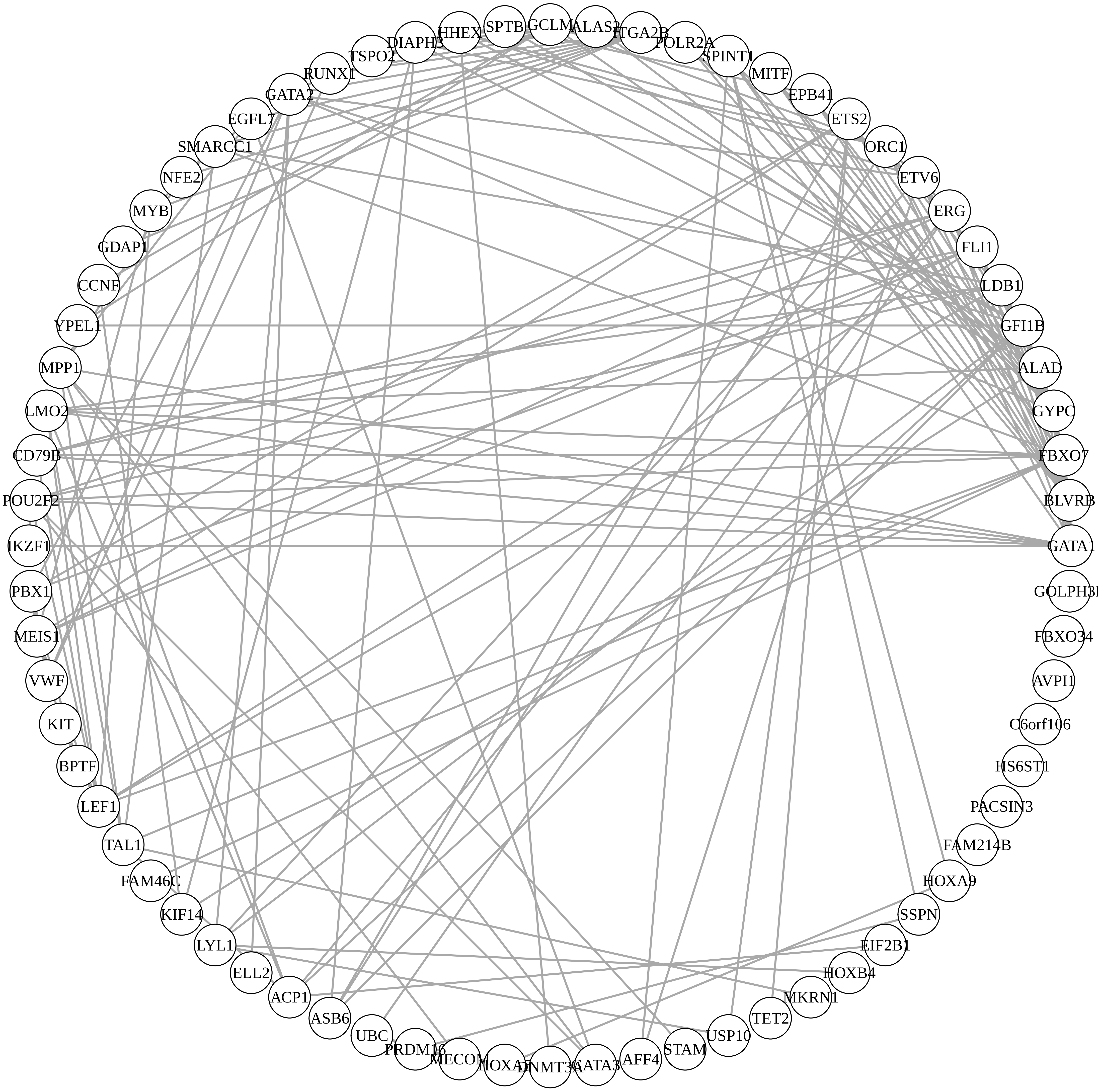

human_HSCtoMEP_time 10

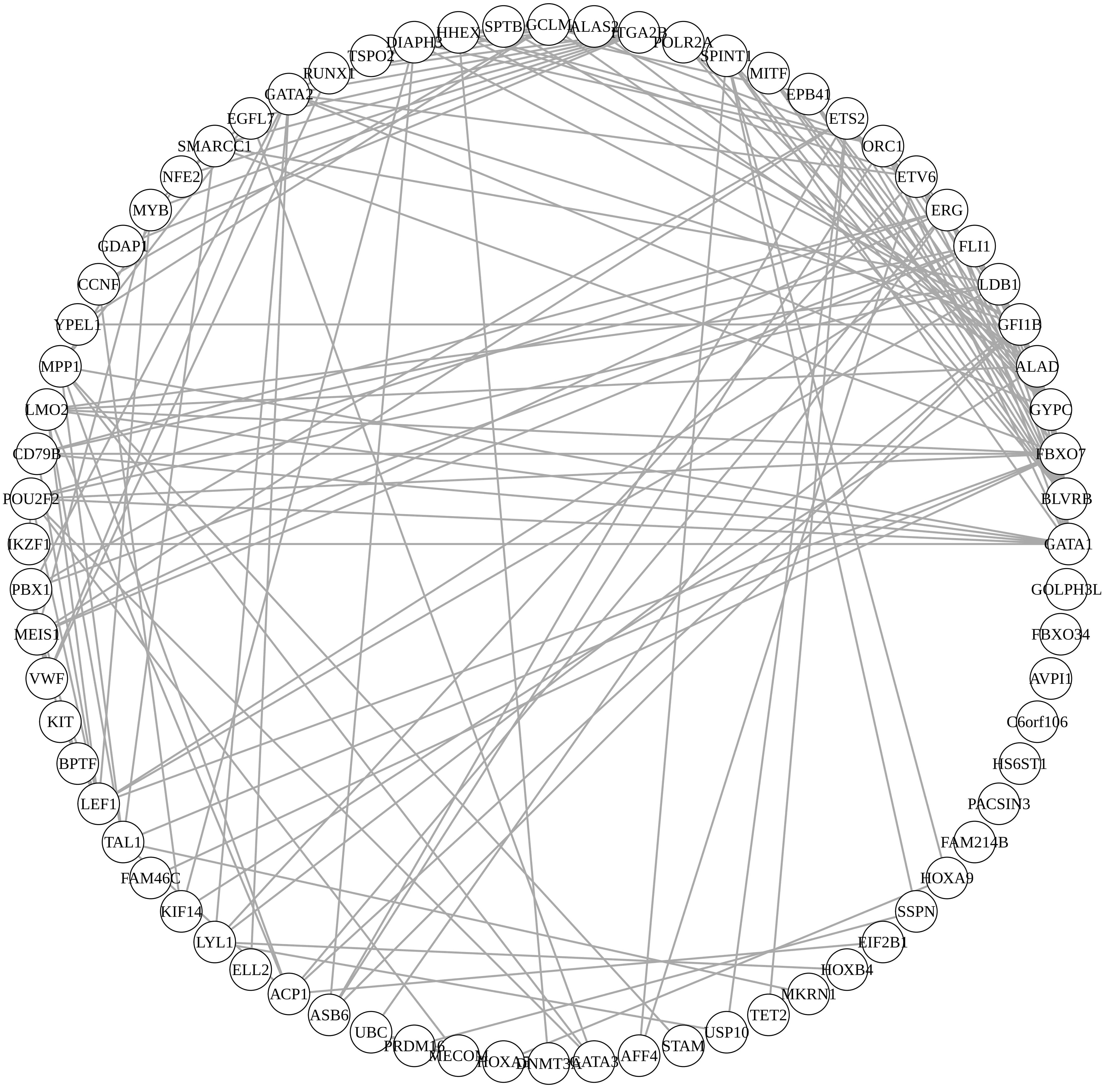

human_HSCtoMEP_time 11

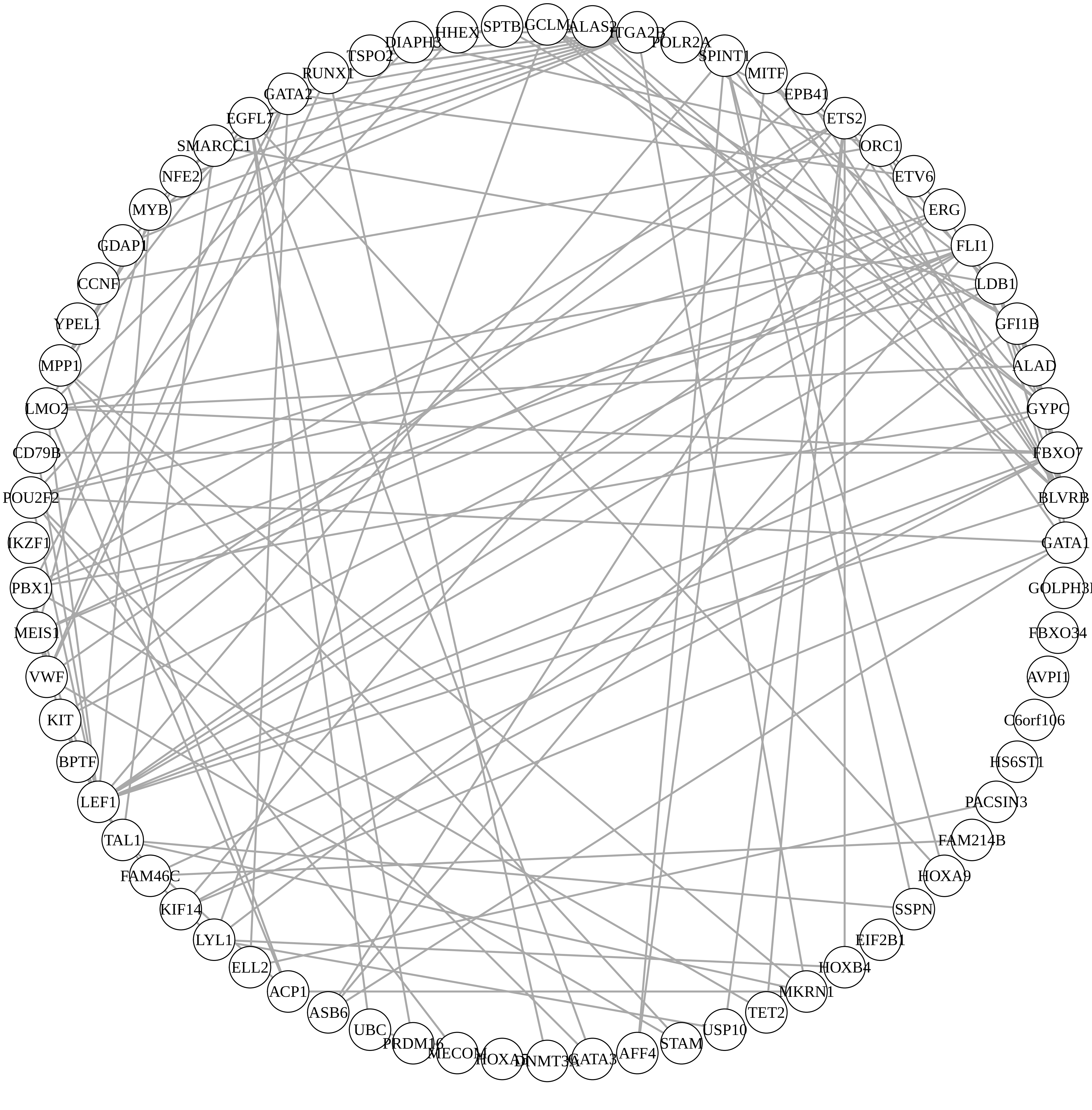

human_HSCtoMEP_time 12

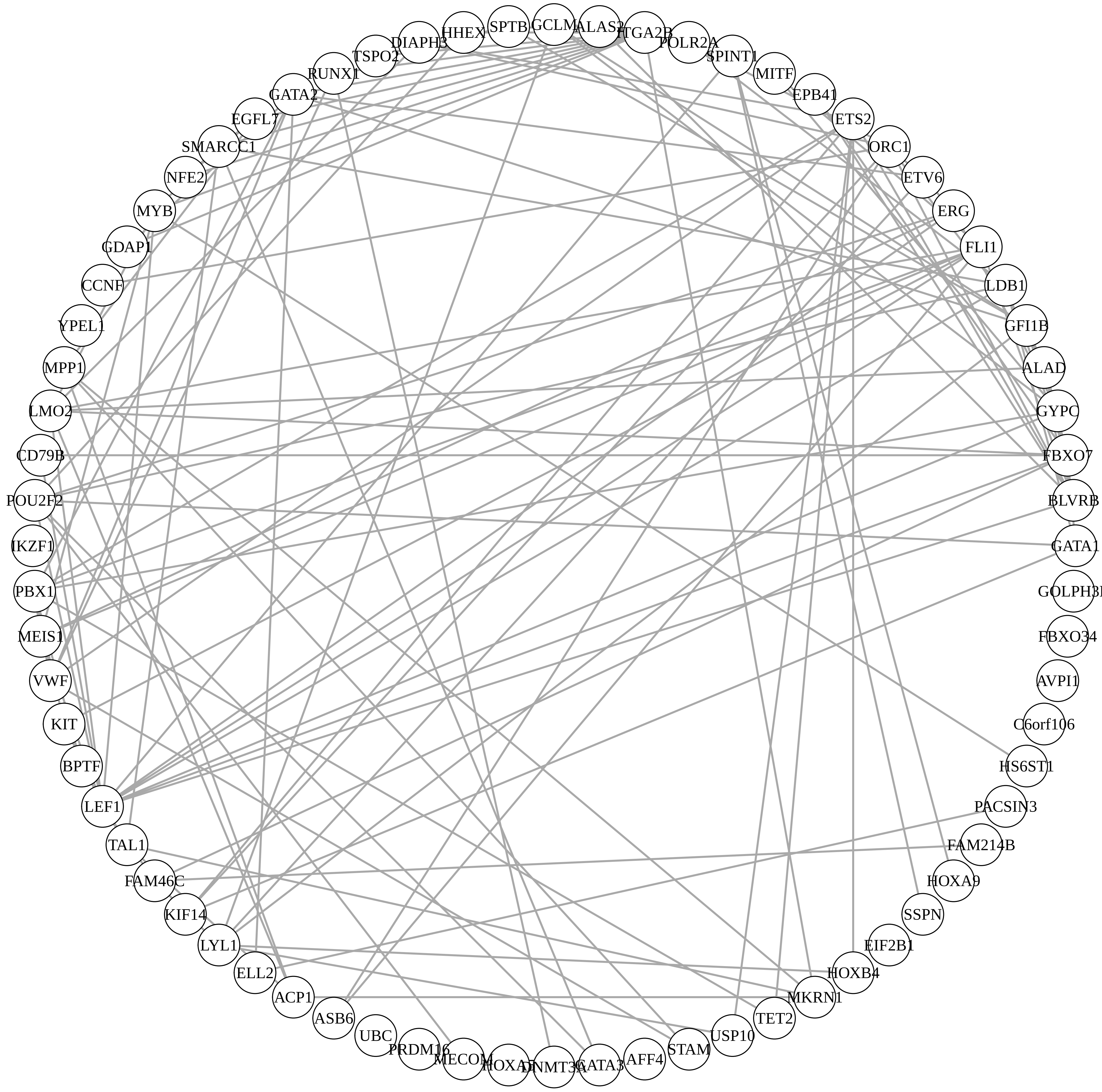

human_HSCtoMEP_time 13

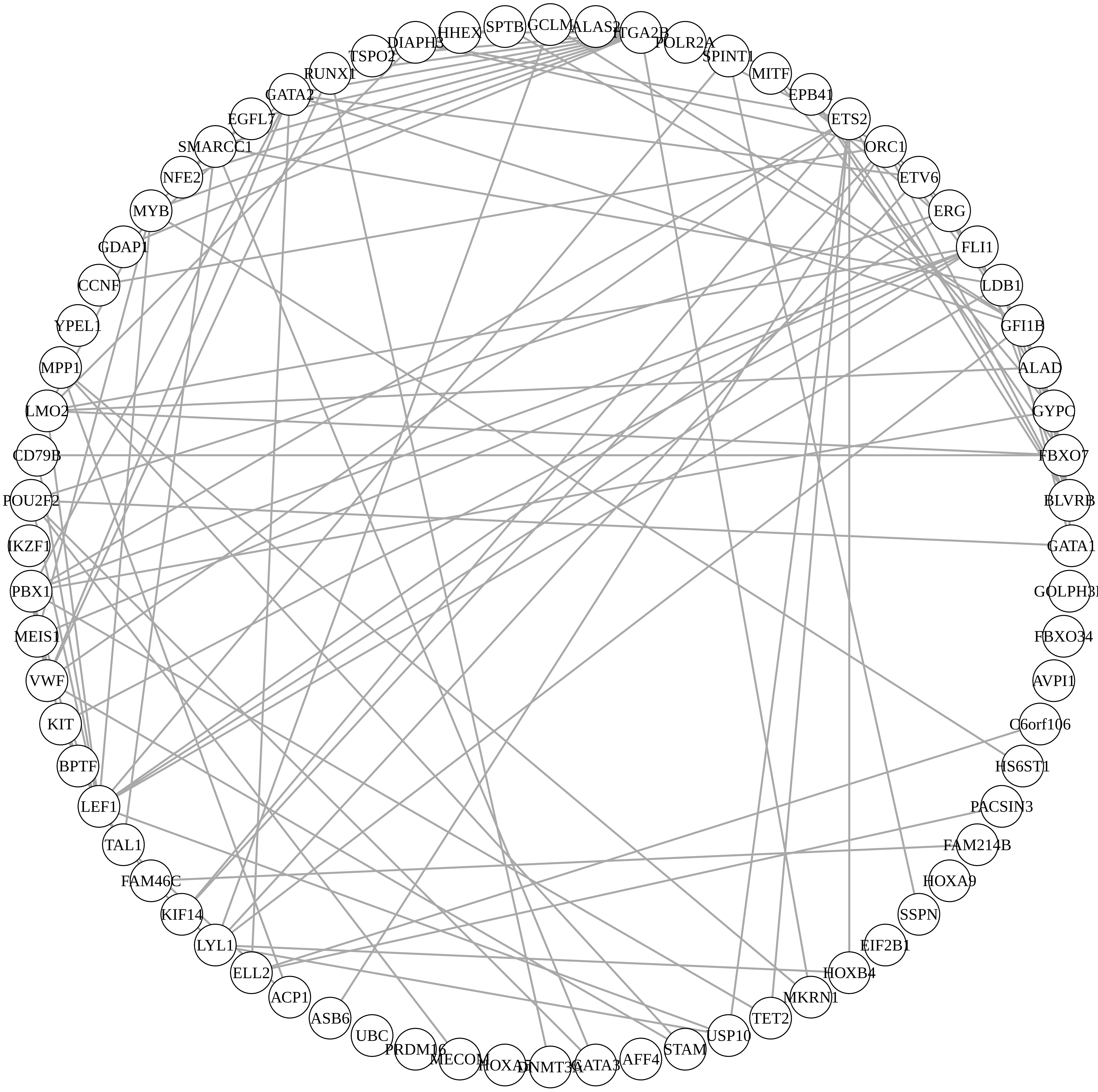

human_HSCtoMEP_time 14

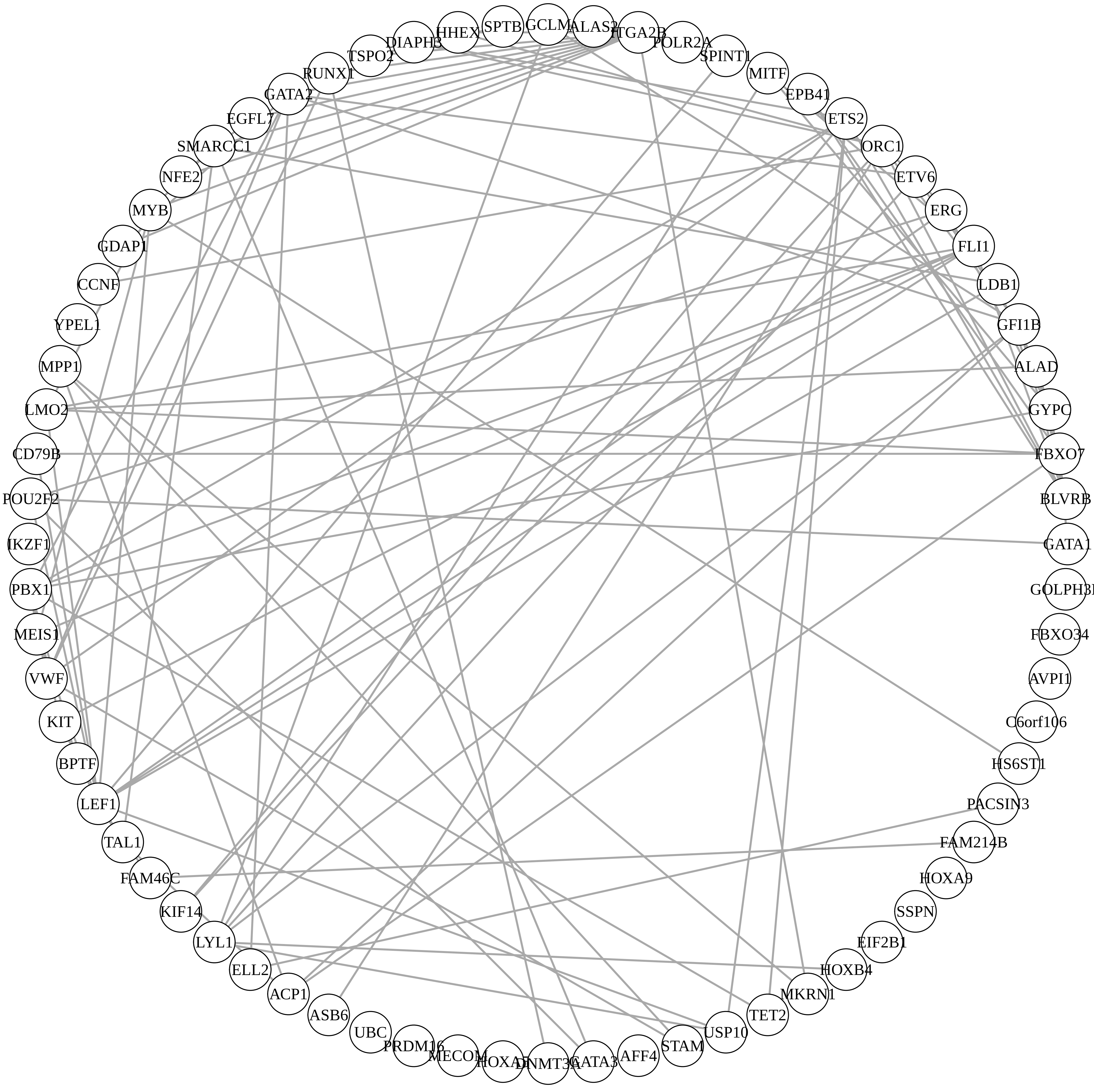

human_HSCtoMEP_time 15

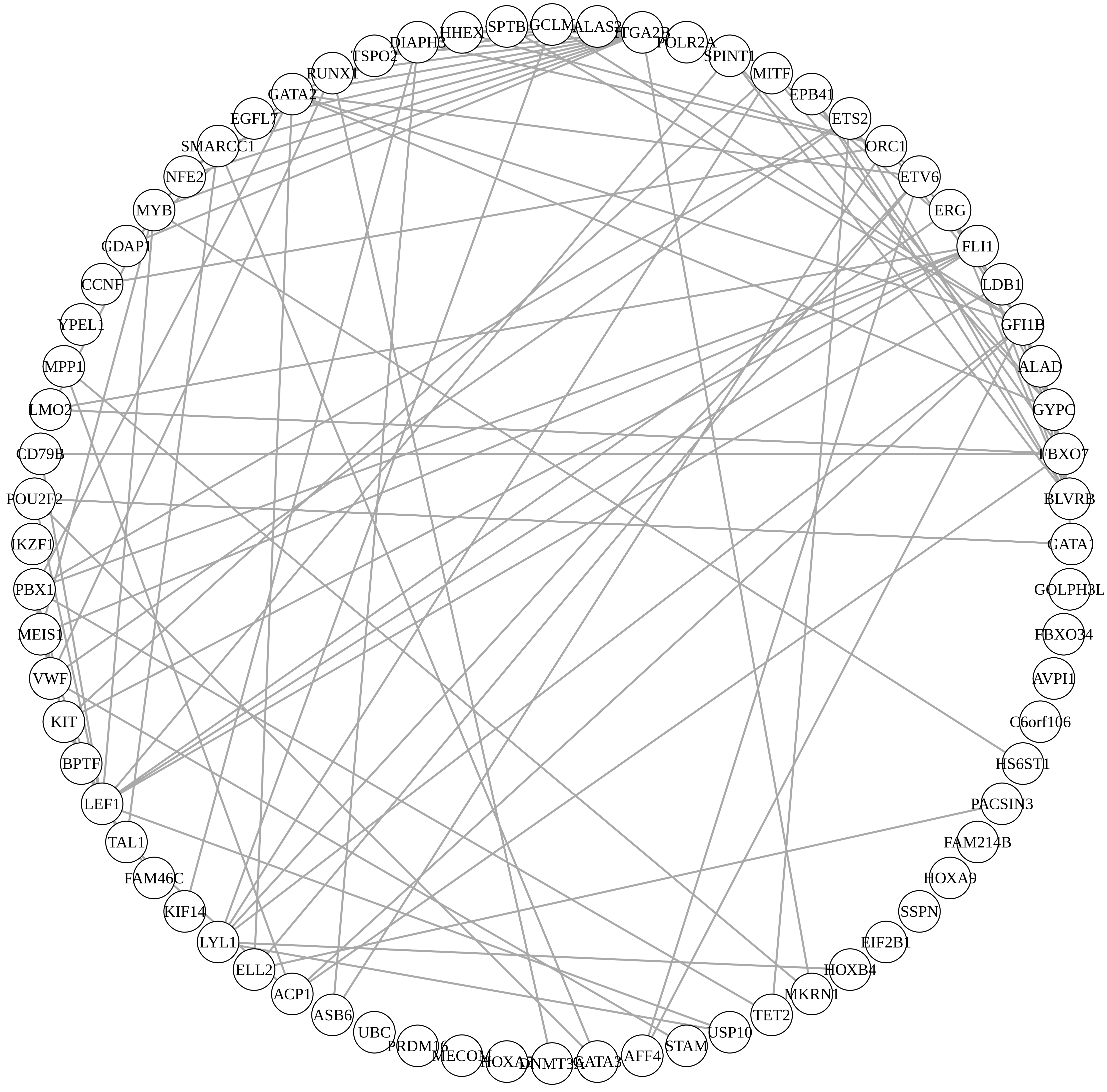

human_HSCtoMEP_time 16

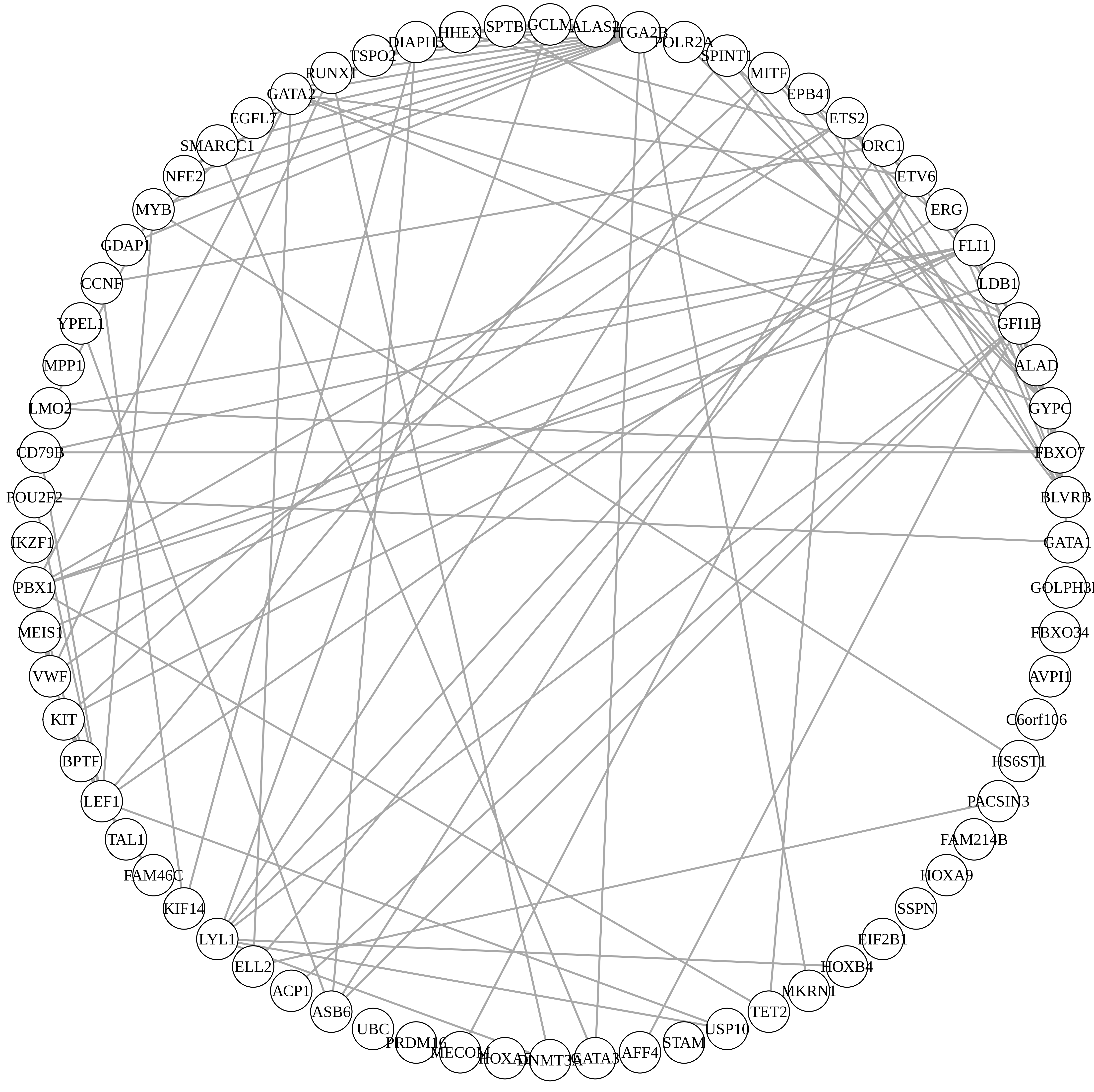

human_HSCtoMEP_time 17

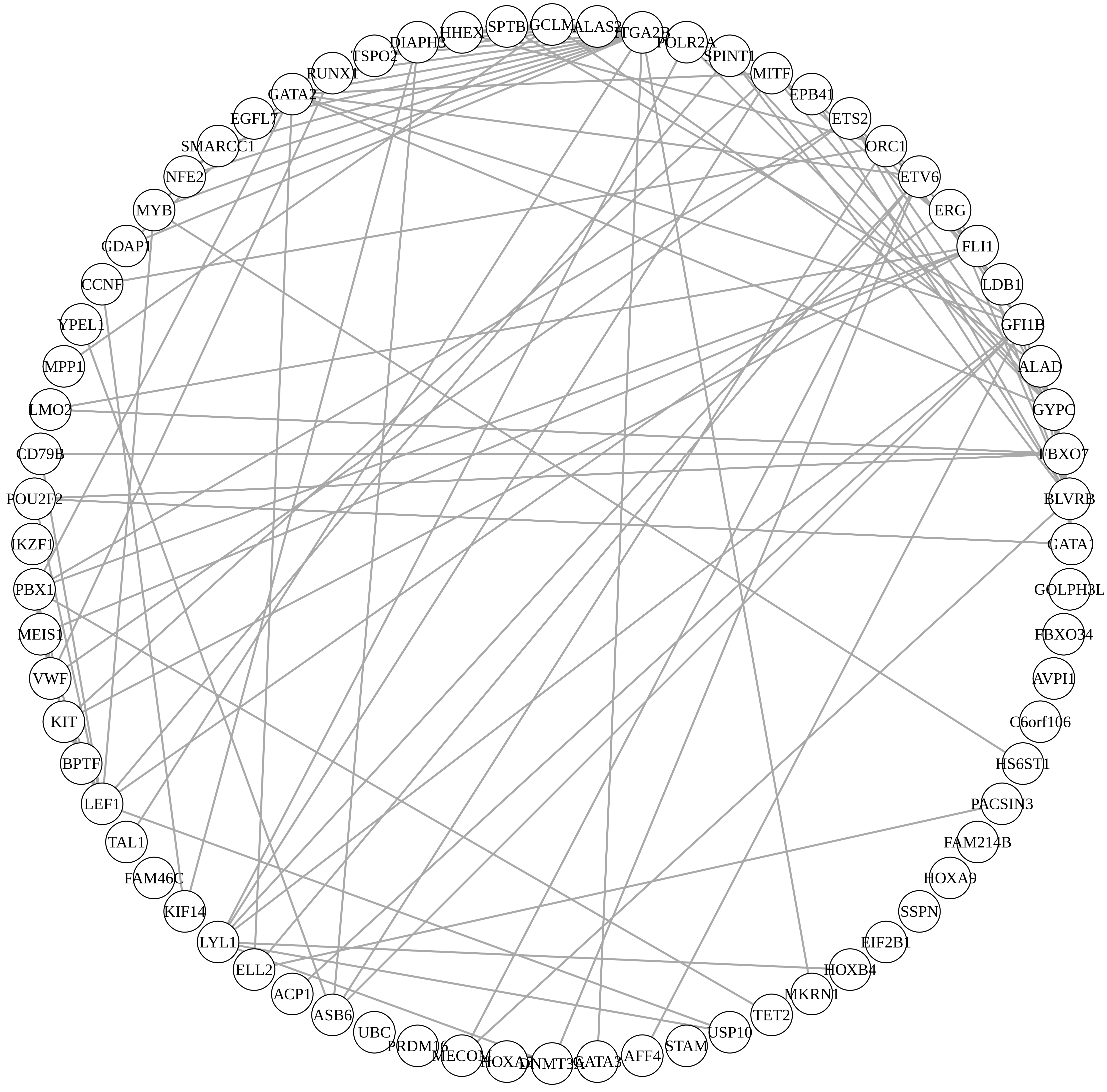

human_HSCtoMEP_time 18

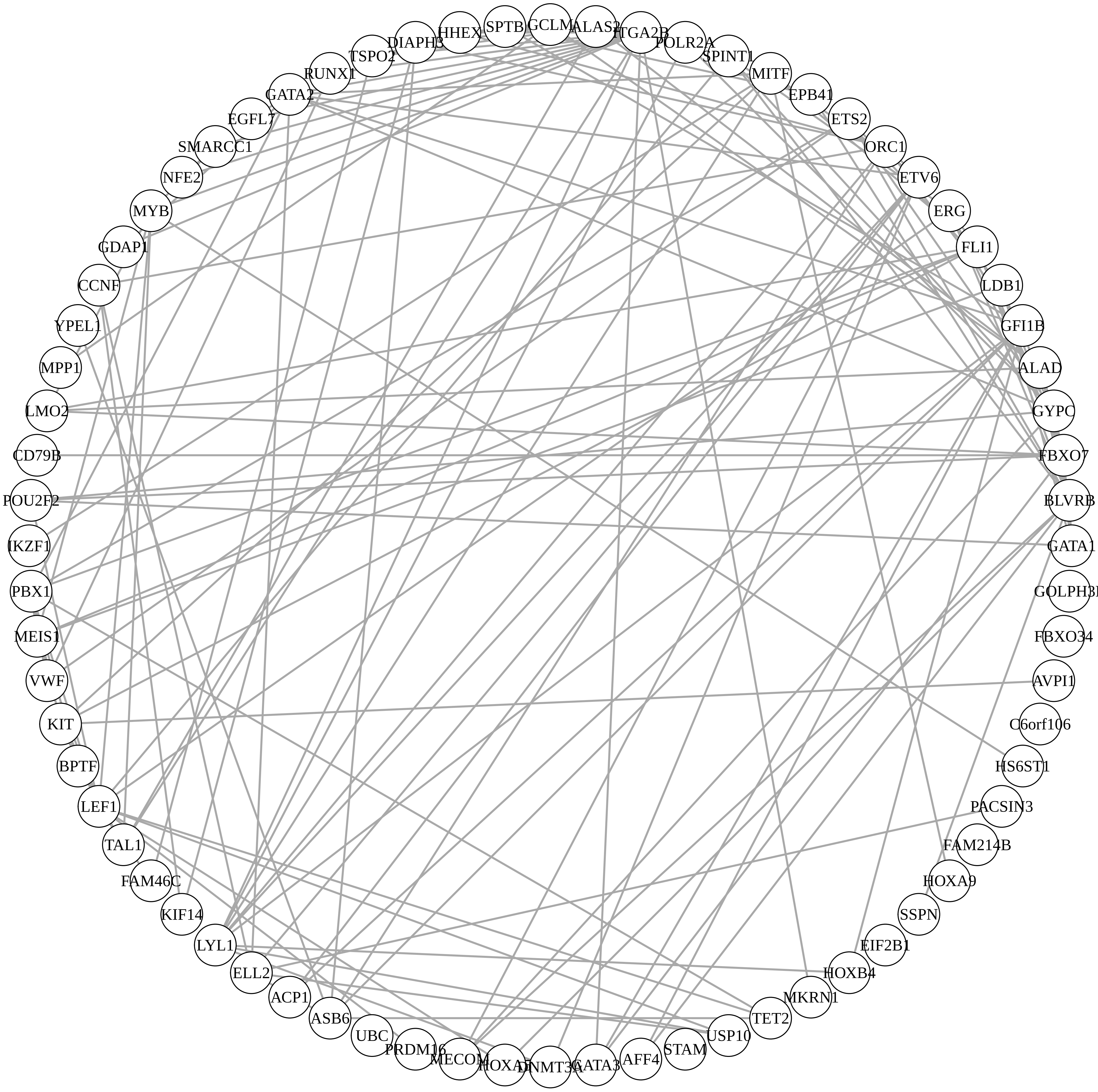

human_HSCtoMEP_time 19

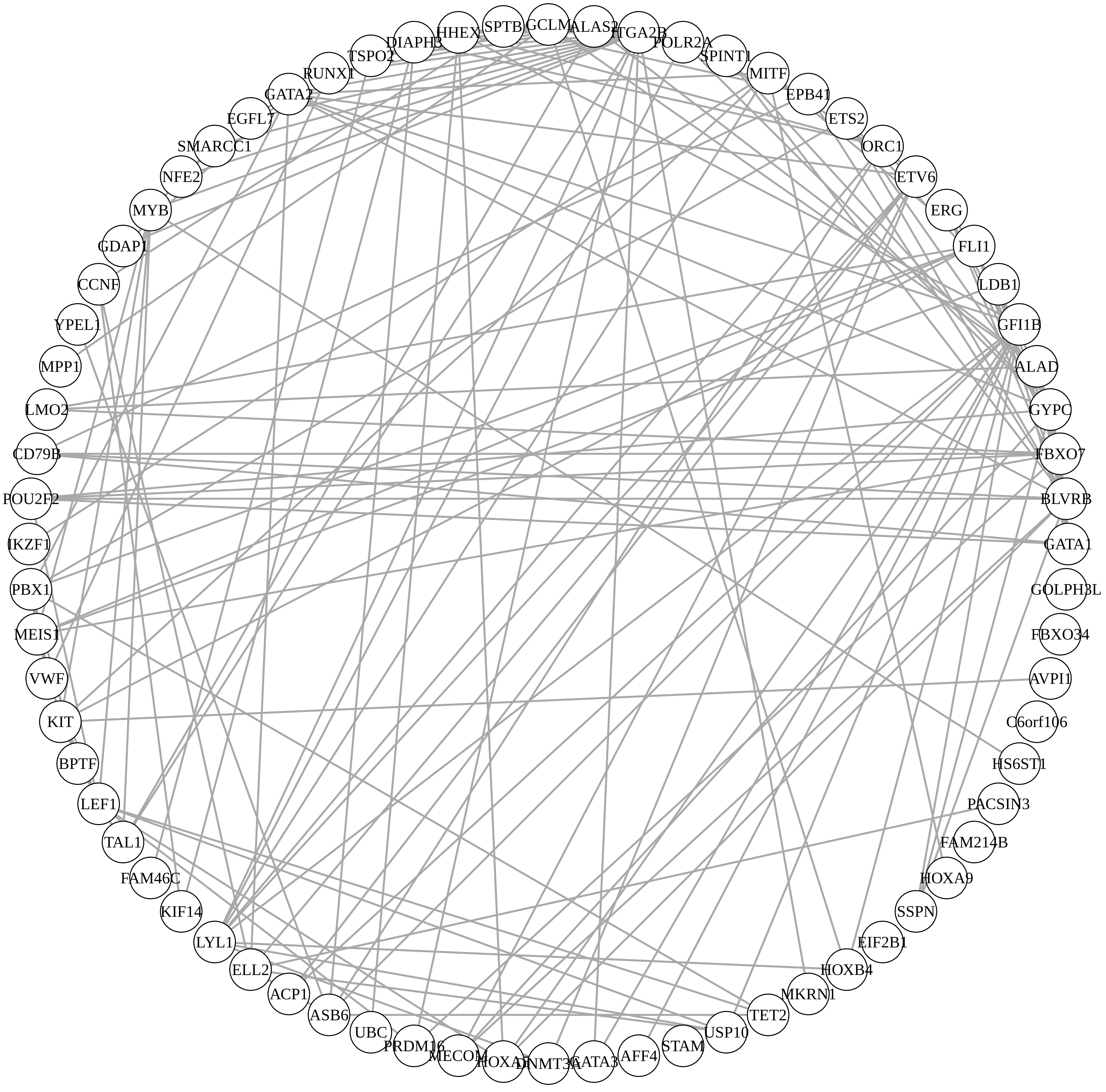

human_HSCtoMEP_time 20

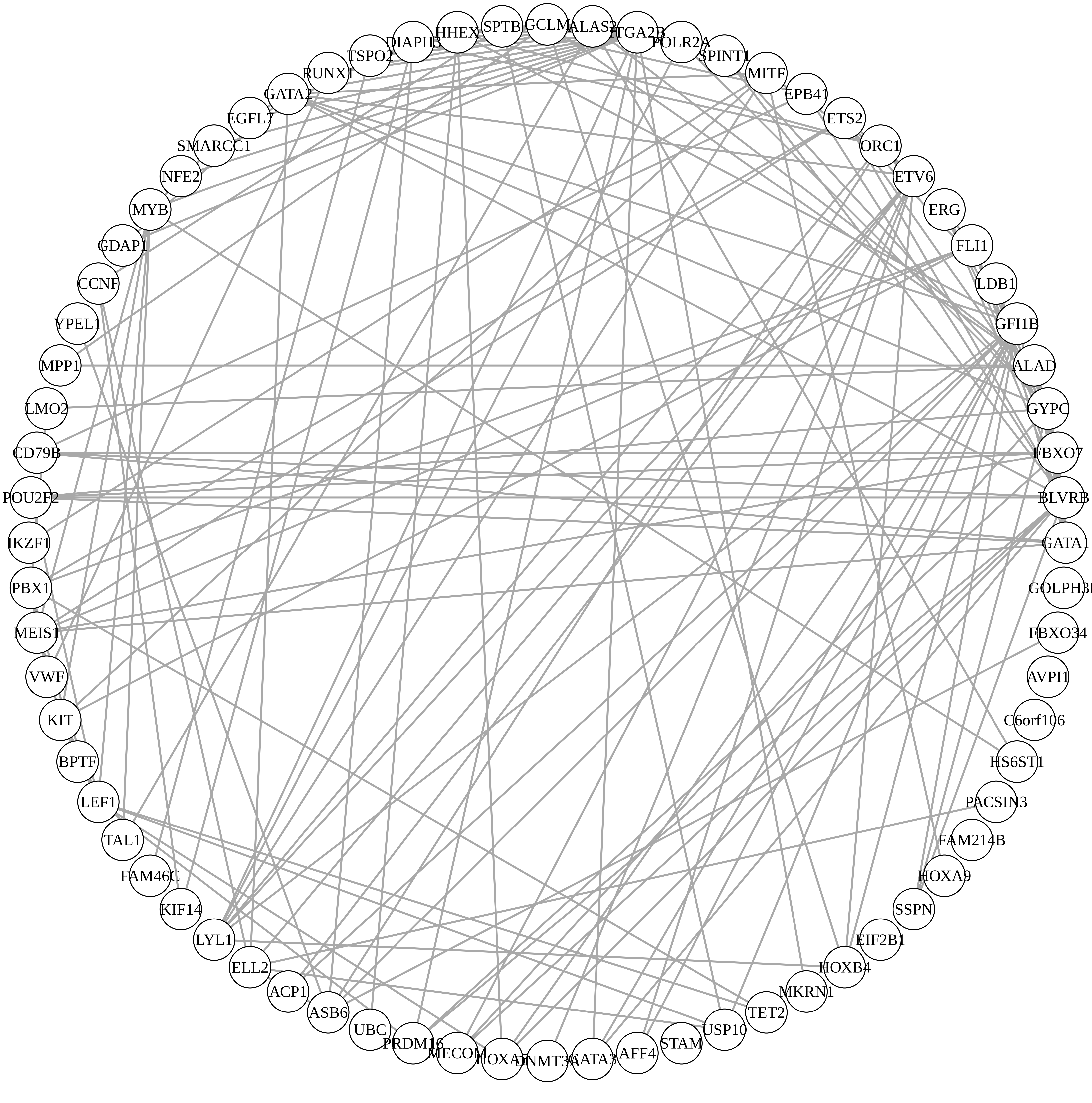

human_HSCtoMEP_time 21

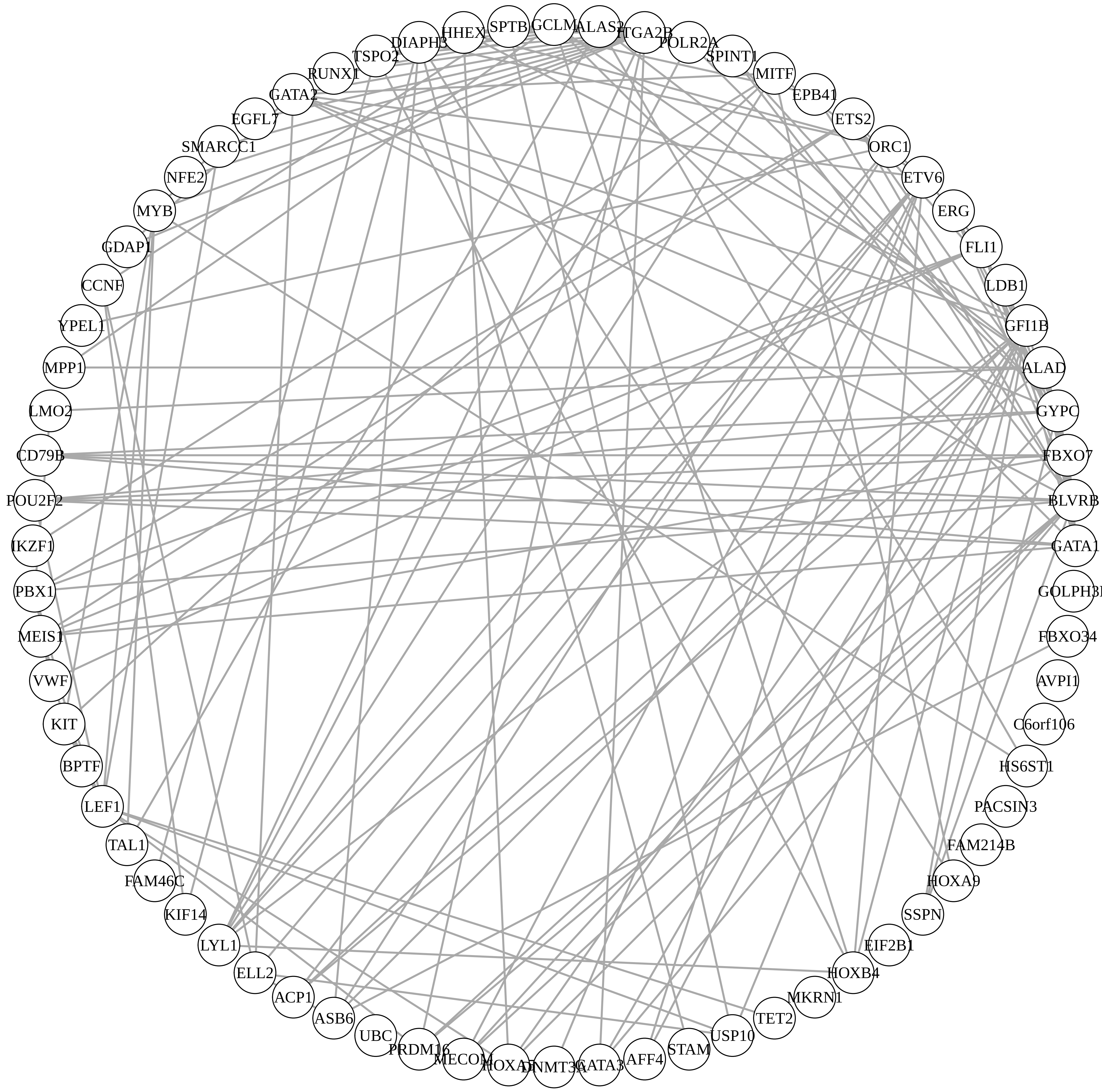

human_HSCtoMEP_time 22

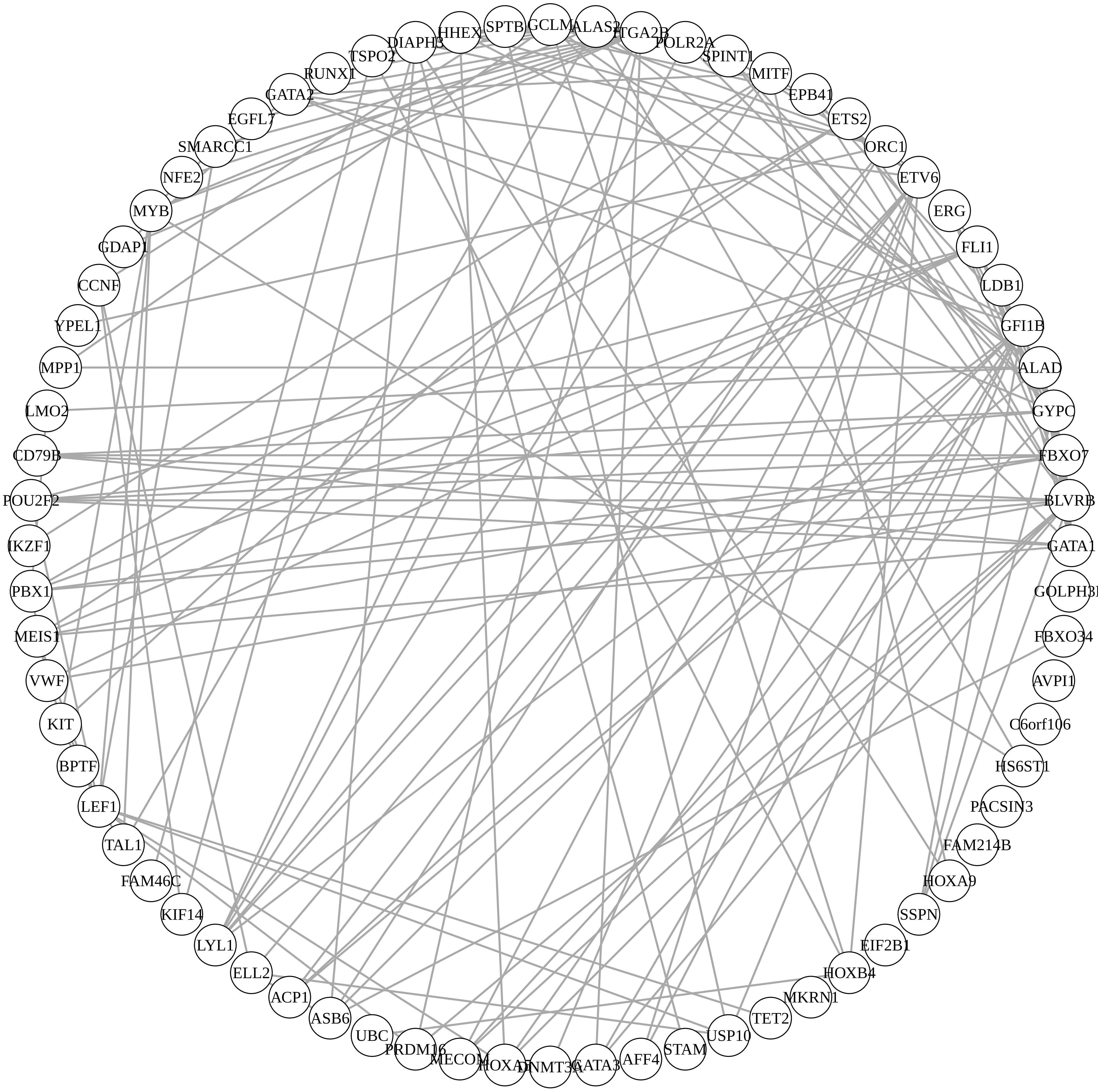

human_HSCtoMEP_time 23

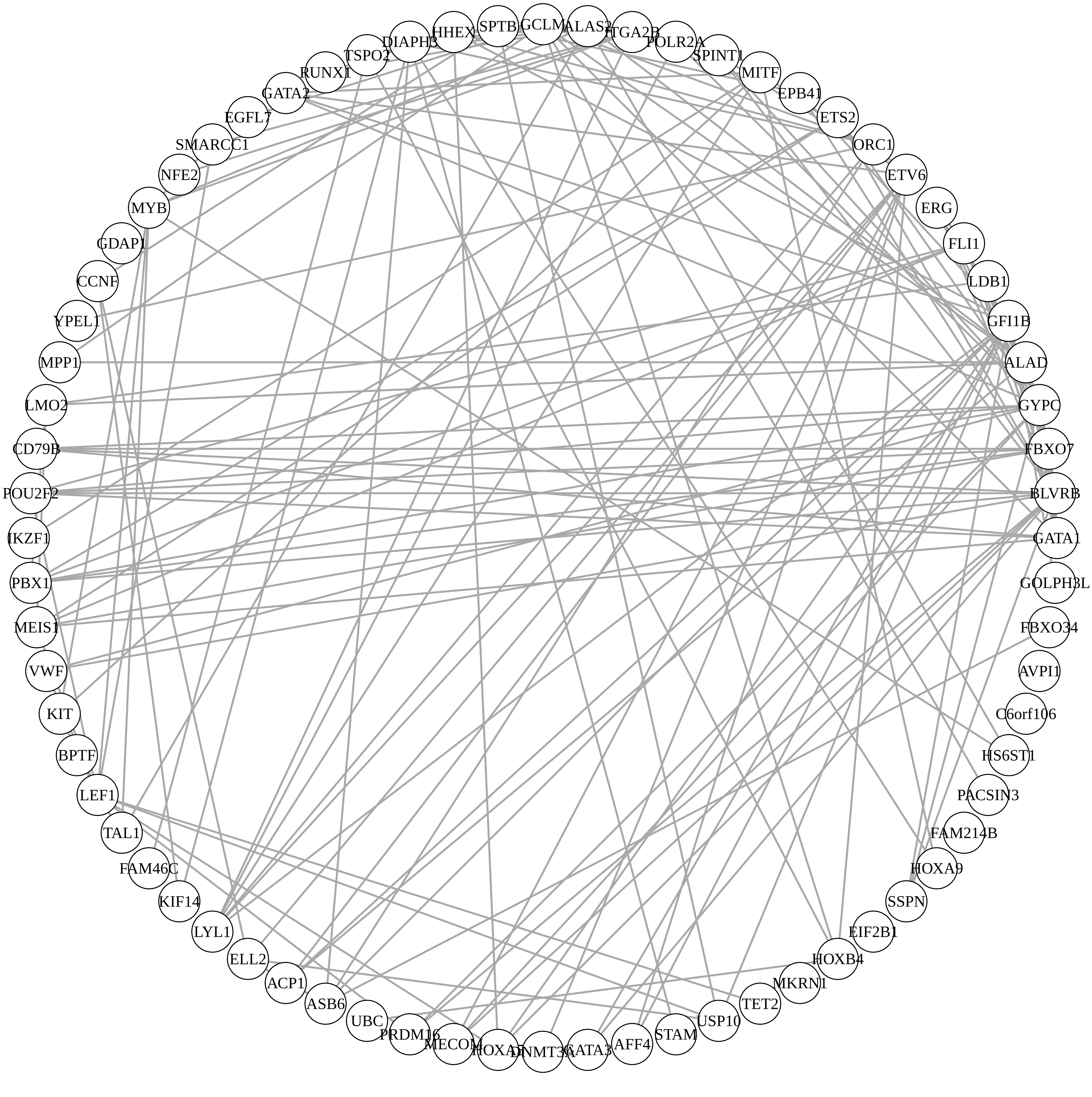

human_HSCtoMEP_time 24

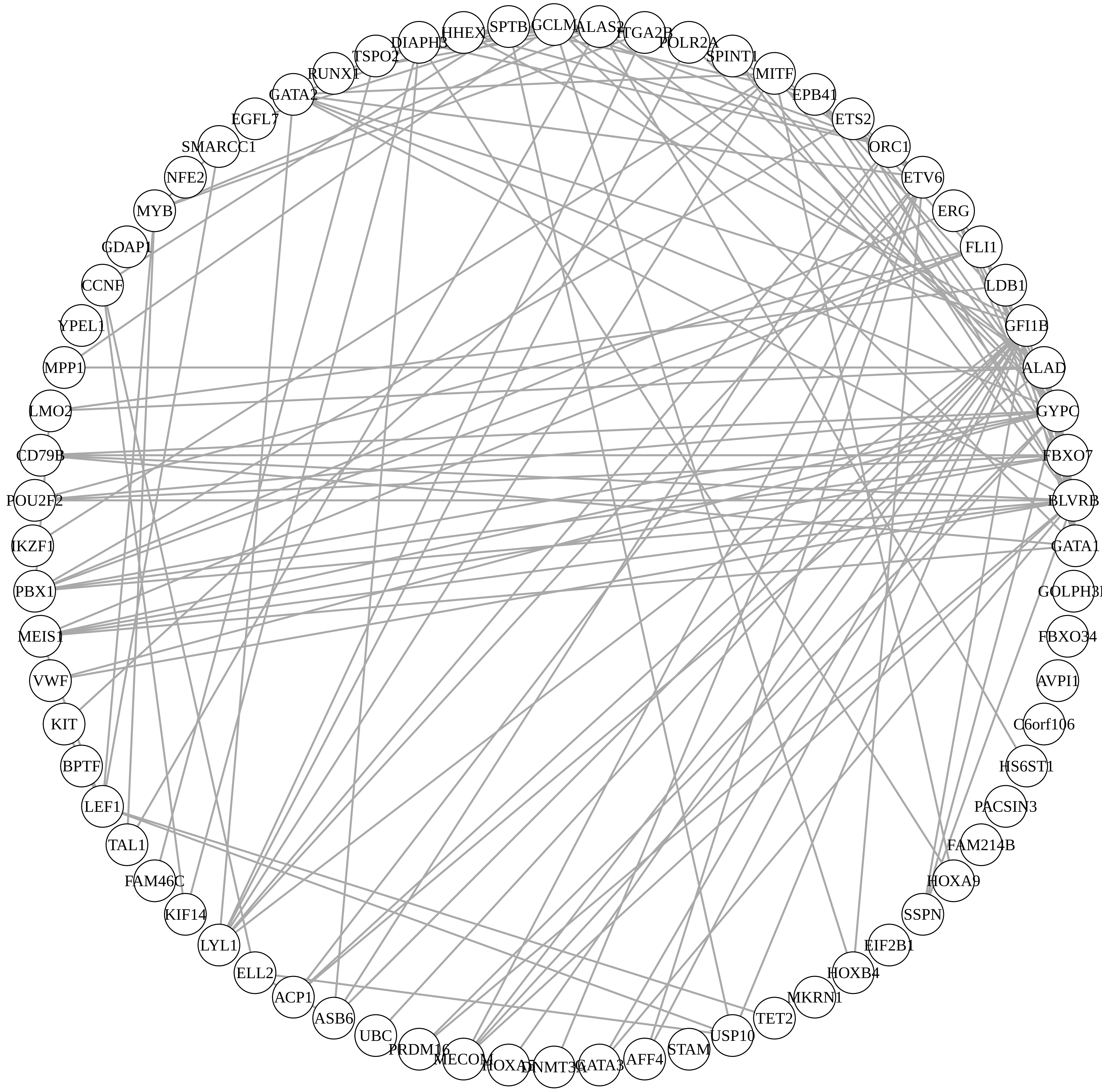

human_HSCtoMEP_time 25

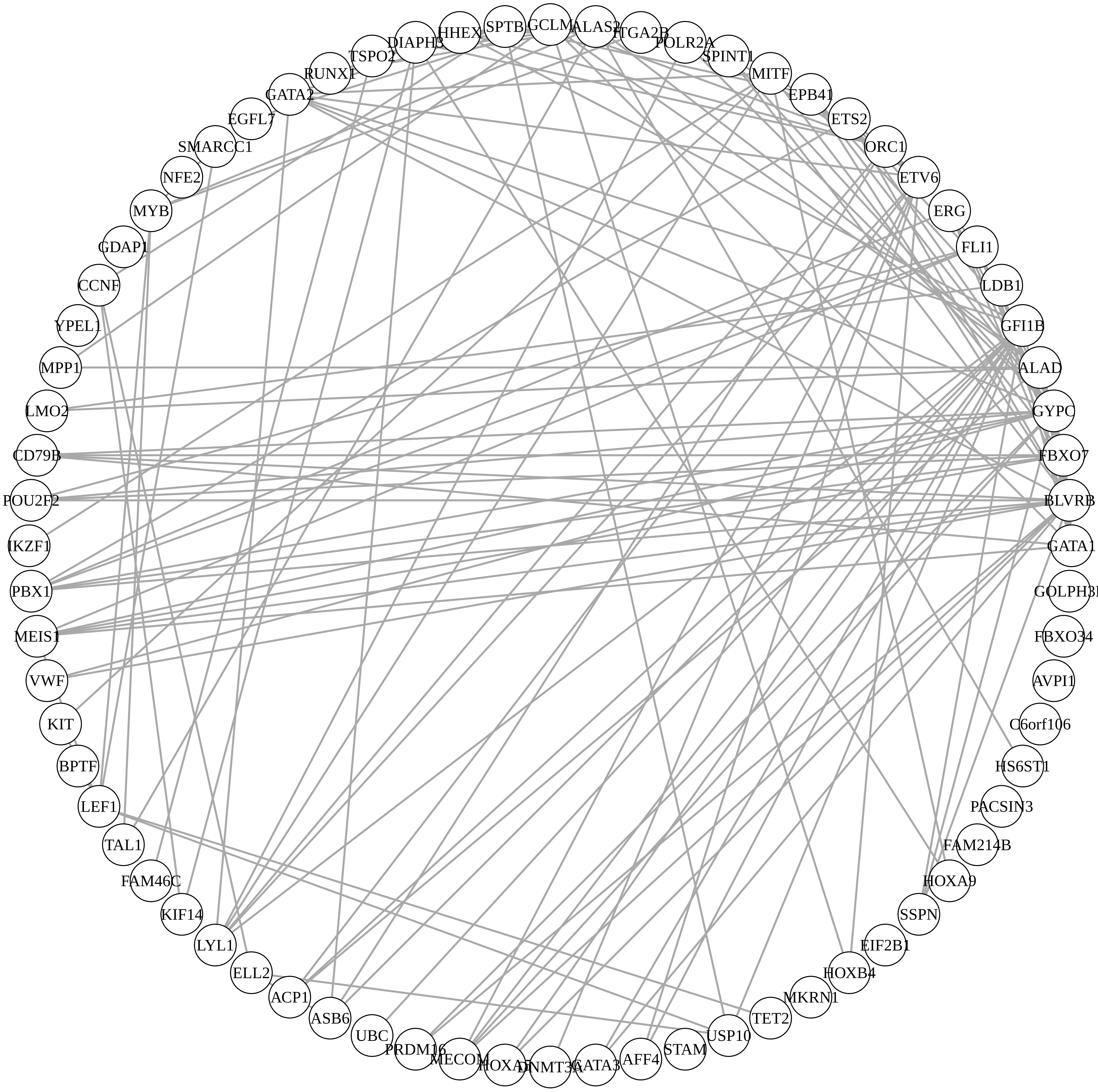

human_HSCtoGMP_time 1

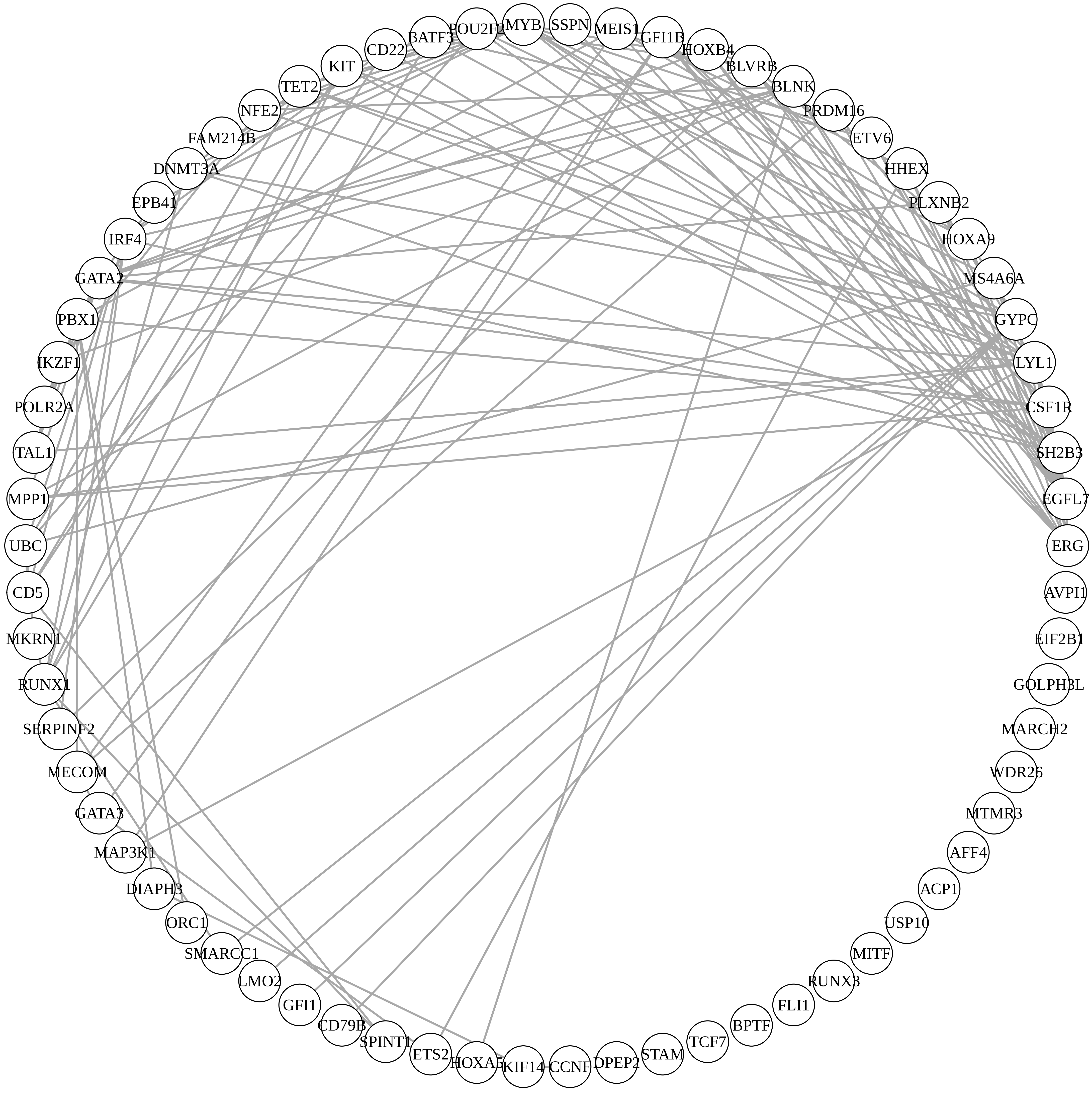

human_HSCtoGMP_time 2

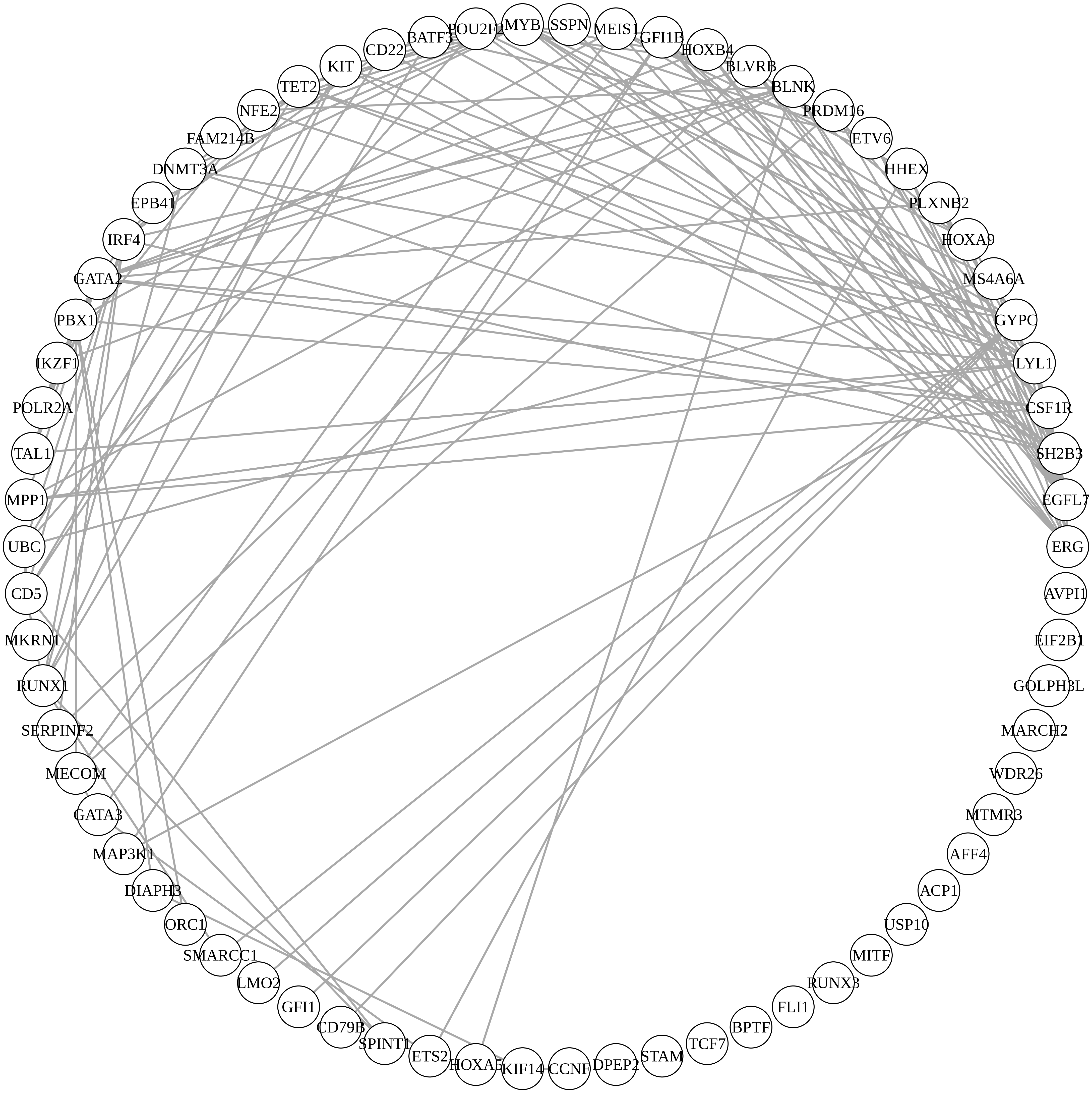

human_HSCtoGMP_time 3

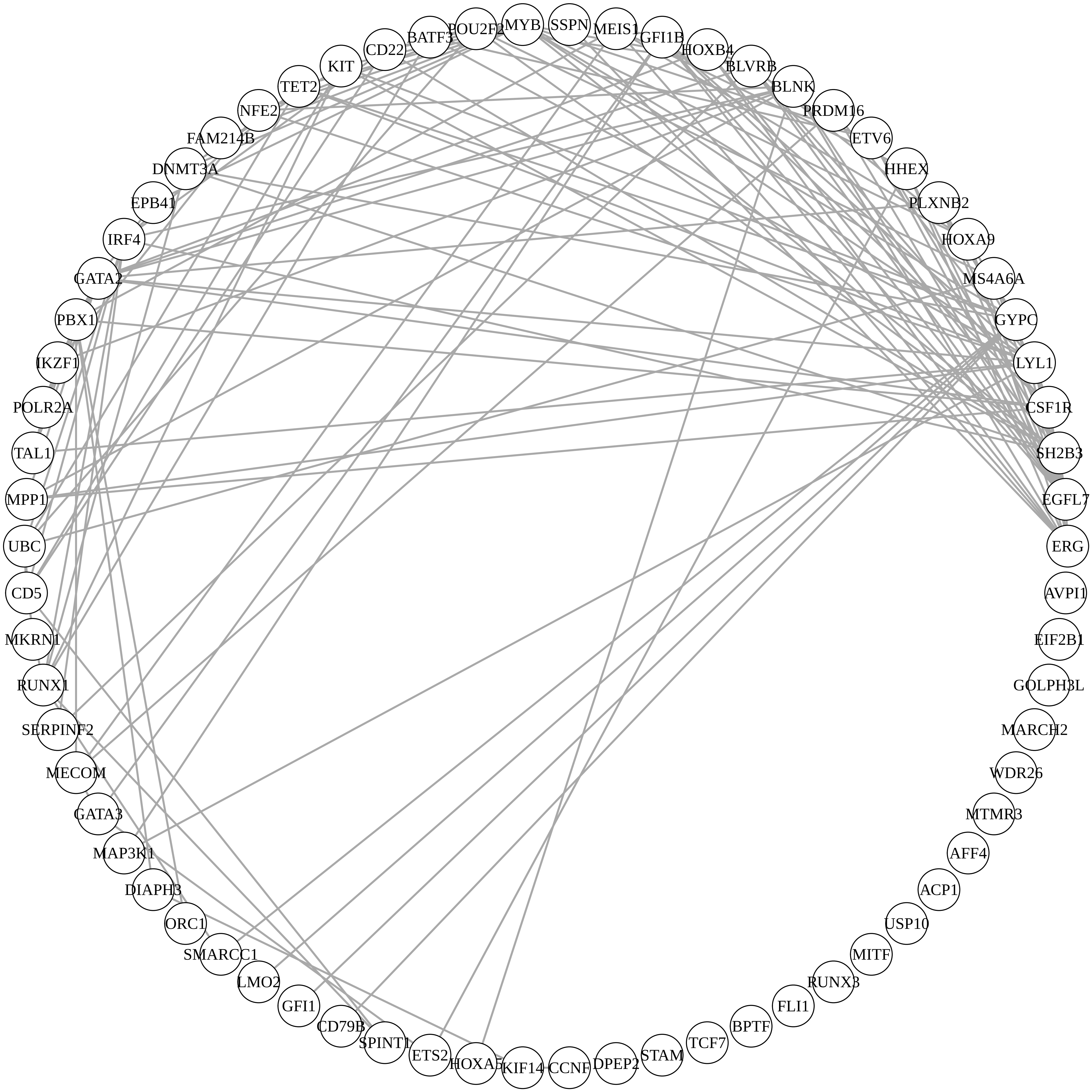

human_HSCtoGMP_time 4

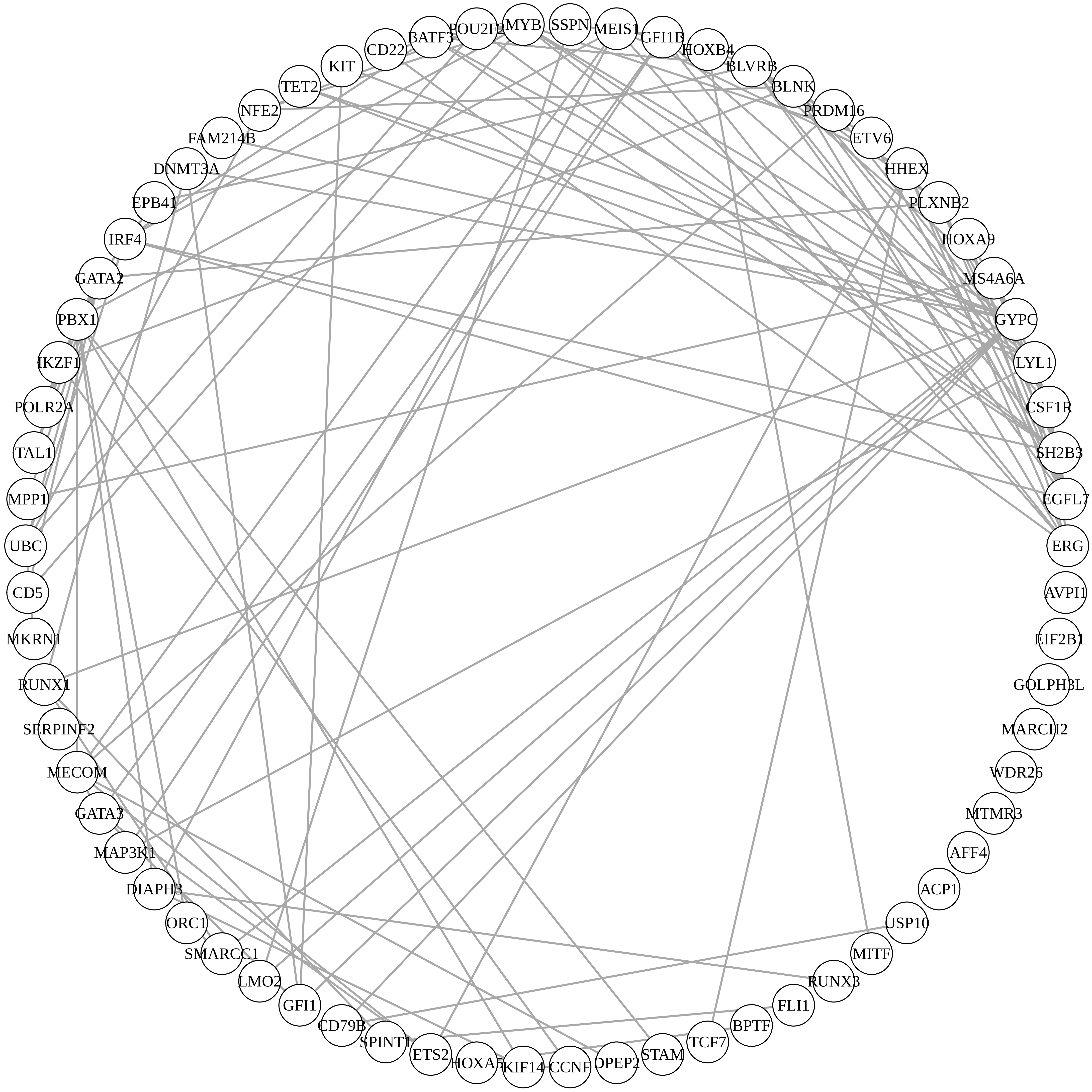

human_HSCtoGMP_time 5

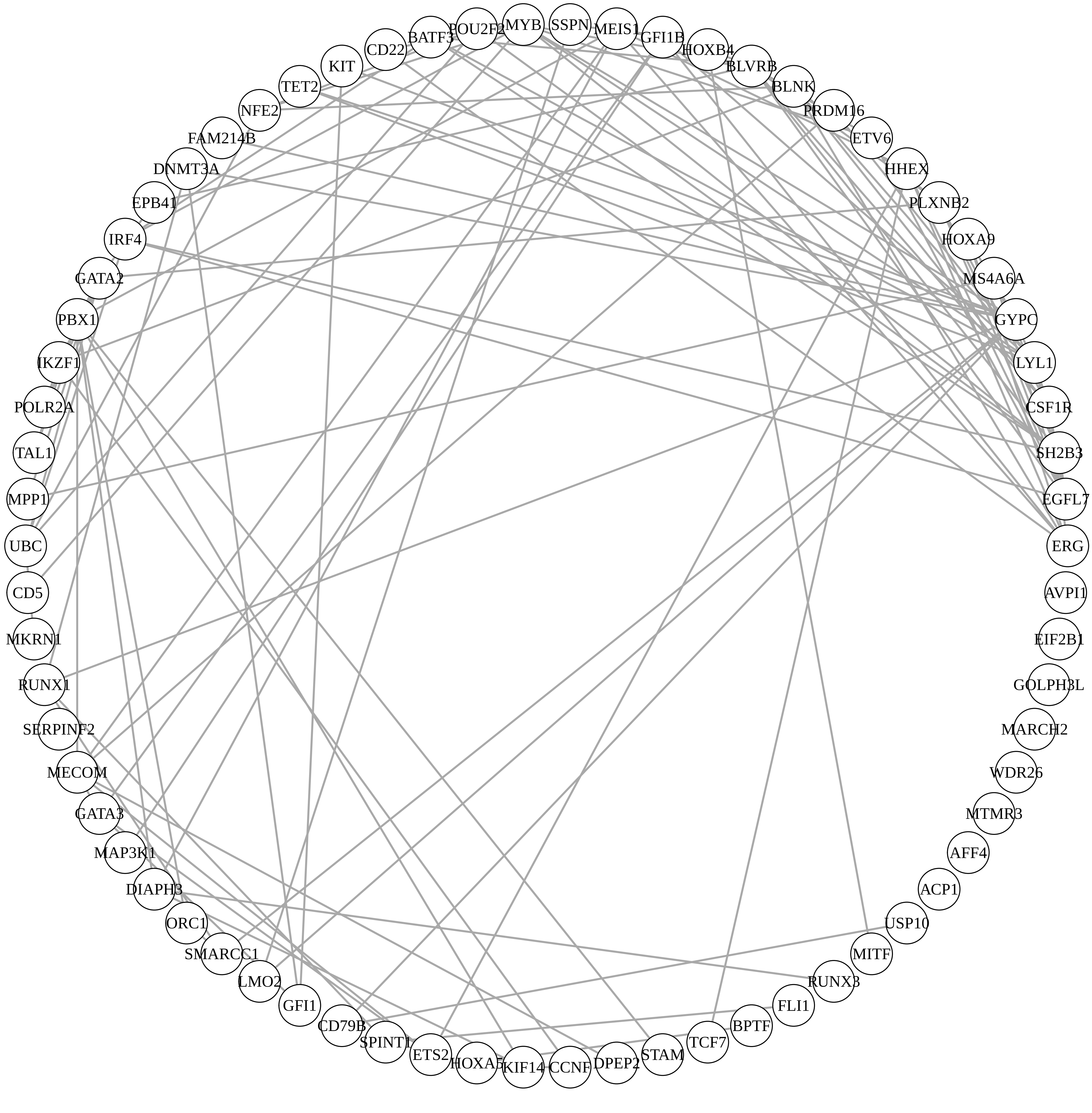

human_HSCtoGMP_time 6

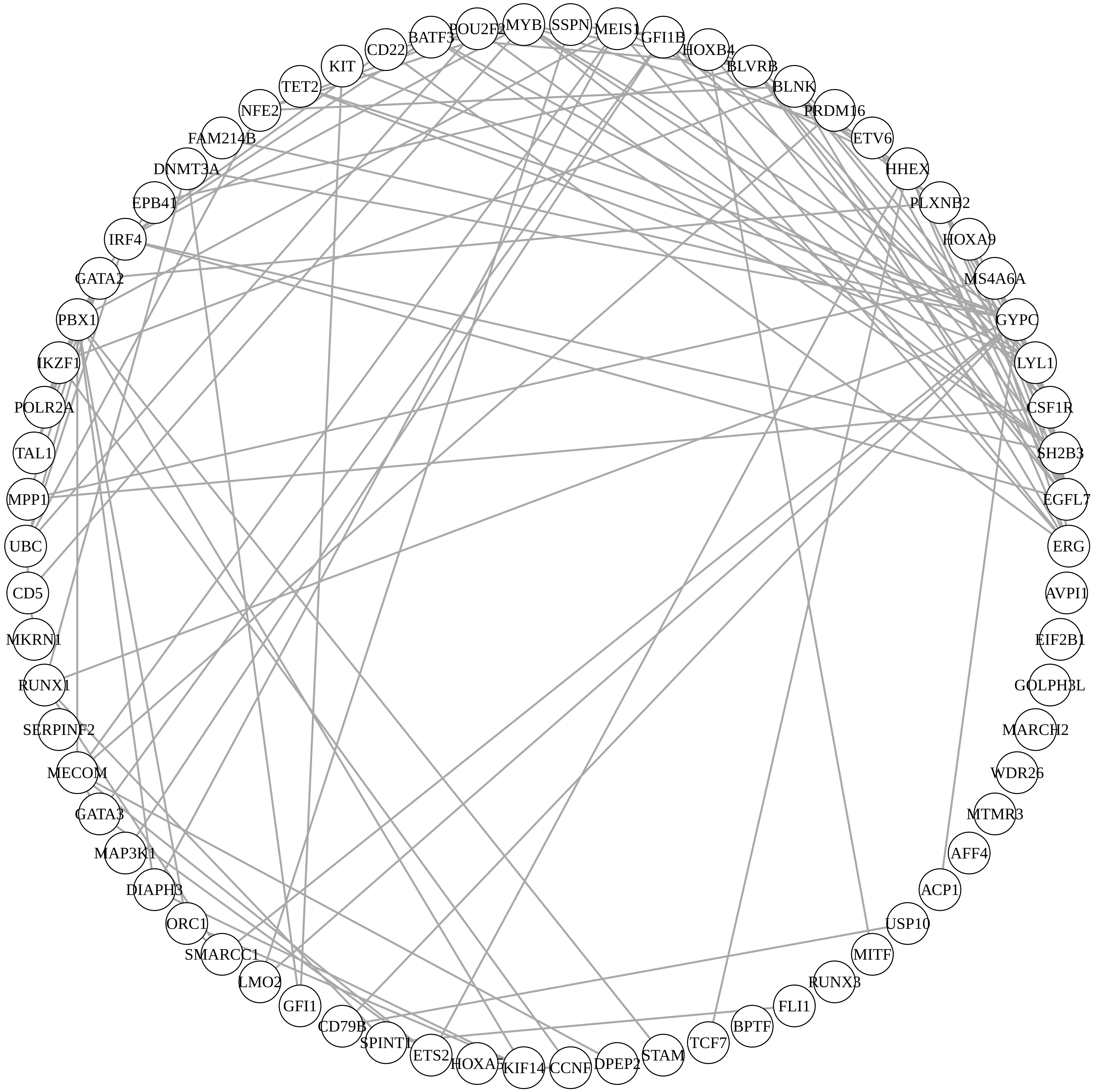

human_HSCtoGMP_time 7

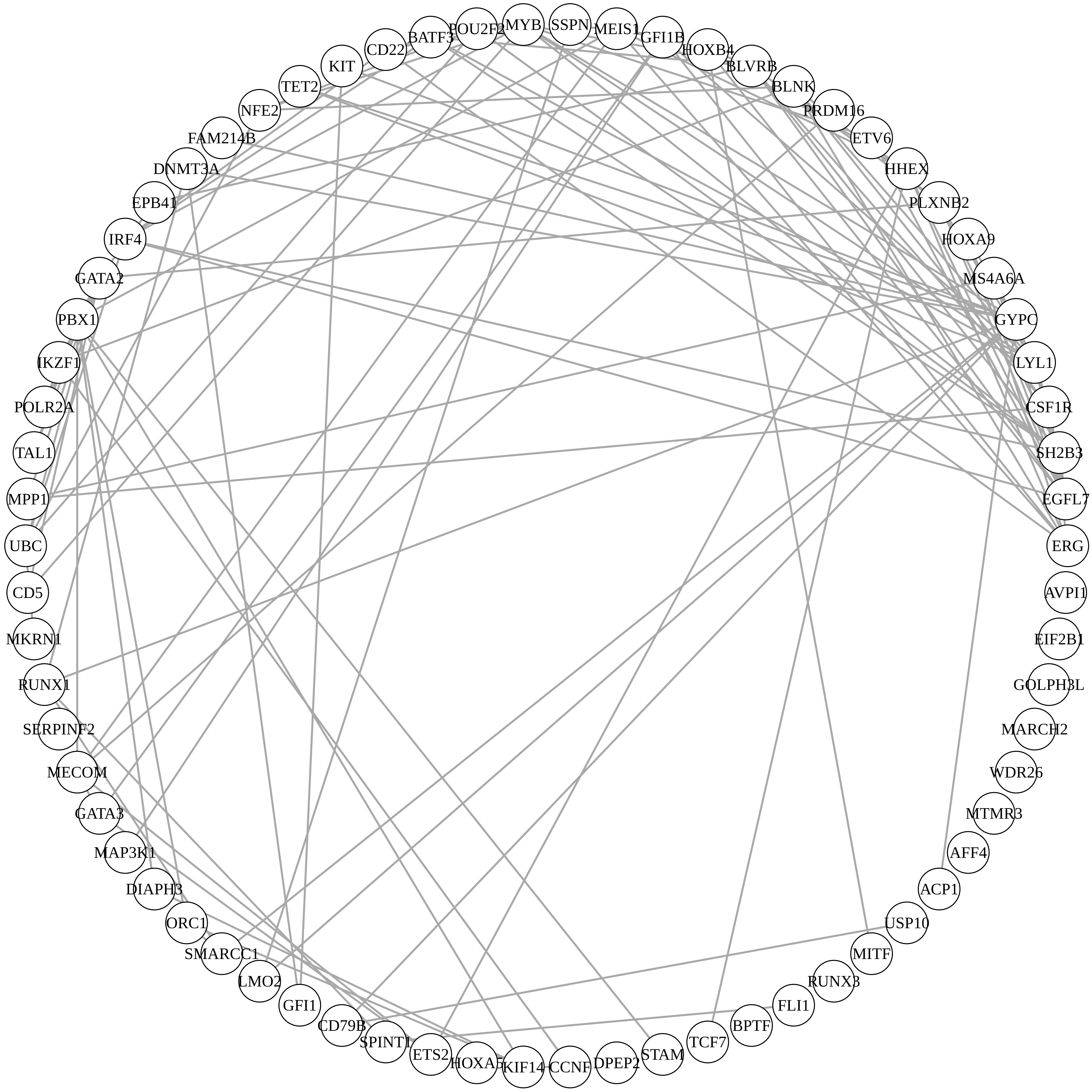

human_HSCtoGMP_time 8

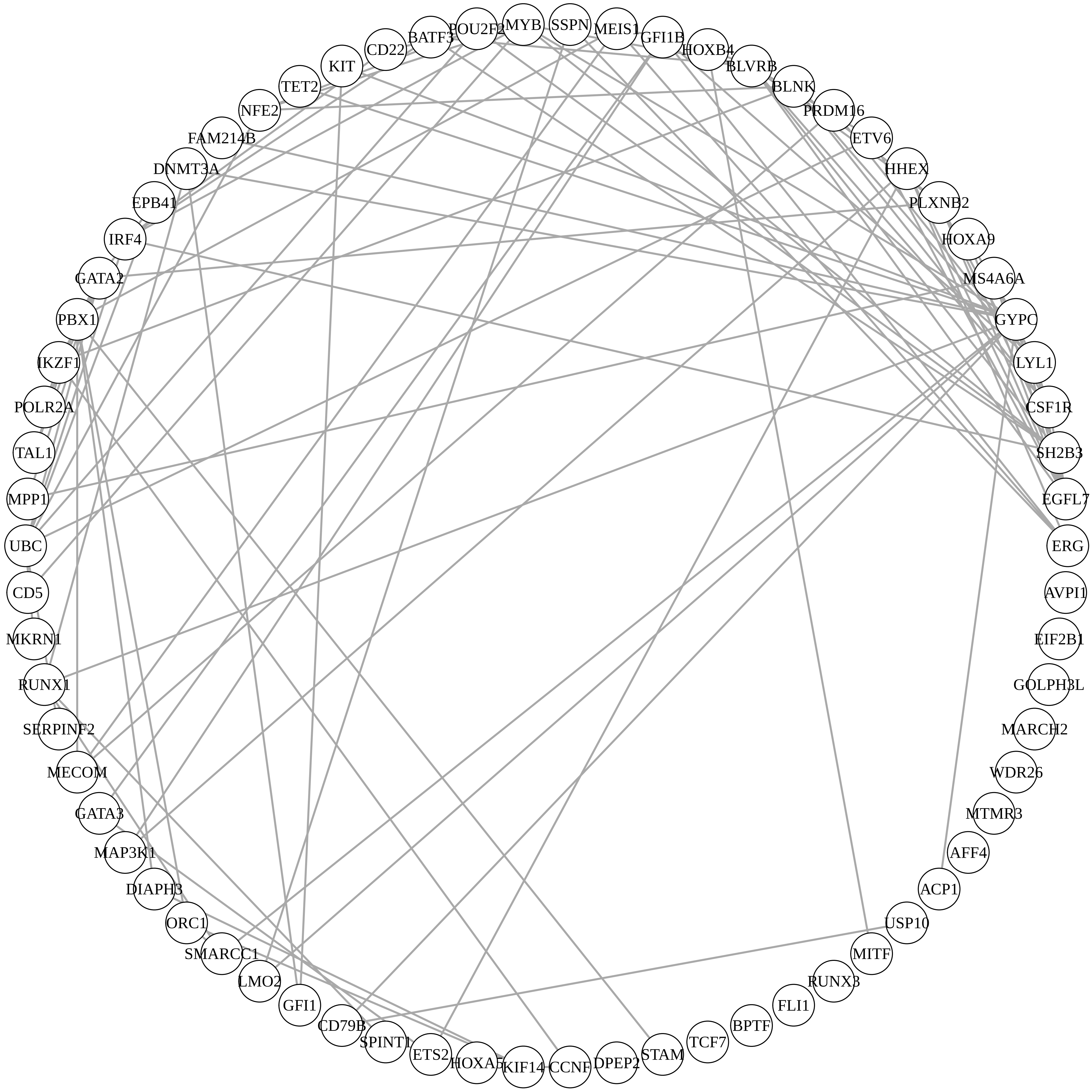

human_HSCtoGMP_time 9

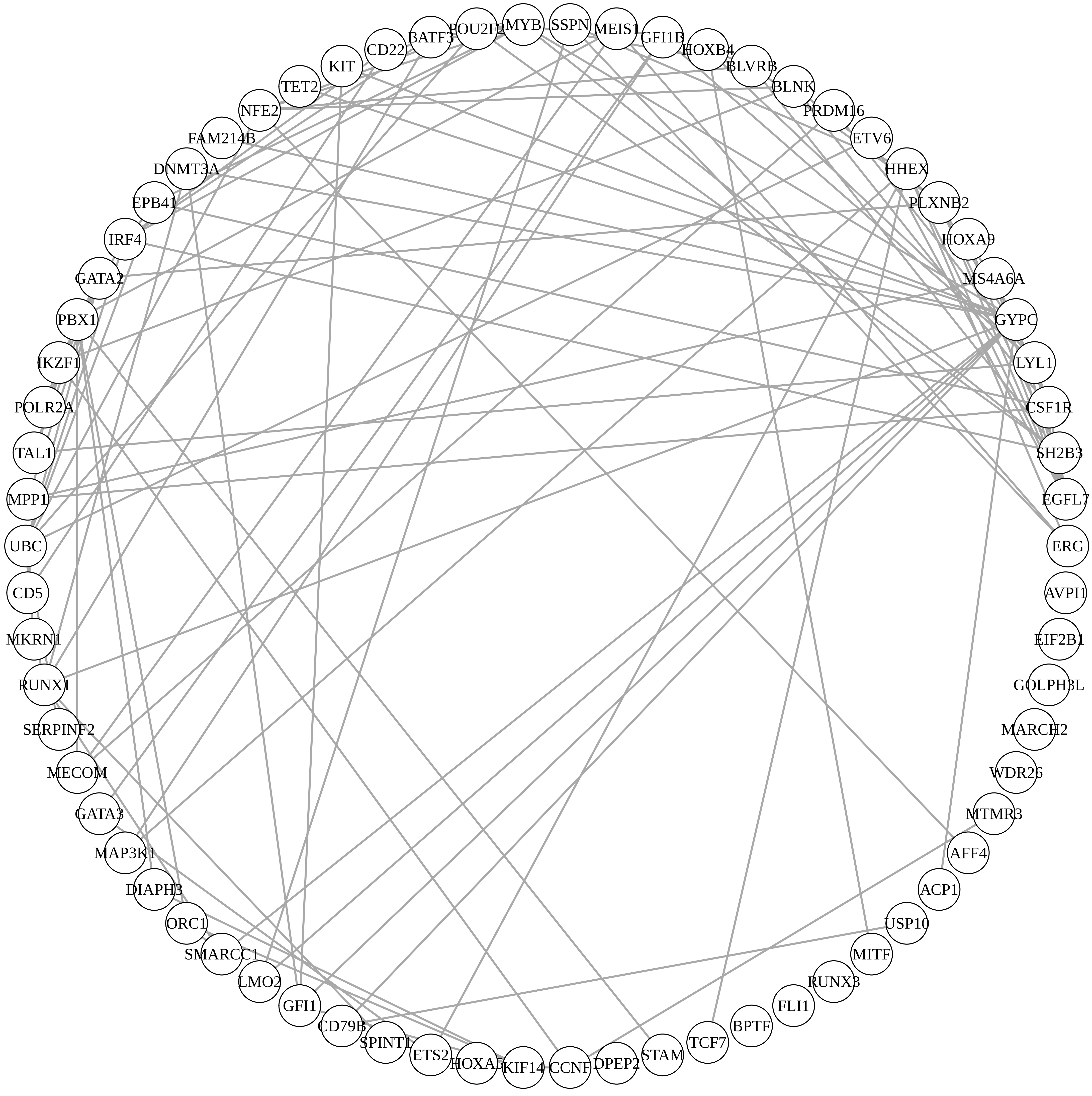

human_HSCtoGMP_time 10

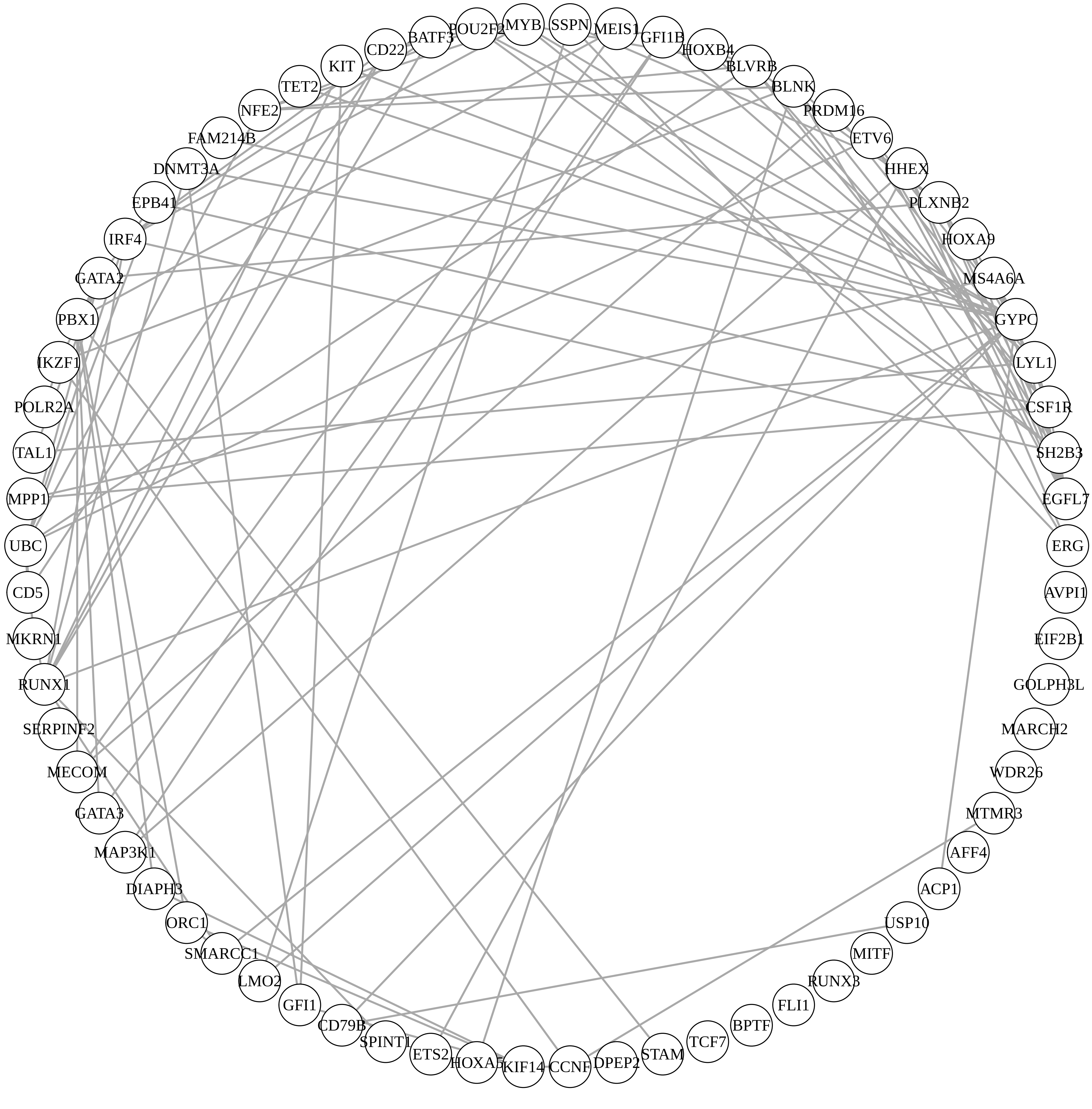

human_HSCtoGMP_time 11

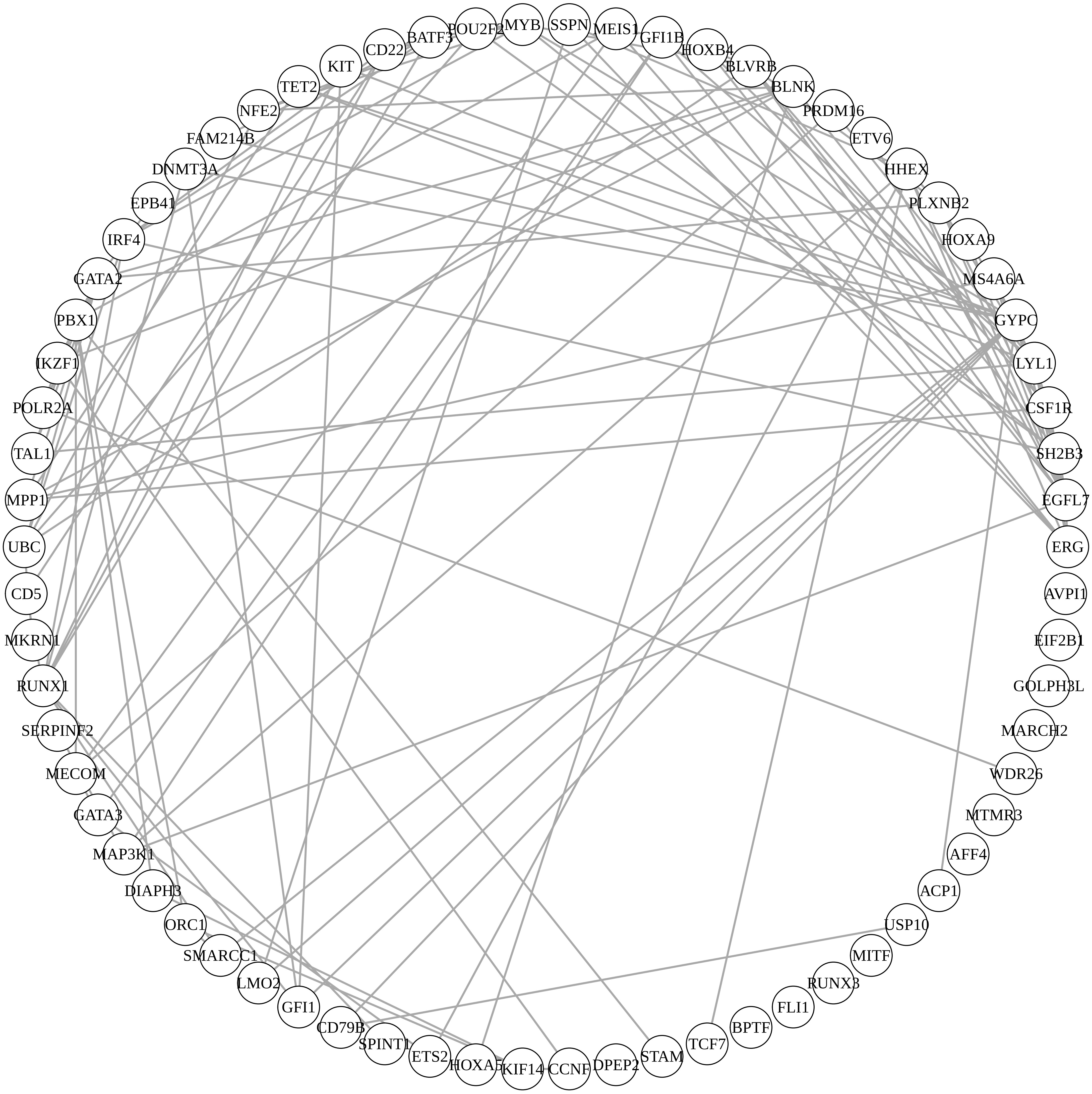

human_HSCtoGMP_time 12

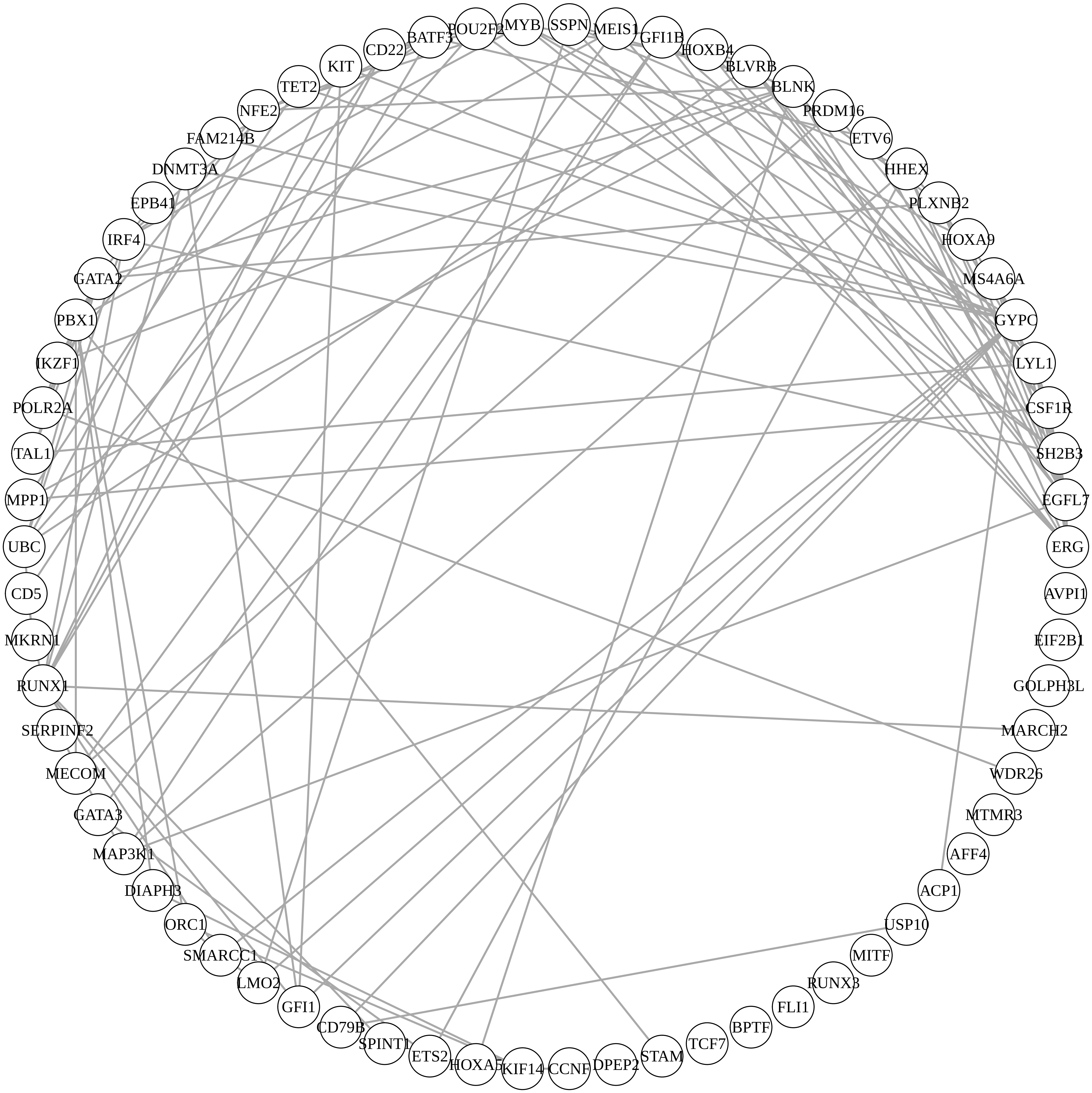

human_HSCtoGMP_time 13

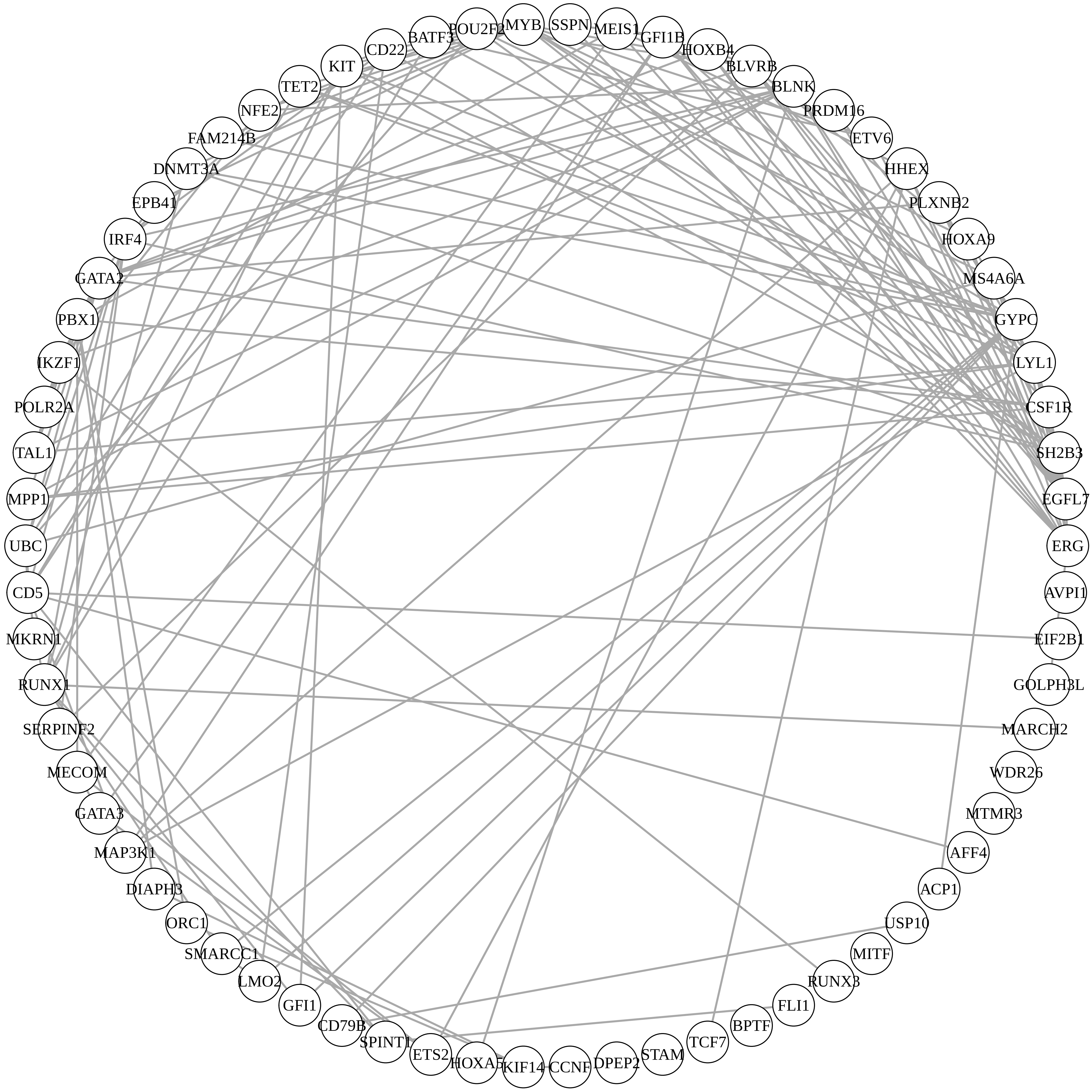

human_HSCtoGMP_time 14

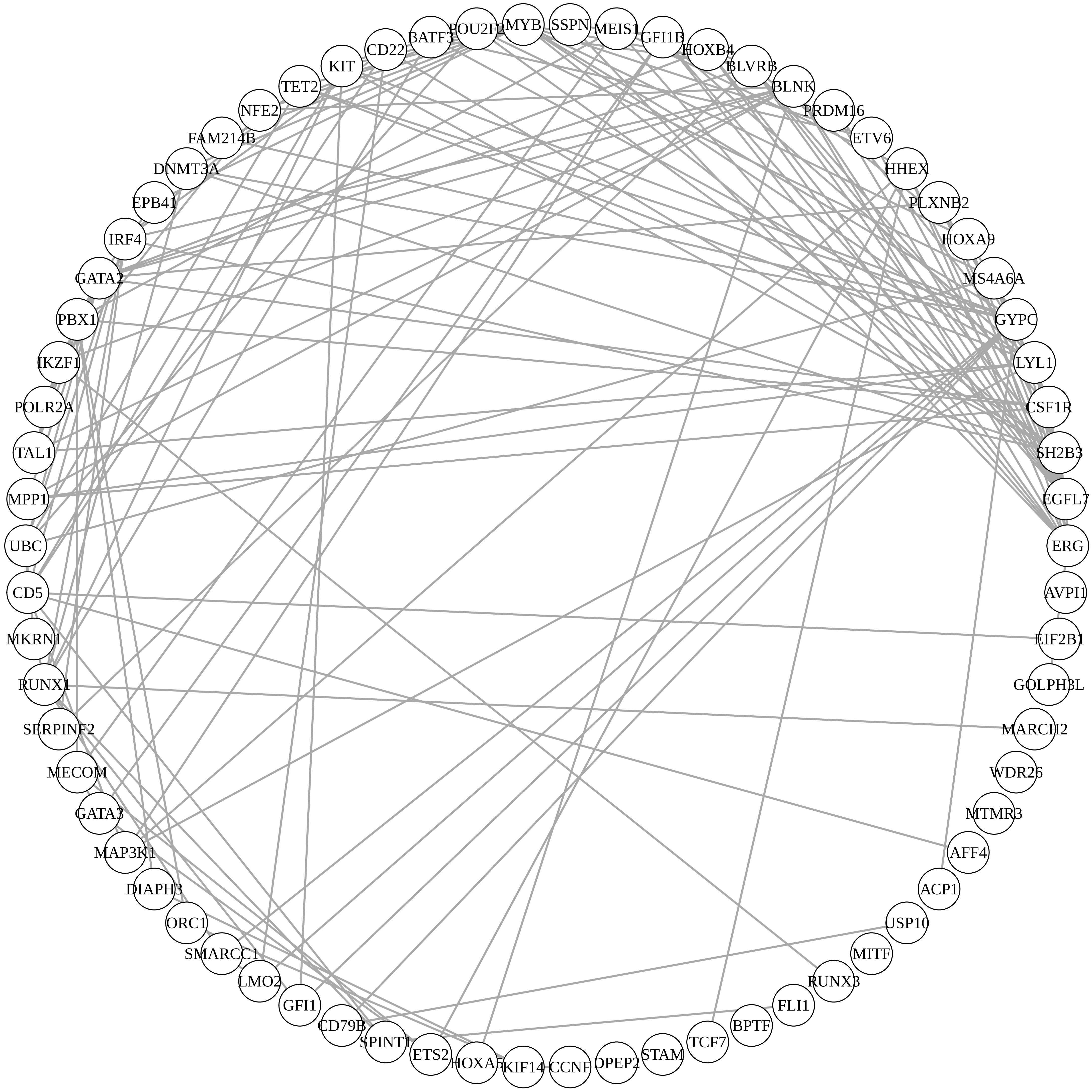

human_HSCtoGMP_time 15

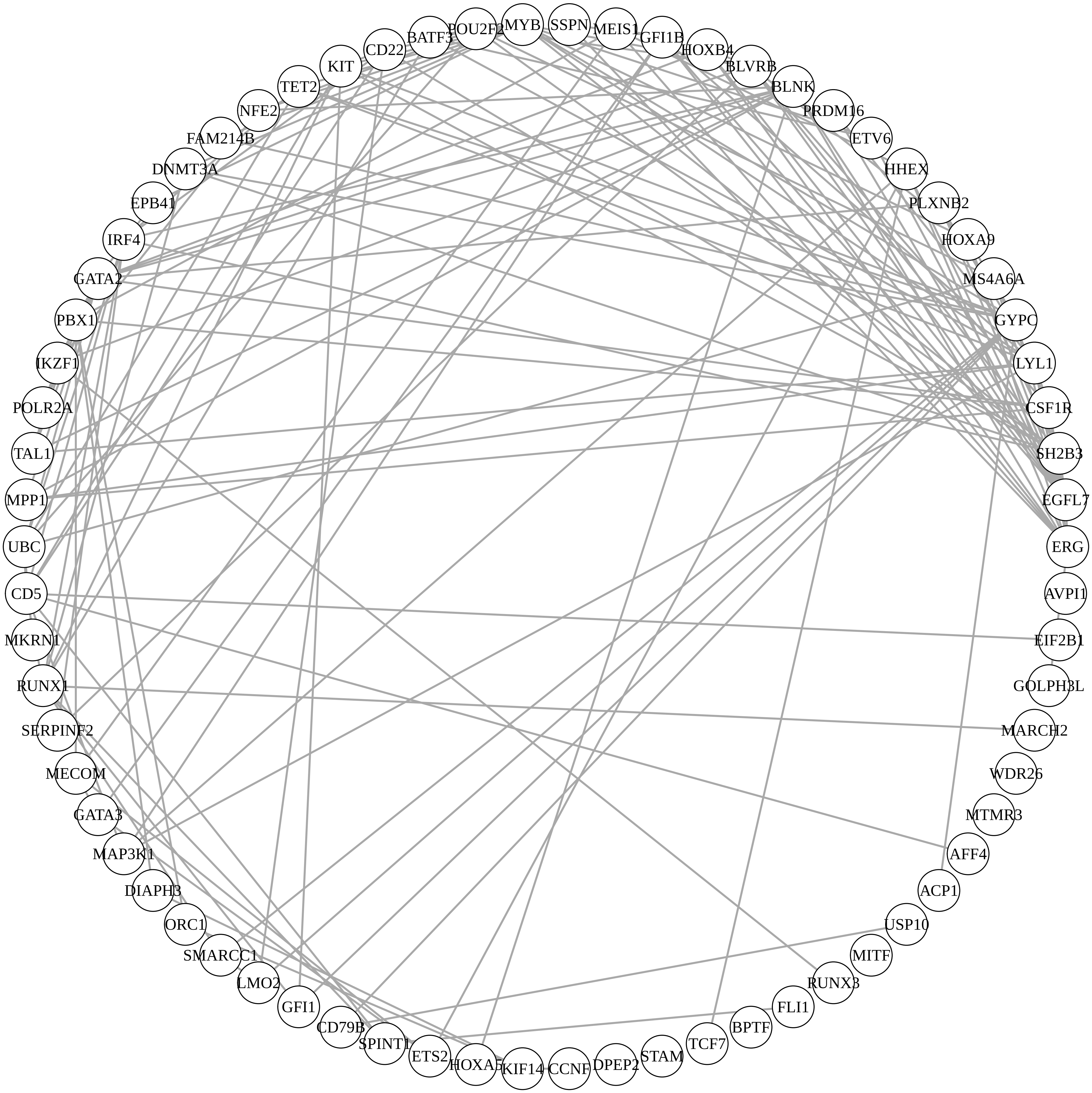

human_HSCtoGMP_time 16

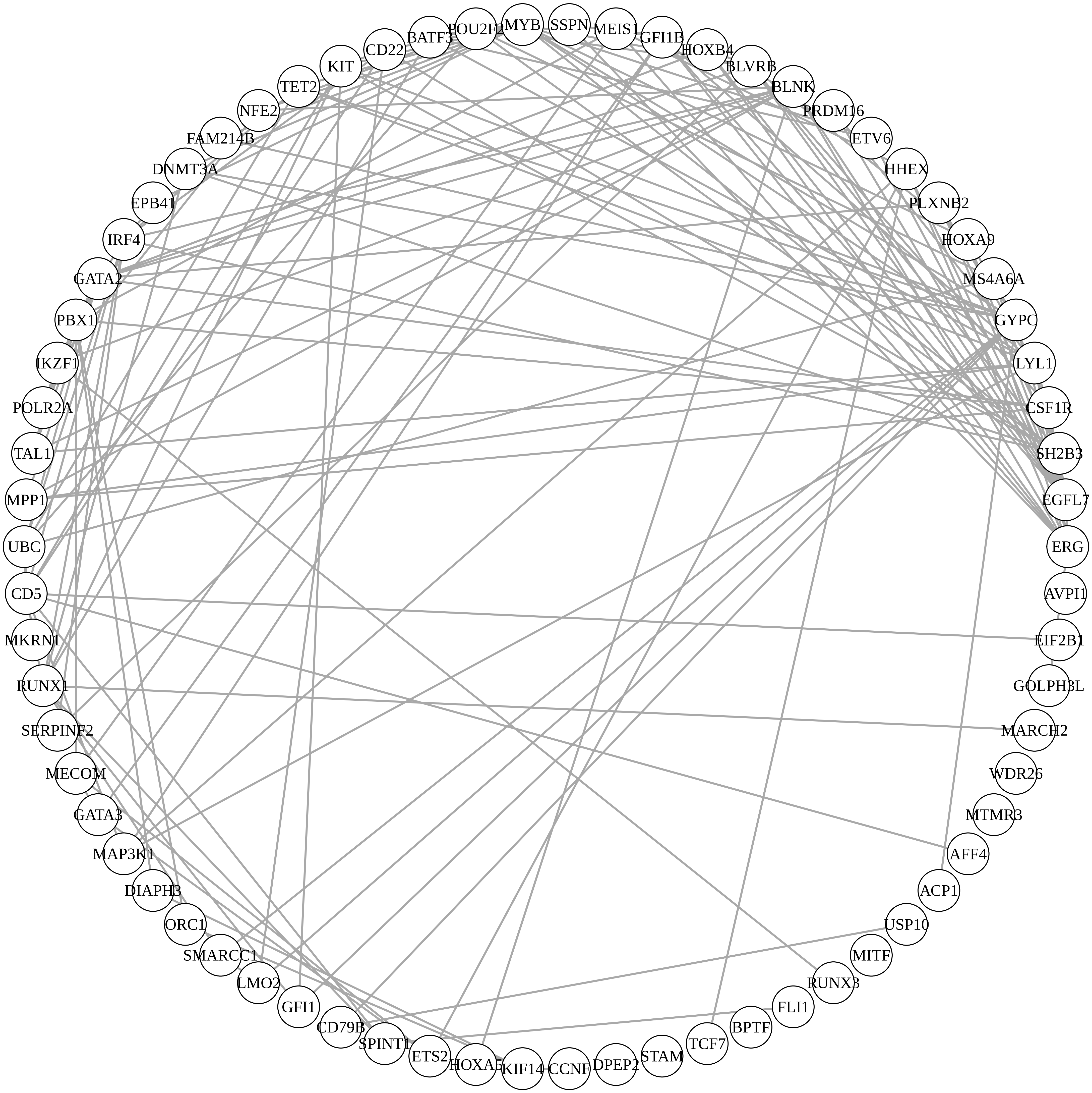

human_HSCtoGMP_time 17

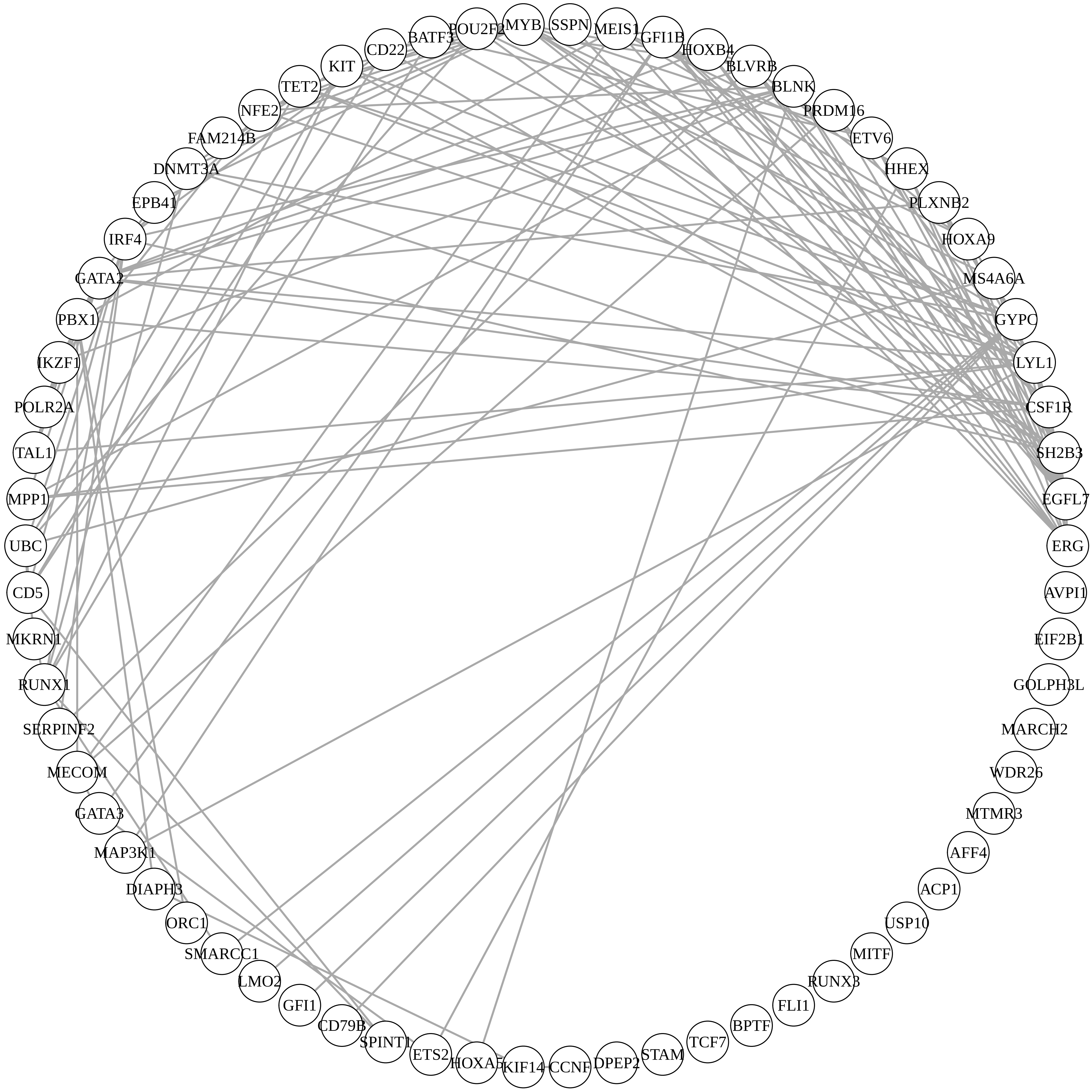

human_HSCtoGMP_time 18

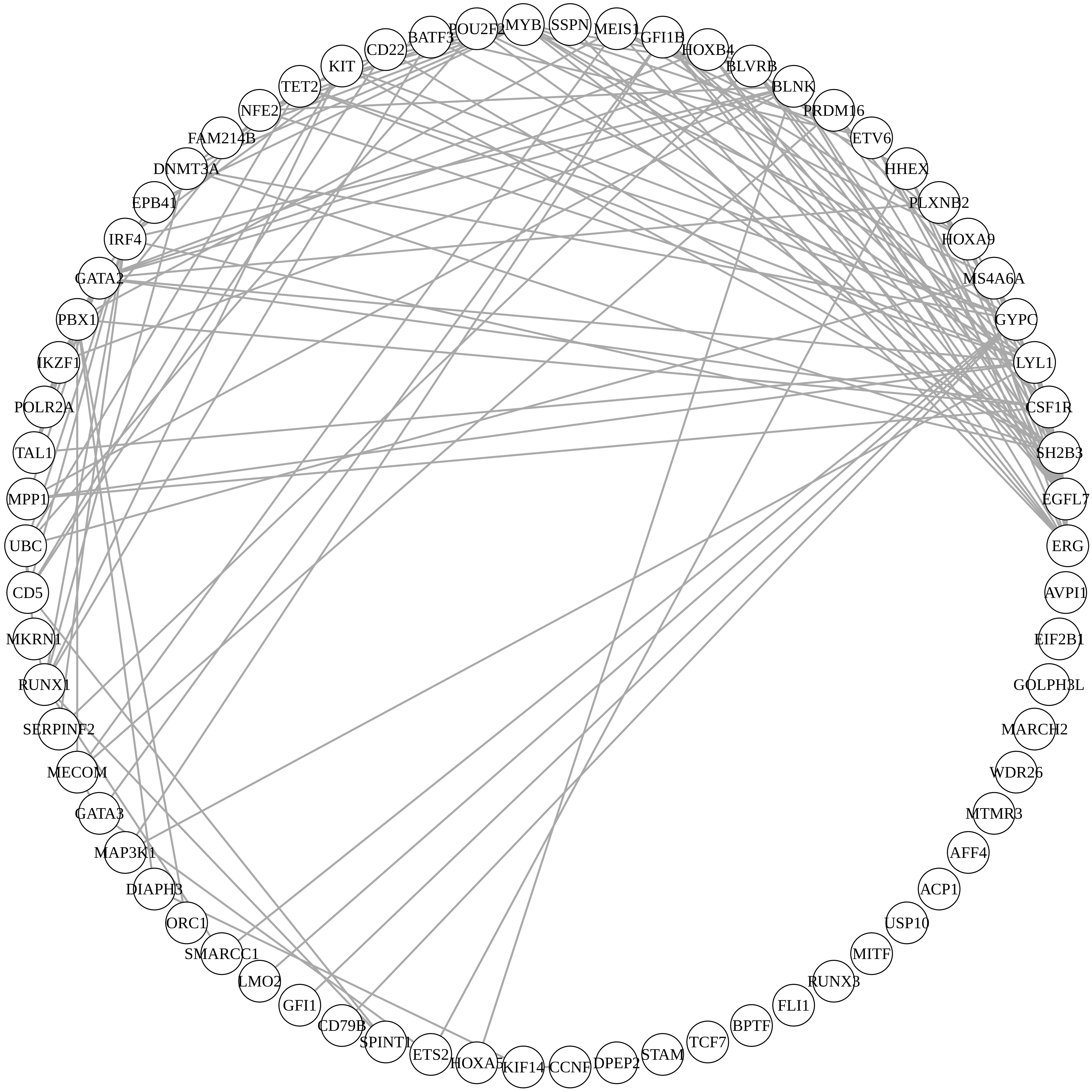

human_HSCtoGMP_time 19

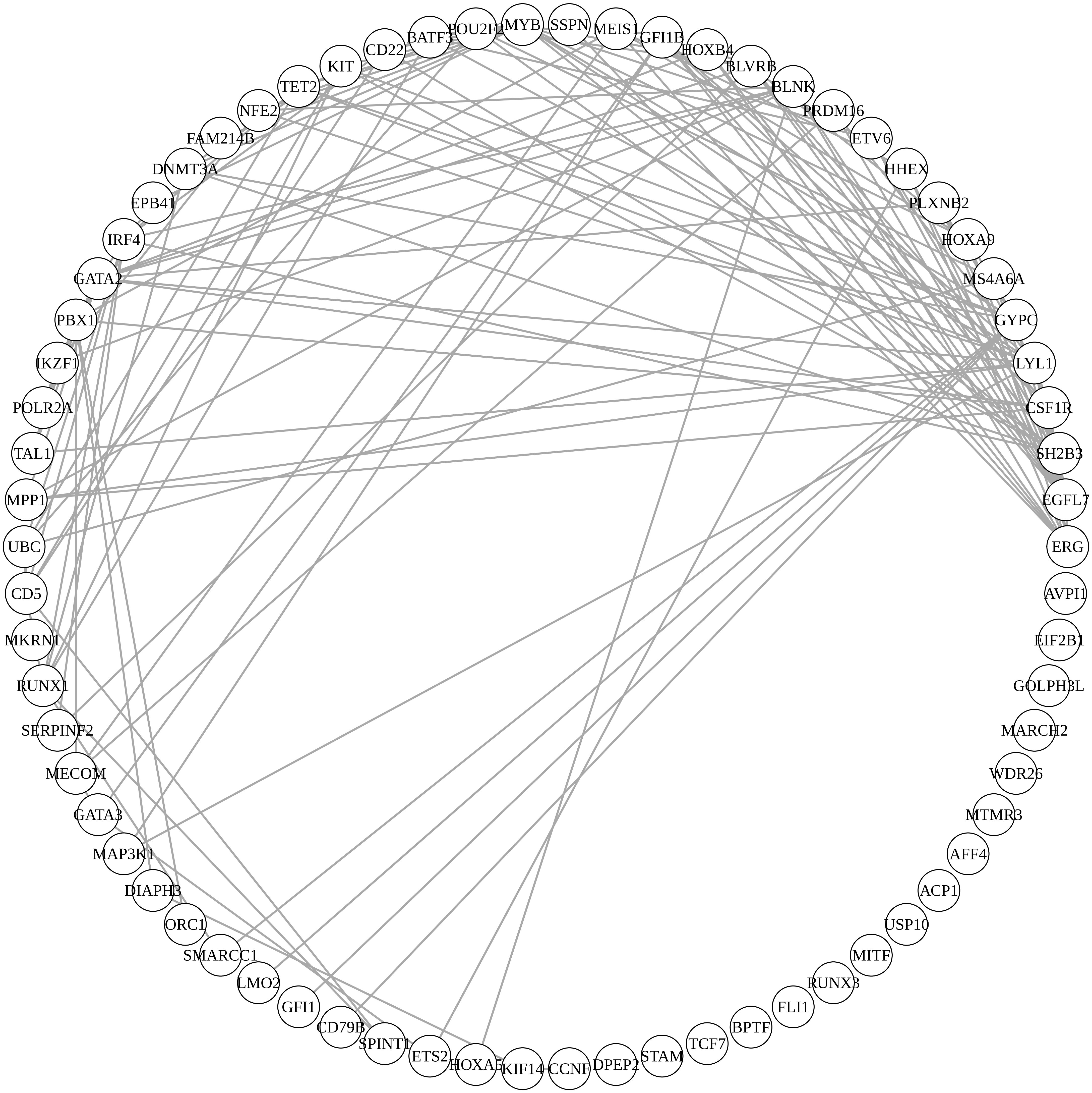

human_HSCtoGMP_time 20

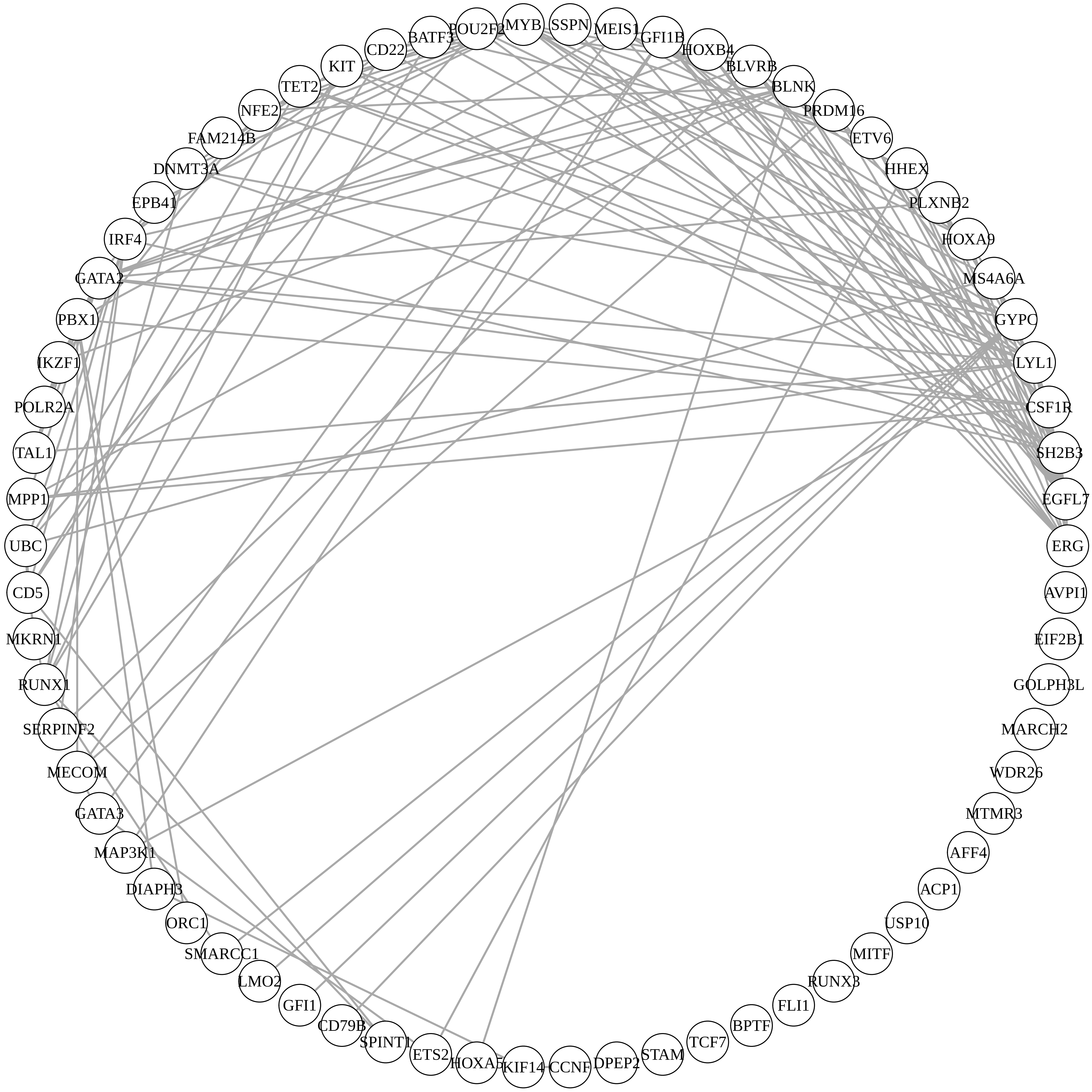

human_HSCtoGMP_time 21

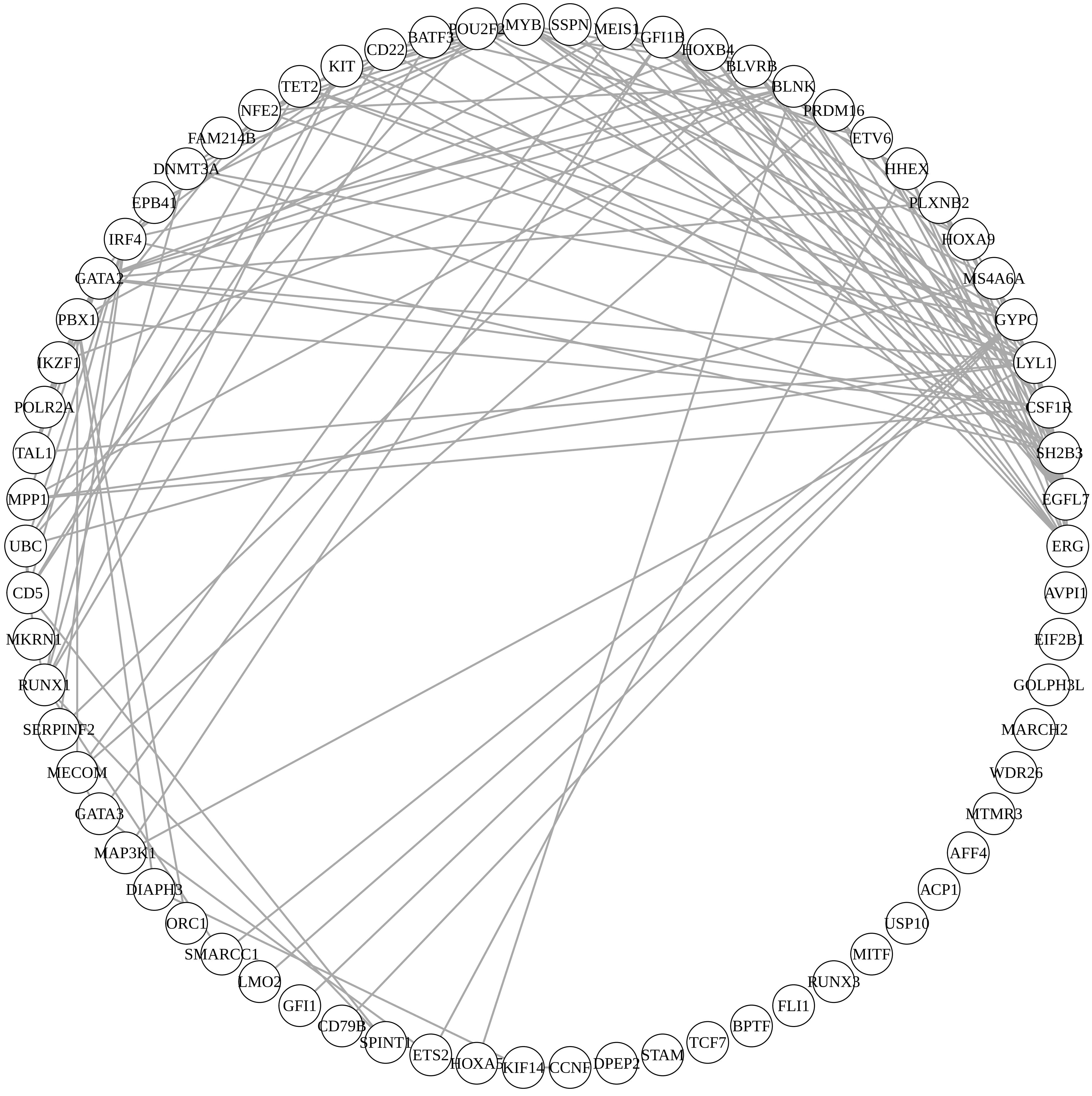

human_HSCtoGMP_time 22

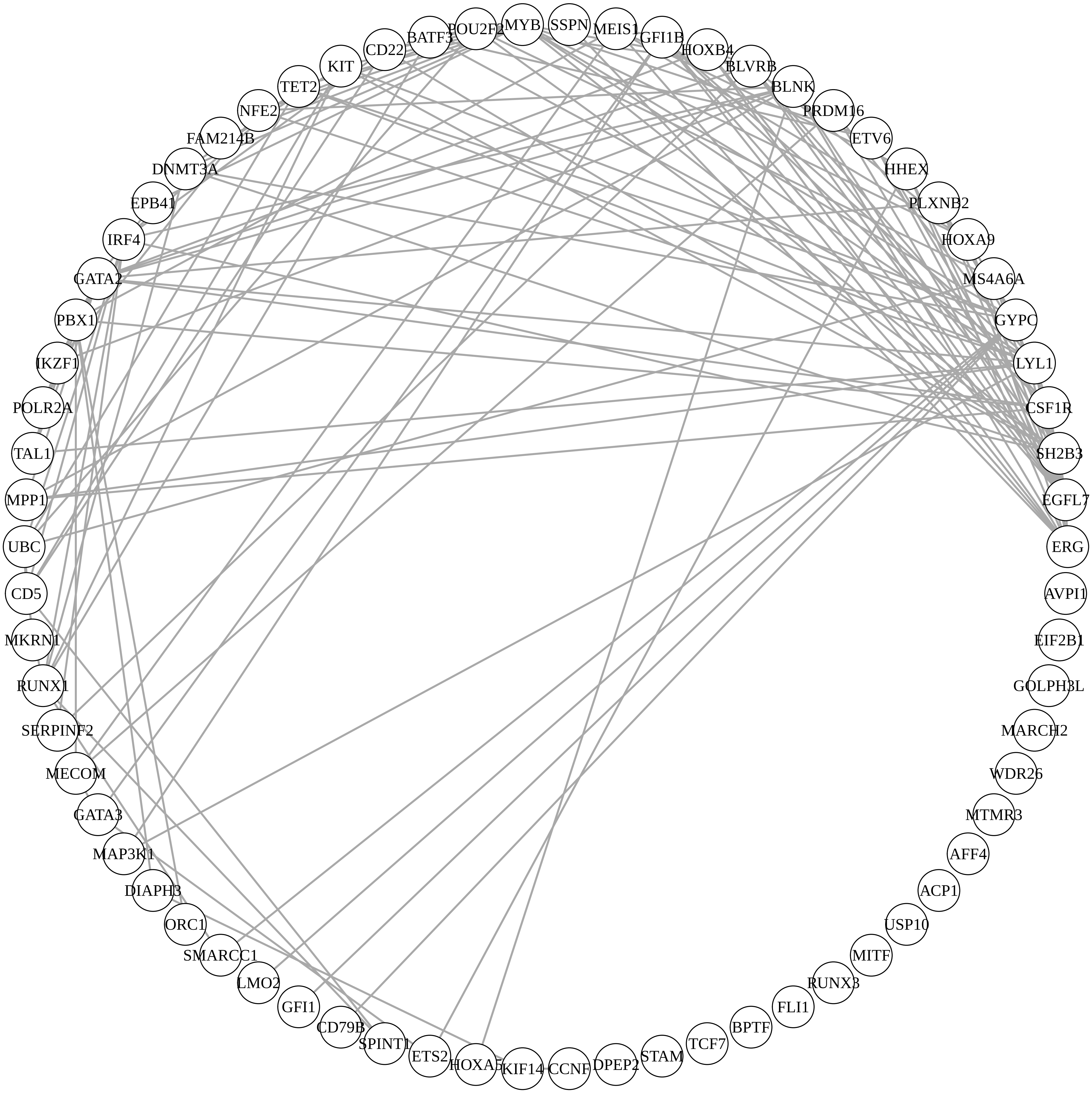

human_HSCtoGMP_time 23

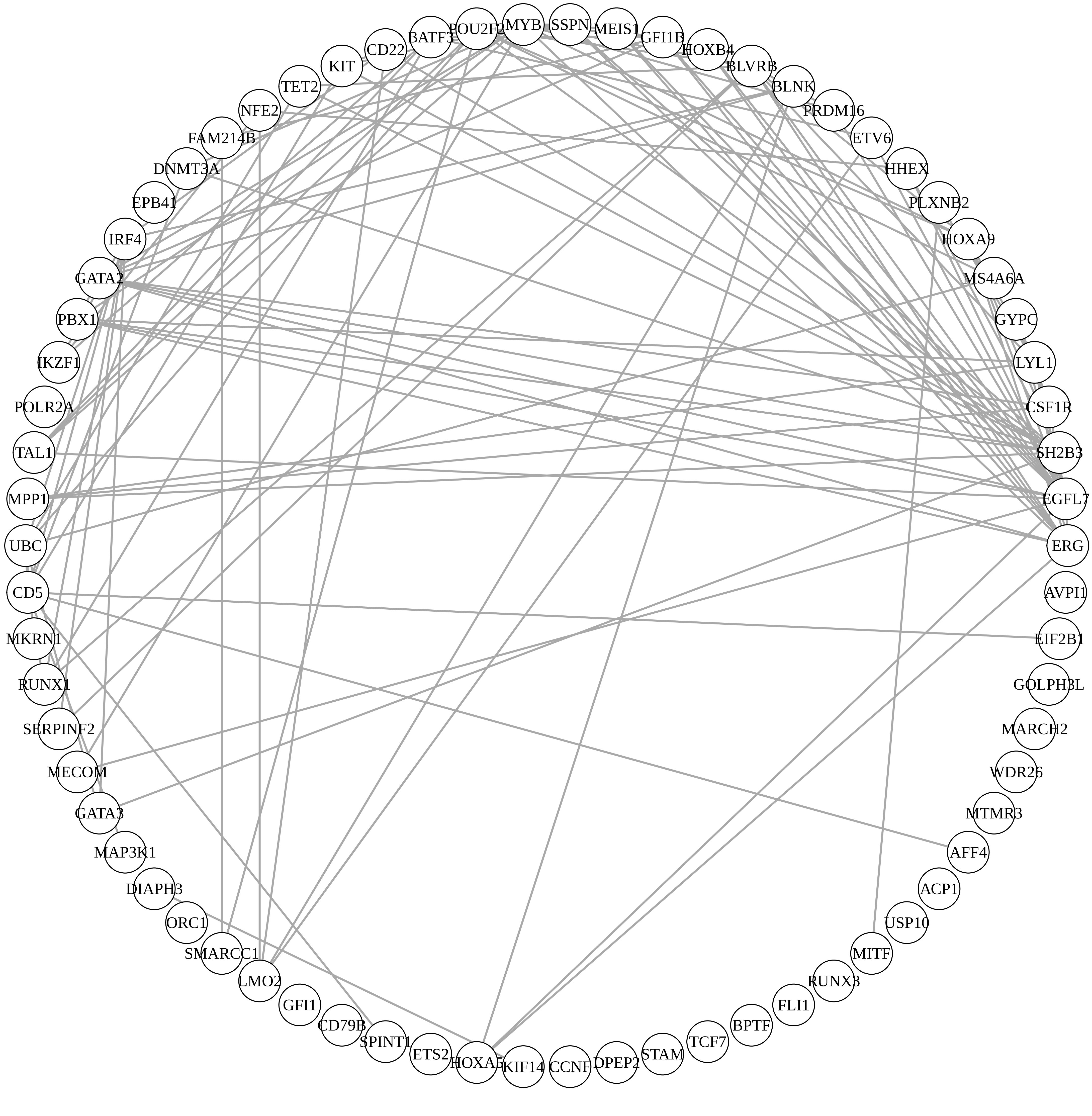

human_HSCtoGMP_time 24

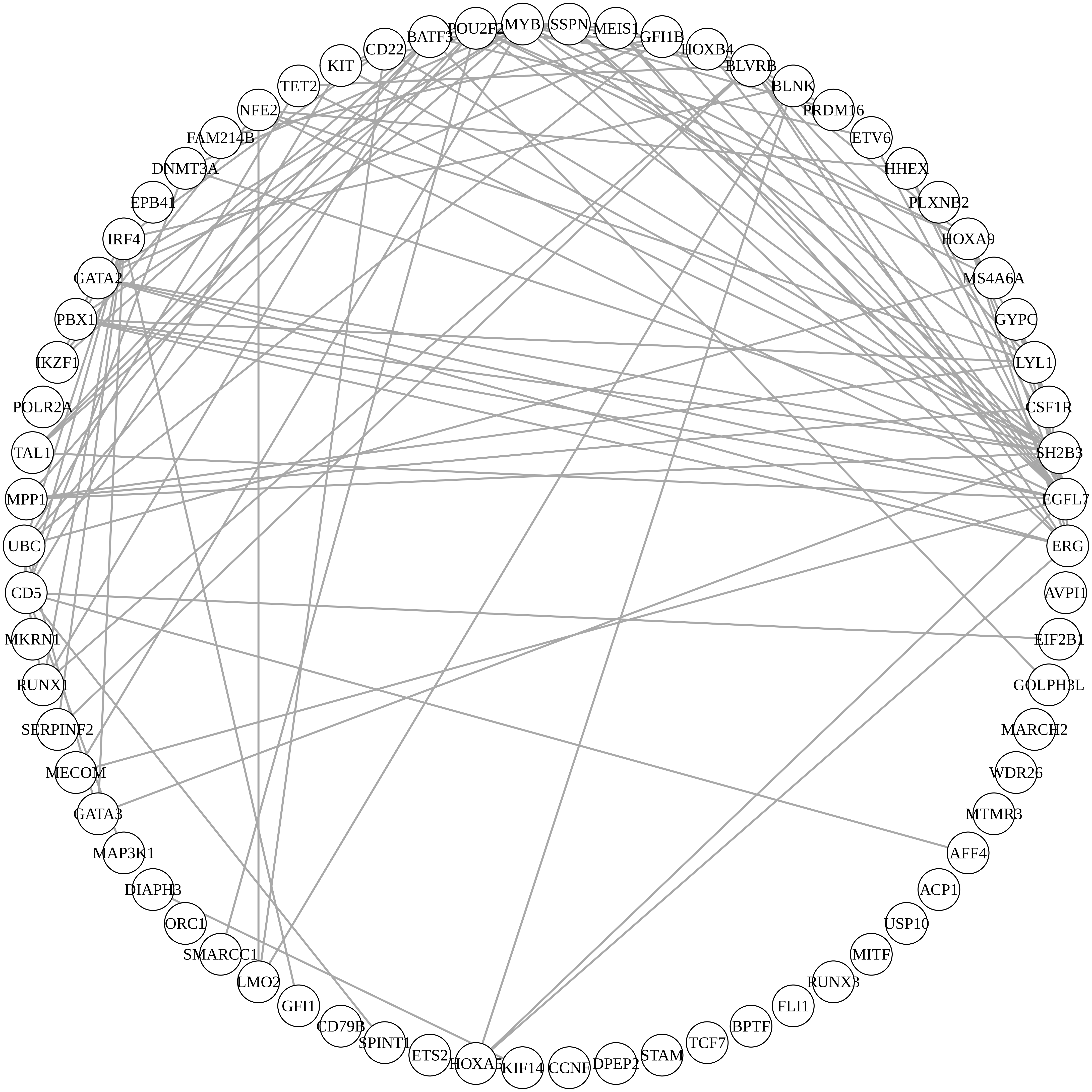

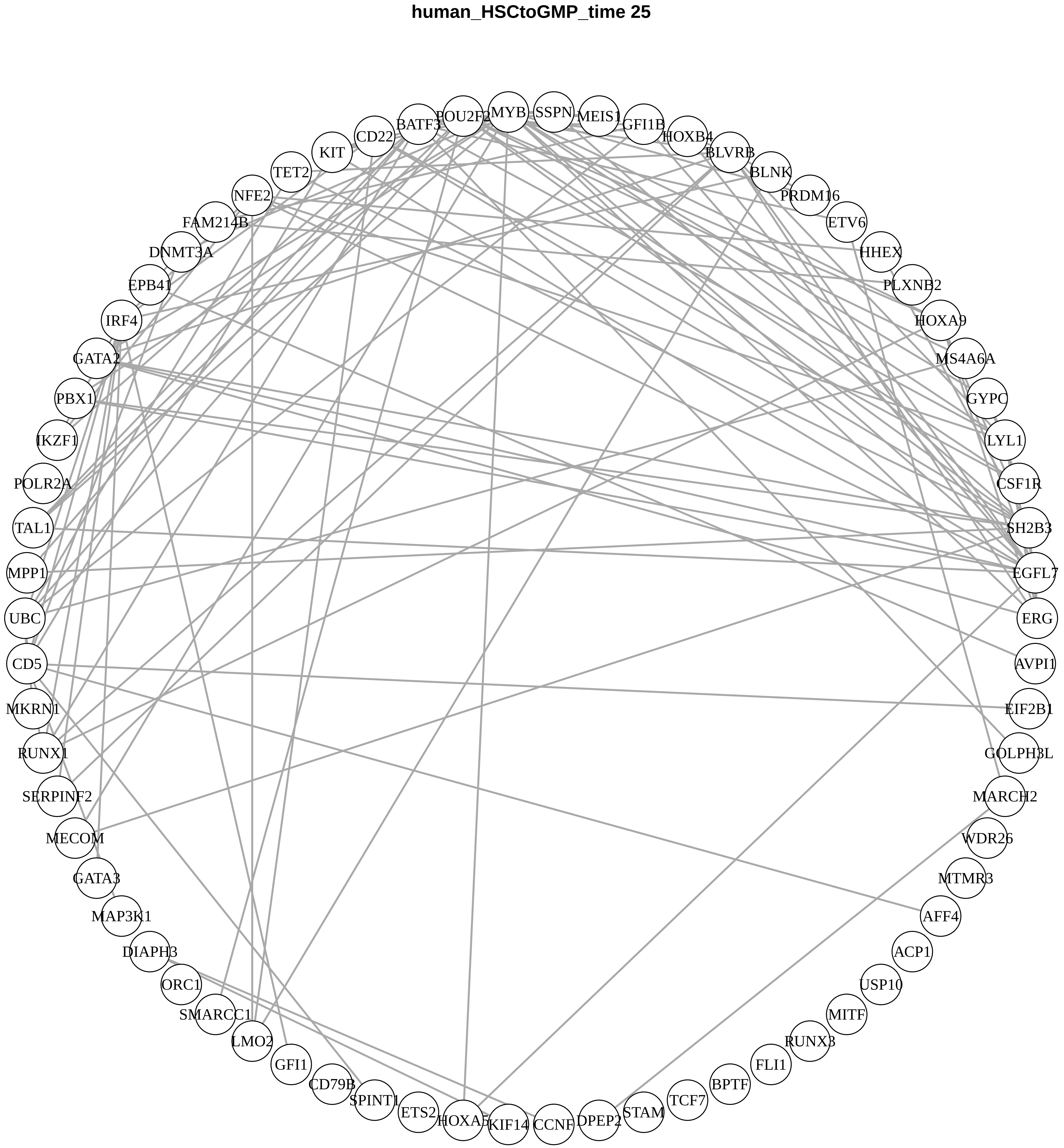

human_HSCtoGMP_time 25
MYB
SSPN
POU2F2
MEIS1
BATF3
GFI1B
CD22
HOXB4
KIT
BLVRB
TET2
BLNK
NFE2
PRDM16
FAM214B
ETV6
DNMT3A
HHEX
EPB41
PLXNB2
IRF4
HOXA9
GATA2
MS4A6A
PBX1
GYPC
IKZF1
LYL1
POLR2A
CSF1R
TAL1
SH2B3
MPP1
EGFL7
UBC
ERG
CD5
AVPI1
MKRN1
EIF2B1
RUNX1
GOLPH3L
SERPINF2
MARCH2
MECOM
WDR26
GATA3
MTMR3
MAP3K1
AFF4
DIAPH3
ACP1
ORC1
USP10
SMARCC1
MITF
LMO2
RUNX3
GFI1
FLI1
CD79B
BPTF
SPINT1
TCF7
ETS2
STAM
HOXA5
DPEP2
KIF14
CCNF

human_HSCtoLMPP_time 1

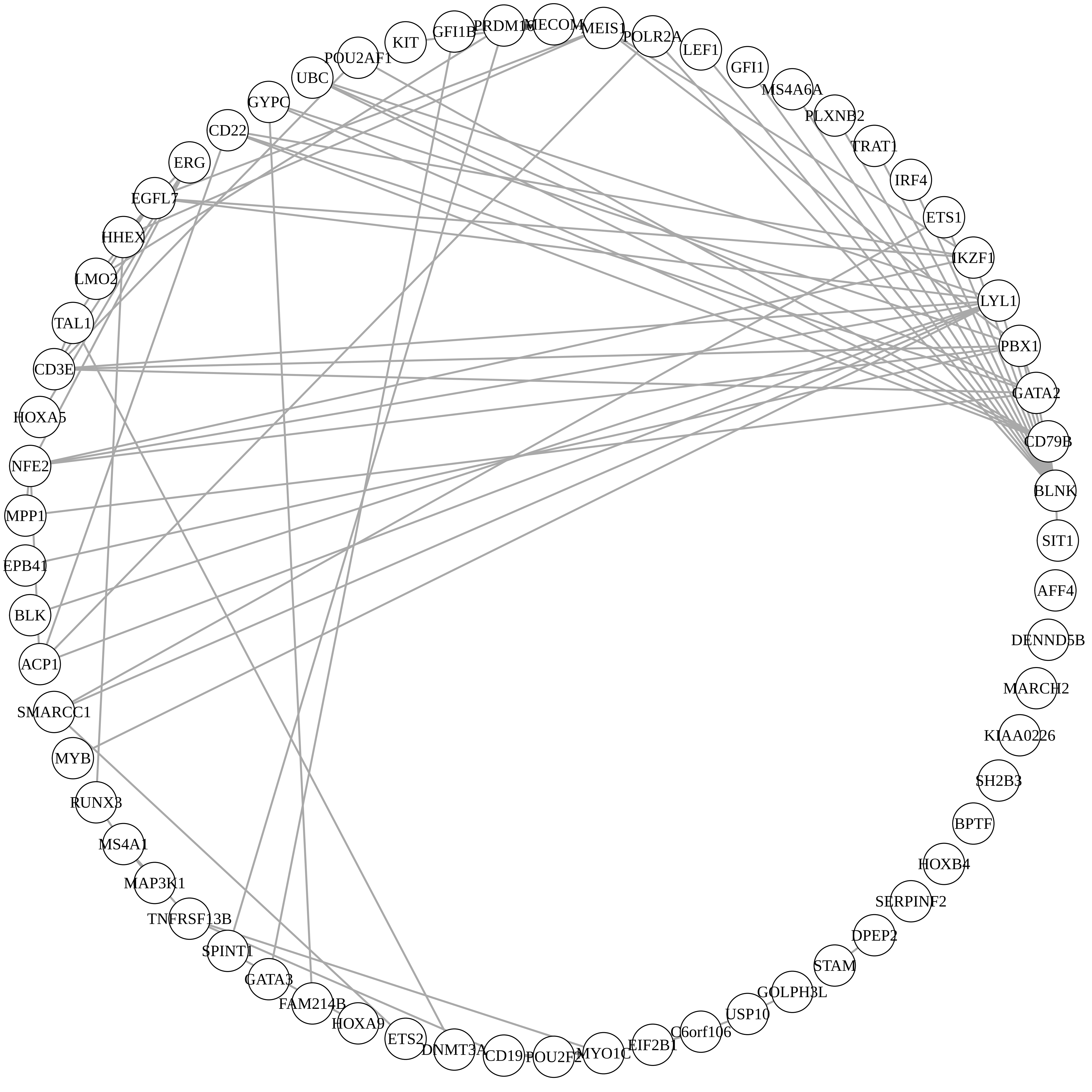

human_HSCtoLMPP_time 2

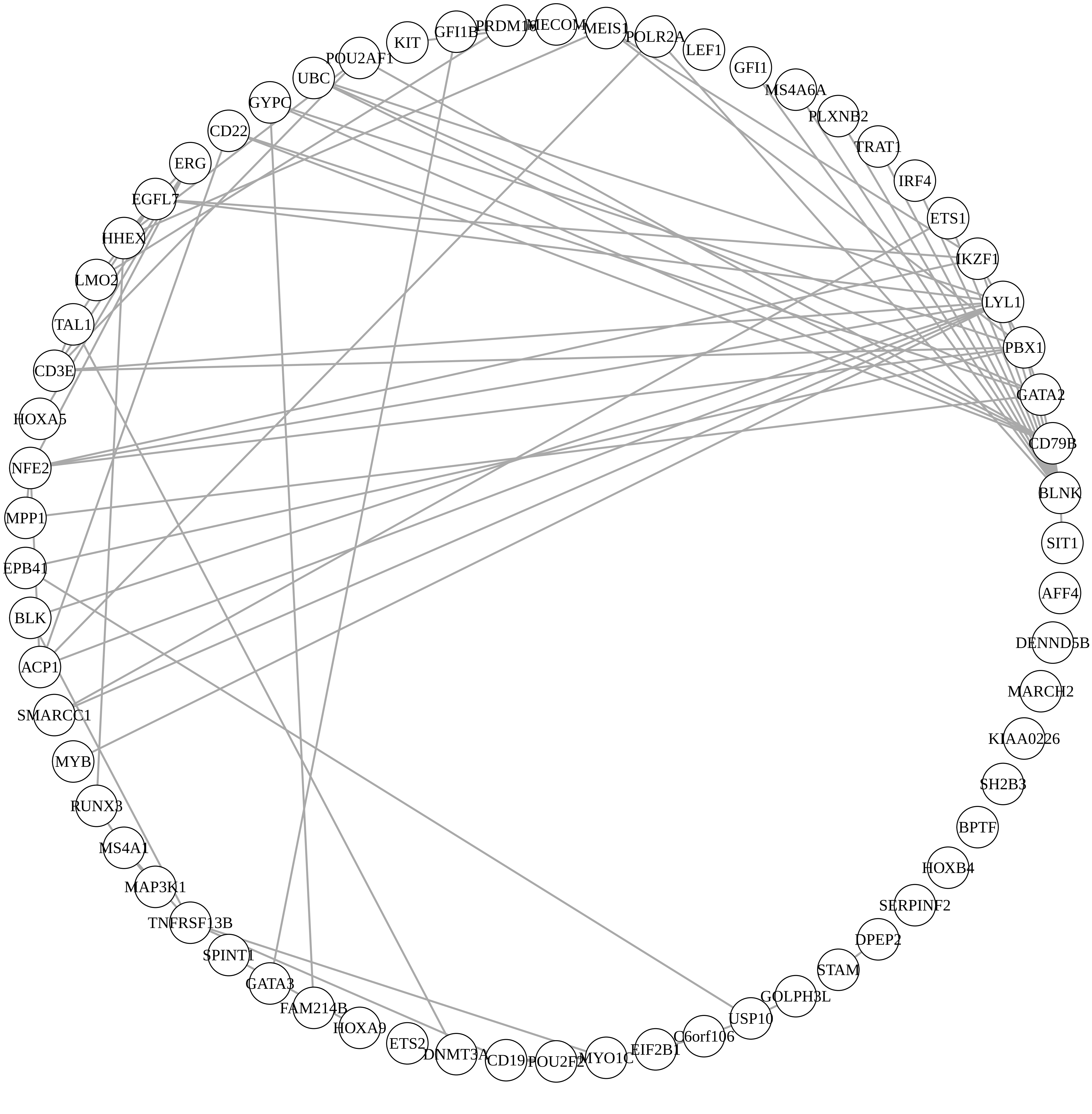

human_HSCtoLMPP_time 3

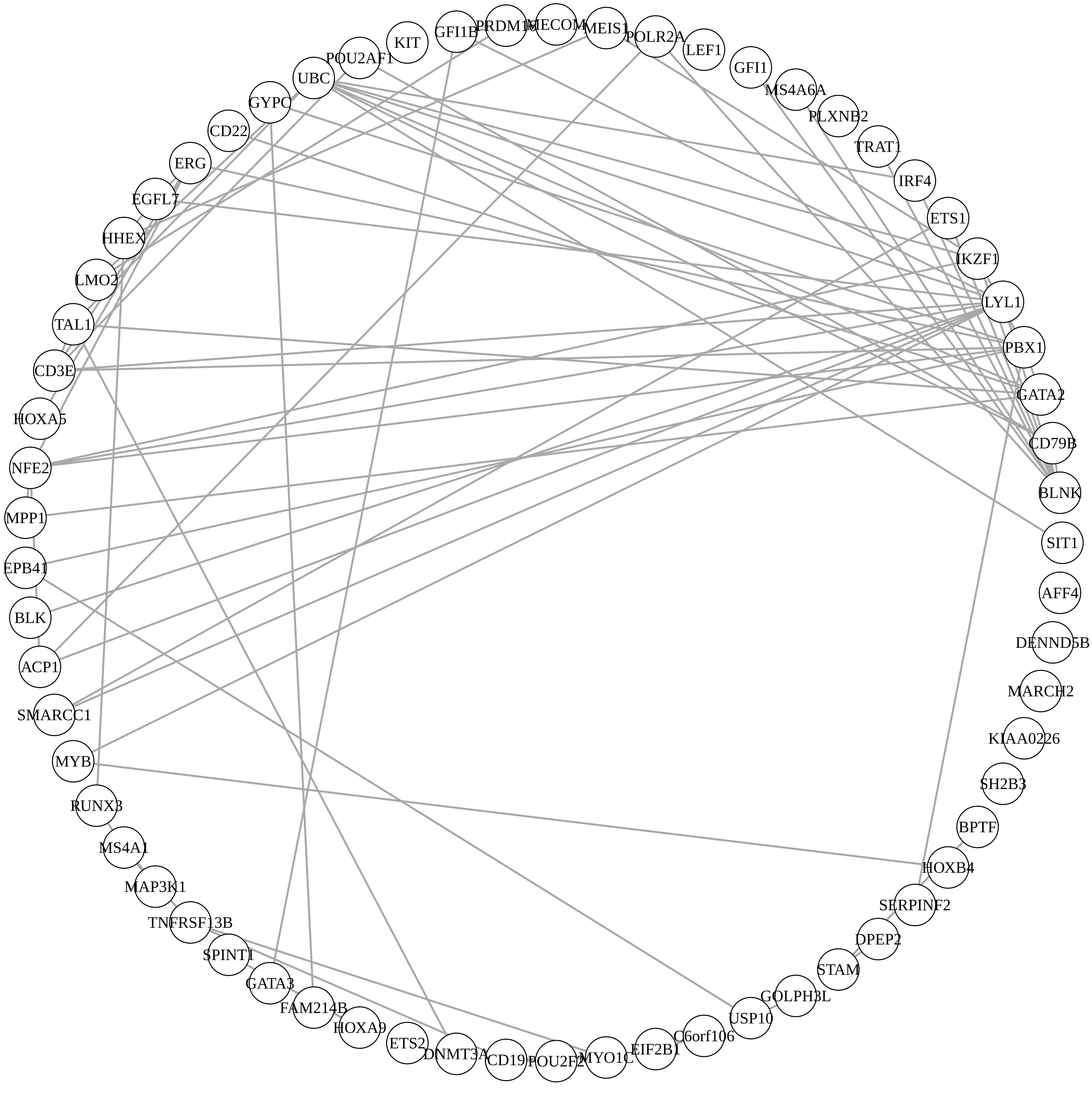

human_HSCtoLMPP_time 4

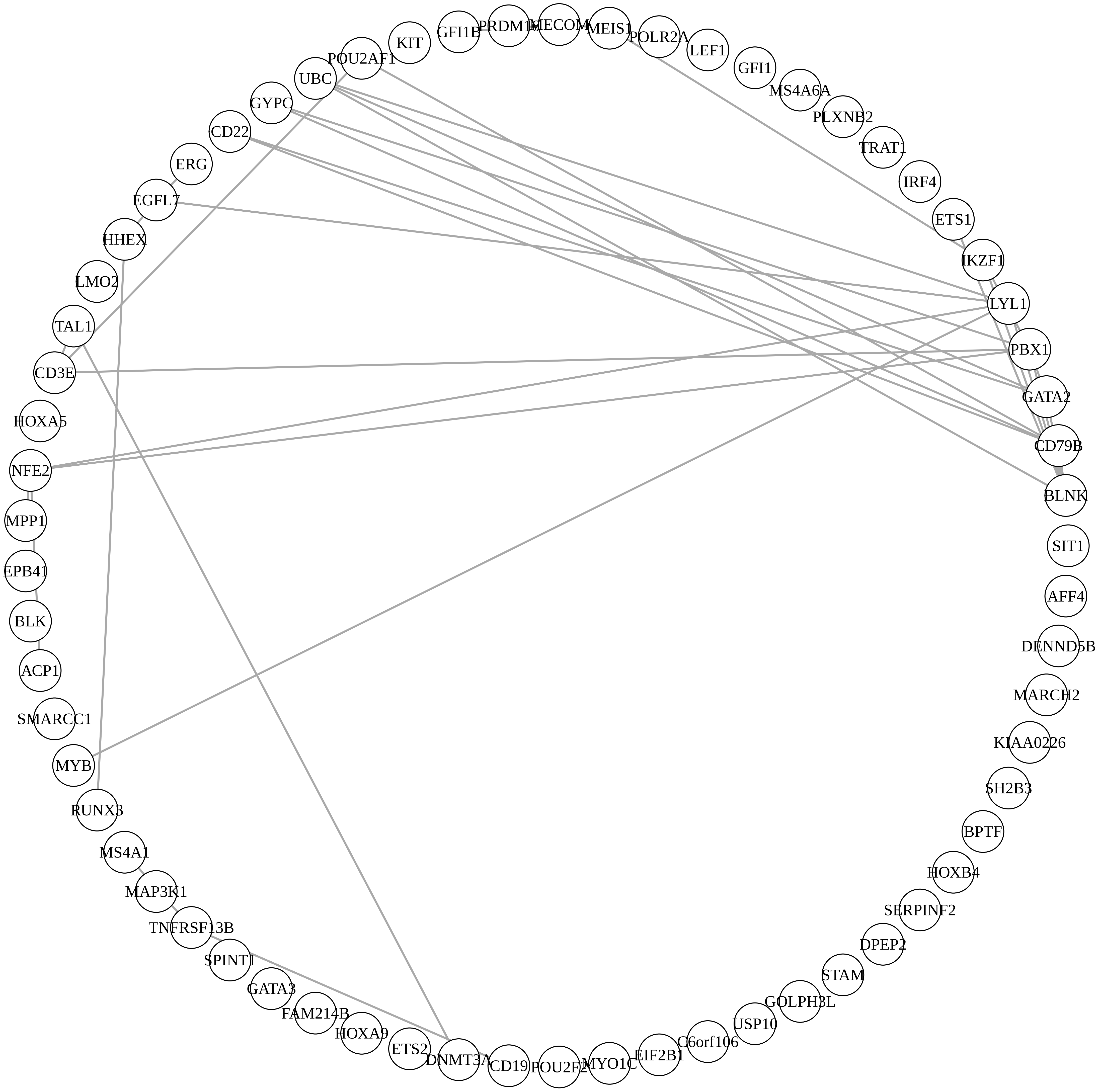

human_HSCtoLMPP_time 5

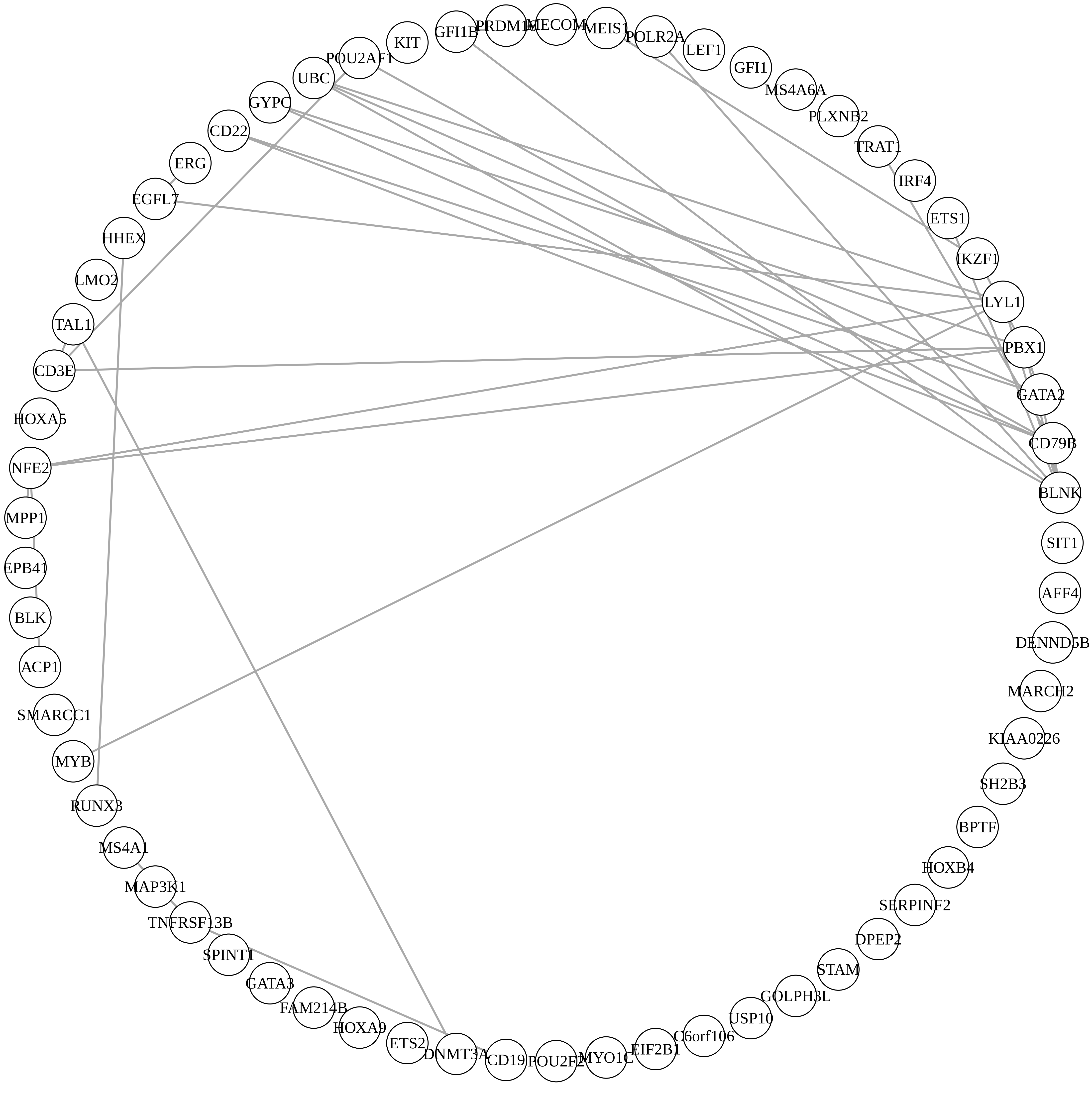

human_HSCtoLMPP_time 6

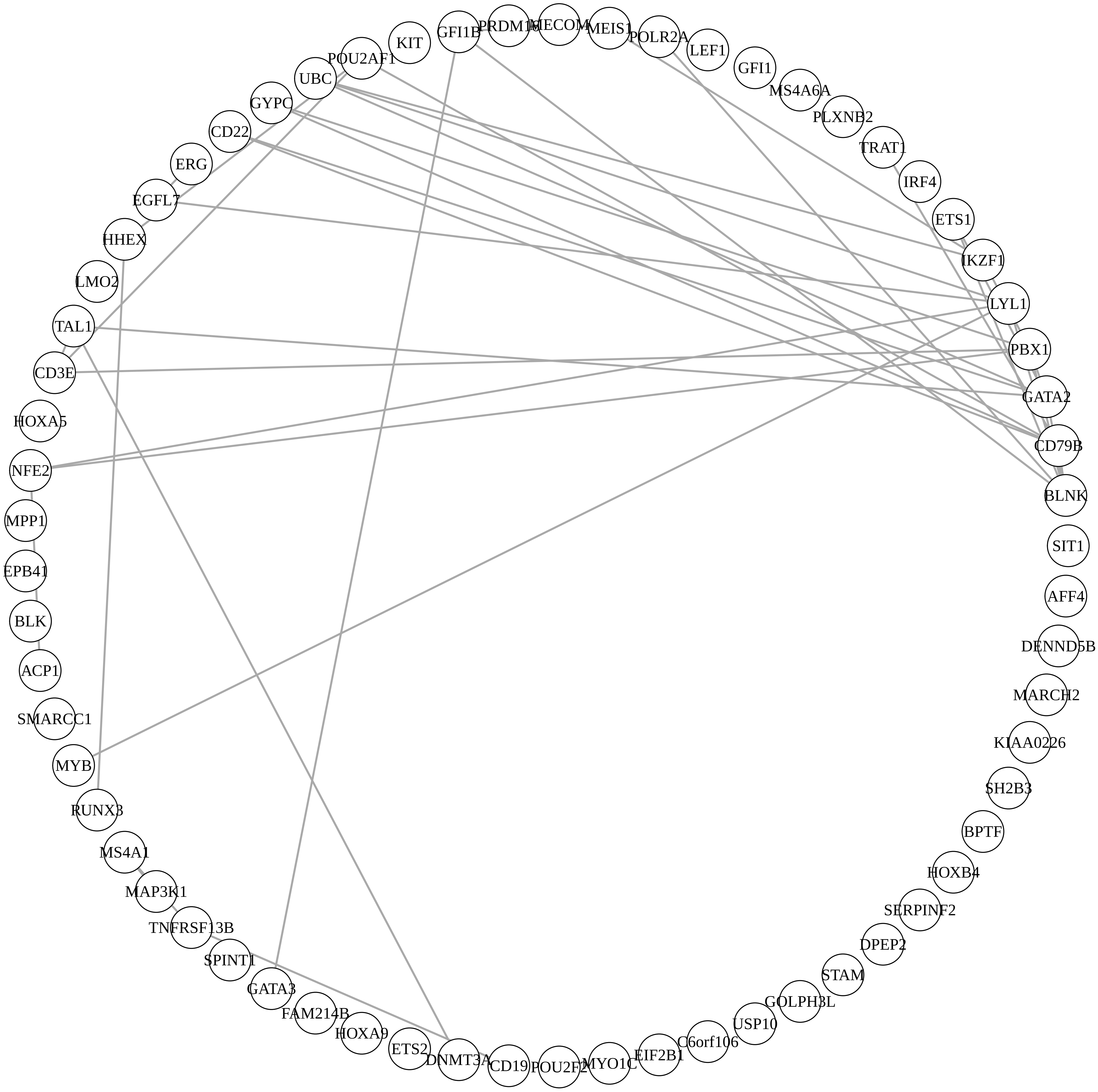

human_HSCtoLMPP_time 7

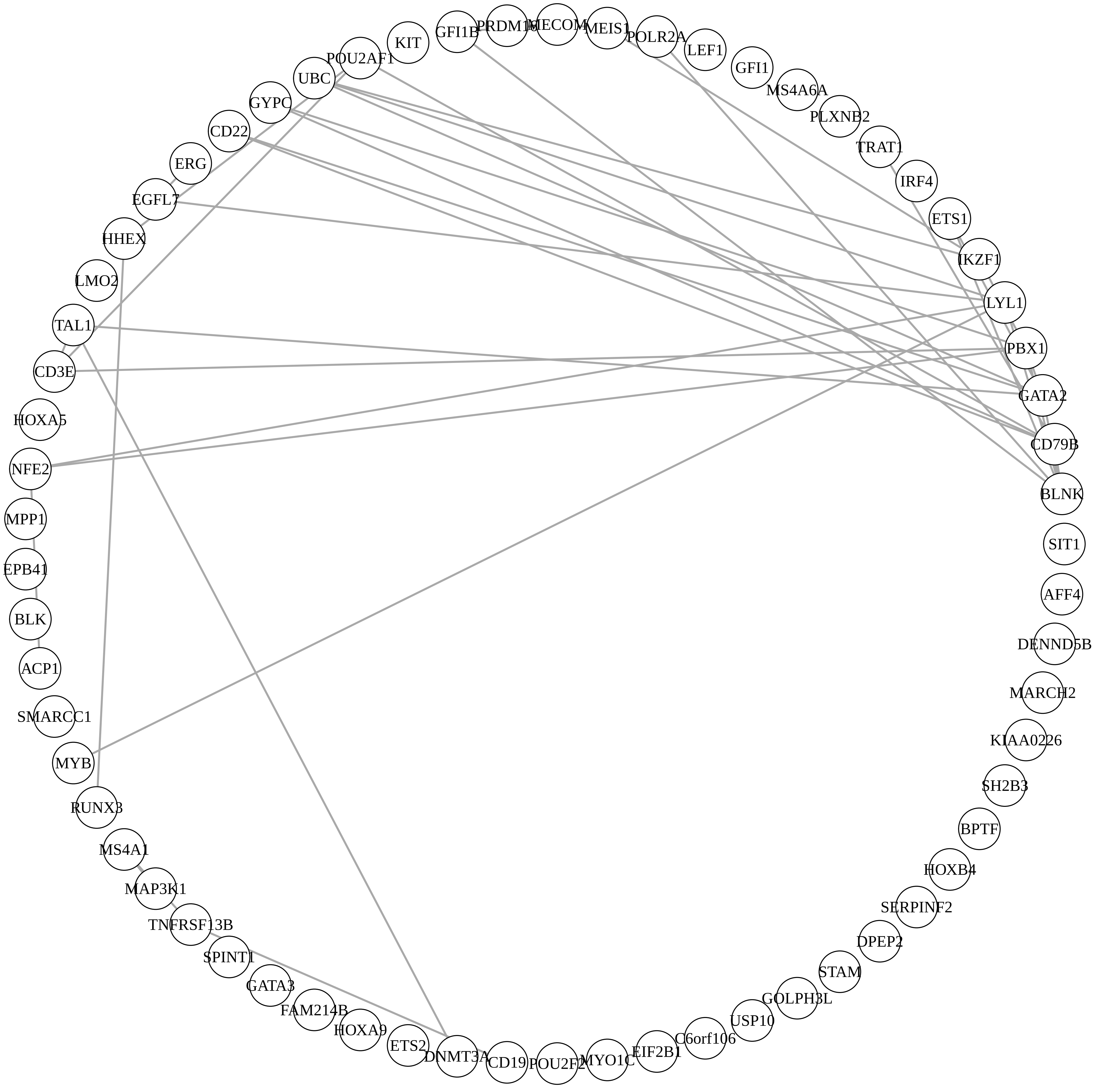

human_HSCtoLMPP_time 8

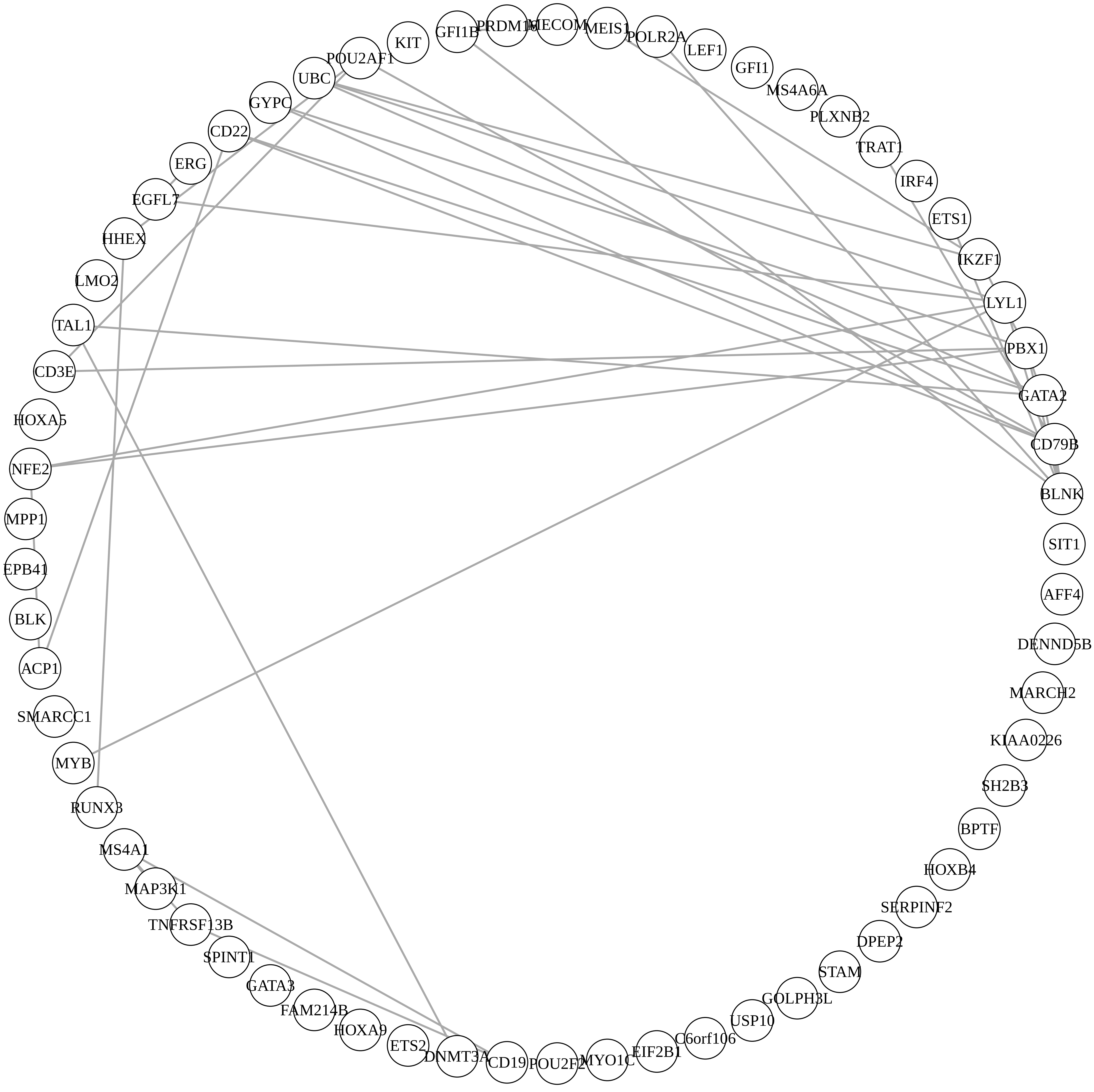

human_HSCtoLMPP_time 9

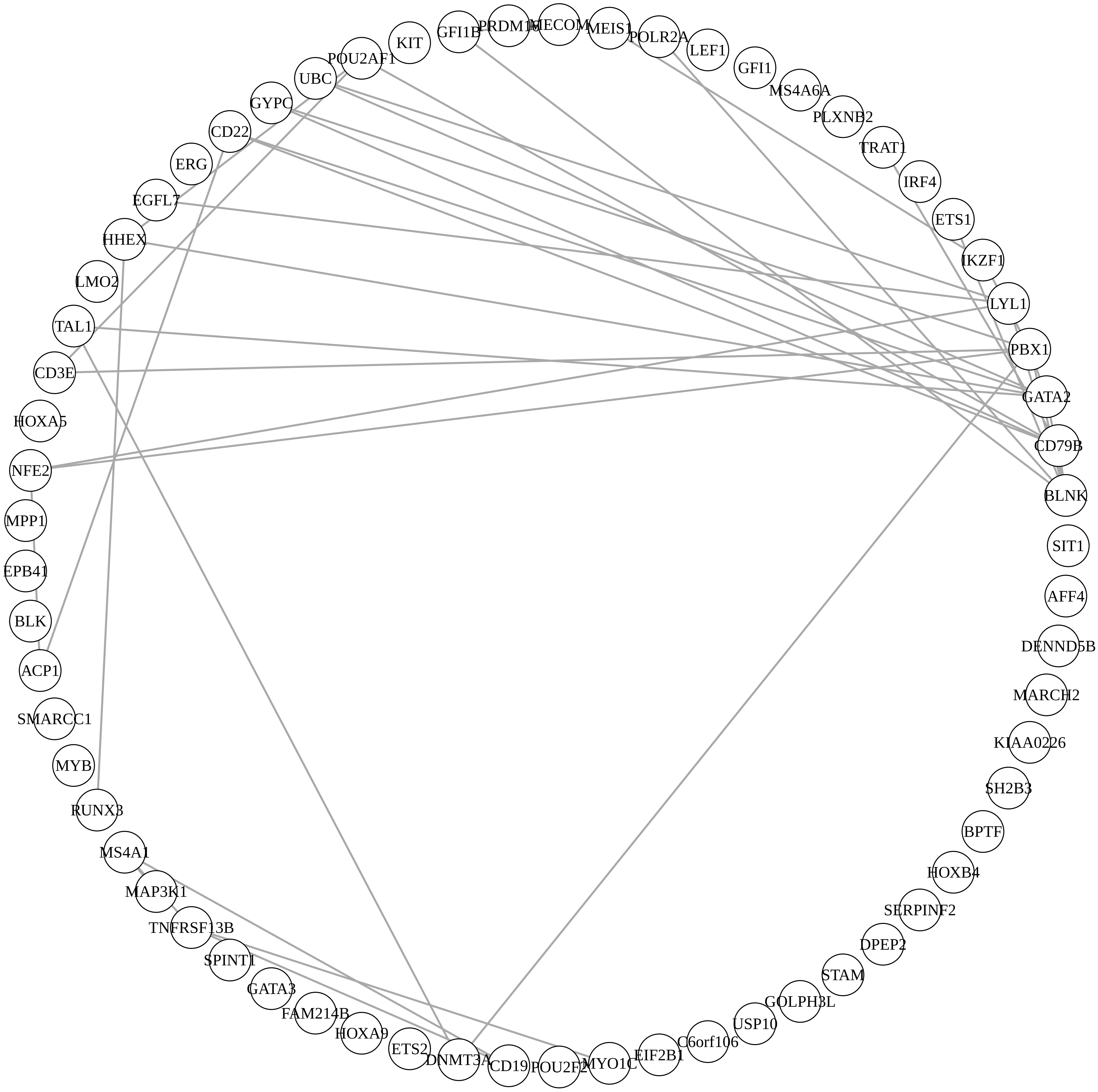

human_HSCtoLMPP_time 10

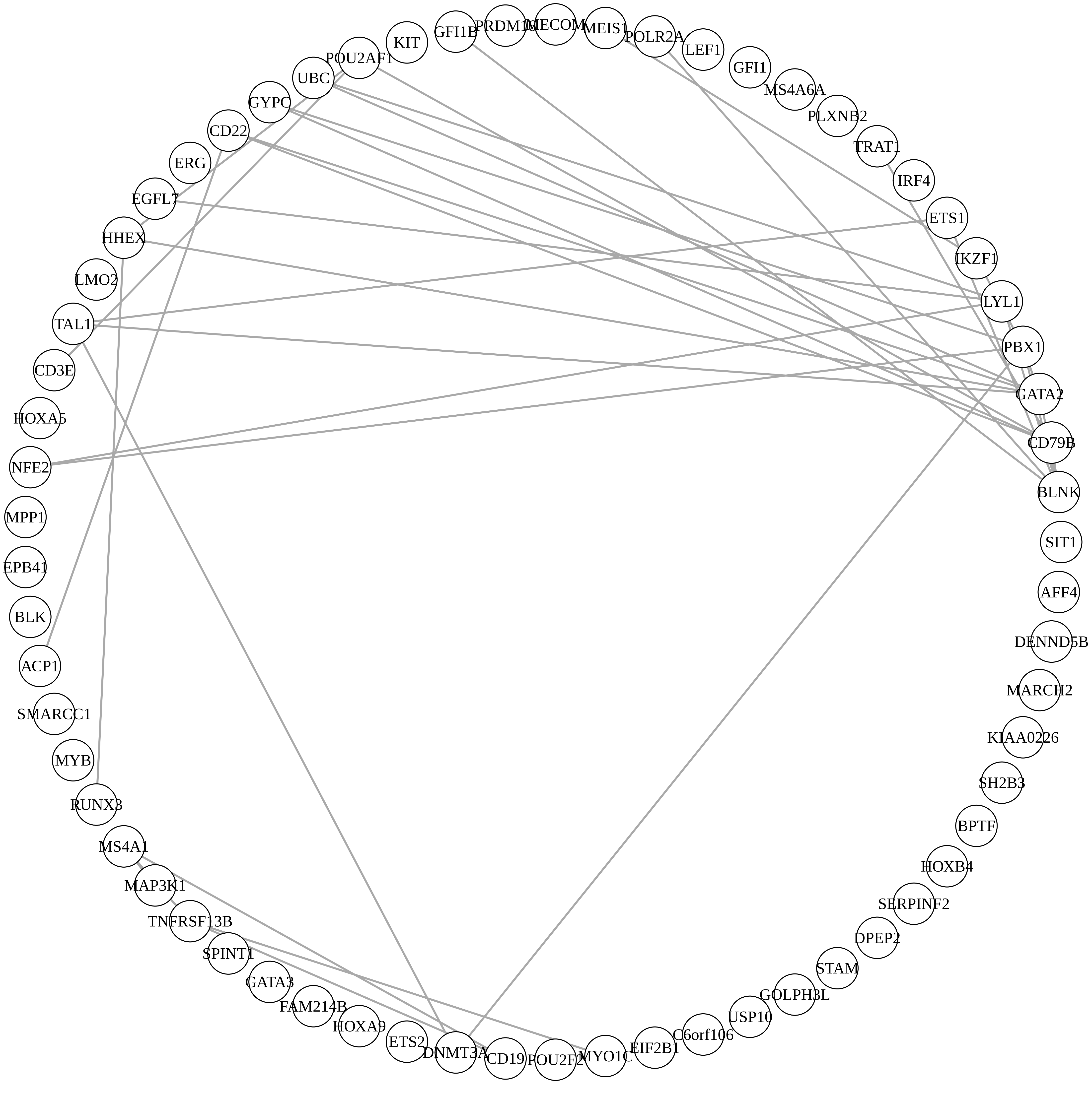

human_HSCtoLMPP_time 11

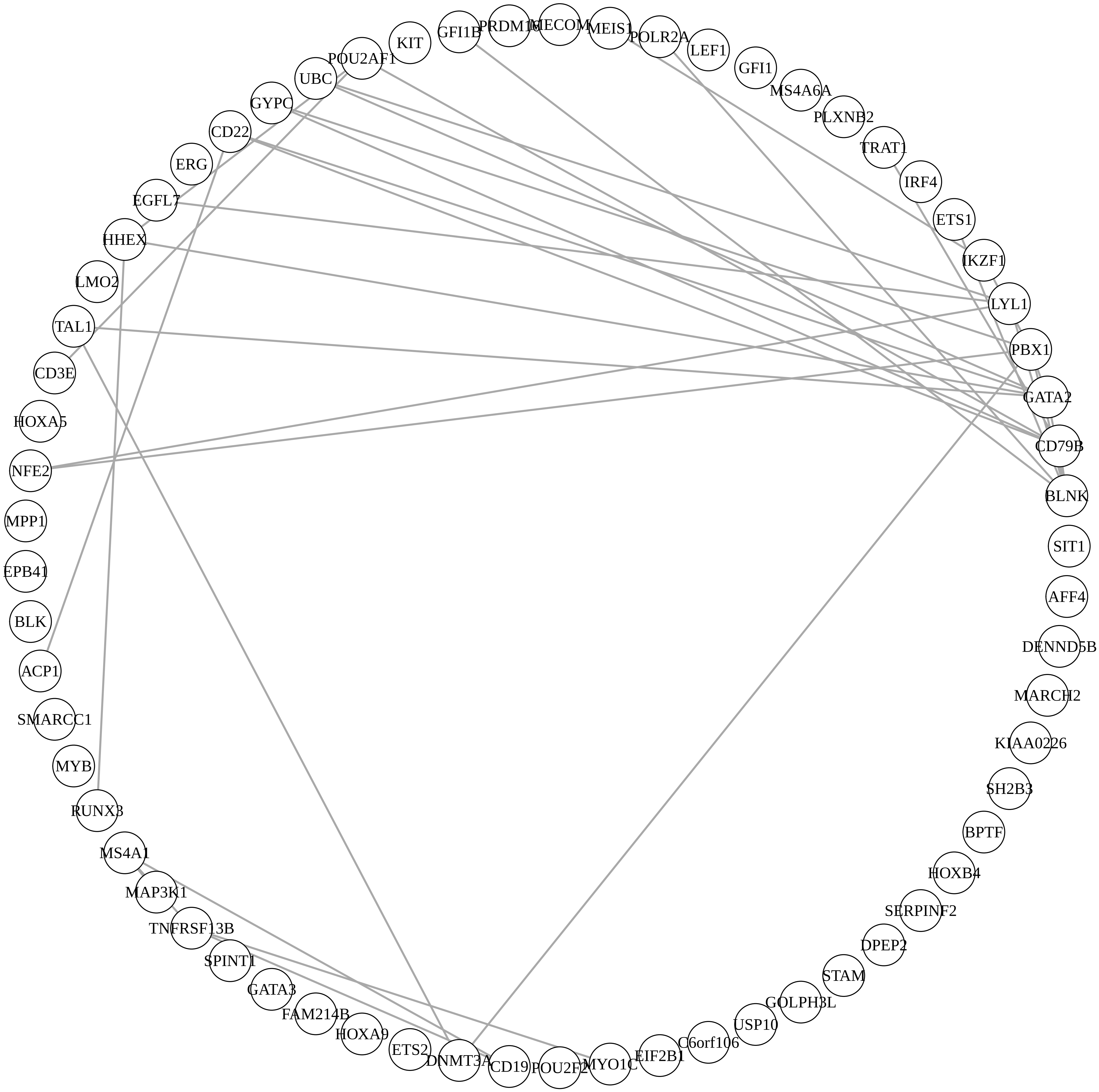

human_HSCtoLMPP_time 12

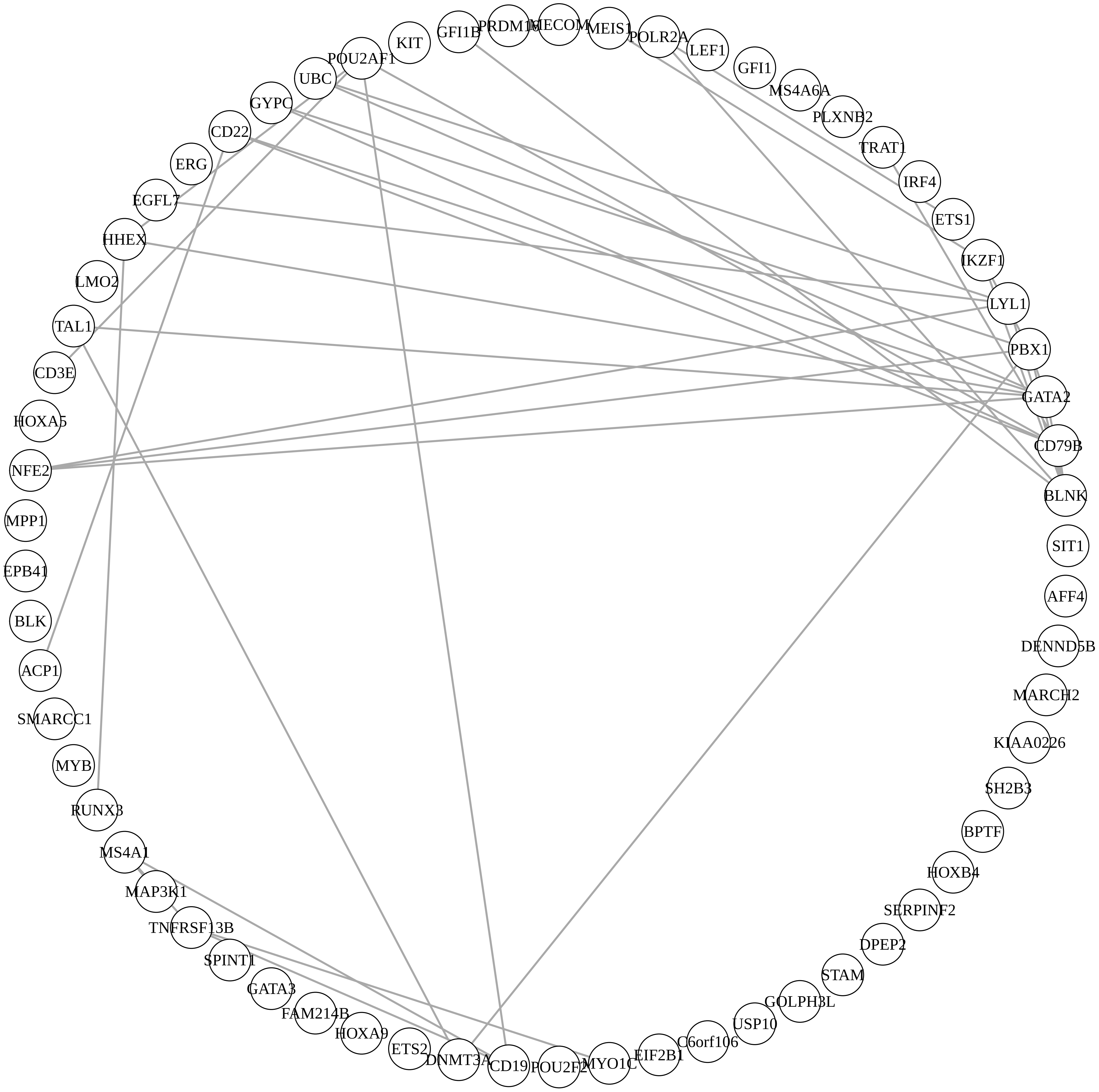

human_HSCtoLMPP_time 13

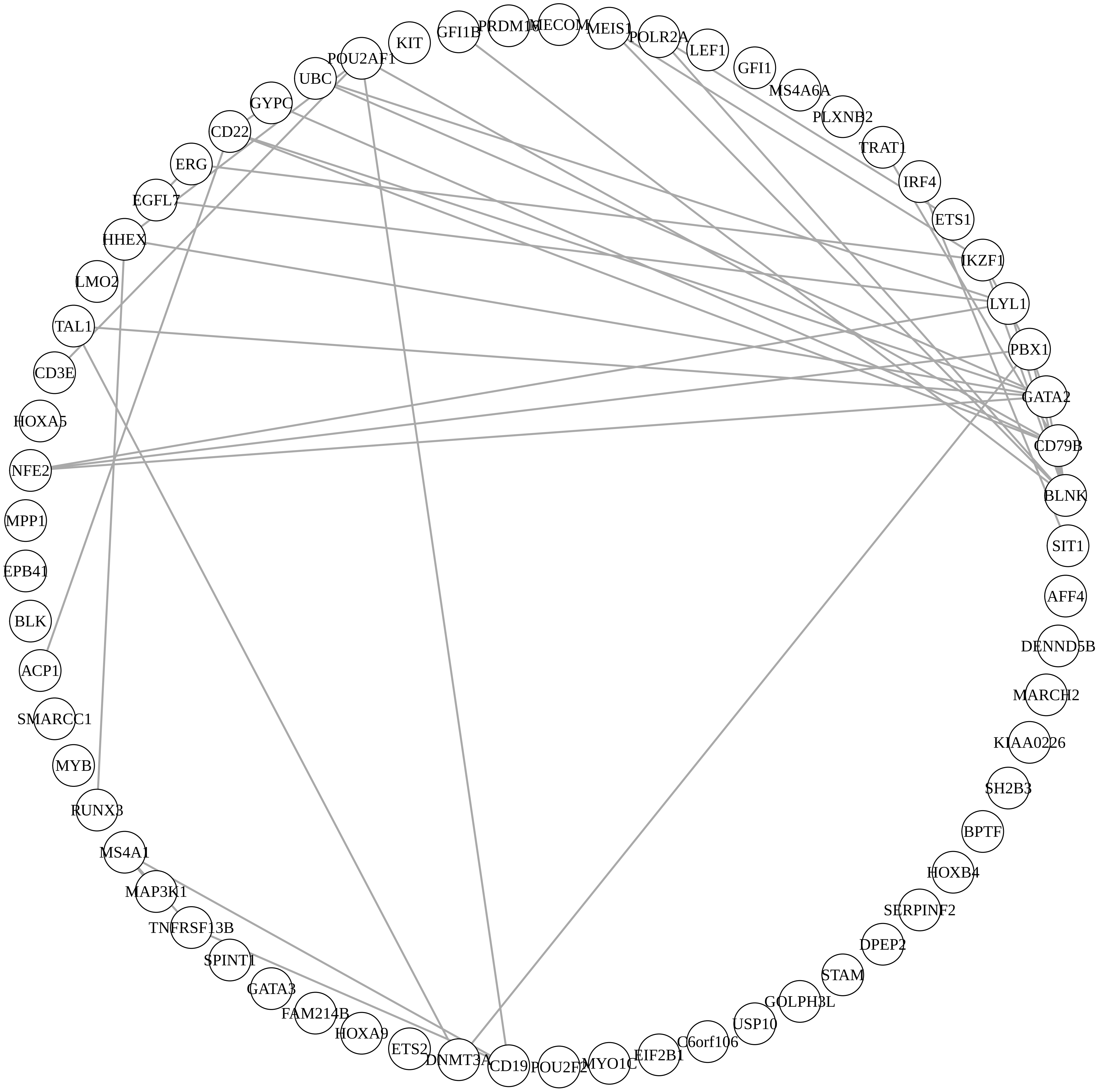

human_HSCtoLMPP_time 14

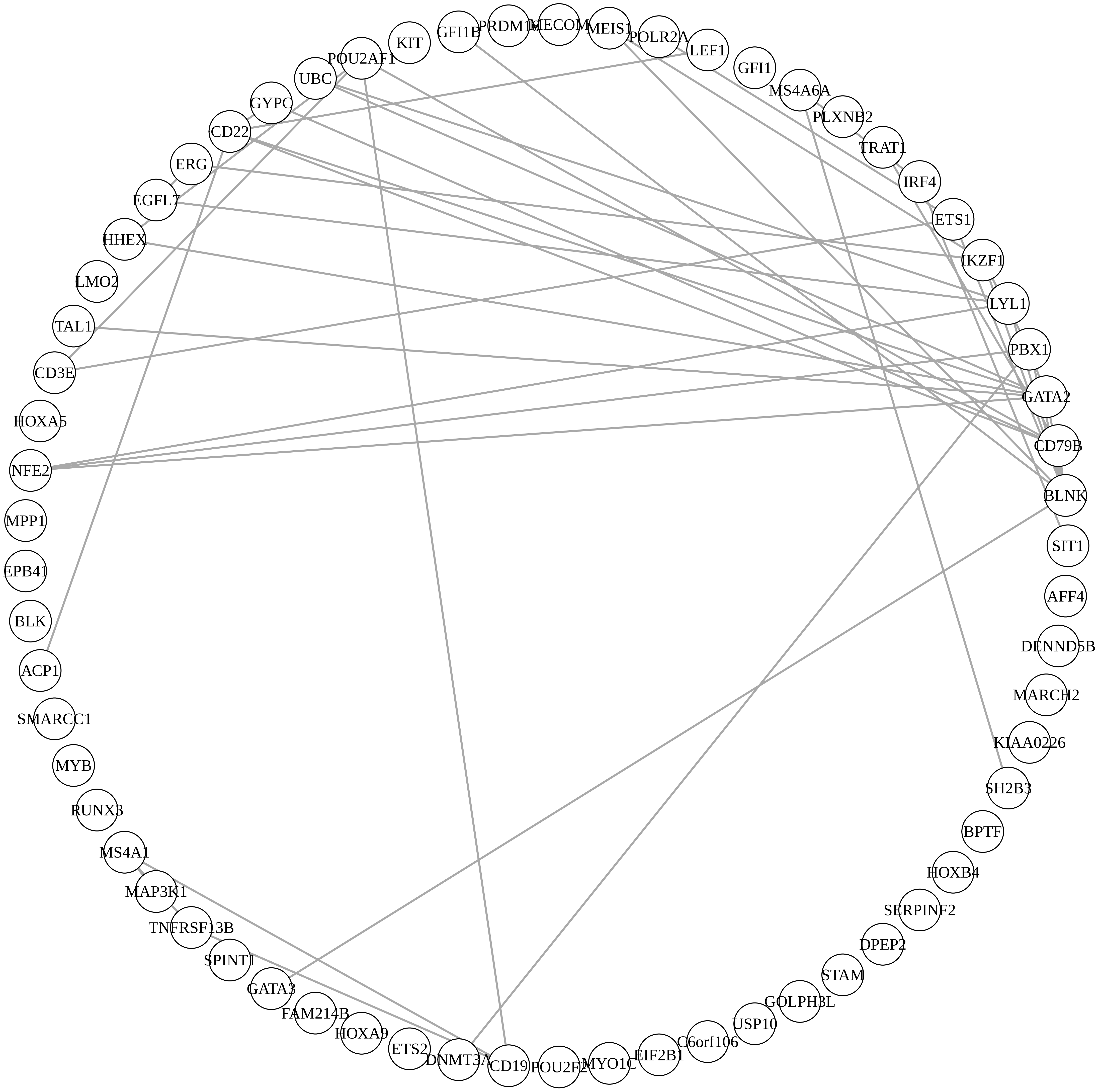

human_HSCtoLMPP_time 15

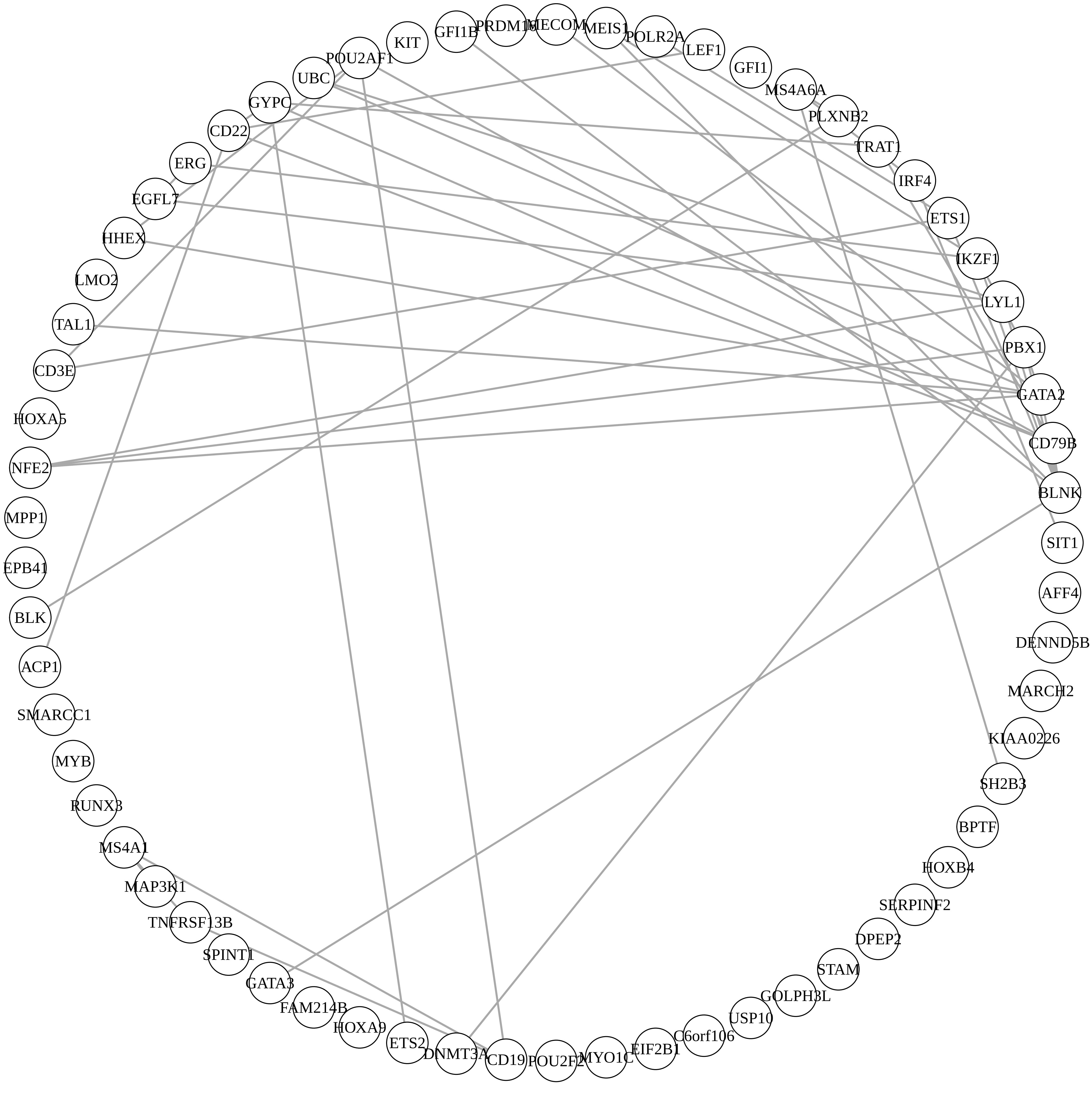

human_HSCtoLMPP_time 16

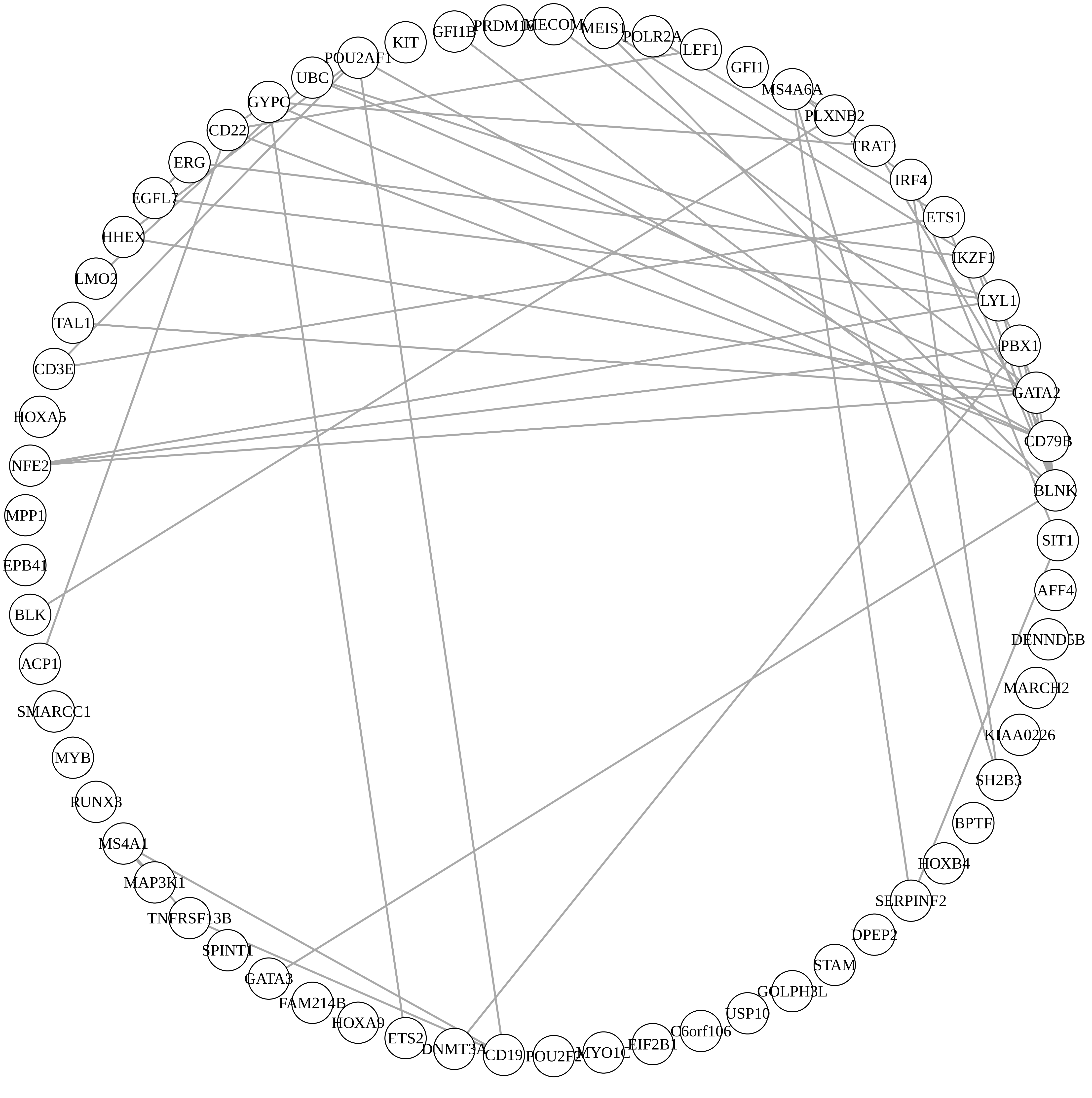

human_HSCtoLMPP_time 17

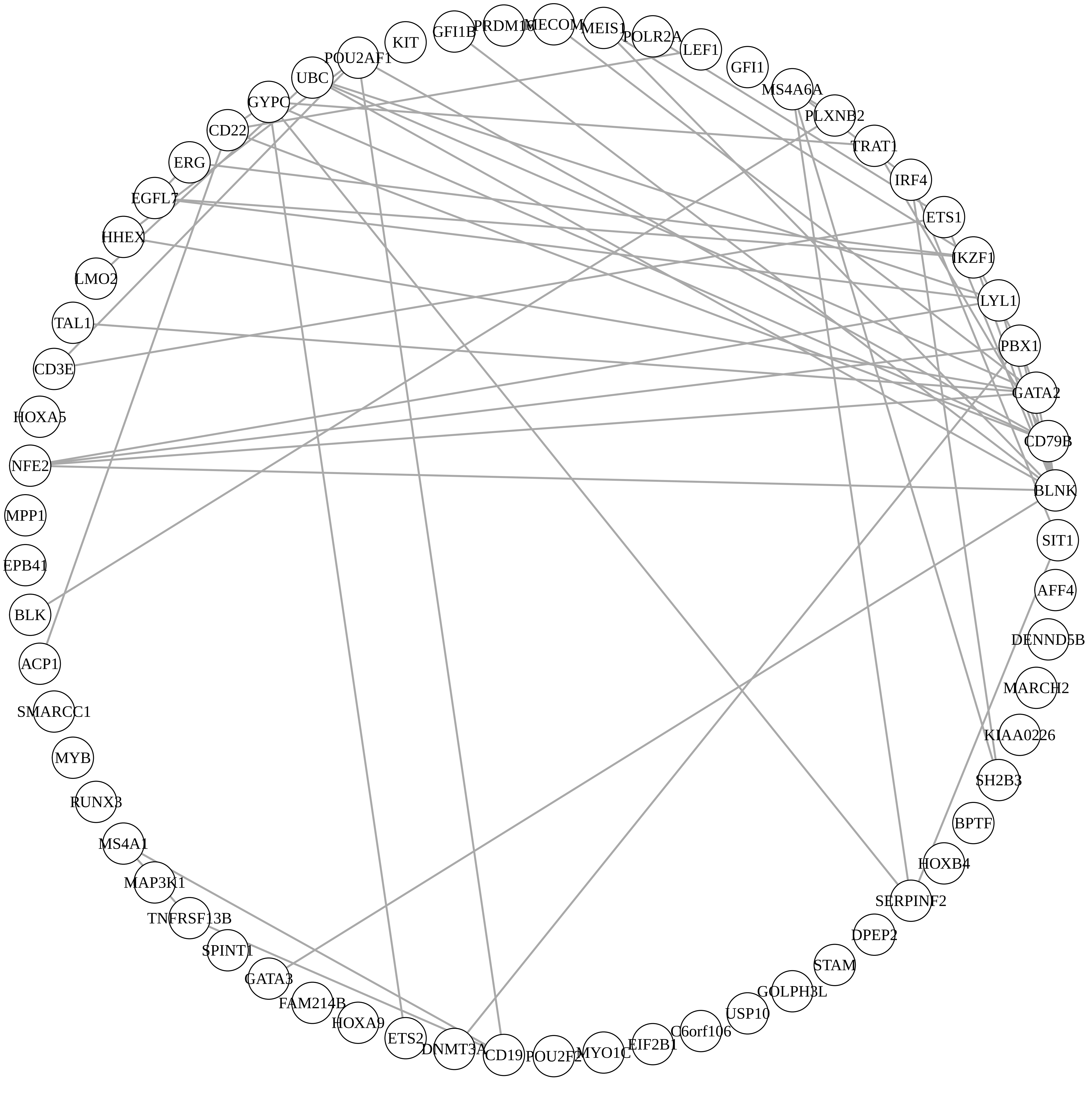

human_HSCtoLMPP_time 18

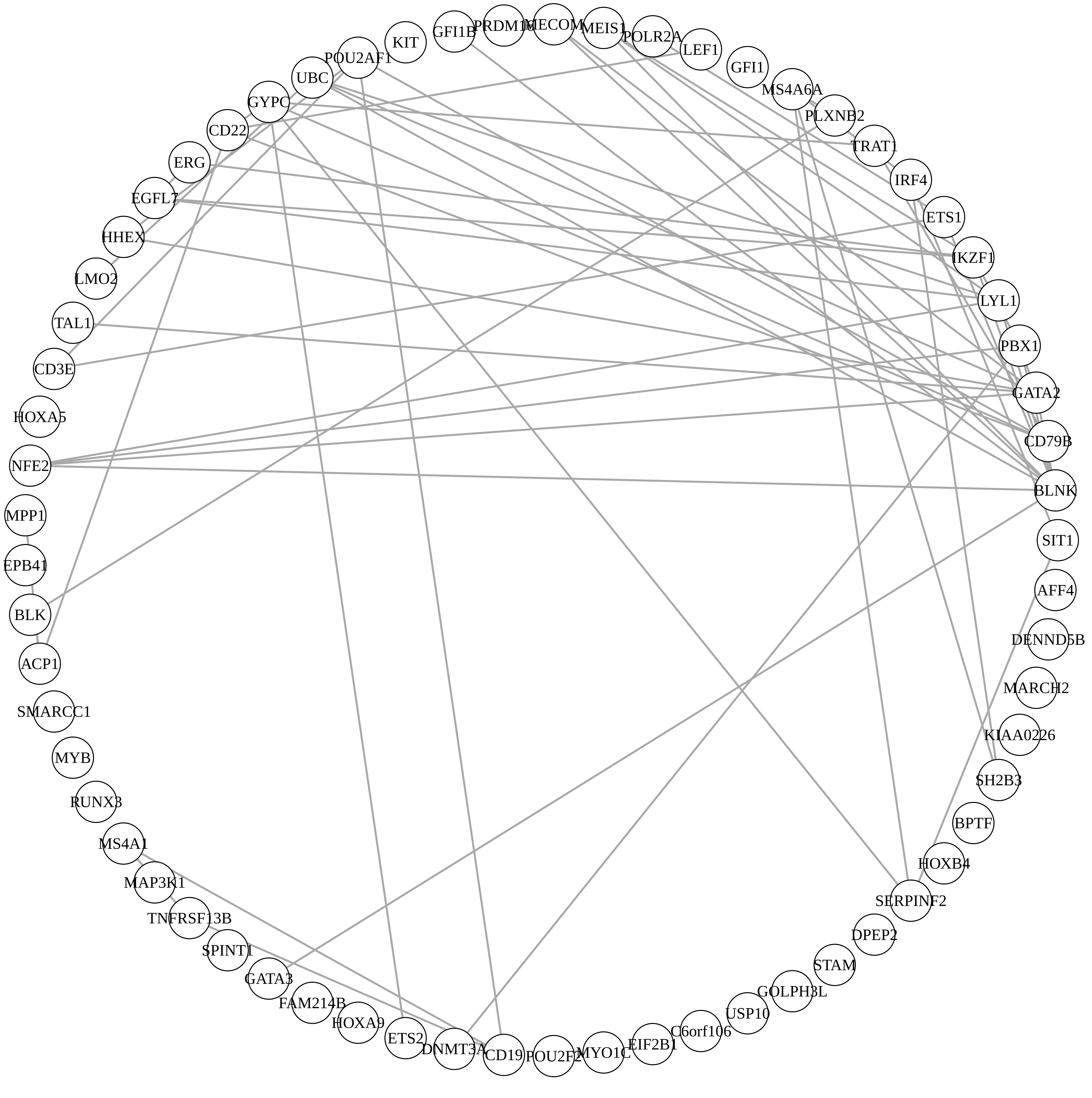

human_HSCtoLMPP_time 19

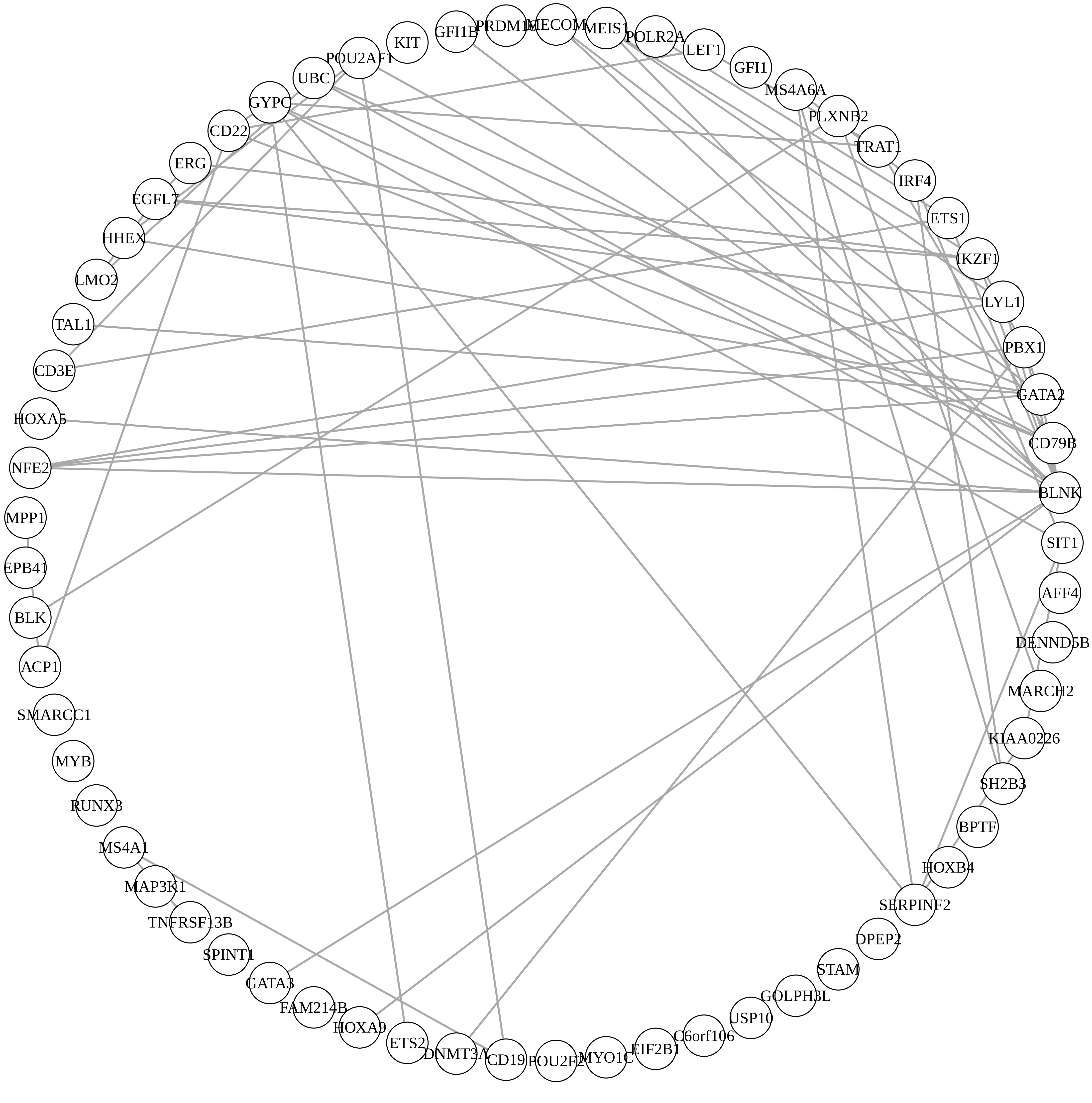

human_HSCtoLMPP_time 20

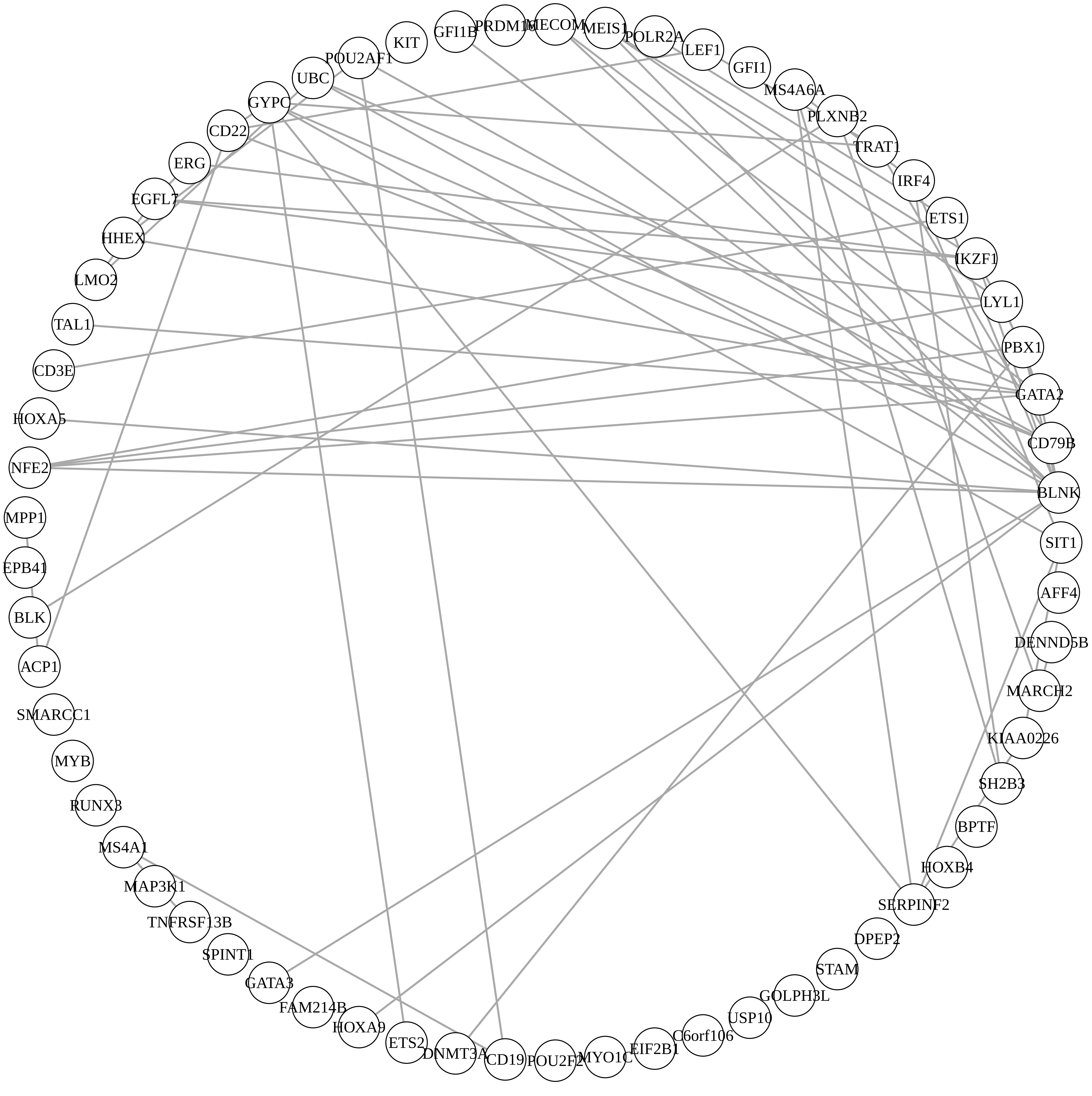

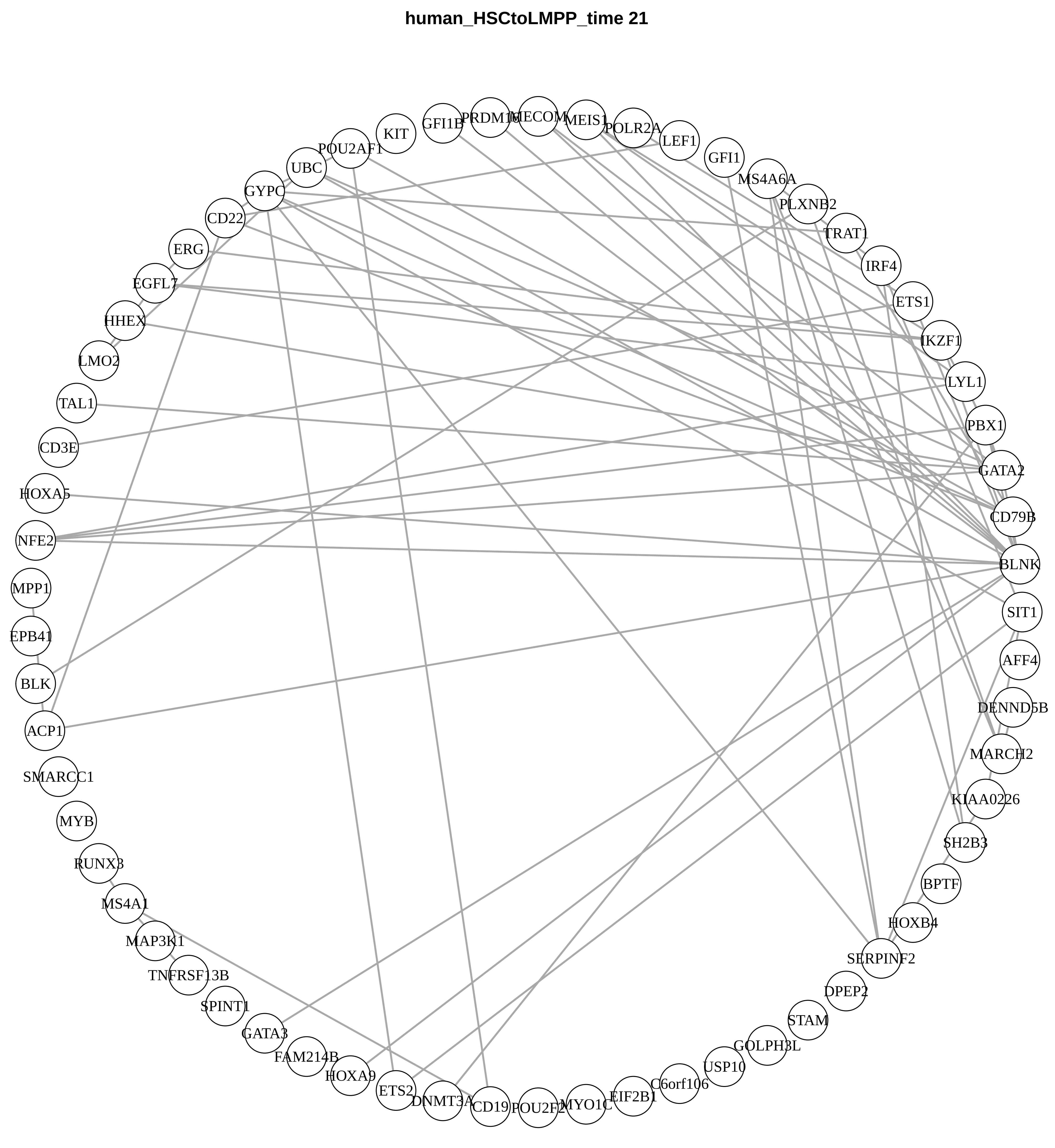
human_HSCtoLMPP_time 21
KIT
GFI1B
PRDM16
MECOM
MEIS1
POLR2A
LEF1
GFI1
MS4A6A
PLXNB2
TRAT1
IRF4
ETS1
IKZF1
LYL1
PBX1
GATA2
CD79B
BLNK
SIT1
AFF4
DENND5B
MARCH2
KIAA0226
SH2B3
BPTF
HOXB4
SERPINF2
DPEP2
STAM
GOLPH3L
USP10
C6orf106
EIF2B1
MYO1C
POU2F2
CD19
DNMT3A
ETS2
HOXA9
FAM214B
GATA3
SPINT1
TNFRSF13B
MAP3K1
MS4A1
RUNX3
MYB
SMARCC1
ACP1
BLK
EPB41
MPP1
NFE2
HOXA5
CD3E
TAL1
LMO2
HHEX
EGFL7
ERG
CD22
GYPC
UBC
POU2AF1

human_HSCtoLMPP_time 22

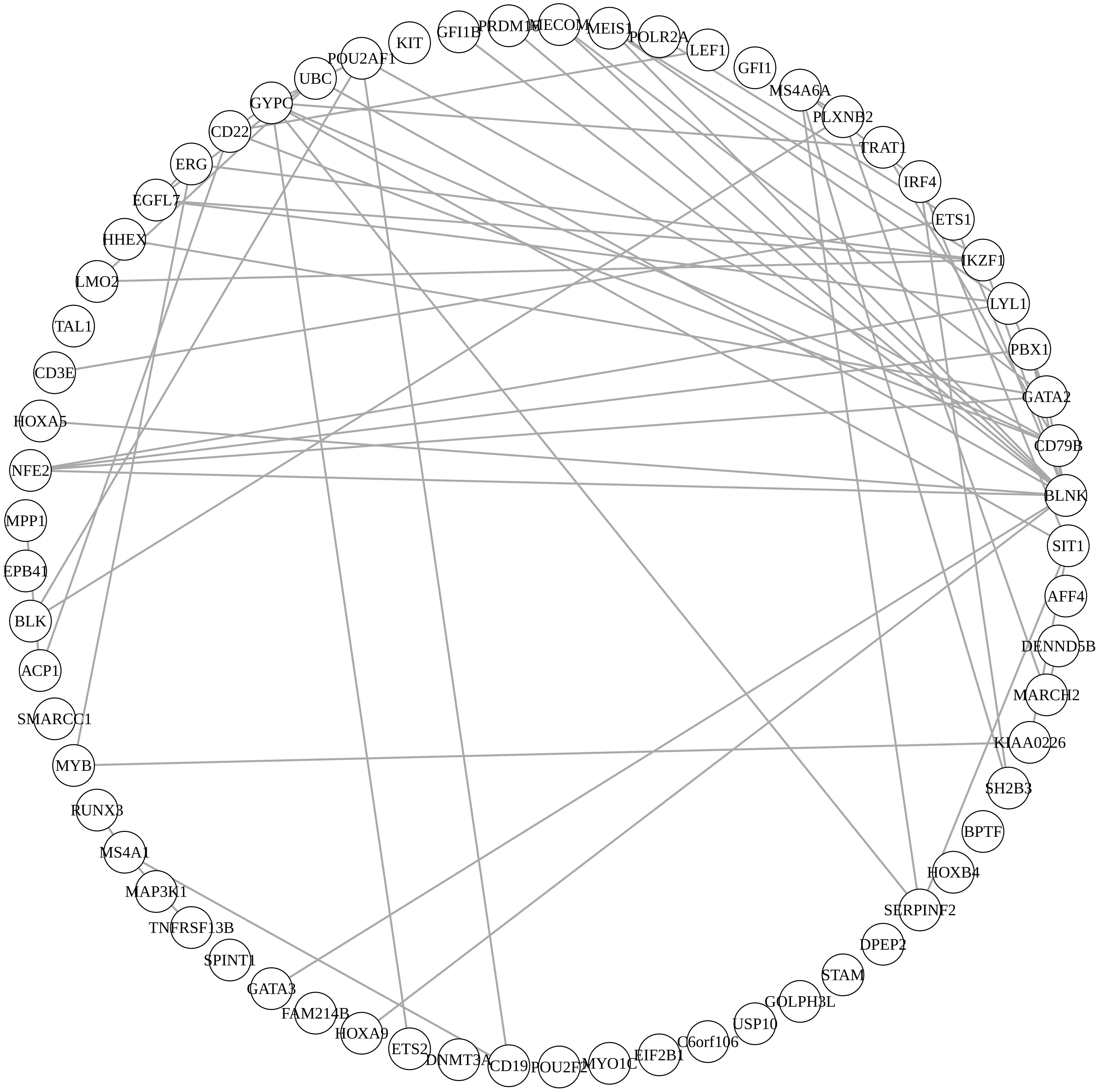

human_HSCtoLMPP_time 23

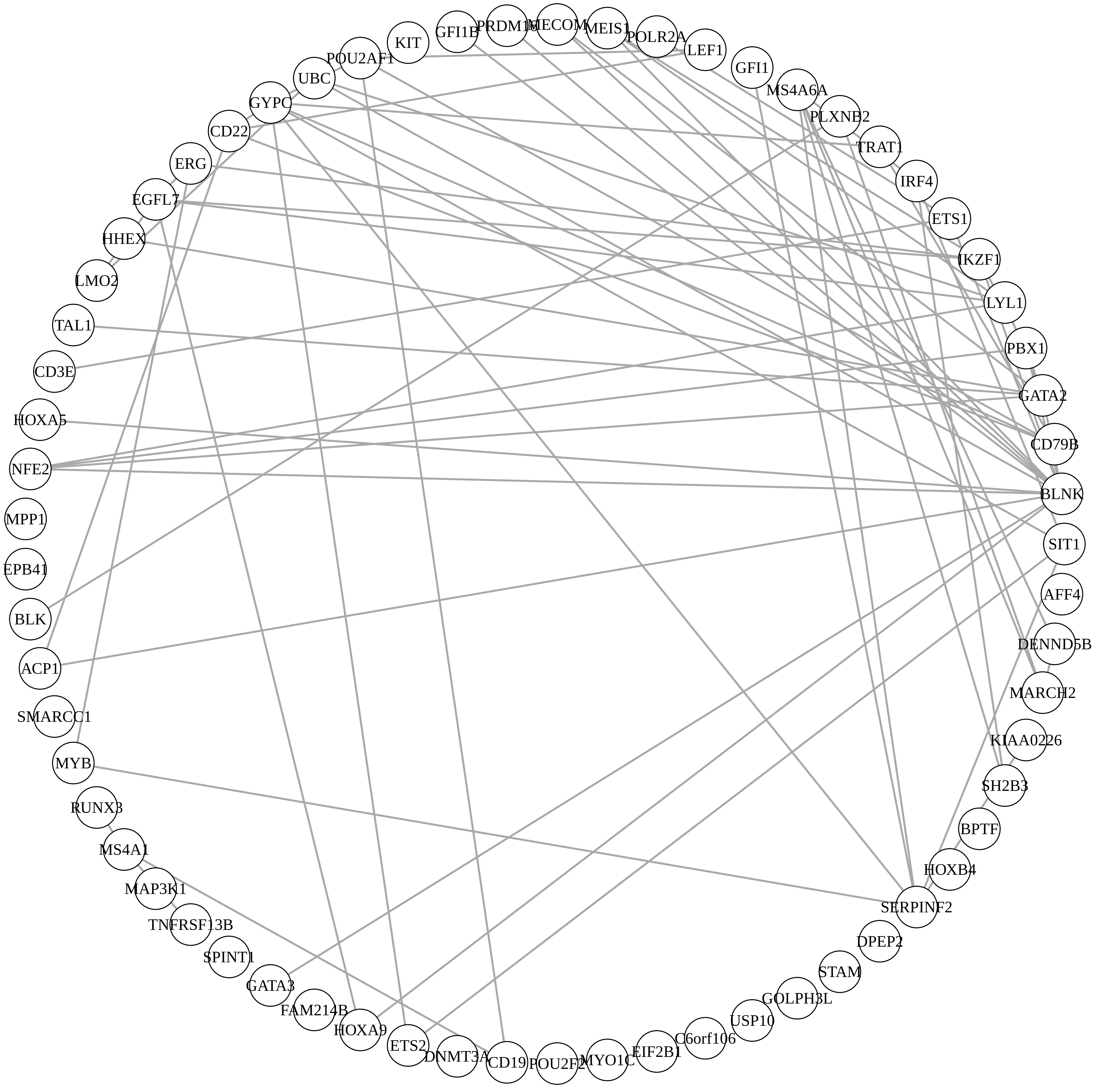

human_HSCtoLMPP_time 24

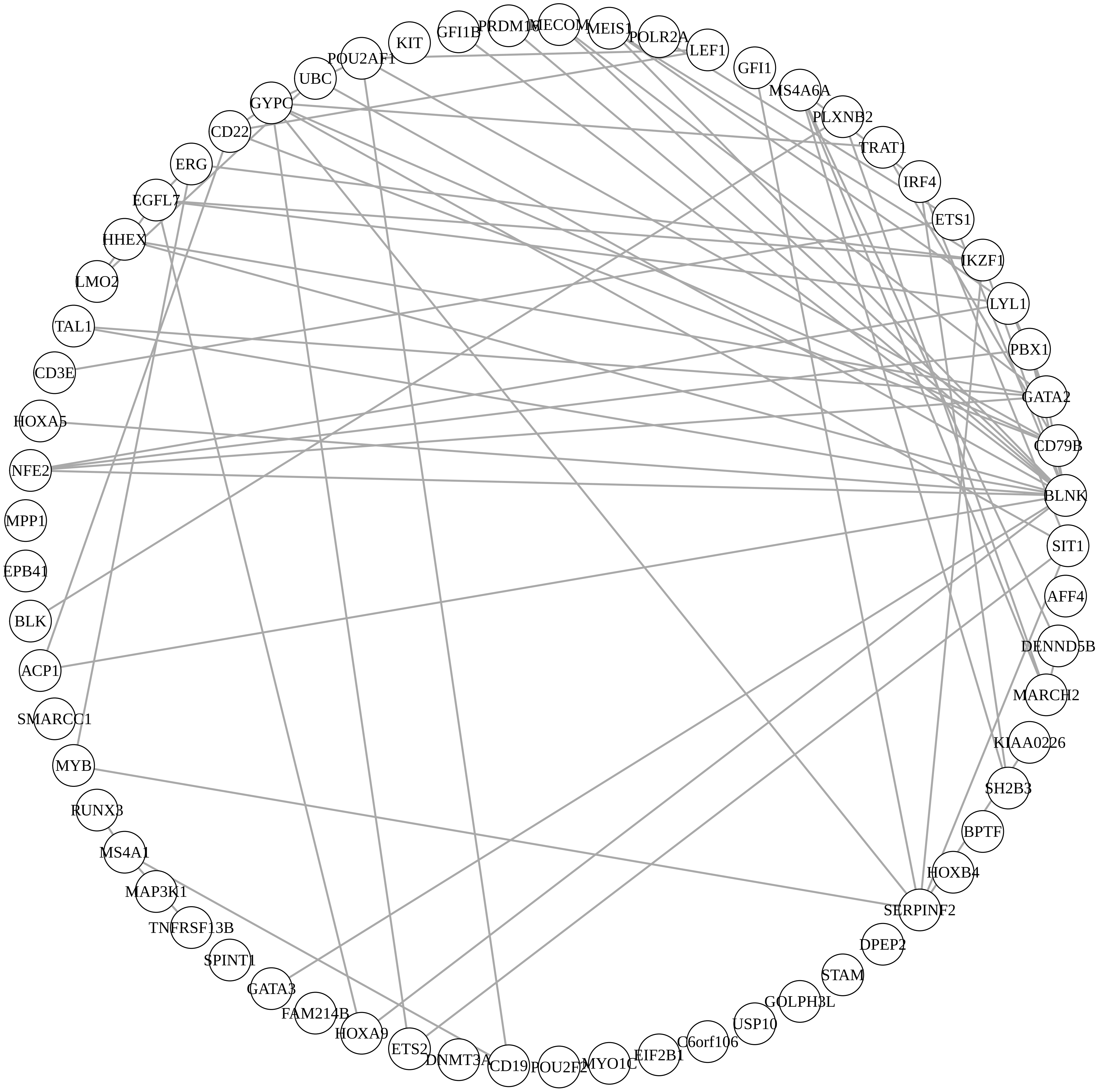

human_HSCtoLMPP_time 25

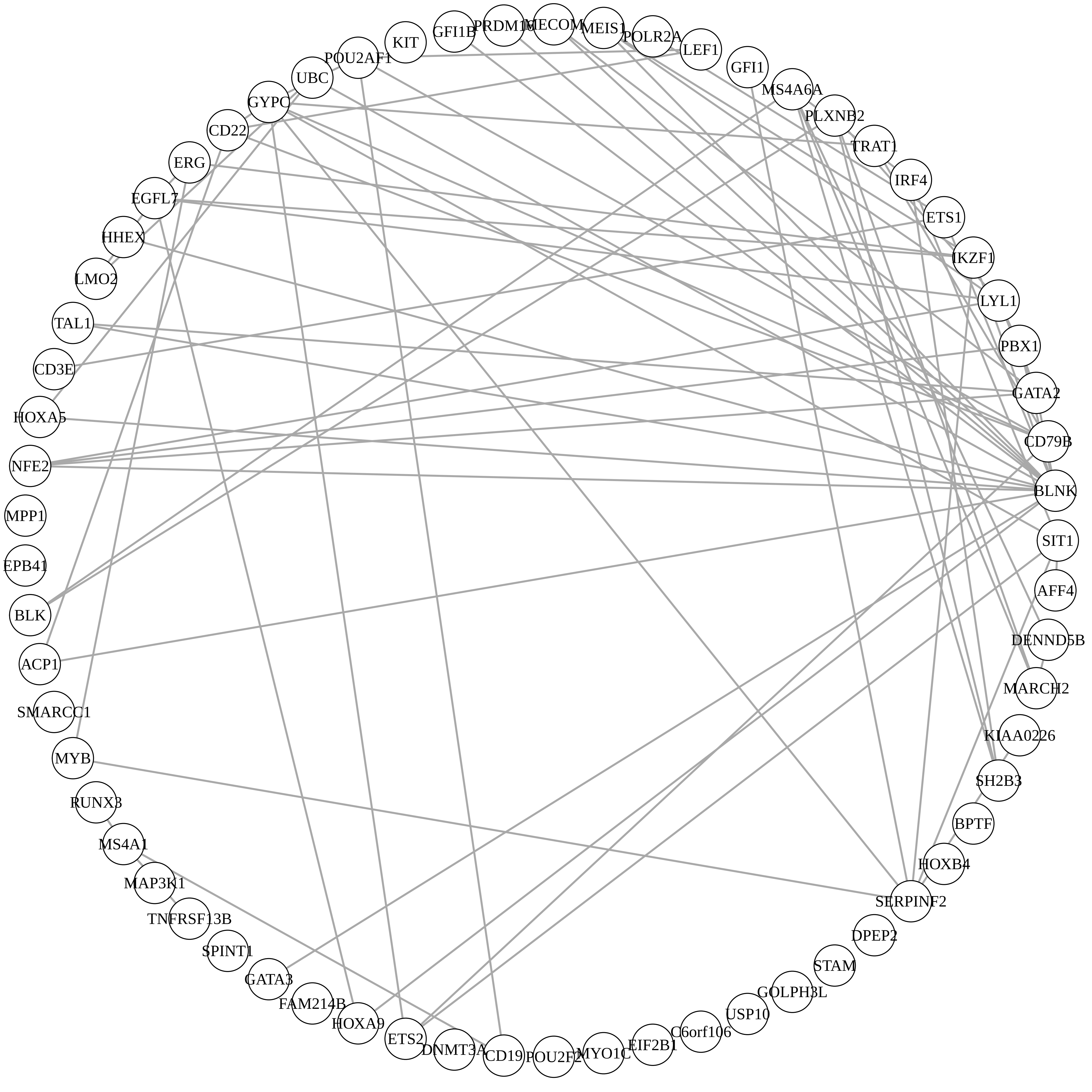

Supplement: Supplementary file 1 [file genes-13-01890-s001.zip › Supp Figure S1.pdf]
