# Supplementary material for: Time-Varying Gene Expression Network Analysis Reveals Conserved Transition States in Hematopoietic Differentiation between Human and Mouse
Source: Genes (Basel). 2022 Oct 18;13(10):1890. doi: 10.3390/genes13101890 (PMC9601530; doi:10.3390/genes13101890)

Degree distribution of networks from HSC to GMP in human

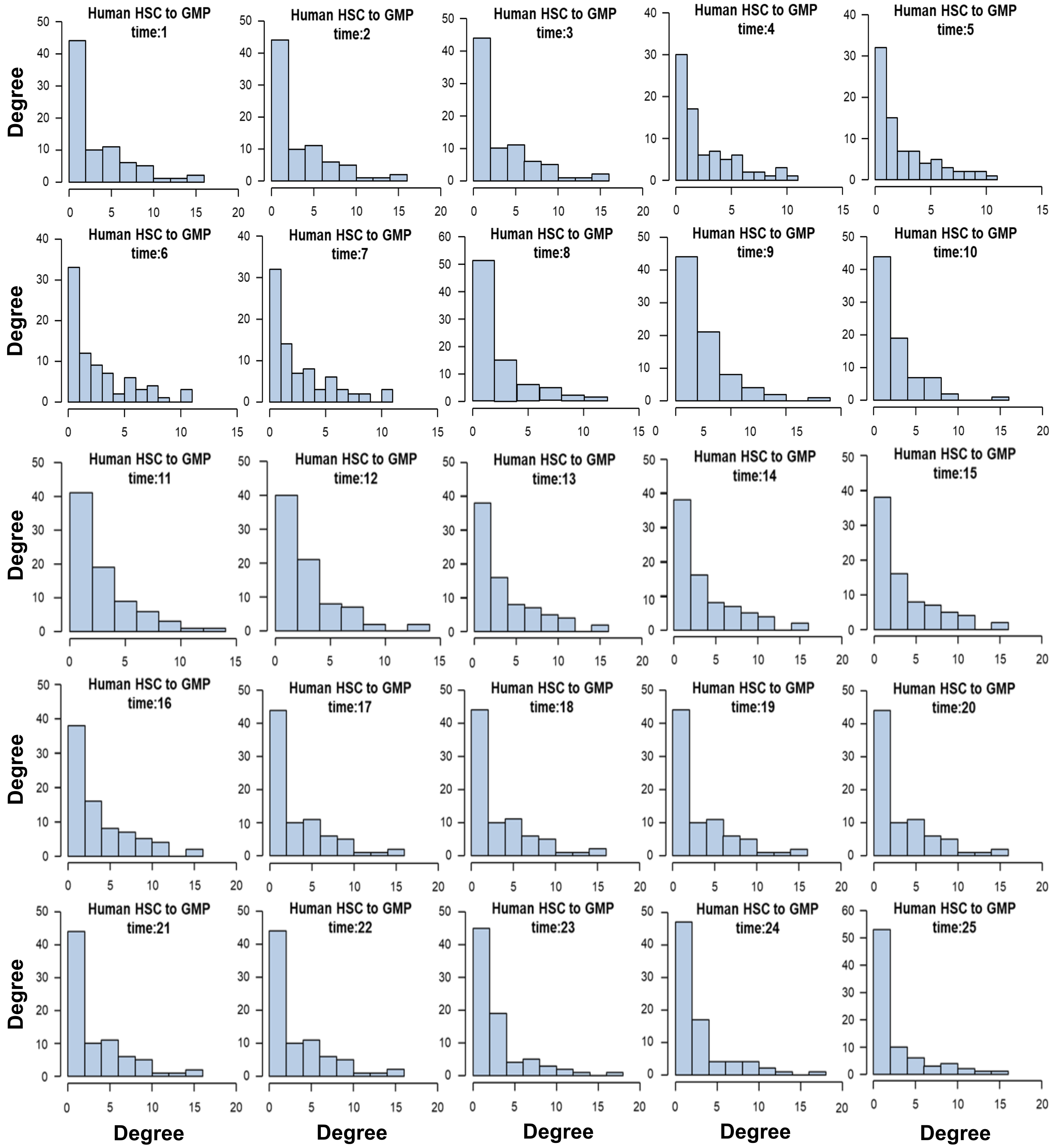

Degree distribution of networks from HSC to MEP in human

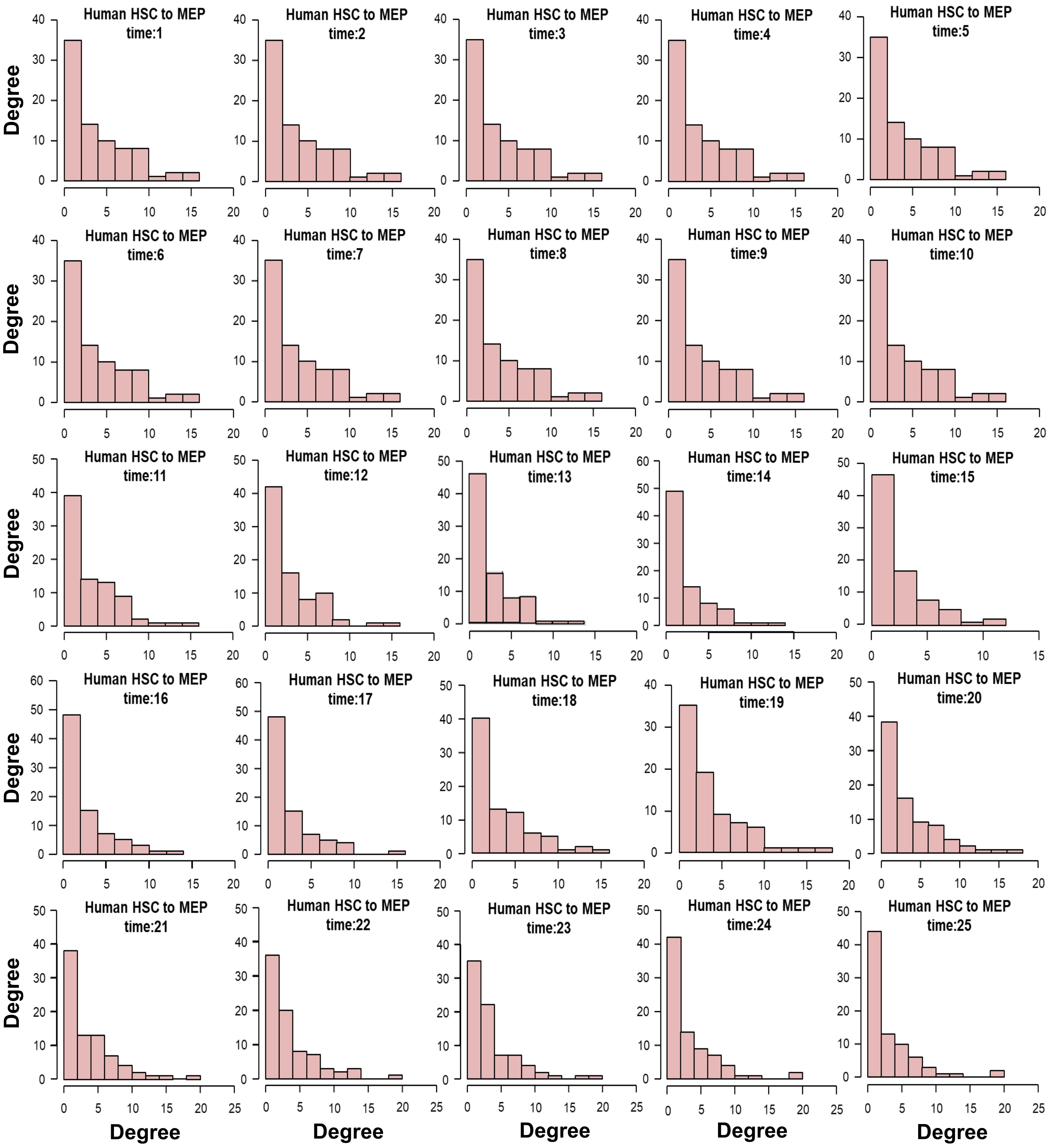

Degree distribution of networks from HSC to LMPP in human

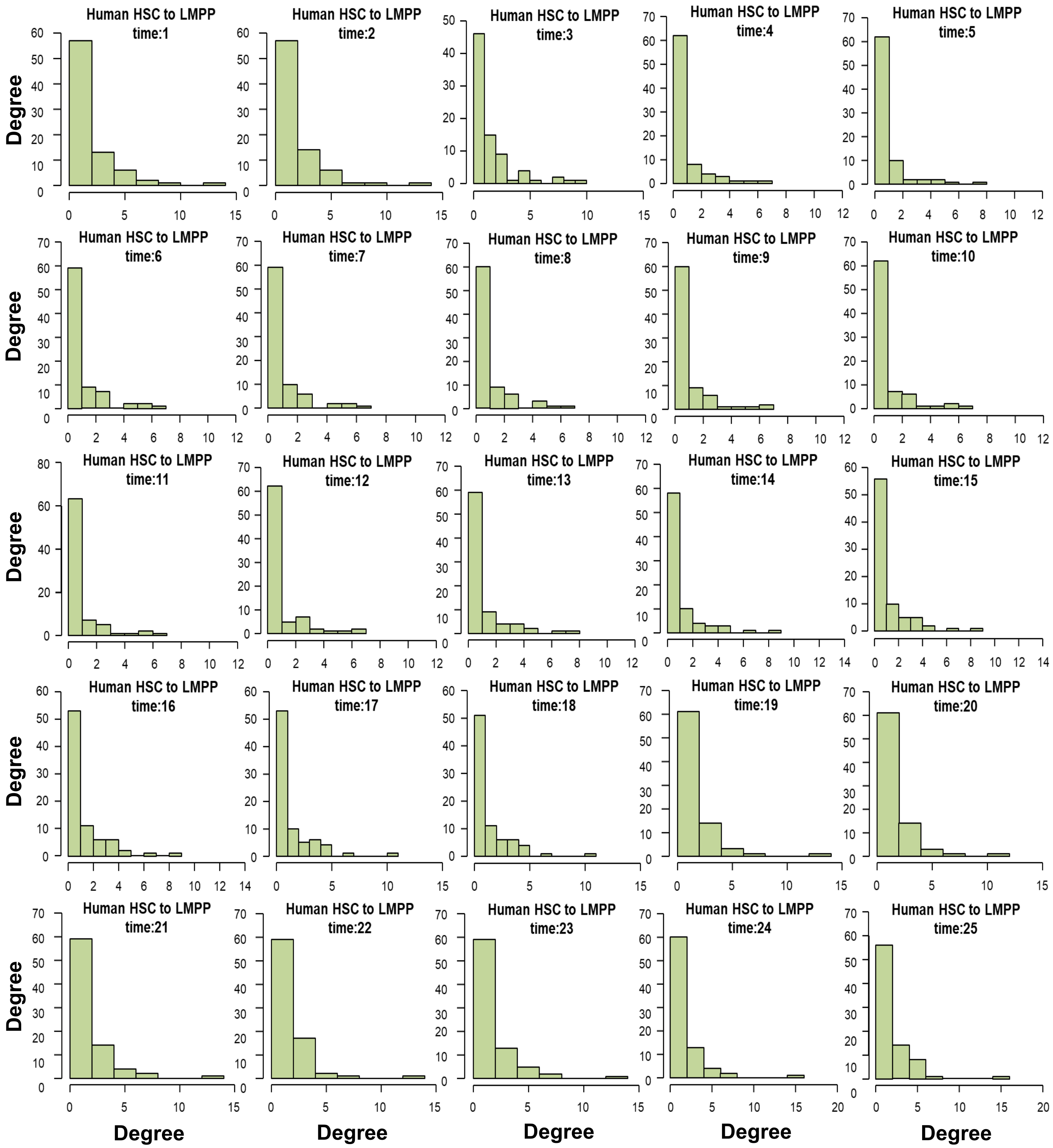

Degree distribution of networks from HSC to GMP in mouse

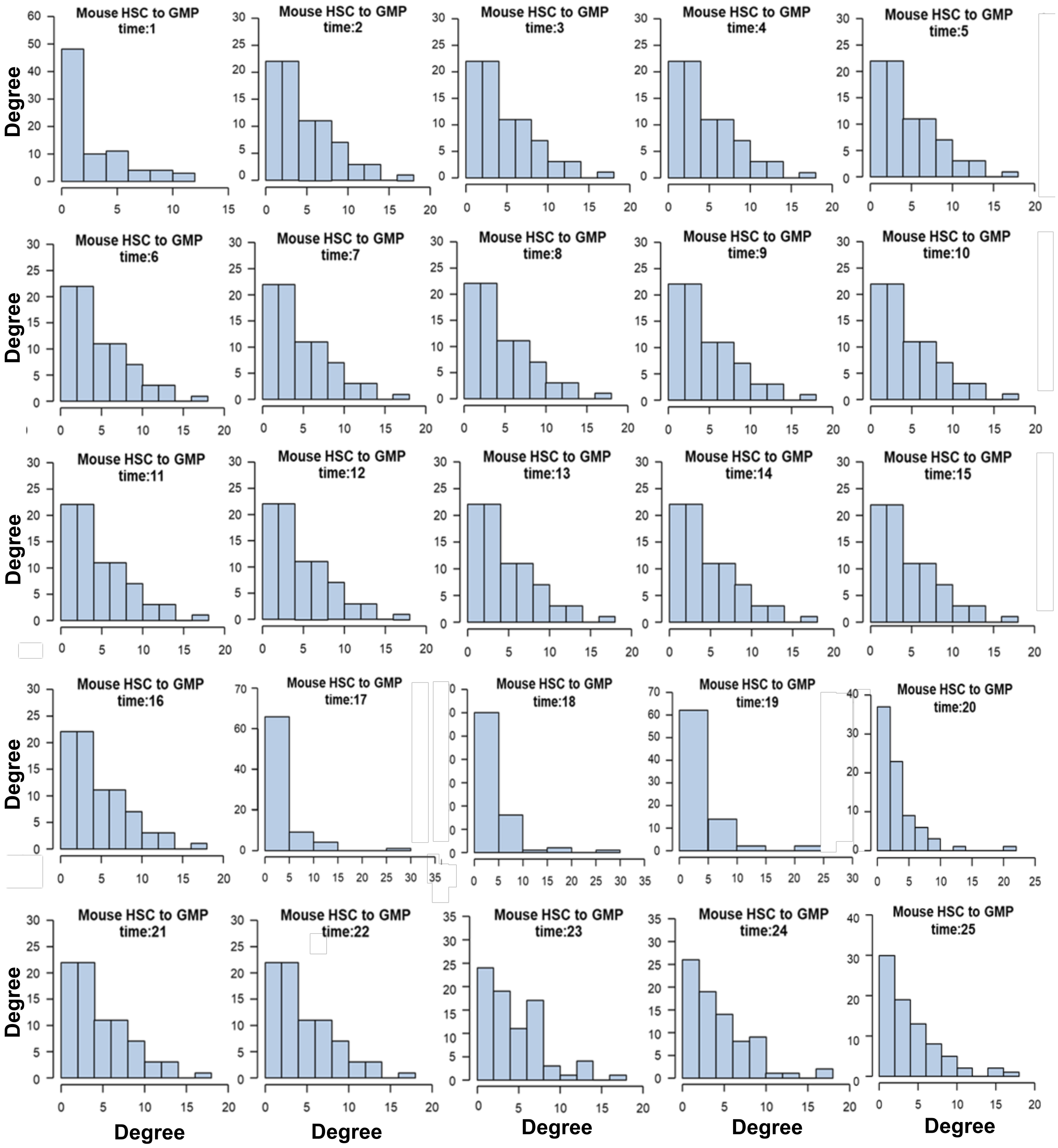

Degree distribution of networks from HSC to MEP in mouse

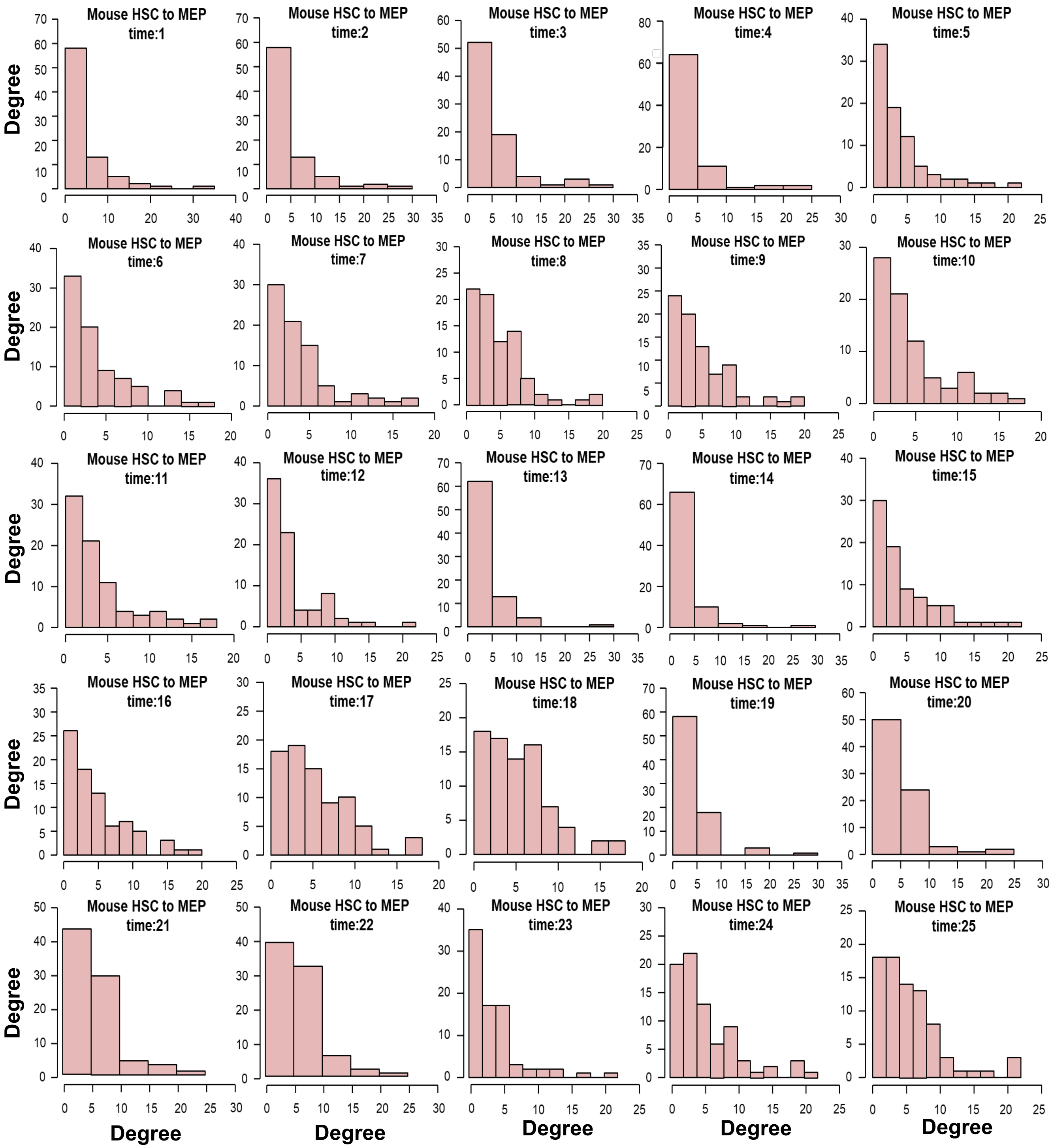

Degree distribution of networks from HSC to LMPP in mouse

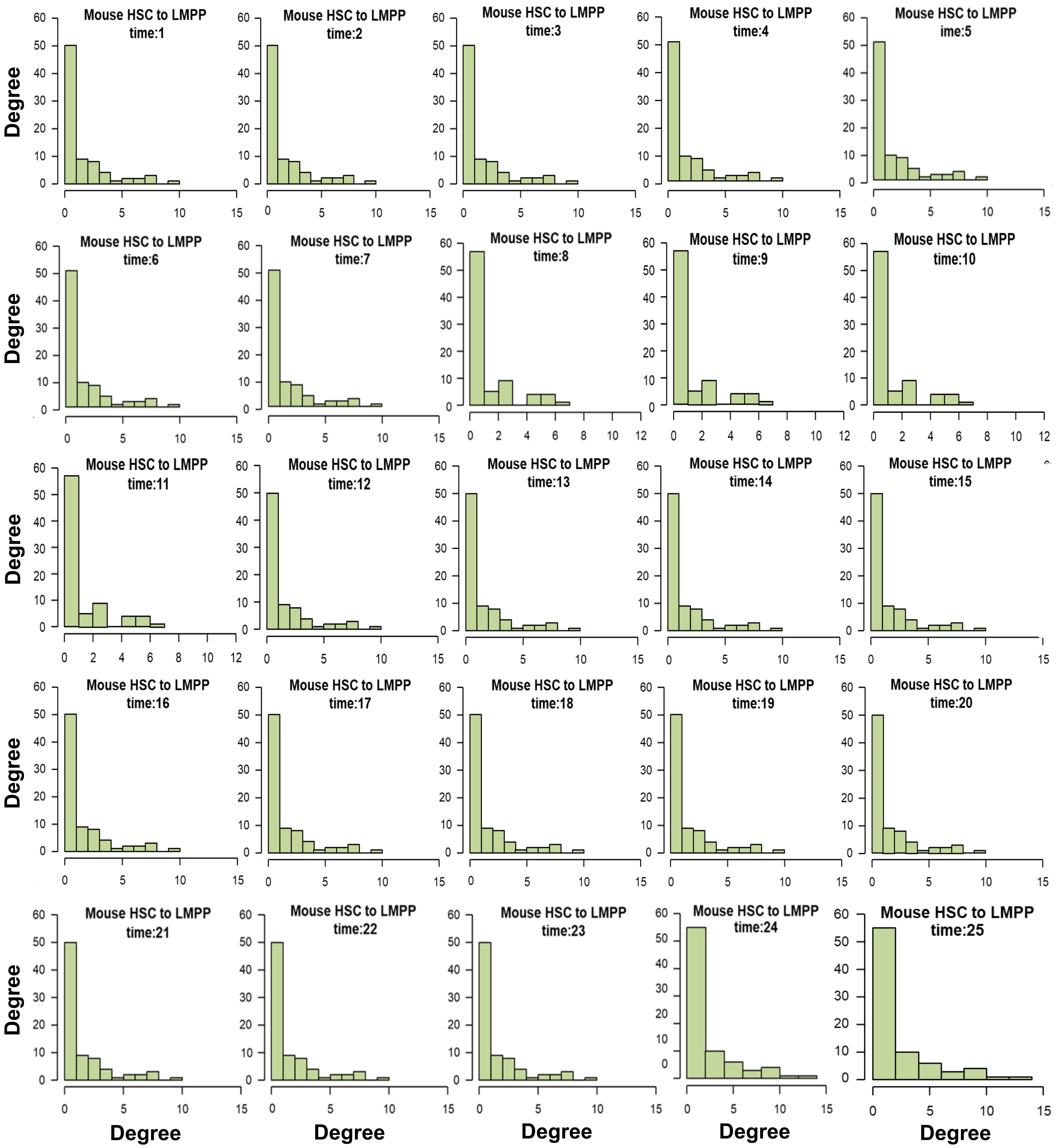

Supplement: Supplementary file 1 [file genes-13-01890-s001.zip › Supp Figure S2.pdf]
